# Global impact and cost-effectiveness of tuberculosis interventions

**DOI:** 10.1038/s44360-026-00171-5

**Published:** 2026-07-21

**Authors:** Katherine C. Horton, Alvaro Schwalb, Martin J. Harker, Lara Goscé, Elena Venero-Garcia, Lily O’Brien, Arkaprabha Gun, Tom Sumner, C. Finn McQuaid, Rebecca A. Clark, Tomos O. Prys-Jones, Roel Bakker, Yiran E. Liu, Mmamapudi Kubjane, Christian Lienhardt, Richard G. White, Rein M. G. J. Houben

**Affiliations:** 1 TB Modelling Group, TB Centre, LSHTM, London, UK; 2 Department of Infectious Disease Epidemiology, LSHTM, London, UK; 3 Instituto de Medicina Tropical Alexander von Humboldt, Universidad Peruana Cayetano Heredia, Lima, Peru; 4 Global Health Economics Centre, LSHTM, London, UK; 5 Institute for Global Health, University College London, London, UK; 6 Department of Epidemiology, Biostatistics and Occupational Health, School of Population and Global Health, McGill University, Montreal, Quebec Canada; 7 KNCV Tuberculosis Foundation, The Hague, Netherlands; 8 SEICHE Center for Health and Justice, Yale University School of Medicine, New Haven, CT USA; 9 Justice Collaboratory, Yale Law School, New Haven, CT USA; 10 Health Economics and Epidemiology Research Office, Wits Health Consortium, University of the Witwatersrand, Johannesburg, South Africa; 11 French Institute for Research in Sustainable Development (IRD), Montpellier, France; 12 FAST-TB, CRDF Global, Arlington, VA USA

**Keywords:** Tuberculosis, Epidemiology

## Abstract

While a range of interventions exists for tuberculosis (TB), their potential impact and cost-effectiveness are seldom directly compared across settings with different TB epidemiologies and structural drivers. Here we calibrated a deterministic TB model to epidemiological indicators from Brazil, India and South Africa. We implemented seven interventions across countries focusing on prevention, screening and diagnosis and treatment, as well as prison screening (Brazil) and nutritional supplementation (India). We standardized scale-up (2025–2030), coverage (80% of the target population) and strength of evidence using published efficacy data. We estimated the epidemiological impact and incremental cost-effectiveness ratios (ICERs), expressed as costs per disability-adjusted life year (DALY) averted, through 2050. Only three interventions prevented >10% of incident TB episodes by 2050: vaccination (median 15–28% across countries), symptom-agnostic community-wide screening (31–38%) and prison screening (23%). Other interventions had limited impact, ranging from 0% (shorter drug-susceptible treatment) to 5% (nutritional supplementation). ICERs varied widely by intervention and setting. Shorter drug-resistant treatment was cost-saving across settings, with the next lowest ICERs for prison screening (US$146 per DALY) and nutritional supplementation (US$281 per DALY). Interventions with meaningful epidemiological impact can be cost-effective but must target populations beyond clinic-diagnosed individuals and their households. Achieving such potential requires a shift in priorities regarding funding, policy and product development.

## Abstract (web summary)

A modelling analysis at a global scale estimates that three interventions (vaccination, community-wide screening and prison screening programmes) could prevent more than 10% of tuberculosis cases between now and 2050, highlighting the need to shift policy focus towards prevention.

## Main

An estimated 10 million people develop tuberculosis (TB) each year, resulting in 1.3 million deaths^1^. Although TB trends have shown a slow decline, recovery from TB programme disruptions following the COVID-19 pandemic is now again threatened by global funding cuts^2,3^ and potential increases in known structural drivers of TB, such as HIV, undernutrition and incarceration^4,5^.

The armamentarium against the global TB epidemic has expanded substantially in the past decade, including increased access to PCR-based tools for rapid screening and diagnosis^6^, shorter treatment for drug-susceptible TB (DS-TB)^7^ and, in particular, drug-resistant TB (DR-TB)^8^, and promising TB vaccine candidates to prevent progression to disease^9^. In addition, interventions addressing key structural drivers of TB—such as antiretroviral therapy (ART) for individuals co-infected with HIV, nutritional supplementation and systematic screening for TB in prisons—have shown efficacy in reducing disease burden^10,11^.

However, at a time of constrained budgets for TB care and management globally, an expanding toolkit presents more choices to make in terms of investments, both in research and product development, as well as in selecting interventions to implement at the national programme level. For example, policymakers must decide whether the health benefits of nutritional supplementation are worth the added implementation costs to a TB programme, while funding and research agencies must weigh the large investment required for a phase 3 vaccine trial against the costs of developing and testing new approaches to community-wide screening^9,10,12^.

Evidence of direct comparisons between such different interventions, however, is scarce. Development of new tools is largely guided by target product profiles, which often focus on the costs and benefits of a single class of interventions or a single product^13,14^. Furthermore, modelling exercises associated with target product profiles are often limited, considering only the health benefits of single products or interventions and frequently focusing on targeted subpopulations rather than the wider population^14,15^.

Also, few projects have compared the potential impact of interventions across the domains of prevention, screening and diagnosis, and treatment^16,17^. As a result, policy-directed discussions most often focus on individual intervention domains^18–21^. Lastly, most multi-intervention studies were conducted over a decade ago and reflected policy choices for a single country^16,22^, region^19^ or an aggregated global population^17^. It is unclear whether such findings would still hold true with current tools or remain valid when considering the wide geographical heterogeneity in TB burden, trends and the distribution of structural drivers of TB. In addition, these studies seldom considered costs, a critical component of policy and product development decisions. Combined with recent progress in our understanding of the natural history of TB^23^, there is a strong need to revisit the question of the potential epidemiological benefits and costs of current TB interventions.

In this study, we compared the epidemiological and budgetary impacts, as well as the cost-effectiveness, of a standardized set of interventions targeting the domains of TB prevention, screening and diagnosis, and treatment, taking into account locally relevant structural determinants.

## Results

### Calibration and baseline

**Fig. 1:**
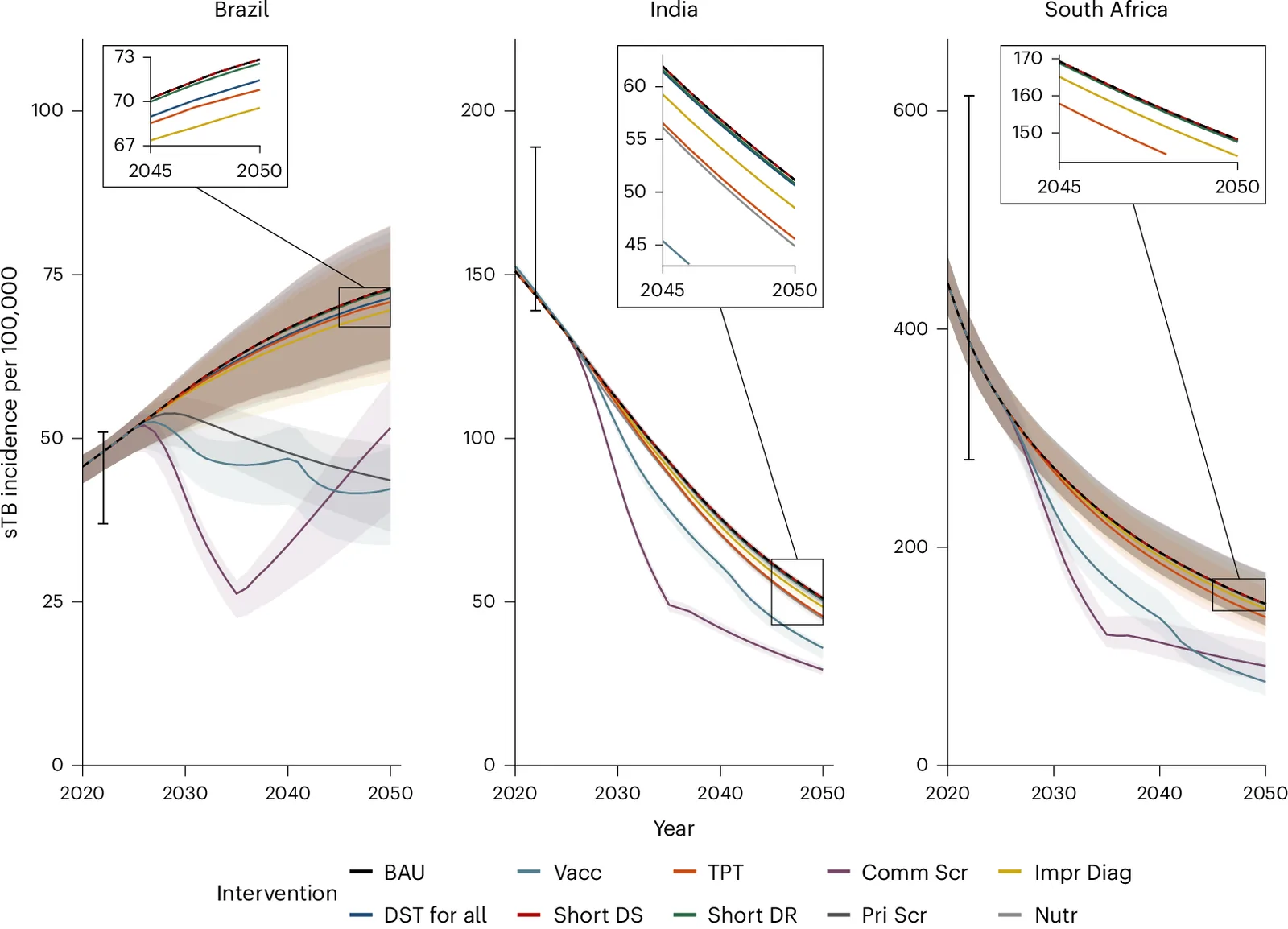
Incidence trends for BAU and interventions. Lines show incidence trends for BAU (black dashed line) and interventions (coloured lines) for each country. Shading reflects 95% UIs across 500 calibrated parameter sets. Note that UIs overlap for most interventions. Error bars indicate the 2023 incidence calibration target. Pop-outs show the last 5 years of incidence curves (without uncertainty) that lie close to the baseline (black dashed line). Vacc, vaccination; Comm Scr, community-wide screening; Impr Diag, improved diagnosis in clinics; DST for all, DST for all clinic-diagnosed individuals; short DS, shorter treatment for DS-TB; short DR, shorter treatment for DR-TB; Pri Scr, mass screening in incarcerated individuals; Nutr, nutritional support for households of individuals receiving TB treatment.

All 500 selected parameter sets were fitted to the calibration targets for our dynamic compartmental model (see Fig. 5; a detailed overview of posterior parameter ranges and calibration targets is provided in Supplementary Note 4, section 1). Figure 1 shows projected incidence trends under a business-as-usual (BAU) scenario up to 2050 (black dashed line) for each of the archetype countries (Brazil, India and South Africa), with different baseline burdens and projected trends up to 2050.

### Epidemiological impact

The coloured lines in Fig. 1 show the estimated impact of the interventions, with relatively consistent patterns between countries when comparing interventions with BAU. Most trajectories remained close to BAU, indicating a limited estimated population-level impact. Exceptions were vaccination (green line), community-wide screening (purple line) and annual systematic screening in prisons in Brazil (dark grey line).

Trajectories for community-wide screening reflected the cessation of activities in 2035. Thereafter, incidence slopes began to revert to BAU in each setting, reducing the difference with BAU by 2050. For vaccination, the catch-up campaign in 2040 was reflected in the change in trajectory. Trends in TB prevalence and mortality appeared similar (Supplementary Note 4, section 2).

**Fig. 2:**
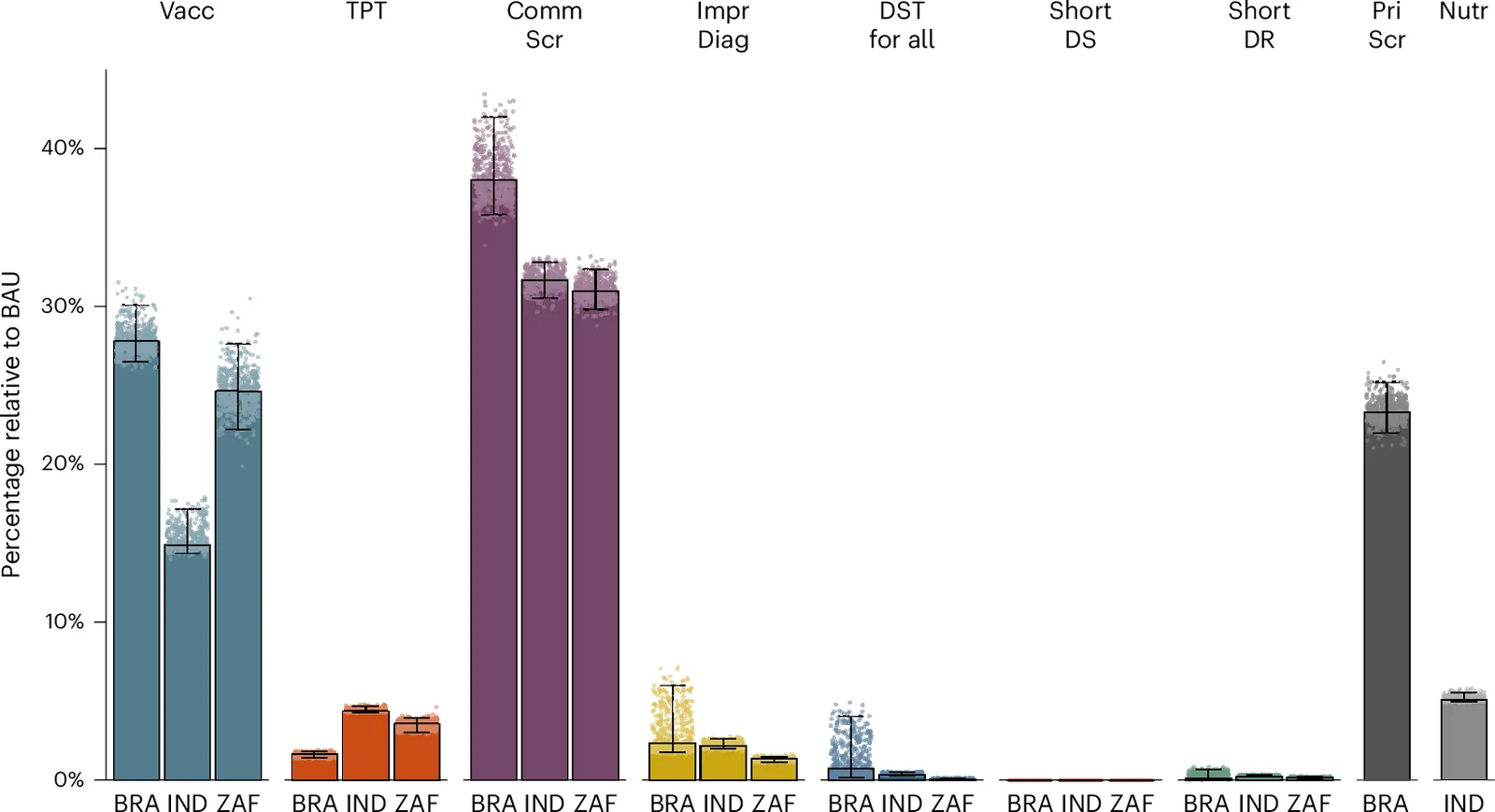
Percentage of incident sTB episodes averted (2025–2050). Bars show median estimates. Error bars reflect 95% UIs across 500 calibrated parameter sets. Dots show estimates for individual calibrated parameter sets. BRA, Brazil; IND, India; ZAF, South Africa.

When considering the difference in incidence trends between BAU and interventions, we estimated the proportion of symptomatic TB (sTB) episodes averted between 2025 and 2050 (Fig. 2 and Supplementary Note 4, section 2). We found that three interventions showed a high impact (>10% averted): vaccination (median 15–28% of sTB episodes averted across countries), community-wide screening (31–38%) and screening in prisons in Brazil (23%). Other interventions, which targeted individuals accessing care through clinics (improved diagnostic approaches in clinics, increased access to drug susceptibility testing (DST), shorter treatment for DS-TB and DR-TB) or their households (TB preventive treatment (TPT), nutritional support), were each estimated to avert fewer than 5% of total episodes in the population. For these interventions, the median percentage of sTB episodes averted ranged from 0% (shorter treatment for DS-TB) to 5% (nutritional support in India).

Proportional reduction in prevalence in 2050 showed a similar pattern (Supplementary Note 4, section 2.5), with the overall impact being similar among the vaccination, community-wide screening and prison screening interventions (median 30–49% reductions), in part driven by the rebound in prevalence following the cessation of community-wide screening in 2035.

### Budget impact and incremental costs

Total budget impact (in US dollars (USD) and local currencies) and incremental costs for the interventions are provided in the supplementary tables (Supplementary Note 4, section 3). Differences between settings were driven largely by the interaction between population size and TB burden, which determined the absolute size of the target populations.

**Fig. 3:**
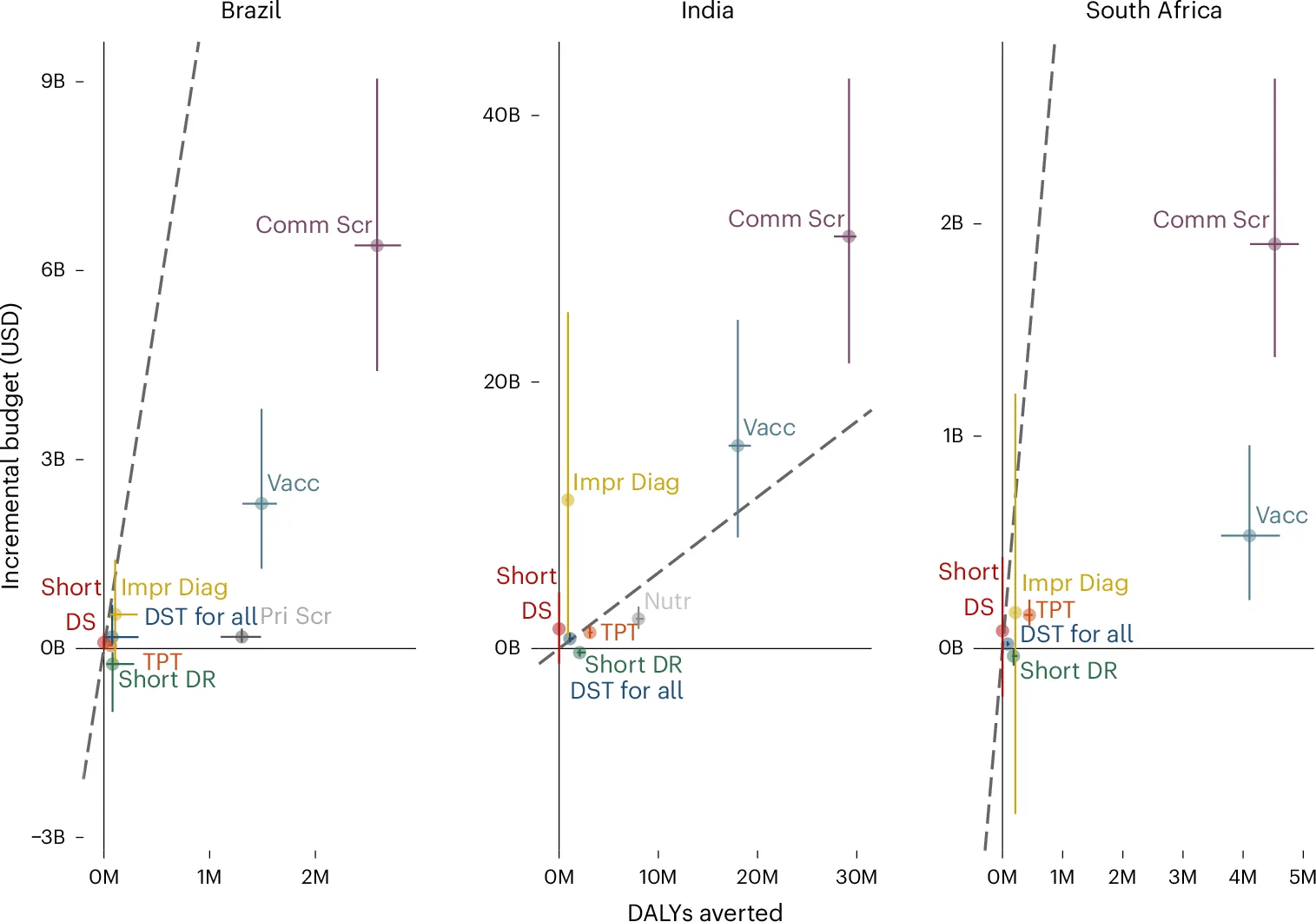
ICERs for intervention plots. Points show median estimates. Error bars show 95% UIs for DALYs averted (horizontal lines) and incremental budget (vertical lines) across 500 calibrated parameter sets. The dashed line shows the upper bound of cost-effectiveness thresholds. The plots show data for community-wide screening with low diagnostic costs. See Supplementary Note 4, section 3.4, for community-wide screening with high costs. B, billion; M, million.

Incremental costs, compared with the costs for BAU, varied widely—from cost savings (median −0.2, −0.3 and −0.04 billion USD for shorter DR-TB treatment in Brazil, India and South Africa, respectively) to over 240 billion USD (high-cost community-wide screening in India). Figure 3 shows the incremental costs of each intervention against disability-adjusted life years (DALYs) averted. Implementing prison-based screening cost only 0.18 billion more than the 1.6 billion for BAU, due to savings in TB treatment costs. Community-wide screening and vaccination had the highest incremental costs in each country, although incremental costs were 80–87% lower for low-cost community-wide screening than for high-cost community-wide screening across all settings (Supplementary Note 4, section 3.2).

### Cost-effectiveness

We present the incremental costs and DALYs averted for each intervention compared with BAU in Fig. 3. The incremental cost-effectiveness ratios (ICERs) are provided in Supplementary Note 4, section 3.3. We found that only shorter treatment for DR-TB was likely to be cost-saving (that is, had a negative incremental cost). With no epidemiological impact for shorter DS-TB treatments (following non-inferiority in trial findings^7^), this intervention exceeded the estimated cost-effectiveness threshold in all countries (Fig. 3).

Both interventions targeting key structural drivers had very low estimated ICERs, with 146 USD per DALY for prison screening in Brazil and 281 USD per DALY for nutritional support in India.

In Brazil, the ICERs fell below the estimated cost-effectiveness threshold range (6,968–10,735 USD per DALY), with 81–100% of simulations being cost-effective compared with BAU for all interventions, except for high-cost community-wide screening (ICER of 14,275 USD per DALY; 1–18% probability of being cost-effective) and shorter DS-TB treatment. In India, with a threshold range of 413–569 USD per DALY, shorter DR-TB treatment was cost-saving; nutritional supplementation had an ICER of 281 USD per DALY (99–100% probability of being cost-effective); and TPT had an ICER of 394 USD per DALY (63–98% probability of being cost-effective). All other interventions had ICERs above the threshold range and a 0–24% probability of cost-effectiveness compared with BAU. In South Africa, all interventions other than shorter DS-TB treatment produced ICERs below the threshold (2,444–3,285 USD per DALY), with a 72–100% probability of being cost-effective compared with BAU. Between countries, the ICERs for low-cost community-wide screening varied in line with the baseline prevalence of infectious TB (2,509, 1,072 and 426 USD per DALY for Brazil, India and South Africa, respectively).

### Sensitivity analyses

**Fig. 4:**
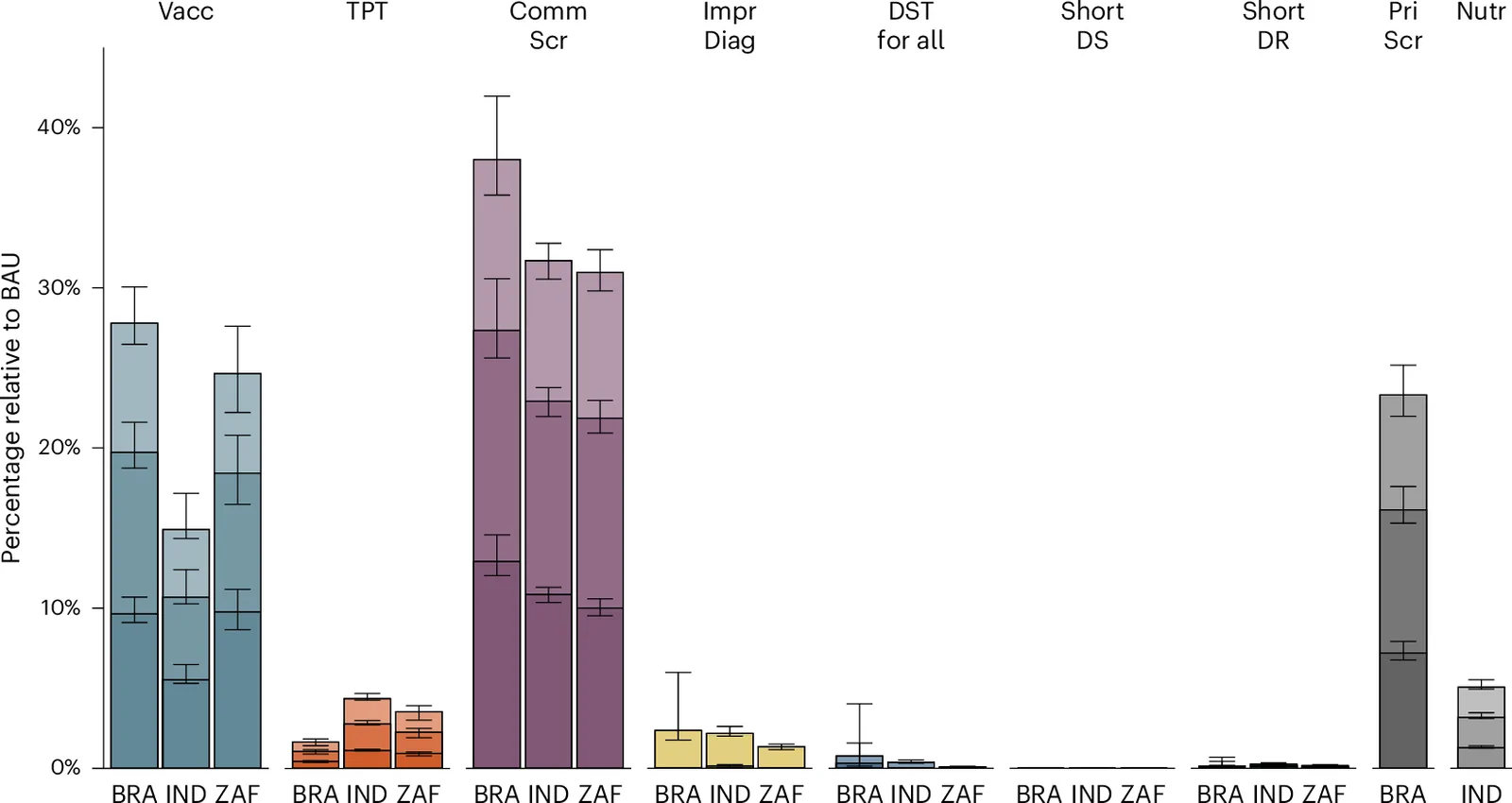
Percentage of incident sTB episodes averted (2025–2050). Data are shown for 80% (lightest shade), 50% (medium shade) and 20% (darkest shade) coverage. Bars show median estimates. Error bars reflect 95% UIs across 500 calibrated parameter sets.

Costs generally scaled with coverage, while epidemiological impacts scaled non-linearly (Fig. 4); therefore, the probabilities of an intervention being cost-effective were higher at lower coverages for some interventions (Supplementary Note 4, sections 5 and 6). Notably, implementation of vaccination, community-wide screening or screening in prisons at 20% coverage was projected to avert more incident sTB episodes than the implementation of any other intervention at 80% coverage (Fig. 4).

**Fig. 5:**
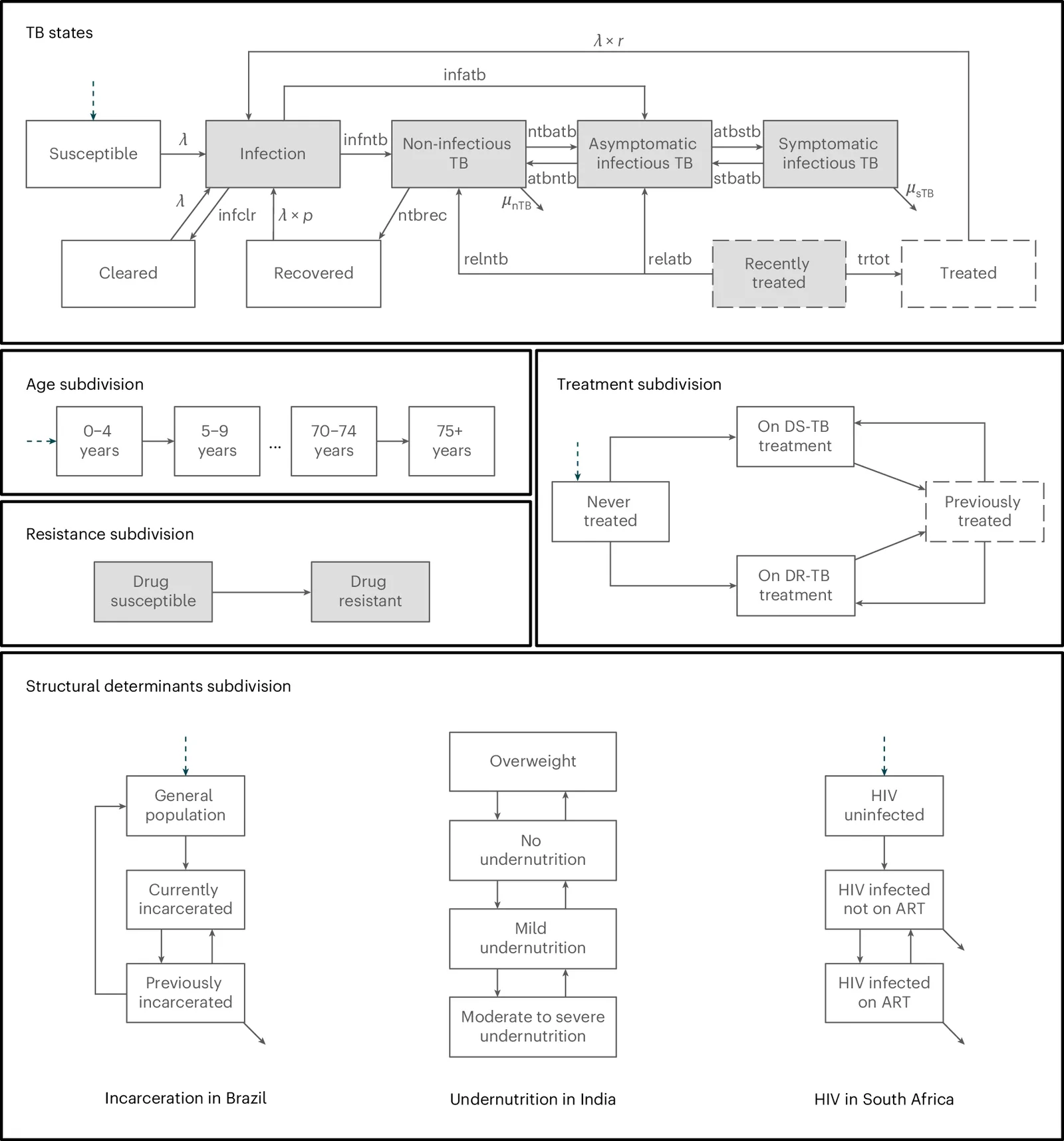
Epidemiological model structure and subdivisions. The schematic presents the model structure for the natural history of TB, incorporating both general subdivisions and country-specific structural determinants: incarceration (Brazil), undernutrition (India) and HIV (South Africa). Background mortality is assumed for all states but is not shown; arrows exiting from the corners of certain states represent specific mortality rates. The model is age-stratified into 5-year bands up to 75 years and older. Compartments shaded in grey indicate states in which individuals harbour viable *M. tuberculosis*, further subdivided by drug resistance. Compartments with dashed outlines indicate states that appear only in the previously treated stratum within the treatment subdivision. Model parameters are defined in Supplementary Note 1.

After aligning the core natural history parameters, the population impact of vaccination was similar in India and South Africa.

## Discussion

Our comparison of TB interventions found that, across settings, only community-wide screening or vaccination—and, in Brazil, also annual screening in prisons—prevented more than 10% of incident TB episodes. Other interventions targeting smaller populations (for example, TPT among household contacts (HHCs) or shorter DS-TB regimens for those diagnosed in clinics) prevented fewer than 5% of incident episodes. Cost-effectiveness ratios varied widely between interventions, but some interventions with high absolute costs (vaccination, community-wide screening) provided value for money in certain settings (Brazil and South Africa, but not India). Results were reasonably consistent across settings.

The results highlight how the potential population-level impact is largely driven by the combination of the size and disease burden of the target population, often summarized as the population attributable fraction (PAF)^24^. If an intervention is restricted to clinic-diagnosed individuals or their HHCs, the target population size imposes a hard ceiling on the potential population-level benefits, even if the within-group burden is high. If interventions prevent transmission—either through early diagnosis (community-wide or prison screening) or by preventing progression to disease (prevention-of-disease vaccines)—they can have a substantial epidemiological impact. Our results also demonstrate the importance and cost-effectiveness of addressing structural drivers of TB, with examples showing promise similar to the known benefits of ART roll-out in South Africa^25,26^. Considering costs as part of intervention evaluation highlighted the benefits of reducing regimen costs (shorter DR-TB treatment) and diagnostic algorithm costs (low-cost community-wide screening). Conversely, if an intervention with limited epidemiological benefit (for example, a non-inferior shorter DS-TB treatment) also has a higher cost, it will struggle to be cost-effective.

Our country archetypes provide contrasting future projections under BAU, driven primarily by the persistence or continued reduction of impacts from key structural drivers: increasing incarceration trends in Brazil, continued high coverage of ART in South Africa and reductions in undernutrition in India. While future trends in structural drivers and their impact are highly uncertain in the current global funding landscape^2,20^, the relative consistency of results across different background trajectories suggests that our findings are robust.

Our results update previous comparisons across intervention domains^16,17^. Some findings align, including the importance of addressing disease and transmission at a population level^17^. A key contrast is the projected population-level impact of new shorter treatment regimens, particularly for DS-TB; previous projections are driven largely by assumed improvements in treatment outcomes, whereas empirical evidence has demonstrated only non-inferiority^13,16^. The low cost and high microbiological success of current DS-TB regimens in trial settings (~95% treatment success in the standard-of-care group^27^) leave little room for assuming or obtaining empirical evidence of improvements or cost savings from new regimens. For TPT, our estimates of the population-level epidemiological impact of HHC-targeted TPT were similar to those in other studies^28,29^, whereas we did not model mass TPT for complete populations^17^.

As with any modelling exercise, there are limitations to our work. Our choice to compare interventions using empirical evidence of impact may constrain the apparent potential of certain approaches and overlook the gap between trial efficacy and real-world effectiveness^30^. However, going beyond the current evidence would risk introducing subjective imbalances in the assumed potential efficacy between intervention categories and would limit a more objective comparison of potential population-level health benefits and costs. We did not consider operational limitations to achieving 80% coverage, which many interventions have not yet reached outside of research settings^12,31^, nor did we consider the differential costs that may be required to overcome operational challenges for each intervention when scaling up from current levels. Rather than overlooking operational challenges, our results highlight the relative potential of interventions and the value of resolving logistical challenges to unlock the epidemiological benefits of an intervention or reduce costs.

In our economic analyses, although we used local costs where possible, some health system costs may have been omitted. As such, absolute values for budget impact should be interpreted with caution. However, the level of detail should be comparable across intervention categories, enabling meaningful comparisons. We also did not consider patient costs, which limited the quantification of potential cost benefits for individual patients rather than for society as a whole. However, cost and DALY savings are driven predominantly by the prevention of new incident TB episodes, which was our primary outcome, rather than by savings from the management of existing TB, suggesting that our broad findings are robust to this consideration. Countries included in our analysis represent lower-middle-income (India) and upper-middle-income (South Africa and Brazil) nations. Cost-effectiveness may vary in low-income settings.

Our exercise only considered each intervention compared with BAU and did not explore independent improvements in BAU, such as increased clinic attendance or other changes in structural determinants of TB (for example, long-acting antiretrovirals for HIV in South Africa). We also did not model intervention combinations where potential synergies are quantified, such as the benefit of shorter DS-TB treatments in the context of community screening, or a staggered introduction of community-wide screening followed by vaccination, which might have shown additional benefits for one or more of the interventions.

Despite these limitations, our work provides a much-needed perspective on the potential impact of interventions across the TB response portfolio. We show that interventions need to reach populations with substantial contributions to the TB burden (as quantified by the PAF) in order to make progress in preventing new TB episodes and reducing mortality at the population level. This point has been made before^32–34^, but our results provide a stark picture of the continued morbidity and mortality we implicitly accept if we fail to extend our interventions beyond current patients with TB and their HHCs. Our incremental budget results highlight that this requires a different level of ambition in terms of funding, but also that such investments probably provide good value for money as well as meaningful population-level impact.

Community-wide screening was highly impactful but also had the greatest budgetary impact. While below the cost-effectiveness threshold in high-burden populations, the ICER increased as the burden decreased. Community-wide screening in settings such as Brazil, where the estimated incidence is at or below 50 per 100,000, is therefore unlikely to be the most efficient approach. Our work shows that other, less costly interventions may also achieve substantial epidemiological impact in such settings. Here, a combination of lower-cost, high-impact interventions, such as community-wide vaccination and screening targeted at a high-risk population (for example, incarcerated individuals), may be more appropriate^11,35^. For higher-burden settings, identifying cost savings for screening approaches is key—for example, simplifying algorithms to a single low-cost bacteriological test with performance comparable to current PCR tools, or identifying more efficient symptom-agnostic screening approaches^36,37^. In addition, linking such efforts with vaccination campaigns (either in parallel or sequentially) could ensure that impacts are sustained after the cessation of community-wide screening at a set burden threshold. Such multifaceted approaches—combining vaccination, mass screening and the treatment of multiple disease phenotypes—have historically been shown to have an impact in high-burden settings, most notably among First Nation populations in Alaska during the mid-twentieth century^33,38^.

Our work demonstrates the value of an approach that compares different interventions across the domains of prevention, screening and diagnosis, and treatment when considering priorities for research and funding. In addition, the development of target product profiles should look beyond the benefits for individuals or a limited target population and also consider the desired population-level impact, as well as the improvements over current performance needed to achieve that impact. That should include how existing interventions can or should be adapted for performance in recently defined TB phenotypes and outcomes, such as asymptomatic TB (aTB) among community-diagnosed individuals, or the risk of post-TB lung disease, which is a major driver of TB-related morbidity^39^.

Overall, our results show that interventions with meaningful impact can also be cost-effective, but achieving their potential may require a shift in priorities in public health funding, policy and product development.

## Methods

### Epidemiological archetypes

We considered three country archetypes to explore the robustness of our findings across diverse geographies and TB epidemiologies, including current burden, historical trends and structural drivers. We selected Brazil, India and South Africa, where the estimated TB incidence in 2023 ranged between 49 and 427 per 100,000 population^1^. In each country, we identified the structural determinant with the highest estimated PAF: incarceration (36.9%) in Brazil^5^, undernutrition (13–45.2%) in India^1,40^ and HIV (49%) in South Africa^1^.

### Principles of intervention comparison

As our objective was to provide a standardized comparison of TB interventions across the domains of prevention, screening and diagnosis, and treatment, we used a set of principles to guide the choice of interventions within each category and their implementation in the model. First, interventions were included based on the availability of published empirical evidence for the target population, epidemiological mechanisms and efficacy. This, for example, excluded products with as-yet-unknown efficacy, such as the recently proposed pan-TB treatment^15^. Second, we standardized implementation where possible, aligning the start year (2025), scale-up duration (2025–2030) and shape (linear), as well as the coverage target (80% of the target population) and the time horizon to accumulate potential indirect effects (2030–2050). Finally, coverage was assumed to apply to the entire process—for example, from initiation to the completion of TPT. While this is a simplification of existing operational challenges^41^ or availability (for example, TPT or TB vaccination), it enables a full comparison of potential epidemiological impact rather than a mix of potential impact and operational constraints specific to individual interventions.

### Choice and data on interventions

In each country, we compared interventions covering key domains. For the prevention domain, this included a prevention-of-disease vaccine^9^ and TPT^42^. Within the screening and diagnosis domain, we included a symptom-agnostic community-wide screening intervention^12^, expanded new diagnostic tools in TB clinics^6^ and expanded DST^6^. For the treatment domain, we included the introduction of a shorter, non-inferior (that is, equally effective) regimen for DS-TB^7^ and a shorter, more efficacious regimen for DR-TB^8^. Interventions targeting the country-specific structural drivers included annual symptom-agnostic screening of incarcerated individuals in Brazil^11^ and the nationwide implementation of the recently completed RATIONS nutritional intervention in India^10^. We did not model a separate intervention for South Africa, as the key intervention addressing HIV (wide availability of ART) is already implemented, nor did we model TPT for people living with HIV, given the high initiation rates recently achieved in South Africa^1^. Table 1 describes the interventions for each category. Full details, including the test positivity of diagnostic algorithms, can be found in Supplementary Note 2, section 2.

**Table 1:** Interventions included

Domain + category	Intervention	Target population + coverage^a^	Epidemiological impact^b^	Per-person cost^c^	References
**Prevention**					
Vaccination	Prophylactic prevention-of-disease vaccine for individuals ≥15 years old	Target population: all adults (≥15 years old) Coverage: 80% of unvaccinated 15–19 year olds annually; mass campaign to reach 80% of ≥20 year olds by 2030 with a catch-up campaign in 2040	50% reduction in progression to infectious disease, effective in both uninfected and infected people, with waning efficacy after 10 years	US$10.50	^9^
TPT	WHO policy for assessing HHCs	Target population: HHCs of individuals started on TB treatment Coverage: 80% of child (<15 years old) HHCs receive TPT and 80% of all HHCs assessed for TB	TPT: 60% reduction in progression to disease among recently exposed children HHC screening: 3% of HHCs have TB disease; these are identified and treated	US$24.00–37.00 for HHC assessment; US$22.00–84.00 for TPT for DS-TB; US$41.00–149.50 for TPT for DR-TB	^42,63^
**Screening and diagnosis**					
Community-wide screening	Annual symptom-agnostic community-wide screening using CXR, followed by Xpert Ultra if positive	Target population: all adults (≥15 years old) Coverage: scale-up to 80% in 2025–2030; five annual rounds covering 80% of adults; complete stop after 2035	Early diagnosis of bacteriologically confirmed TB Increased DST coverage Lower on-treatment mortality due to earlier treatment initiation	US$41.50 for CXR screening (high-cost estimate) or US$2.50–5.50 for CXR screening (low-cost estimate); US$17.00–24.50 for Xpert Ultra	^12,36,43^
Improved diagnostic approaches in clinics	Xpert Ultra as part of the TB diagnostic pathway in clinics, replacing CXR and empirical diagnosis	Target population: individuals (all ages) with presumptive TB entering the TB diagnostic pathway in clinics Coverage: 80%	Increased sensitivity for bacteriologically confirmed TB Increased specificity for non-disease states Increased DST coverage	US$24.00–37.00 (versus US$13.50–31.00 in BAU)	^6,64^
DST	Xpert Ultra for all individuals diagnosed with TB in clinics, to detect rifampicin resistance	Target population: individuals diagnosed with TB in clinics receive an Xpert Ultra test. Coverage: 80%	Increased initiation of appropriate rifampicin-resistant treatment Reduced on-treatment mortality for DR-TB due to earlier initiation	US$17.00–24.50	^6^
**Treatment**					
Shorter treatment for DS-TB	WHO-recommended 4-month treatment for DS-TB	Target population: individuals initiating DS-TB treatment Coverage: 80%	Treatment duration shortened from 6 to 4 months Non-inferior treatment success	US$199.00–276.00 (versus US$119.50–227.50 in BAU)	^7^
Shorter treatment for DR-TB	WHO-recommended 6-month treatment for rifampicin-resistant TB	Target population: individuals initiating treatment for rifampicin-resistant TB Coverage: 80%	Treatment duration shortened to 6 months Treatment success increased to 89%, including reduced on-treatment mortality	US$394.50–502.50 (versus US$450.50–3,600.50 in BAU)	^8,65,66^
**Structural drivers**					
Incarceration (Brazil only)	Annual symptom-agnostic screening for all incarcerated individuals, using Xpert Ultra	Target population: currently incarcerated individuals Coverage: 80%	Early diagnosis of bacteriologically confirmed TB Increased DST coverage Lower on-treatment mortality due to earlier treatment initiation	US$21.50	^11^
Undernutrition (India only)	Nutritional supplementation for patients with TB and their HHCs (RATIONS study)	Target population: all individuals initiating TB treatment and their HHCs Coverage: 80%	Patients with TB: 33% reduced risk of on-treatment mortality HHCs: 39% reduced risk of progression to disease	US$16.50 for patients with TB; US$6.00 for HHCs	^10,21^

^a^See the figures in Supplementary Note 4, section 1, for the shape of scale-up and coverage for each intervention.

^b^See Supplementary Note 2, section 2, for data and methods.

^c^Ranges indicate values across countries. See Supplementary Note 3, section 3.2, for data and methods for cost sources.

Scale-up and coverage scenarios are illustrated in Supplementary Note 4, section 1. Intervention coverage was scaled up between 2025 and 2030 and then kept constant. For TB vaccines, we implemented routine vaccination of unvaccinated adolescents aged 15–19 years and two mass catch-up campaigns for adults aged ≥20 years (at scale-up and in 2040)^20^. Mass community-wide screening would not be maintained for 25 years, as historical and contemporary data suggest that its impact will diminish over time^12,43^. Instead, we assumed a 5-year scale-up to 80%, followed by five annual campaigns, after which screening would stop.

### Epidemiological model

To estimate the potential epidemiological impact of all selected interventions, we developed a dynamic transmission model that extends an existing modelling infrastructure used to explore TB vaccine impact^20^, incorporating previously published structure and parameters for TB natural history^44^. The model was age-stratified, with different TB natural history parameters for progression, diagnosis, infectivity and mortality in children (<15 years old) and adults (Supplementary Note 1, section 2.3).

The model’s core natural history structure and subdivisions are shown in Fig. 5 and described in detail (with parameterization) in Supplementary Note 1. We recognize three disease states, aligning with the recent recategorization by the World Health Organization (WHO)^23,45^. In brief, the model captures two bacteriologically positive infectious states, differentiated by symptom reporting (sTB and aTB), both of which contribute to transmission. Bacteriologically negative, non-infectious TB (nTB) does not contribute to transmission and includes individuals with TB-related pathology detectable on imaging—for example, chest X-ray (CXR)—but without bacteriological evidence of TB, although a proportion will progress to bacteriologically confirmed TB^46^. In addition, the model recognizes self-clearance of *Mycobacterium tuberculosis* infection, as well as regression and recovery from disease states. Extrapulmonary TB was not modelled explicitly but was included within the nTB state. The model was further subdivided to include treatment and infection with drug-susceptible or rifampicin-resistant strains.

We implemented diagnostic pathways for individuals undergoing screening and diagnosis in both clinic- and community-based settings. We explicitly considered individuals without TB entering the diagnostic pathways, with test performance (expressed as test positivity) specified across the different model states. See Supplementary Table 3 in Supplementary Note 1, section 1.3, for values and data sources.

Structural drivers were included to capture key population differences by epidemiological risk and intervention impact, including incarceration (not incarcerated, currently incarcerated, recently incarcerated—that is, individuals who have been released from prison within the past 7 years) in Brazil; nutritional status (body mass index (BMI) of <17, 17–18.4, 18.5–24.9 and >25 kg m^−2^ (adult thresholds)) in India; and HIV status (HIV-negative, HIV-positive, HIV-positive and receiving ART) in South Africa. Data on subgroup sizes and trends are described in detail in Supplementary Note 1, sections 4–6. Parameterization of natural history was based on a review of published data and modelling^5,47–49^.

Heterogeneous mixing was implemented by 5-year age groups up to 75 years and older^50^. In addition, we incorporated assortative mixing by incarceration status and strongly increased mixing among incarcerated individuals to reflect known issues of overcrowding^51^ (Supplementary Note 1, section 6.5).

### Economic model

To estimate the budgetary impact, incremental costs and cost-effectiveness, we constructed a costing model that linked the intervention activities and epidemiological outcomes to costs. This included costs for existing TB diagnosis and treatment services, for which demand was altered by the interventions, as well as the costs of providing each intervention from 2025 to 2050. See Supplementary Note 3 for details on the economic model.

Costs were sourced for each country based on previous studies of these interventions or publicly available data sources^10,20,52–57^(see Supplementary Note 3, section 3.2, for details). We used a health system perspective, thus providing results relevant to public health funders. All costs are expressed in 2023 USD. We used a standard 5% annual discount rate for costs and benefits across all countries^58^. For community-wide screening, we used both high- and low-cost scenarios for the diagnostic tools due to the wide discrepancy in estimated diagnostic costs between countries^53,55,56^(Supplementary Note 3, section 3.2.1). To capture uncertainty in costs, we generated a gamma distribution around each point estimate using a conservative range of ±50%, from which we sampled 500 cost sets for each country. Each cost set was randomly matched to a parameter set from the epidemiological model to estimate the costs of the activities. Estimated cost-effectiveness threshold ranges described by Ochalek et al.^59^ in 2018 were adopted and updated to 2023 gross domestic product values^59^ (Supplementary Note 3, section 2.6).

### Model calibration

For each epidemiological archetype, we calibrated the model to trends in TB incidence, notifications and mortality in 2010 and 2023, as reported by the WHO. In addition, we calibrated to the prevalence and the proportion of aTB where available (India and South Africa). We also calibrated the model to indicators of structural drivers, including incarceration rate and notification rate among incarcerated individuals (Brazil); PAF for BMI under 18.5 kg m^−2^ (India); and TB incidence, notification and mortality among HIV-positive individuals (South Africa). A full list of calibration targets, data sources and methods is provided in Supplementary Note 1, section 7.

Calibration was performed by seeding an approximate Bayesian computation–Markov chain Monte Carlo (ABC-MCMC) procedure with a variable step size, followed by manual fitting to all calibration targets. After ABC-MCMC identified at least 25,000 additional parameter sets consistent with all calibration targets, we selected 500 at random.

Models were calibrated up to 2025, after which we generated the BAU comparator scenario by keeping all parameters constant from 2025, except for projections of HIV and ART prevalence in South Africa (based on projections by the Joint United Nations Programme on HIV/AIDS) and changes in BMI distribution in India (based on ref. ^60^). The BAU scenario was used for comparison against interventions, each of which was run across all 500 parameter sets per epidemiological archetype, providing paired estimates of epidemiological impact.

### Model outcomes

For each intervention, we generated trends in infectious TB prevalence (aTB + sTB) to match current disease measurements from prevalence surveys, as well as incident sTB episodes, TB deaths and DALYs averted compared with BAU up to 2050. DALYs were calculated based on published disability weights for sTB^61^ and life years lost due to TB deaths, and included post-TB DALYs incurred after treatment or recovery from sTB (in the absence of DALY estimates available for aTB)^39^. See Supplementary Note 3, section 4, for more details. We calculated the budgetary impact as the total costs for each intervention over the 2025–2050 period, as well as the incremental costs and ICERs, expressed as cost per DALY averted relative to BAU. We generated median estimates for the results, with corresponding 95% uncertainty intervals (UIs) calculated as the 2.5th to 97.5th percentiles of the parameter sets, to quantify uncertainty.

### Sensitivity analyses

In addition to the high-coverage scenarios presented in the main text, we also examined the potential impact and cost-effectiveness of interventions implemented at lower coverage levels of 50% and 20%. We also explored the epidemiological impact of between-country variation in the parameterization of natural history by assuming the same natural history parameters across countries (disregarding calibration targets).

### Ethics and inclusion

The research presented in this article did not involve human participants and, therefore, did not require review by any research ethics committee. All data used for model parameterization and calibration were obtained from publicly available, published sources. Sources and the validity of local epidemiological and cost data were discussed with researchers from each country, as reflected in the authorship and acknowledgements.

### Reporting summary

Further information on research design is available in the Nature Portfolio Reporting Summary linked to this article.

## Supplementary information

Supplementary Information Supplementary Notes 1–4.

Reporting Summary

Peer Review File

## Data Availability

Data are available at https://doi.org/10.17605/OSF.IO/ZYKSD (ref. ^62^).

## Code availability

Analysis scripts are available at https://doi.org/10.17605/OSF.IO/ZYKSD (ref. ^62^) and via GitHub at https://github.com/lshtm-tbmg/PACE-TB.
