# Supplementary material for: Global impact and cost-effectiveness of tuberculosis interventions
Source: Nat Health. 2026 Jul 21;1(11):1320–30. doi: 10.1038/s44360-026-00171-5 (PMC13727693; doi:10.1038/s44360-026-00171-5)
Supplement: Supplementary file 1 [file 44360_2026_171_MOESM1_ESM.pdf]

nature health

Supplementary information https://doi.org/10.1038/s44360-026-00171-5

Global impact and cost-effectiveness of tuberculosis interventions

In the format provided by the authors and unedited

TABLE OF CONTENTS

S1 MODEL STRUCTURE 7

1 TB DIMENSION 7
1.1 OVERVIEW 7
1.2 STRUCTURE 8
1.2.1 NATURAL HISTORY 9
1.2.2 DIAGNOSIS AND TREATMENT 11
1.2.3 DRUG RESISTANCE 14
1.3 PARAMETERS 18
1.4 DIFFERENTIAL EQUATIONS 27
1.4.1 EXPRESSIONS 27
1.4.2 NEVER TREATED 27
1.4.3 PREVIOUSLY TREATED 28
1.4.4 ON TREATMENT 29
2 AGE DIMENSION 32
2.1 OVERVIEW 32
2.2 STRUCTURE 32
2.3 INTERACTION BETWEEN AGE AND TB DIMENSION 32
2.4 EQUATIONS 33
2.5 PARAMETERS 33
3 RISK DIMENSION: HIV IN SOUTH AFRICA 35
3.1 OVERVIEW 35
3.2 STRUCTURE 35
3.4 EQUATIONS 38
3.4.1 EXPRESSIONS 39
3.4.2 HIV DIMENSION EQUATIONS 39
3.4.3 NEVER TREATED 39
3.4.4 PREVIOUSLY TREATED 40
3.5 PARAMETERS 42

4 RISK DIMENSION: NUTRITION IN INDIA 45
4.1 OVERVIEW 45
4.2 STRUCTURE 45
4.3 INTERACTION BETWEEN NUTRITION AND TB DIMENSION 46
4.4 EQUATIONS 47
4.4.1 EXPRESSIONS 47
4.4.2 NUTRITION DIMENSION EQUATIONS 47
4.4.3 NEVER TREATED 47
4.4.4 PREVIOUSLY TREATED 48
4.5 PARAMETERS 50
5 RISK DIMENSION: INCARCERATION IN BRAZIL 53
5.1 OVERVIEW 53
5.2 STRUCTURE 53
5.3 INTERACTION OF PRISONS AND TB DIMENSION 54
5.4 EQUATIONS 54
5.4.1 EXPRESSIONS 54
5.4.2 PRISON DIMENSION EQUATIONS 55
5.4.3 NEVER TREATED 55
5.4.4 PREVIOUSLY TREATED 57
5.5 PARAMETERS 59
6 CALIBRATION 63
6.1 CALIBRATION TARGETS 63
7 REFERENCES 68

S2 INTERVENTIONS 79

1. PRINCIPLES 79
2. INTERVENTIONS 79
2.1 BUSINESS-AS-USUAL 80
2.1.1 BUSINESS-AS-USUAL: BRAZIL 80
2.1.2 BUSINESS-AS-USUAL: INDIA 83

2.1.3 BUSINESS-AS-USUAL: SOUTH AFRICA 85
2.2 VACCINATION 88
2.3 TB PREVENTIVE TREATMENT 91
2.4 NUTRITION 95
2.5 COMMUNITY-WIDE SCREENING 98
2.6 IMPROVED DIAGNOSIS 102
2.7 DRUG SUSCEPTIBILITY TESTING 105
2.8 SCREENING IN PRISONS 108
2.9 SHORTER DS-TB TREATMENT 111
2.10 SHORTER DR-TB TREATMENT 114
3. SCALE-UP 117
4 REFERENCES 118

S3 HEALTH ECONOMIC MODEL 122

1 ECONOMIC ANALYSES 122
2 ECONOMIC PRINCIPLES 123
2.1 PERSPECTIVE 123
2.2 TIME HORIZON 123
2.3 DISCOUNTING 123
2.4 CURRENCIES AND COST YEAR 124
2.5 COMPOSITE MEASURES OF TOTAL HEALTH 124
2.6 COST-EFFECTIVENESS THRESHOLDS 124
3 RESOURCE USE AND COSTS 125
3.1 RESOURCE USE 125
3.2 COSTS 128
3.2.1 COSTS PER COMPONENT 128
3.2.2 COSTS PER ACTIVITY 130
3.2.3 COSTS PER INTERVENTION 131
4 HEALTH EFFECTS 133
4.1 DALY CALCULATIONS 133

4.2 POST-TB SEQUELAE 134
4.3 DISABILITY WEIGHTS 135
4.4 LIFE YEARS 135
5 REFERENCES 137

S4 RESULTS 140

1 CALIBRATION PLOTS AND POSTERIORS 141
1.1 CALIBRATION PLOTS 141
1.1.1 Brazil calibration results 141
1.1.2 India calibration results 142
1.1.3 South Africa calibration results 143
1.2 CALIBRATION POSTERIORS 144
1.2.1 Brazil calibration posteriors 144
1.2.2 India calibration posteriors 145
1.2.3 South Africa calibration posteriors 146
2 EPIDEMIOLOGICAL IMPACT 147
2.1 TB prevalence trends 147
2.2 TB mortality trends 148
2.3 Incident sTB episodes averted - proportion of total 149
2.4 Incident sTB episodes averted - absolute numbers (1000s) 150
2.5 Reduction in TB prevalence in 2050 - proportional (graph) 151
2.6 Reduction in TB prevalence in 2050 - proportional (table) 152
2.7 TB deaths averted - proportion of total (graph) 153
2.8 TB deaths averted - proportion of total 154
2.9 TB deaths - absolute numbers (1000s) 155
2.10 Disability-adjusted life years - total (millions) 156
2.11 Disability-adjusted life years averted compared to BAU (millions) 157
3 ECONOMIC IMPACT 158
3.1 Total cost of each intervention 158
3.2 Incremental cost of each intervention compared to BAU 159

3.3 Cost–utility analysis by country 160
3.3.1 Brazil 160
3.3.2 India 162
3.3.3 South Africa 164
4 SENSITIVITY ANALYSIS: ALIGNED PARAMETERS 167
4.1 Aligned regression and progression parameters between India and South Africa 167
5 SENSITIVITY ANALYSIS: 50% COVERAGE 168
5.1 TB prevalence trends [50% coverage] 168
5.2 TB mortality trends [50% coverage] 169
5.3 Incident sTB episodes averted - proportion of total (graph) [50% coverage] 170
5.4 Incident sTB episodes averted - proportion of total (table) [50% coverage] 171
5.5 Incident sTB episodes averted - absolute numbers (1000s) [50% coverage] 172
5.6 Reduction in TB prevalence in 2050 - proportional (graph) [50% coverage] 173
5.7 Reduction in TB prevalence in 2050 - proportional (table) [50% coverage] 174
5.8 TB deaths averted - proportion of total (graph) [50% coverage] 175
5.9 TB deaths averted - proportion of total [50% coverage] 176
5.10 TB deaths - absolute numbers (1000s) [50% coverage] 177
5.11 Disability-adjusted life years - total (millions) [50% coverage] 178
5.12 Disability-adjusted life years averted compared to BAU (millions) [50% coverage] 179
5.13 Total cost of each intervention [50% coverage] 180
5.14 Incremental cost of each intervention compared to BAU [50% coverage] 181
5.15 Cost–utility analysis by country [50% coverage] 182
5.15.1 Brazil [50% coverage] 182
5.15.2 India [50% coverage] 184
5.15.3 South Africa [50% coverage] 186
5.16 Cost-effectiveness planes for all interventions (including high-cost community-wide screening) [50% coverage] 188
5.17 Cost-effectiveness planes for all interventions (excluding high-cost community-wide screening) [50% coverage] 189
6 SENSITIVITY ANALYSIS: 20% COVERAGE 190
6.1 TB prevalence trends [20% coverage] 190
6.2 TB mortality trends [20% coverage] 191
6.3 Incident sTB episodes averted - proportion of total (graph) [20% coverage] 192

6.4 Incident sTB episodes averted - proportion of total (table) [20% coverage] ... 193
6.5 Incident sTB episodes averted - absolute numbers (1000s) [20% coverage] ... 194
6.6 Reduction in TB prevalence in 2050 - proportional (graph) [20% coverage] ... 195
6.7 Reduction in TB prevalence in 2050 - proportional (table) [20% coverage] ... 196
6.8 TB deaths averted - proportion of total (graph) [20% coverage] ... 197
6.9 TB deaths averted - proportion of total [20% coverage] ... 198
6.10 TB deaths - absolute numbers (1000s) [20% coverage] ... 199
6.11 Disability-adjusted life years - total (millions) [20% coverage] ... 200
6.12 Disability-adjusted life years averted compared to BAU (millions) [20% coverage] ... 201
6.13 Total cost of each intervention [20% coverage] ... 202
6.14 Incremental cost of each intervention compared to BAU [20% coverage] ... 203
6.15 Cost–utility analysis by country [20% coverage] ... 204
6.15.1 Brazil [20% coverage] ... 204
6.15.2 India [20% coverage] ... 206
6.15.3 South Africa [20% coverage] ... 208
6.16 Cost-effectiveness planes for all interventions (including high-cost community-wide screening) [20% coverage] ... 210
6.17 Cost-effectiveness planes for all interventions (excluding high-cost community-wide screening) [20% coverage] ... 211

S1 MODEL STRUCTURE

1 TB DIMENSION

This section aims to describe the TB dimension in TBMod. The TB dimension is the cornerstone of the structure encompassing the natural history of TB, while acknowledging treatment history and drug resistance. TBMod has been under development for ~7 years, and is an adaptation of TBVax informed by work on the natural history of TB (Clark, Mukandavire, et al., 2023; Horton et al., 2023; Portnoy, Arcand, et al., 2023; Portnoy, Clark, Quaife, et al., 2023; Portnoy, Clark, Weerasuriya, et al., 2023; Richards et al., 2023).

1.1 OVERVIEW

- The TB dimension contains a total of 49 states describing natural history, diagnosis and treatment, and drug resistance (**Figure S1**). At its core, 25 states describe the natural history, with some states being replicated, as necessary, to reflect individuals never treated (_N) and those previously treated (_P), as well as individuals with drug-susceptible infection or disease (_s_) or drug-resistant infection or disease (_r_). A further 24 states describe individuals on treatment, accounting for the state from which an individual was diagnosed and whether an individual is placed on standard first-line treatment (ST_) or second-line treatment for drug resistant TB (RT_).
- As an overview of **Figure S1**, individuals never treated (_N) can be placed on treatment and after completion will move on to become individuals previously treated (_P), which can be placed on treatment once again.
- Further details on structure are shown in the next section. Components of the model structure - natural history, diagnosis and treatment, and drug resistance - are discussed in subsequent sections with simplified model structure diagrams.

1.2 STRUCTURE

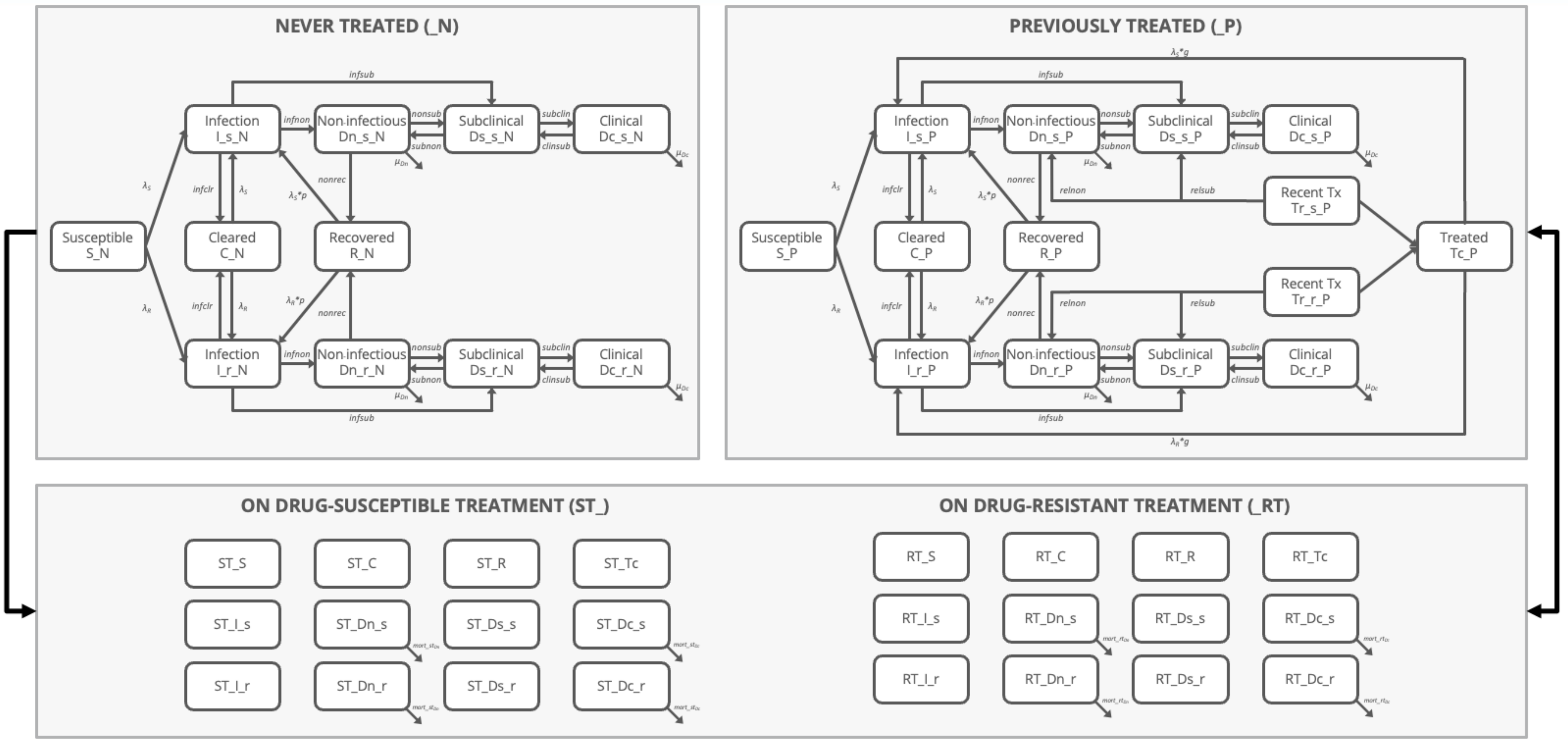

Figure S1. TB dimension structure. TB disease state and parameter naming follow conventions from research over the past decade; results are reported using current WHO terminology.

1.2.1 NATURAL HISTORY

This section aims to describe in detail the TB natural history structure, without considerations of treatment history or strain susceptibility.

Figure S2 shows 9 states that exemplify the essence of the TB natural history. Disease state classification was informed by the ICE-TB framework (Coussens et al., 2024) , and naming follows current WHO definitions (Falzon et al., 2024).

- Susceptible individuals (S) are infected at rate λ and progress to the infected compartment. Infected individuals (I) may clear infection (C) at rate *infclr* or progress to non-infectious disease (Dn) or asymptomatic disease (Ds) at rates *infnon* and *infsub*, respectively.
- Individuals with non-infectious disease (Dn) recover (R) at rate *nonrec* or progress to asymptomatic disease (Ds) at rate *nonsub* or die from TB-associated mortality at rate μ_{Dn}. Individuals with asymptomatic disease (Ds) regress to non-infectious disease (Dn) at rate *subnon* or progress to symptomatic disease (Dc) at rate *subclin*. Individuals with symptomatic disease (Dc) regress to asymptomatic disease (Ds) at rate *clinsub* or die from TB-associated mortality at rate μ_{Dc}.
- Diagnosis and treatment are described in detail in the following section. Recently treated individuals (Tr) may relapse to non-infectious disease (Dn) or asymptomatic disease (Ds) at rates *relnon* and *relsub*, respectively. Furthemore, recently treated individuals (Tr) progress to Treated (Tc). Individuals who have recovered from non-infectious disease (R) and those who have been treated for infectious disease (Tc) have a different risk of reinfection expressed through a relative risk factor *p* (protection after recovery) and *g* (greater risk after treatment)

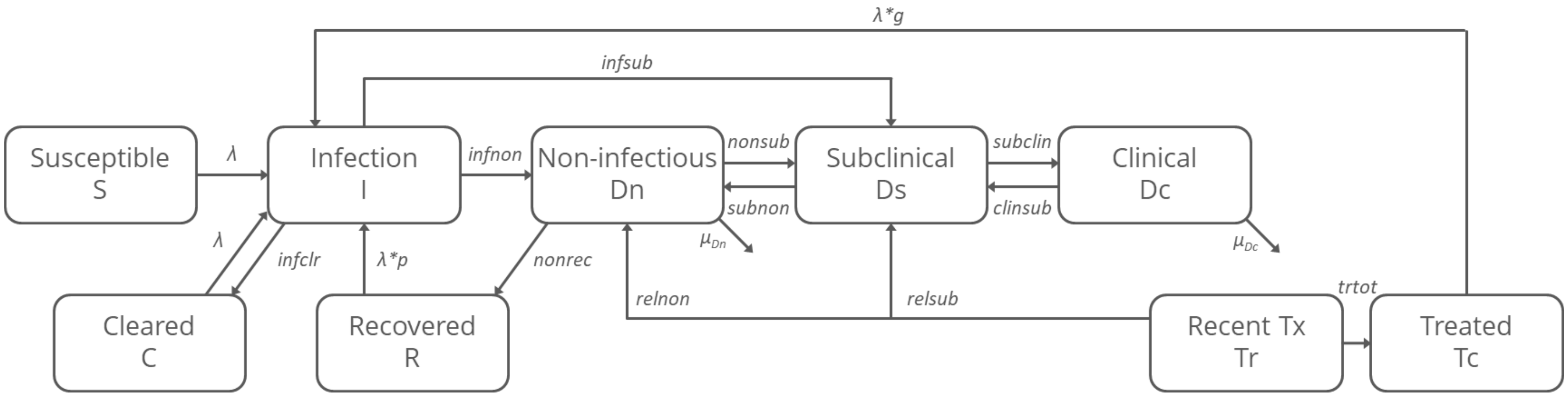

Figure S2. Natural history structure within TB dimension. TB disease state and parameter naming follow conventions from research over the past decade; results are reported using current WHO terminology.

<u>Force of infection (λ_{str})</u>

The formula calculates the force of infection (λ_{str}) for a given strain of *Mycobacterium tuberculosis* (*Mtb*) by considering the probability of transmission per contact (*pT*), a scaling factor for contacts (*cscal*), and the sum of age-specific contact rates (*C[m,y]*). Additionally, it incorporates the number of individuals with symptomatic (*TDc*) and asymptomatic TB (*TDs*) (accounting for the relative infectiousness *t* of asymptomatic disease), the relative infectiousness of the strain (*relinf*), the relative infectiousness of children compared to adults (*k*), and the total population in each age group (*Ny*).

Susceptible individuals (S) are infected at rate λ_S or λ_R. λ is defined as follows:

$$\lambda_{str} = pT * cscal * \sum_{y=1}^{n_{ygroups}} C[m,y] * \left(\frac{\left(TDc_{str_y} + tTDs_{str_y}\right) * relinf_{str} * k}{N_y} \right)$$

where
- *str* indicates strain (drug susceptible or drug resistant)
- *pT* indicates probability of transmission per contact
- *cscal* indicates a multiplier to scale contacts to fit to burden data
- *y* indicates the age group of contact
- $n_{ygroups}$ indicates the number of contact age groups
- *C[m,y]* indicates the number of age-specific contacts. It is the element of the contact matrix ***C*** indicating the number of yearly contacts between individuals of age groups m and y.
- TDc_{stry} indicates the total number of individuals with symptomatic TB of strain *str* in age group *y*
- *t* indicates the relative infectiousness of asymptomatic TB relative to symptomatic TB
- TDs_{stry} indicates the total number of individuals with asymptomatic TB of strain *str* in age group *y*
- $relinf_{str}$ indicates the relative infectiousness of the strain
- *k* indicates the relative infectiousness of children (<15 years) relative to adults (≥15 years)
- *Ny* indicates the total population size in age group *y*

1.2.2 DIAGNOSIS AND TREATMENT

This section aims to provide a detailed description of the transitions into and out of the treatment compartments. **Figure S3** shows the stratification of the TB natural history model described previously into two similar structures representing treatment history: never treated (_N) and previously treated (_P).

- Diagnosis and treatment are structured across two nearly parallel sub-strata, one for individuals never treated (_N) and one for individuals previously treated (_P). Note that Recently treated (Tr) and Treated (Tc) are only featured among those previously treated, as per definition.
- Individuals from any TB state (except those Recently treated (Tr)) may be placed on standard first-line treatment (ST_) or second-line treatment for drug-resistant TB (RT_).
- On-treatment states do not distinguish treatment history, i.e. individuals with asymptomatic TB (Ds) will be placed on either ST or RT treatment regardless of their treatment history.

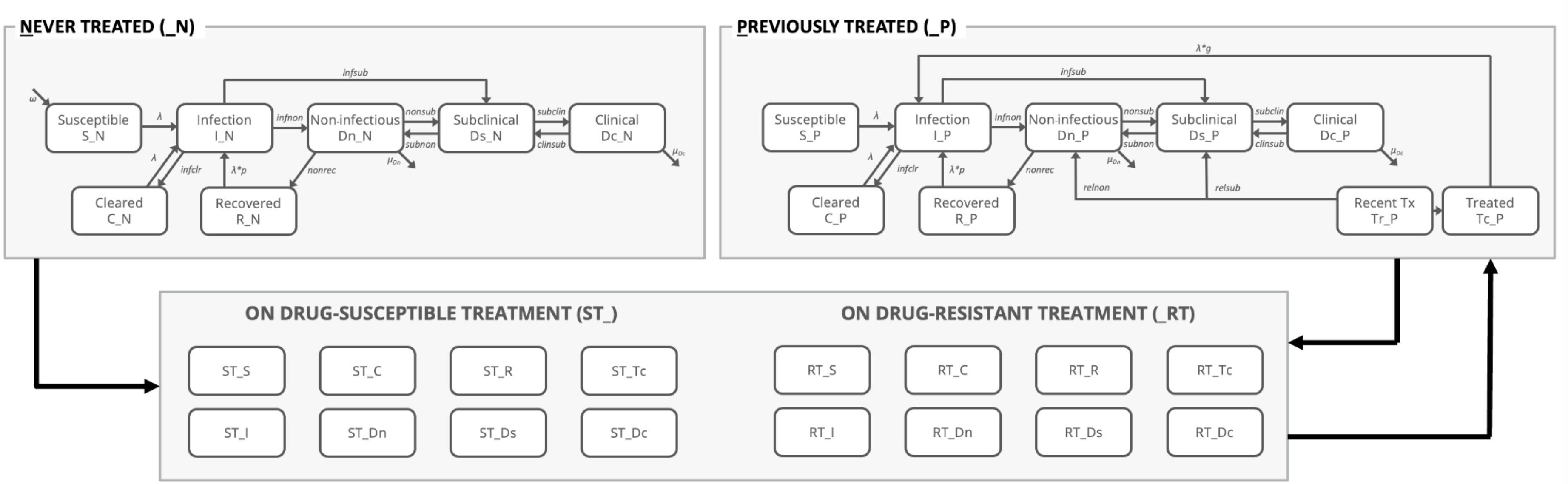

Figure S3. Diagnosis and treatment structure. TB disease state and parameter naming follow conventions from research over the past decade; results are reported using current WHO terminology.

Figure S4 provides further detail on the flows of treatment initiations for each state. Boxes in light grey and dashed borders are duplicates (i.e. not repeated in each treatment history state), but shown here to help representation of state flows.

- There are separate on-treatment compartments for each TB state. Individuals initiate treatment standard treatment (ST_), following blue dashed lines, or drug-resistant treatment (RT_), following yellow dashed lines. Treatment initiation is determined by a treatment initiation rate (*tinit*), a country-specific factor that controls the overall treatment initiation rate (*eta*), and a relative factor (*relinit*), which varies across TB states.
- Initiation of drug-resistant treatment is defined by access to drug susceptibility testing (DST), which differs based on treatment history (*dst_n, dst_p*) and probability of a positive DST result, which is determined by drug resistance status (*dst_s, dst_r*).
- Recently treated individuals (Tr_P) are not placed on treatment due to recent treatment history.

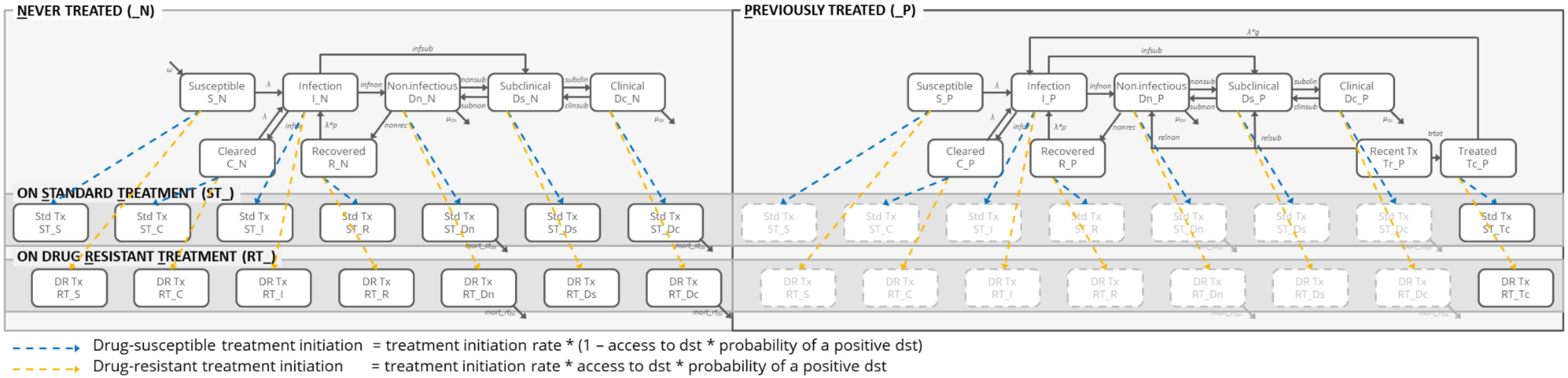

Figure S4. Treatment initiation in diagnosis and treatment structure. TB disease state and parameter naming follow conventions from research over the past decade; results are reported using current WHO terminology.

Figure S5 provides detail on the flows of treatment completion for each state. Boxes in light grey and dashed borders are duplicates (i.e. not repeated in each treatment history state), but shown here to help representation of state flows.

- Individuals who are placed on treatment from susceptible (S), cleared (C), recovered (R), or treated (Tc) states move to the same state in the previously treated sub-dimension (_P) according to the rate of treatment completion (inverse of treatment duration), which is specific to the treatment regimen (*comp_st, comp_rt*). These transitions are not shown in the figure below.
- Individuals complete treatment successfully (green solid lines), or unsuccessfully (orange dotted lines). Successful treatment completion is defined by the rate of treatment completion (inverse of treatment duration), which is specific to the treatment regimen (*comp_st, comp_rt*), and the probability of treatment success (succ_st, succ_rt), which acknowledges a relative treatment success for individuals with drug-resistant TB who are placed on standard treatment (succ_strs).
    - Infected individuals (I) move to the previously treated cleared state (C_P) if treatment is successful and the previously treated infection state (I_P) if treatment is unsuccessful.
    - Individuals with non-infectious disease (Dn) move to the previously treated recovered state (R_P) if treatment is successful and the previously treated non-infectious disease state (Dn_P) if treatment is unsuccessful.
    - Individuals with asymptomatic disease (Ds) or symptomatic disease (Dc) move to the previously treated recent treatment state (Tr_P) if treatment is successful and the previously treated asymptomatic disease state (Ds_P) or the previously treated symptomatic disease state (Dc_P) if treatment is unsuccessful.
- Individuals with non-infectious disease (Dn) or symptomatic disease (Dc) are also at risk of mortality whilst on treatment, which is specific to the treatment regimen (*mort_st, mort_rt*).

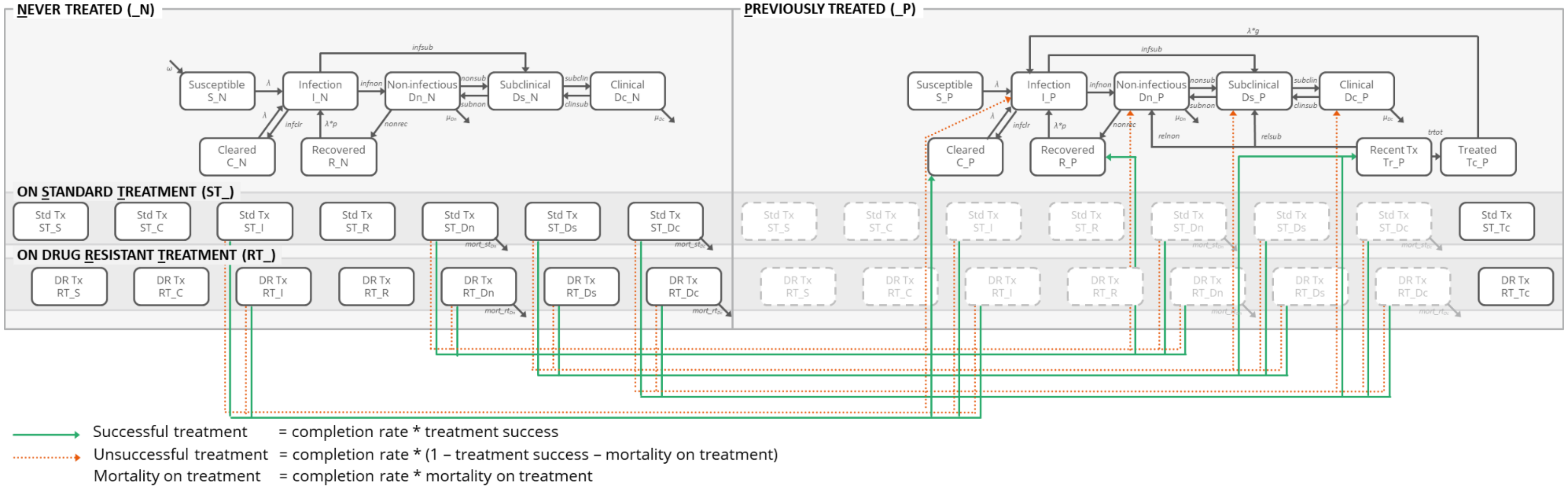

Figure S5. Treatment completion in diagnosis and treatment structure. TB disease state and parameter naming follow conventions from research over the past decade; results are reported using current WHO terminology.

1.2.3 DRUG RESISTANCE

This section aims to describe in detail the stratification into strain susceptibility.

Figure S6 adds further stratification of the TB natural history by introducing strain susceptibility on infection and disease states. The figure shows duplicate states (shown in light grey with dashed outlines) which are placed for convenient representation of structure.

- Drug resistance status is modelled using two nearly parallel sub-strata, one for individuals with drug-susceptible strains (_s) and one for individuals with drug-resistant (i.e., rifampicin-resistant) strains (_r).
- Infected (I), recently treated (Tr) and all three disease states (Dn, Ds, Dc) are subdivided based on strain susceptibility.
- Both transmission of drug-resistant strains and acquisition of drug resistance during treatment are modelled.
- Relapse rates are disaggregated by drug-resistance strain (*relnon_s*, *relsub_s*, *relnon_r*, *relsub_r*)

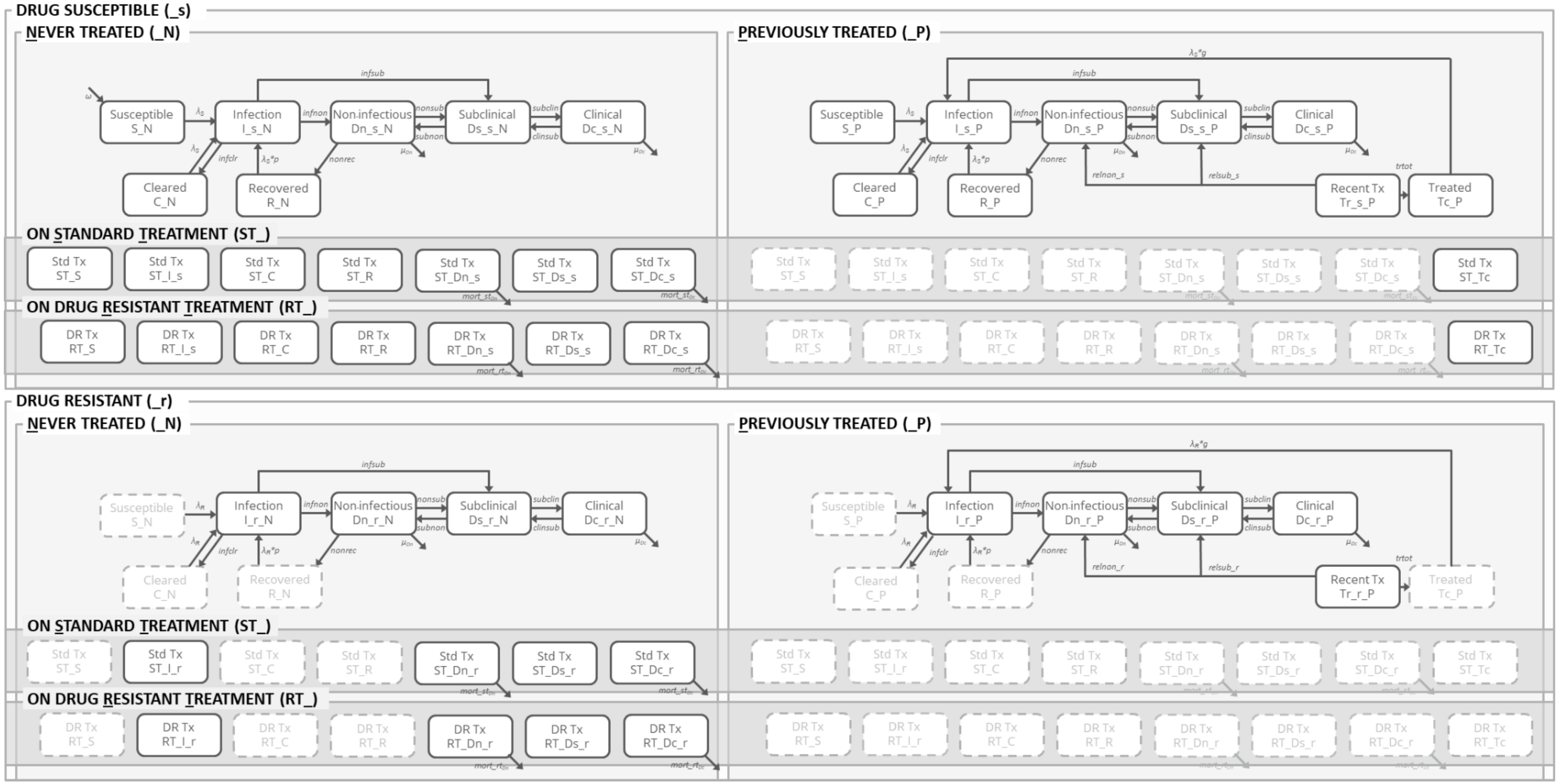

Figure S6. Drug resistance structure. TB disease state and parameter naming follow conventions from research over the past decade; results are reported using current WHO terminology.

Figure S7 shows the transmission of drug susceptible and drug resistant strains. The figure shows duplicate states (shown in light grey with dashed outlines) which are placed for convenient representation of structure.

- Individuals in susceptible (S), cleared (C), recovered (R), and treatment (Tc) compartments can be infected with drug-susceptible or drug-resistant strains.

- Individuals with drug-susceptible infections (I_s) can be superinfected with drug-resistant strains (solid orange lines); however, it is assumed that there is no superinfection with drug-resistant strains among individuals with established drug-susceptible disease (Dn_s, Ds_s, Dc_s). Superinfection with drug-susceptible strains among individuals with drug-resistant infection (I_r) or disease (Dn_r, Ds_r, Dc_r) is not modelled.
- Transmission of drug-resistant strains acknowledges a fitness cost due to the genetic mutations that confer resistance (see Section 1.2.1 above).
- A fixed proportion of individuals with drug-susceptible infections (I_s) and with drug-susceptible disease (Dn_s, Ds_s, Dc_s) acquire resistance whilst on standard treatment at rate *acqres* (shown in dashed orange lines).

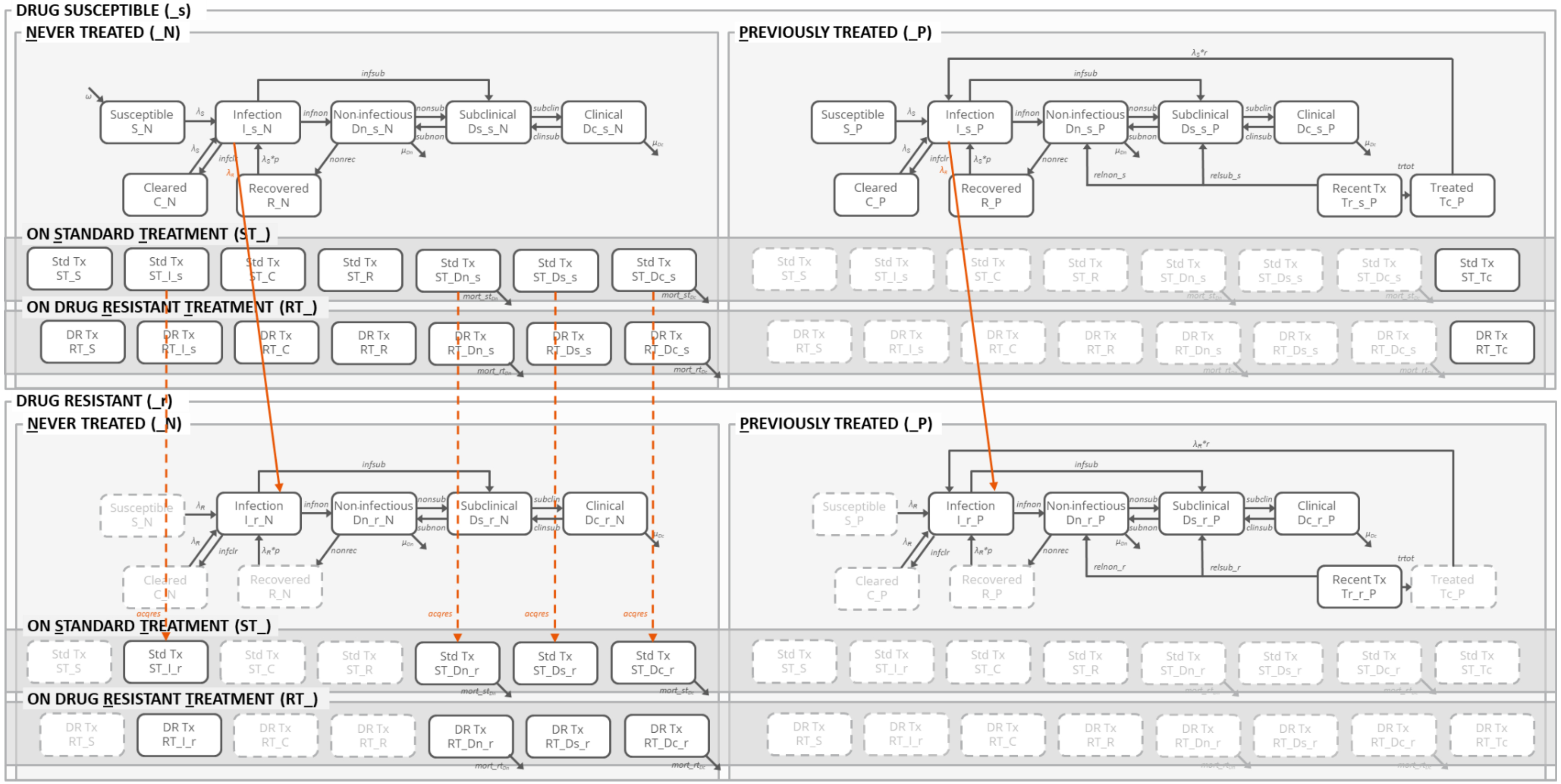

Figure S7. Drug resistance transmission and acquisition in drug resistance structure. TB disease state and parameter naming follow conventions from research over the past decade; results are reported using current WHO terminology.

1.3 PARAMETERS

The following section will look into the parameters in detail, describing their use, their values or ranges and whether or not they are age or time-dependent.

Table S1. TB dimension parameters.

Parameter	Description (units)	Prior/Value	Age-dependent	Time-dependent	Reference
pT	Probability of transmission per contact per day	0.0013<br>See note 1	No	No	Estimated from data
cscal	Multiplier to scale contacts to fit to burden data	ZAF/IND: 0-4<br>BRA: 0-2.5	No	No	Assumption
relfit	Relative fitness of drug-resistant strains relative to drug-susceptible strains	0.4-0.9	No	No	(Kendall et al., 2017)
k	Relative infectiousness of children compared to adults	0.25-1.00	Yes	No	Assumption
t	Relative infectiousness of asymptomatic compared to symptomatic	0.62-1.00	No	No	(Emery et al., 2023)<br>Range truncated at 1.00
p	Relative risk of infection following recovery from non-infectious	0.14-0.30	No	No	(Andrews et al., 2012)
g	Relative risk of infection following treatment completion	2.14-4.27	No	No	(Glynn et al., 2010; Verver et al., 2005)
infclr	Clearance from infection per year	0.93-3.30	No	No	(Horton et al., 2023)
infnon	Progression from infection to non-infectious per year	0.04-0.23	No	No	(Horton et al., 2023)
infsub	Progression from infection to asymptomatic per year	0.01-0.10	No	No	(Horton et al., 2023)
nonrec	Recovery from non-infectious per year	0.14-0.23	No	No	(Horton et al., 2023)
nonsub	Progression from non-infectious to asymptomatic per year	0.21-0.28	Yes	No	(Horton et al., 2023)
subnon	Regression from asymptomatic to non-infectious per year	1.24-2.03	No	No	(Horton et al., 2023)
subclin	Progression from asymptomatic to symptomatic per year	0.56-0.94	Yes	No	(Horton et al., 2023)
clinsub	Regression from symptomatic to asymptomatic per year	0.46-0.72	No	No	(Horton et al., 2023)
μ_{Dn}	Non-infectious TB mortality per year	0.00-0.45<br>See note 2	Yes	No	Assumption
μ_{Dc}	Symptomatic TB mortality per year	0.017-0.45	No	No	(Ragonnet et al., 2021)
tinit	Sigmoidal curve describing rate of treatment initiation	See note 3	Yes	Yes	Assumption
midx	Parameter to define *tinit*	See note 3	No	No	Assumption

sh	Parameter to define *tinit*	See note 3	No	No	Assumption
eta	Country-specific access factor	ZAF/BRA: 0-1<br>IND: 0-1.5<br>See note 3	No	No	Assumption
relinit	Relative access to TB screening by core TB state	See note 3	No	No	See note 3
dst_n	Access to drug-susceptibility testing in never treated individuals	See note 4	No	Yes	(World Health Organization, 2023)<br>WHO case notifications<br>WHO drug resistance testing
dst_p	Access to drug-susceptibility testing in previously treated individuals	See note 4	No	Yes	(World Health Organization, 2023)<br>WHO case notifications<br>WHO drug resistance testing
dst_s	Probability of a positive drug-susceptibility test result for uninfected individuals and individuals with drug susceptible TB	0.01<br>See note 4	No	No	(Zifodya et al., 2021)
dst_r	Probability of a positive drug-susceptibility test result for individuals with drug resistant TB	0.95<br>See note 4	No	No	(Zifodya et al., 2021)
comp_st	Treatment completion rate for standard treatment per year (1/duration of treatment)	2.0	No	Yes	(World Health Organization, 2022b)
comp_rt	Treatment completion rate for drug resistant treatment per year (1/duration of treatment)	ZAF/IND: 1.3-2.0<br>BRA: 0.67-2.0	No	Yes	(World Health Organization, 2022a)
succ_st	Probability of treatment success for standard treatment of drug susceptible TB	See note 5	No	Yes	(World Health Organization, 2023)<br>WHO treatment outcomes
succ_rt	Probability of treatment success for drug resistant treatment of drug resistant TB	See note 5	No	Yes	(World Health Organization, 2023)<br>WHO treatment outcomes
mort_st	Probability of death on drug susceptible treatment	See note 5	No	Yes	(World Health Organization, 2023)<br>WHO treatment outcomes
mort_rt	Probability of death on drug resistant treatment	See note 5	No	Yes	(World Health Organization, 2023)<br>WHO treatment outcomes
succ_strs	Relative treatment success of standard treatment for drug resistant TB	0.35-0.70	No	No	(Espinal et al., 2000)<br>Range used is lower limit of previously treated and upper

					limit of never treated
relnon_s	Relapse from recently treated to non-infectious after standard treatment	0.00-0.01	No	No	Assumption
relsub_s	Relapse from recently treated to asymptomatic after standard treatment	0.00-0.01	No	No	Assumption
relnon_r	Relapse from recently treated to non-infectious after standard treatment	0.01-0.06	No	No	(Kendall et al., 2017)
relsub_r	Relapse from recently treated to asymptomatic after standard treatment	0.01-0.06	No	No	(Kendall et al., 2017)
trtot	Transition from recently treated to treated per year	1<br>See note 6	No	No	Assumption
acqres	Rate of acquisition of rifampicin resistance during standard treatment per year	0.003-0.012	No	No	(Kendall et al., 2017)

Note 1: Probability of transmission per contact (pT)

The daily probability of transmission per respiratory contact between an infectious and healthy individual has a fixed value of 0.0013 (0.0002-0.0048). This value was estimated from the daily mean number of infectious contacts ((Prem et al., 2021; World Health Organization, 2023)) multiplied by the reversion-adjusted annual risk of infection from TST immunoreactivity surveys (Cauthen et al., 2002; Houben & Dodd, 2016).

$$pT = (mC[m,y] * TBprev\ * (ARI\ *\ rev))/365.25$$

where *mC[m,y]* indicates the mean number of age-specific contacts per year
TBprev indicates the country specific TB prevalence (%) from WHO
ARI indicates annual risk of infection from TST immunoreactivity surveys
rev indicates reversion-underestimation factor of ARI (Schwalb et al., 2023)

The parameter *cscal* is a calibration variable that acts upon *pT* and it ranges from 0 to 4 for IND and ZAF, and 0 to 2.5 for BRA.

Note 2: Non-infectious disease mortality per year

Non-infectious disease mortality is included in recognition that non-infectious disease includes both asymptomatic and symptomatic

disease (as per ICE-TB framework, (Coussens et al., 2024)) and is particularly severe amongst children. The prior parameter range is defined from 0 to the upper bound of the mortality rate for symptomatic infectious disease (Ragonnet et al., 2021).

Note 3: Treatment initiation rate

Treatment initiation rate (*init*) is defined as follows:

$$init = tinit * eta * relinit$$

where *tinit* indicates a sigmoid curve symbolising the scale-up of treatment access

eta indicates a country-specific calibration factor (between 0 and 1)

relinit indicates a TB state-specific factor

Tuberculosis treatment was assumed to start in 1960, aligned roughly with the discovery and widespread use of rifampicin, and increase following a sigmoid curve (**Figure S8**).

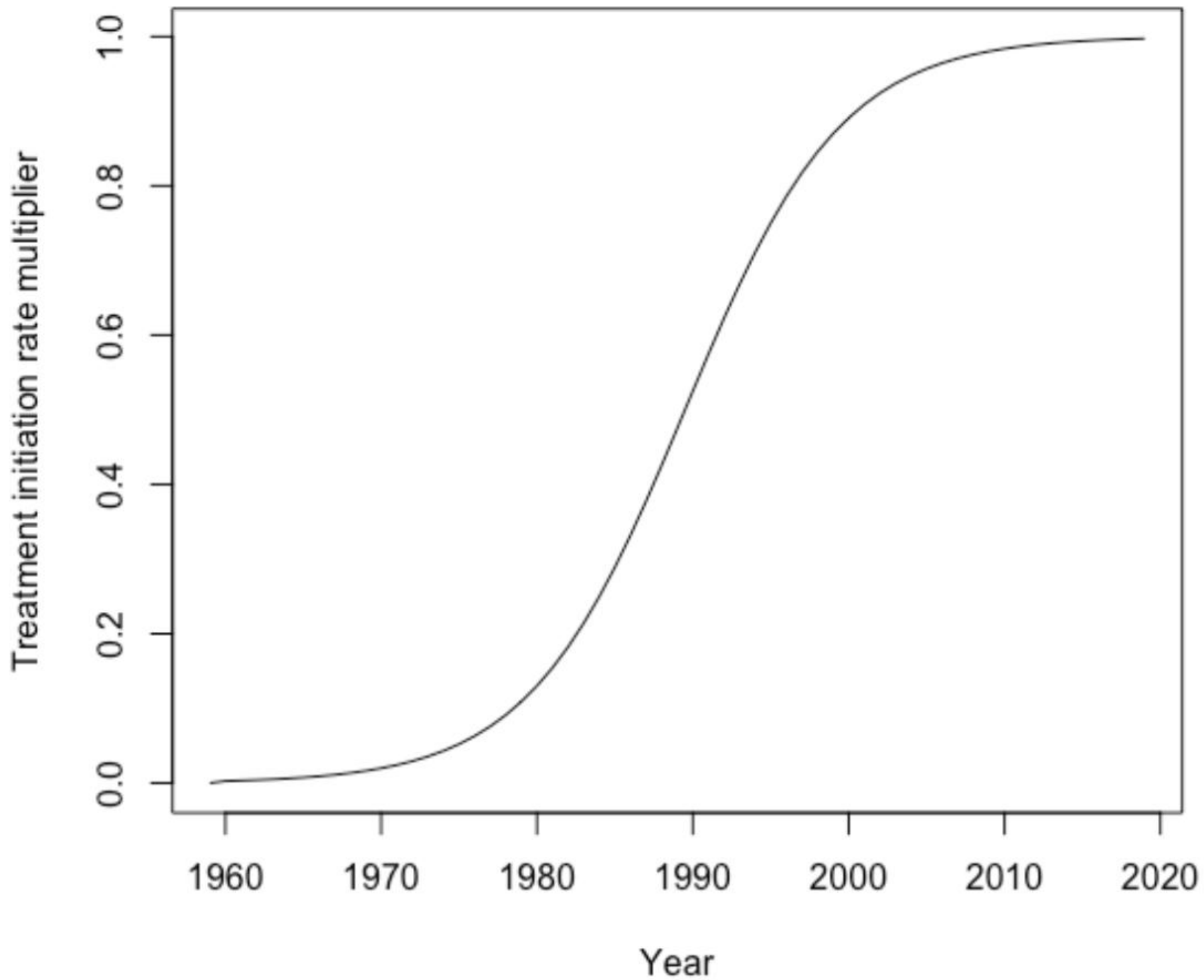

Figure S8. Sigmoid curve representing the scale-up in tuberculosis treatment.

The sigmoid curve *tint* is defined as follows:

$$tinit = \frac{1}{1 + e^{(-sh*(x-midx))}}$$

where *sh* indicates the steepness of the curve
midx indicates the midpoint of the curve

These parameters are fixed values defined by each country team.

Table S2. Country-specific sigmoid curve parameters

Parameter	South Africa	India	Brazil
sh	0.2	0.33	0.1
midx	2003.4	1970	2000

The TB state-specific factor (*relinit*) is used to recognise variability in treatment initiation.To enable adjustment of screening, diagnosis and treatment initiation between TB states each of these is specified separately, informed pre-baseline by:

- Relative access to diagnosis pathway (*access)*: Values are based on screening positivity of prolonged cough, using Dc as reference. We assume a relative access increase of 50% for those previously treated (_P).
- Probability of a positive test (*testpos*): Values are based on country-specific proportions of bacteriologically confirmed and clinically diagnosed notifications, according to WHO datasets, combined with test positivity of Xpert MTB/RIF, smear or culture, and clinical diagnosis.
- Proportion who accept treatment (*accept)*: Values are assumptions, using Dc as reference.

During interventions, values are adjusted to match intervention scale-up.

Table S3. Baseline relative treatment initiation by state

Relinit is equal to access*testpos*accept

State	Relative access to diagnostic pathway (access)	Probability of a positive test (testpos) ZAF	IND	BRA	Proportion who accept treatment (accept)
S_N	0.06	0.0251	0.0294	0.0208	0.50
C_N	0.06	0.0251	0.0294	0.0208	0.50
R_N	0.06	0.067	0.0821	0.0635	0.50
I_s_N	0.06	0.0251	0.0294	0.0208	0.50
I_r_N	0.06	0.0251	0.0294	0.0208	0.50
Dn_s_N	0.19	0.0882	0.1307	0.0909	0.75
Dn_r_N	0.19	0.0882	0.1307	0.0909	0.75
Ds_s_N	0.06	0.4481	0.3549	0.4420	0.50
Ds_r_N	0.06	0.4481	0.3549	0.4420	0.50

Dc_s_N	1.00	0.7922	0.7599	0.7901	1.00
Dc_r_N	1.00	0.7922	0.7599	0.7901	1.00
S_P	0.09	0.0251	0.0294	0.0208	0.50
C_P	0.09	0.0251	0.0294	0.0208	0.50
R_P	0.09	0.067	0.0821	0.0635	0.50
I_s_P	0.09	0.0251	0.0294	0.0208	0.50
I_r_P	0.09	0.0251	0.0294	0.0208	0.50
Dn_s_P	0.29	0.0882	0.1307	0.0909	0.75
Dn_r_P	0.29	0.0882	0.1307	0.0909	0.75
Ds_s_P	0.09	0.4481	0.3549	0.4420	0.50
Ds_r_P	0.09	0.4481	0.3549	0.4420	0.50
Dc_s_P	1.50	0.7922	0.7599	0.7901	1.00
Dc_r_P	1.50	0.7922	0.7599	0.7901	1.00
Tc_P	0.09	0.2366	0.2778	0.1983	0.75

Note 4: Drug susceptibility testing (DST)

DST access is parameterised using data from the WHO Global Tuberculosis Programme databases as reported in the diagnosis, notification and treatment of rifampicin-resistant TB dashboard (World Health Organization, 2023). The proportion of individuals tested for rifampicin-resistant resistant is multiplied by the proportion of notifications that are bacteriologically-confirmed.

Based on the sensitivity and specificity of Xpert Mtb/RIF and Xpert Ultra for the detection of rifampicin resistance (Zifodya et al., 2021), we assume that 1% of individuals who do not have drug resistant infection or disease and 95% of individuals who do have drug resistant infection or disease will receive a positive result on drug susceptibility testing.

Table S4. Country-specific DST access by treatment history.

State	South Africa	India	Brazil
	Value	Value	Value
dst_n	0.621	0.4	0.318

dst_p	0.618	0.67	0.341

Note 5: Treatment success and mortality

This section will describe the parameters used to account for treatment success and mortality.

Treatment success is only relevant to individuals with infection, non-infectious TB, asymptomatic TB, or symptomatic TB, and is parameterised using data from the WHO Global Tuberculosis Programme database on treatment outcomes (World Health Organization, 2023). Transitions following treatment for susceptible, cleared, recovered, and treated individuals are not distinguished by treatment success, and recently treated individuals are not placed on treatment.Treatment success is expected to be higher among infected individuals and individuals with non-infectious TB than among those with bacteriologically positive asymptomatic or symptomatic TB. Only individuals with non-infectious TB and symptomatic TB are at risk of mortality on treatment; mortality is considered as unsuccessful treatment.

Table S5. Definitions of treatment success and mortality by disease state

State	Treatment success	Mortality on treatment	Unsuccessful treatment
Infected	1	0	0
Non-infectious TB [Children]	(1 - mortality on treatment) * Treatment success[1] (defined below)	Mortality on treatment (defined below)	(1 - mortality on treatment) * (1- (treatment success)
Non-infectious TB [Adults]	Midpoint between adjusted treatment success[1] (defined below) and 1	0	(1- treatment success)
Asymptomatic TB	Treatment success (defined below)	0	(1- treatment success)
Symptomatic TB	(1 - mortality on treatment) * Treatment success[1] (defined below)	Mortality on treatment (defined below)	(1 - mortality on treatment) * (1- (treatment success)

[1] Treatment success of non-infectious and symptomatic TB was adjusted to account for mortality on treatment.

For drug susceptible TB, treatment success is defined as the total number of treatment success outcomes (cured or treatment completed) among new, relapse, and previously treated individuals, divided by the total cohort size of new, relapse, and previously treated individuals:

$$\textit{Treatment success} = (newrel\_succ + ret\_nrel\_succ) / (newrel\_coh + ret\_nrel\_coh)$$

Mortality on treatment is defined as the number of deaths on treatment, plus half the number of individuals who are reported as lost to follow-up, among new, relapse, and previously treated individuals, divided by the total cohort size of new, relapse, and previously treated individuals:

*Mortality on treatment = (newrel_died + 0.5*newrel_lost + ret_nrel_died + 0.5*ret_nrel_lost) / (newrel_coh + ret_nrel_coh)*

For drug resistant TB, treatment success is defined as the total number of treatment success outcomes (cured or treatment completed) among individuals with MDR-TB, divided by the total cohort size of individuals with MDR-TB:

Proportion success treatment = (mdr_succ) / (mdr_coh)

Mortality on treatment is defined as the number of deaths on treatment, plus half the number of individuals who are reported as lost to follow-up, among individuals with MDR-TB, divided by the total cohort size of individuals with MDR-TB:

Proportion deaths on treatment = (mdr_died + 0.5*mdr_lost) / (mdr_coh)

Unsuccessful treatment is defined as the outcome for remaining individuals with MDR-TB:

Proportion unsuccessful treatment = 1 - (proportion successful treatment + proportion deaths on treatment)

Table S6. Country-specific treatment success and mortality

State	South Africa	India	Brazil
	Value	Value	Value
succ_stDn / succ_stDc	0.875	0.8617021	0.7882353
succ_stDs	0.77	0.81	0.67
mort_stDn / mort_stDc	0.12	0.062	0.15
succ_rtDn / succ_rtDn	0.7222222	0.7027027	0.6875
succ_rtDs	0.52	0.52	0.55
mort_rtDn / mort_rtDc	0.28	0.26	0.20

The value used for treatment success is different between non-infectious and symptomatic TB compared to asymptomatic TB to account for mortality on treatment.

Note 6: Relapse risk

We assume that individuals are at risk of relapse for a period of one year following treatment (Crampin et al., 2010; Marx et al., 2014).

1.4 DIFFERENTIAL EQUATIONS

This section aims to provide the differential equations which operate the model structure described above. Each differential equation describes flows and transitions experienced in each 49 states of TBMod.

1.4.1 EXPRESSIONS

lambda_all = pT*cscal*k*(t*(Ds_s_N + Ds_s_P) + Dc_s_N + Dc_s_P + relfit*(t*(Ds_r_N + Ds_r_P) + Dc_r_N + Dc_r_P)) / N

lambda_s = pT*cscal*k*(t*(Ds_s_N + Ds_s_P) + Dc_s_N + Dc_s_P) / N

lambda_r = pT*cscal*k*relfit*(t*(Ds_r_N + Ds_r_P) + Dc_r_N + Dc_r_P) / N

treat_init = tinit*eta*(access*testpos*accept)*dx_age

1.4.2 NEVER TREATED

dS_N/dt = -(treat_init + lamda_all)*S_N

dC_N/dt = infclr*(I_s_N + I_r_N) - (treat_init + lambda_all)*C_N

dR_N/dt = nonrec*(1/prog_age)*(Dn_s_N + Dn_r_N) - (treat_init + lambda_all*p)*R_N

dI_s_N/dt = lambda_s*(S_N + C_N + p*R_N) - (infclr + prog_age*(infnon + infsub))*I_s_N - (treat_init + lambda_r)*I_s_N

dI_r_N/dt = lambda_r*(S_N + C_N + p*R_N + I_s_N) - (infclr + prog_age*(infnon + infsub) + treat_init)*I_r_N

dDn_s_N/dt = infnon*prog_age*I_s_N + subnon*(1/prog_age)*Ds_s_N - (nonrec*(1/prog_age) + nonsub*prog_age + muDn*mort_age + treat_init)*Dn_s_N

dDn_r_N/dt = infnon*prog_age*I_r_N + subnon*(1/prog_age)*Ds_r_N - (nonrec*(1/prog_age) + nonsub*prog_age + muDn*mort_age + treat_init)*Dn_r_N

dDs_s_N/dt = infsub*prog_age*I_s_N + nonsub*prog_age*Dn_s_N + clinsub*(1/prog_age)*Dc_s_N - (subnon*(1/prog_age) + subclin*prog_age + treat_init)*Ds_s_N

dDs_r_N/dt = infsub*prog_age*I_r_N + nonsub*prog_age*Dn_r_N + clinsub*(1/prog_age)*Dc_r_N - (subnon*(1/prog_age) + subclin*prog_age + treat_init)* Ds_r_N

dDc_s_N/dt = subclin*prog_age*Ds_s_N - (clinsub*(1/prog_age) + muDc*mort_age + treat_init)*Dc_s_N

dDc_r_N/dt = subclin*prog_age*Ds_r_N - (clinsub*(1/prog_age) + muDc*mort_age + treat_init)*Dc_r_N

1.4.3 PREVIOUSLY TREATED

dS_P/dt = comp_st*ST_S + comp_rt*RT_S - (treat_init + lambda_all)*S_P

dC_P/dt = infclr*(I_s_P + I_r_P) + comp_st*(ST_C + succ_stI*(ST_I_s + succ_strs*ST_I_r)) + comp_rt*(RT_C + succ_rtI*(RT_I_s + RT_I_r)) - (treat_init + lambda_all)*C_P

dR_P/dt = nonrec*(1/prog_age)*(Dn_s_P + Dn_r_P) + comp_st*(ST_R + succ_stDn*(ST_Dn_s + succ_strs*ST_Dn_r)) + comp_rt*(RT_R + succ_rtDn*(RT_Dn_s + RT_Dn_r)) - (treat_init + lambda_all*p)*R_P

dI_s_P/dt = comp_st*(1-succ_stI)*ST_I_s + comp_rt*(1-succ_rtI)*RT_I_s + lambda_s*(S_P + C_P + p*R_P + r*Tc_P) - (infclr + prog_age*(infnon + infsub*) + treat_init + lambda_r)*I_s_P

dI_r_P/dt = comp_st*(1-succ_stI*succ_strs)*ST_I_r + comp_rt*(1-succ_rtI)*RT_I_r + lambda_r*(S_P + C_P + p*R_P + I_s_P + r*Tc_P) - (infclr + prog_age*(infnon + infsub) + treat_init)*I_r_P

dDn_s_P/dt = infnon*prog_age*I_s_P + subnon*(1/prog_age)*Ds_s_P + comp_st*(((1-mort_stDn)*mort_age*(1-succ_stDn))+((1-mort_age)*(1-((1+succ_stDn)/2))))*ST_Dn_s + comp_rt*(((1-mort_rtDn)*mort_age*(1-succ_rtDn))+((1-mort_age)*(1-

((1+succ_rtDn)/2)))*RT_Dn_s + relnon_s*Tr_s_P - (nonrec*(1/prog_age) + nonsub*prog_age + muDn*mort_age + treat_init)*Dn_s_P

dDn_r_P/dt = infnon*prog_age*I_r_P + subnon*(1/prog_age)*Ds_r_P + comp_st*(((1-mort_stDn)*mort_age*((1-succ_stDn)*succ_strs))+((1-mort_age)*(1-(((1+succ_stDn)/2)*succ_strs)))*ST_Dn_r + comp_rt*(((1-mort_rtDn)*mort_age*(1-succ_rtDn))+((1-mort_age)*(1-((1+succ_rtDn)/2)))*RT_Dn_r + relnon_r*Tr_r_P - (nonrec*(1/prog_age) + nonsub*prog_age + muDn*mort_age + treat_init)*Dn_r_P

dDs_s_P/dt = prog_age*(infsub*I_s_P + nonsub*Dn_s_P) + clinsub*(1/prog_age)*Dc_s_P + comp_st*(1-succ_stDs)*ST_Ds_s + comp_rt*(1-succ_rtDs)*RT_Ds_s + relsub_s*Tr_s_P - (subnon*(1/prog_age) + subclin*prog_age + treat_init)*Ds_s_P

dDs_r_P/dt = prog_age*(infsub*I_r_P + nonsub*Dn_r_P) + clinsub*(1/prog_age)*Dc_r_P + comp_st*(1-succ_stDs*succ_strs)*ST_Ds_r + comp_rt*(1-succ_rtDs)*RT_Ds_r + relsub_r*Tr_r_P - (subnon*(1/prog_age) + subclin*prog_age + treat_init)*Ds_r_P

dDc_s_P/dt = subclin*prog_age*Ds_s_P + comp_st*(1-(mort_stDc)*(1-succ_stDc)*ST_Dc_s + comp_rt*(1-(mort_rtDc)*(1-succ_rtDc)*RT_Dc_s - (clinsub*(1/prog_age) + muDc*mort_age + treat_init)*Dc_s_P

dDc_r_P/dt = subclin*prog_age*Ds_r_P + comp_st*(1-(mort_stDc)*(1-succ_stDc*succ_strs)*ST_Dc_r + comp_rt*(1-(mort_rtDc)*(1-succ_rtDc)*RT_Dc_r - (clinsub*(1/prog_age) + muDc*mort_age + treat_init)*Dc_r_P

dTr_s_P/dt = comp_st*(succ_stDs*ST_Ds_s + succ_stDc*ST_Dc_s) + comp_rt*(succ_rtDs*RT_Ds_s + succ_rtDc*RT_Dc_s) - (trtot + relnon_s + relsub_s)*Tr_s_P

dTr_r_P/dt = comp_st*succ_strs*(succ_stDs*ST_Ds_r + succ_stDc*ST_Dc_r) + comp_rt*(succ_rtDs*RT_Ds_r + succ_rtDc*RT_Dc_r) - (trtot + relnon_r + relsub_r)*Tr_r_P

dTc_P/dt = comp_st*ST_Tc + comp_rt*RT_Tc + trtot*(Tr_s_P + Tr_r_P) - (treat_init + lambda_all*g)*Tc_P

1.4.4 ON TREATMENT

dST_S/dt = treat_init*((1-dst_n*dst_s)*S_N + (1-dst_p*dst_s)*S_P) - comp_st*ST_S

dRT_S/dt = treat_init*dst_s*(dst_n*S_N + dst_p*S_P) - comp_rt*RT_S

dST_C/dt = treat_init*((1-dst_n*dst_s)*C_N + (1-dst_p*dst_s)*C_P) - comp_st*ST_C

dRT_C/dt = treat_init*dst_s*(dst_n*C_N + dst_p*C_P) - comp_rt*RT_C

dST_R/dt = treat_init((1-dst_n*dst_s)*R_N + (1-dst_p*dst_s)*R_P) - comp_st*ST_R

dRT_R/dt = treat_init*dst_s*(dst_n*R_N + dst_p*R_P) - comp_rt*RT_R

dST_I_s/dt = treat_init*((1-dst_n*dst_s)*I_s_N + (1-dst_p*dst_s)*I_s_P) - (comp_st + acqres)*ST_I_s

dRT_I_s/dt = treat_init*dst_s*(dst_n*I_s_N + dst_p*I_s_P) - comp_rt*RT_I_s

dST_I_r/dt = acqres*ST_I_s + treat_init*((1-dst_n*dst_r)*I_r_N + (1-dst_p*dst_r)*I_r_P) - comp_st*ST_I_r

dRT_I_r/dt = treat_init*dst_r*(dst_n*I_r_N + dst_p*I_r_P) - comp_rt*RT_I_r

dST_Dn_s/dt = treat_init*((1-dst_n*dst_s)*Dn_s_N + (1-dst_p*dst_s)*Dn_s_P) - (comp_st + acqres)*ST_Dn_s

dRT_Dn_s/dt = treat_init*dst_s*(dst_n*Dn_s_N + dst_p*Dn_s_P) - comp_rt*RT_Dn_s

dST_Dn_r/dt = acqres*ST_Dn_s + treat_init*((1-dst_n*dst_r)*Dn_r_N + (1-dst_p*dst_r)*Dn_r_P) - comp_st*ST_Dn_r

dRT_Dn_r/dt = treat_init*dst_r*(dst_n*Dn_r_N + dst_p*Dn_r_P) - comp_rt*RT_Dn_r

dST_Ds_s/dt = treat_init*((1-dst_n*dst_s)*Ds_s_N + (1-dst_p*dst_s)*Ds_s_p) - (comp_st + acqres)*ST_Ds_s

dRT_Ds_s/dt = treat_init*dst_s*(dst_n*Ds_s_N + dst_p*Ds_s_P) - comp_rt*RT_Ds_s

dST_Ds_r/dt = acqres*ST_Ds_s + treat_init*((1-dst_n*dst_r)*Ds_r_N + (1-dst_p*dst_r)*Ds_r_P) - comp_st*ST_Ds_r

dRT_Ds_r/dt = treat_init*dst_r*(dst_n*Ds_r_N + dst_p*Ds_r_P) - comp_rt*RT_Ds_r

dST_Dc_s/dt = treat_init*((1-dst_n*dst_s)*Dc_s_N + (1-dst_p*dst_s)*Dc_s_P) - (comp_st + acqres)*ST_Dc_s

dRT_Dc_s/dt = treat_init*dst_s*(dst_n*Dc_s_N + dst_p*Dc_s_P) - comp_rt*RT_Dc_s

dST_Dc_r/dt = acqres*ST_Dc_s + treat_init*((1-dst_n*dst_r)*Dc_r_N + (1-dst_p*dst_r)*Dc_r_P) - comp_st*ST_Dc_r

dRT_Dc_r/dt = treat_init*dst_r*(dst_n*Dc_r_N + dst_p*Dc_r_P) - comp_rt*RT_Dc_r

dST_Tc/dt = treat_init*(1-dst_p*dst_s)*Tc_P - comp_st*ST_Tc

dRT_Tc/dt = treat_init*dst_p*dst_s*Tc_P - comp_rt*RT_Tc

2 AGE DIMENSION

2.1 OVERVIEW

- The TB dimension described in detail above is further divided into sixteen 5-year age groups.
- TBMod operates in the background with country and age-specific demographics informed by the UN World Population Prospects.

2.2 STRUCTURE

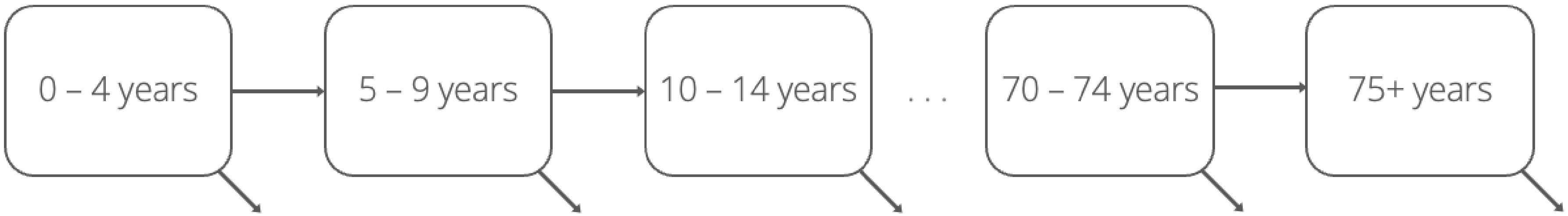

Figure S9. Age dimension structure.

2.3 INTERACTION BETWEEN AGE AND TB DIMENSION

This section will describe how the interaction between age and TB is modelled. We assume that there are differing age-specific parameters in children (<15 years old, i.e. first three age groups) and in adults (≥15 years old, i.e. all other age groups). Particularly, children-specific parameters are either higher or lower when compared to their adult counterpart.

Figure S10 shows where the age-specific adjustment factors are applied in the TB natural history structure. Parameters follow a naming convention where first it specifies where the parameter is acting upon (i.e., *prog* in progression flows) and then which dimension it refers to, in this case *age*. An additional age-specific adjustment (*dx_age*) is not shown but it acts upon treatment initiation.

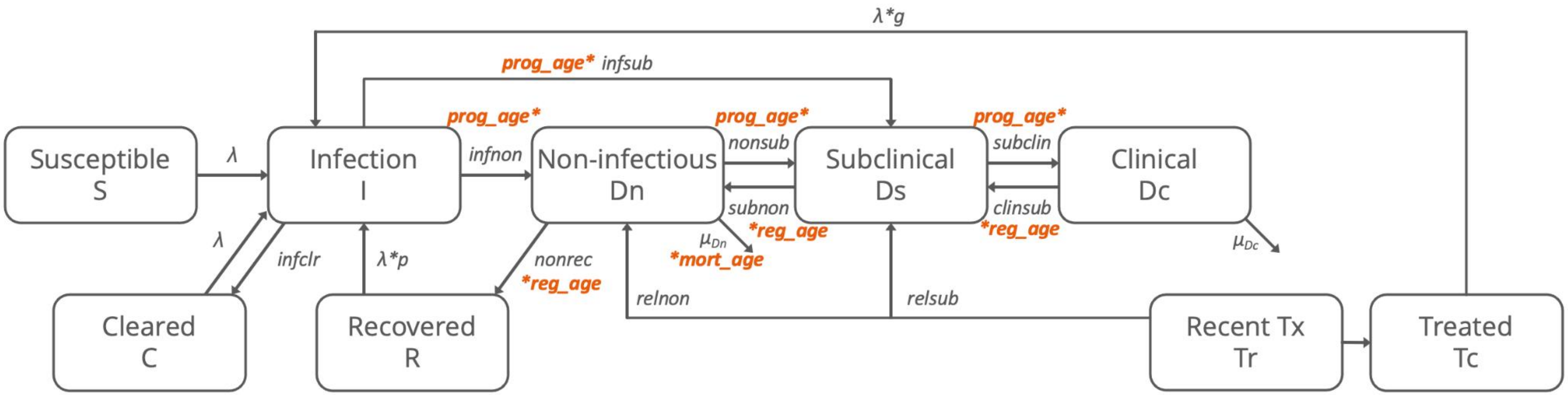

Figure S10. Age adjustments to transitions in the TB dimension. Detailed treatment pathways are shown in TB dimension. TB disease state and parameter naming follow conventions from research over the past decade; results are reported using current WHO terminology.

2.4 EQUATIONS

Equations incorporating age are shown above in Section 1.3.

2.5 PARAMETERS

Table S7. Age and TB interaction parameters

Parameter	Description	Prior/Value	Age-dependent?	Time-dependent?	Reference
prog_age	Relative progression of disease in children	0-14 years: 0.5 - 1.0<br>15+ years: 1.0	Yes	No	Assumption
reg_age	Relative regression of disease in children	1/prog_age	Yes	No	Assumption
mort_age	Relative non-infectious TB mortality in children	0-14 years: 1.0<br>15+ years: 0.0	Yes	No	Assumption

dx_age	Relative diagnostic rate in children	0-14 years: 0.25 - 1.0 15+ years	Yes	No	Assumption

- Decreased transmission in children is modelled with the use of age-specific contact matrices for each country.
- An adjustment factor (*prog_age*) is applied to parameters *nonsub* and *subclin* to account for the lower progression of disease from non-infectious (Dn) to asymptomatic disease (Ds) and from asymptomatic disease (Ds) to symptomatic disease (Dc) in children compared to adults.
- An adjustment factor (*mort_age)* is applied to the μ_{Dn} parameter to only account for non-infectious TB mortality in children. This is done to account for increased TB mortality in children despite not observing a higher number of symptomatic cases compared to adults.
- An adjustment factor (*dx_age*) is applied to the *access* parameter to account for lower care-seeking behaviour/access to diagnostics in children compared to adults.
- An adjustment factor (*k*) is applied to account for lower relative infectivity in asymptomatic (Ds) and symptomatic disease (Dc) in children compared to adults (see Section 1.2.1 for more detail).

3 RISK DIMENSION: HIV IN SOUTH AFRICA

We modelled an additional key risk factor for TB in our three model countries. For South Africa, this was HIV.

3.1 OVERVIEW

- There are three compartments modelling HIV progression in this risk dimension: HIV uninfected (*HIV0*), HIV infected not on ART (*HIVnt*), and HIV infected on ART(*HIVart*) (shown in Figure S11).
- HIV transmission is not modelled. Instead, a HIV infection incidence data is used to simulate progression from HIV uninfected to HIV infected not on ART. Increased risk of death if infected with HIV, mitigated by ART by 90%. Individuals infected with HIV and receiving ART can discontinue ART treatment and regress to HIV infected not on ART.
- Progression from one HIV state to another can happen at any state of the natural history of TB.
- Depending on the HIV state, some parameters modelling TB natural history and treatment may increase or decrease (more detail in section 4.3)

3.2 STRUCTURE

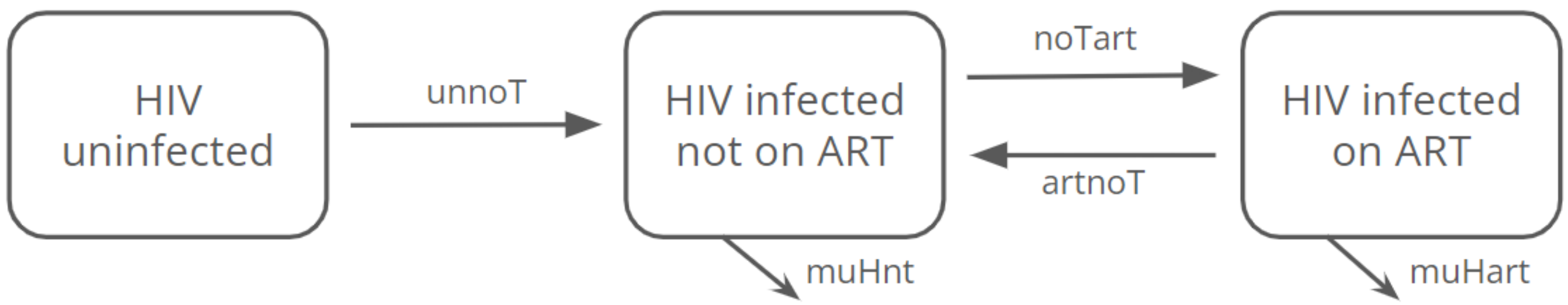

Figure S11. HIV dimension structure

When individuals are not infected with HIV, they are in the *HIV0* (HIV uninfected) compartment. They transition at rate *unnoT* to go to the *HIVnt* compartment, individuals that are HIV infected but are not on ART. In this compartment they can die at a rate *muHnt* or initiate treatment (*noTart*) and move into the *HIVart* compartment (HIV infected on ART). The individuals in this compartment can also die, but at a different rate (*muHart*), since being on ART decreases the mortality rate, or move back to the *HIVnt* compartment if there is discontinuation of treatment (*artnoT*).

- HIV parameters were mostly the same from previous paper using TBMod ((Sumner et al., 2024).
- HIV mortality in *HIVnt* was modified to be overall instead of by CD4 count. For that, the mean mortality rate was calculated to estimate HIV mortality for those not on treatment (*muHnt*) (Stover et al., 2019). The mean was assumed to be the maximum value and 0 assumed to be the minimum for the range 0 since the smallest values on the table from Stover et al. were close to it.
- *muHart* was calculated as the adjusted rate ratio of HIV mortality in *HIVart* compared to *HIVnt* times *muHnt*

3.3 INTERACTION BETWEEN HIV AND TB DIMENSION

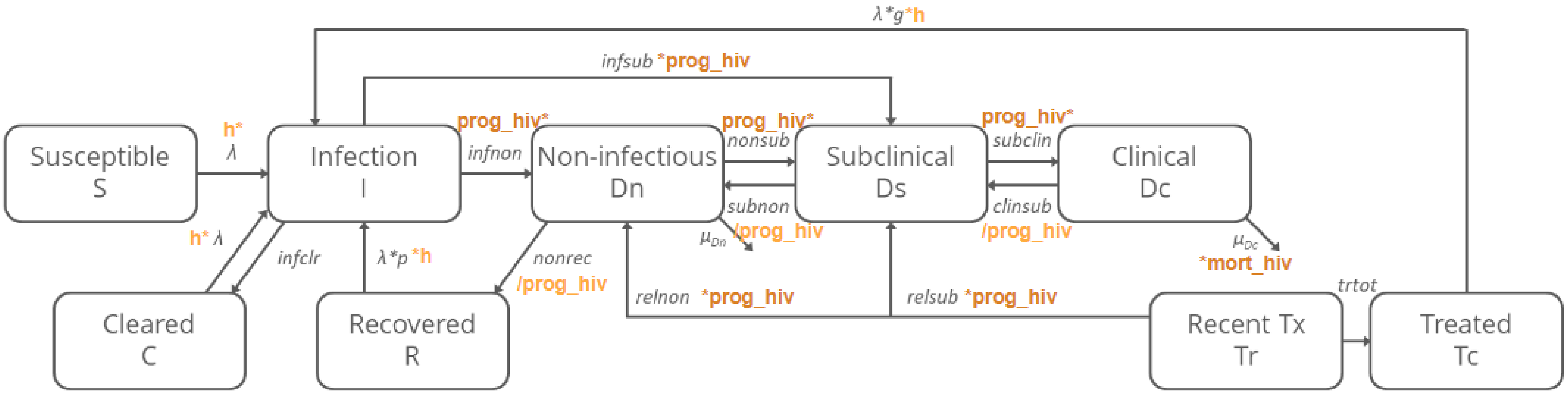

Drug-susceptible treatment initiation = treatment initiation rate * (1 – access to dst * probability of a positive dst)*dx_hiv
Drug-resistant treatment initiation = treatment initiation rate * access to dst * probability of a positive dst *dx_hiv
Successful treatment = completion rate * treatment success
Unsuccessful treatment = completion rate * (1 – treatment success – mortality on treatment * mortOT_hiv)
Mortality on treatment = completion rate * mortality on treatment *mortOT_hiv

Figure S12. HIV adjustments to transitions in the TB dimension. The diagram shows an overview of which TB parameters are affected by HIV. In particular, there are two types of parameters: those that increase the TB parameter (dark orange) and those that decrease the value of the TB parameter (light orange). Note the coloured arrows in the bottom of the figure represent the treatment pathways in order to show the impact of HIV in them. TB disease state and parameter naming follow conventions from research over the past decade; results are reported using current WHO terminology.

- TB progression (*prog_hiv*):
    - Despite the impact of HIV on TB having been investigated in multiple studies, the exact changes HIV has on the natural history of TB have not been fully assessed. For this reason and simplicity, we decided to increase TB progression and decrease natural recovery by the same value. More details on this assumption can be found on the following bullet points.
        - TB progression (*prog_hiv*): It is used to increase all the rates from infected to symptomatic (and relapse).
            - Interaction equation: TB_parameter *(hiv0str + *prog_hiv* * *prog_hivstr*).
            - Note: *hiv0str* is a parameter with value 1 for *HIV0* and value 0 for *HIVnt* and *HIVart*. This way when *prog_hivstr* is 0 for *HIV0* the value of the TB parameter will not be affected, while still maintain the ratio between progression in *HIVnt* and progression in *HIVart*.
            - From non-infectious to symptomatic, because specific data to inform each transition were not found so the incidence rates found for incidence of disease were assumed to be the same for everyone.
            - In the parameters from infected to non-infectious and asymptomatic, it was assumed that, due to a weaker immune system, they would progress faster once infected. No data was found specifically for each progression parameter, so it was assumed to use the same relative rate as other progression parameters.
            - In risk of relapse, HIV infected were also assumed to have a weaker immune system and hence, a higher risk of relapsing.
        - Natural recovery (1/*prog_hiv*):
            - Interaction equation: TB_parameter/(hiv0str + *prog_hiv* * *prog_hivstr*)
            - HIV infected have weaker immune systems i.e., the rate at which they can recover naturally from the disease would be lower, hence we decrease this rate.
            - Since no information was available on this parameter, it was assumed to decrease by the same amount as TB progression increases.
- HIV mortality (*mort_hiv*):
    - Interaction equation: TB_parameter * (hiv0str + *mort_hiv* * *mort_hivstr*)
    - HIV infected have weaker immune systems i.e., Increased risk of death by TB compared to HIV uninfected.
    - It only affects *Dc* because in *Dn* only children die, and although it is assumed to increase the risk equally between children and adults, the studies used to estimate the range only include adults, so we decided to assume this only for *Dc*.
- TB infectiousness (*h*):
    - Interaction equation: TB_parameter * (hiv0str + *h*)

   - Infectiousness is different between HIV+ and HIV- according to multiple papers (Crampin et al., 2006; Winter et al., 2020).
   - It was calculated assuming that HIV infected individuals were more likely to be smear negative and that smear negative TB was less infectious.
- TB diagnosis (*dx_hiv*):
   - Interaction equation: TB_parameter * (hiv0str + *dx_hiv* * *dx_hivstr*)
   - HIV infected individuals are more likely to be screened for TB and hence, more likely to start treatment.
- Mortality on TB treatment (*mortOT_hiv*):
   - Interaction equation: TB_parameter * (hiv0str + *mortOT_hiv* * *mortOT_hivstr*)
   - HIV infected individuals have a weaker immune system and, therefore, worse outcomes in TB treatment than HIV uninfected.
   - Both *HIVart* and *HIVnt* are assumed to be the same since in South Africa, the percentage of people on ART is higher than 80% and those that are not on ART and start treatment for TB are suggested to start also on ART between 2 and 8 weeks after initiation of TB treatment, thus, it was assumed for simplicity that both HIV infected states would have the same impact on treatment mortality (South Africa Department of Health, 2014).
- TB treatment success:
   - Not affected by HIV status
   - A study conducted in South Africa suggested that TB treatment success was not significantly different between HIV unifected and HIV infected (Nyasulu et al., 2022). On the other hand, a study in Kenya suggested that treatment success was significantly different between both groups (Owiti et al., 2015). Nonetheless, ART coverage in Kenya is much lower than in South Africa, and since neither study stratified by ART status, this would suggest that perhaps HIV infected not on ART may have worse outcomes but TB treatment success on HIV infected on ART is similar to HIV uninfected (Nyasulu et al., 2022; Owiti et al., 2015). As mentioned in "TB treatment mortality" section, ART coverage in South Africa is high and those not on ART when they initiate TB treatment are linked to HIV care (South Africa Department of Health, 2014). For these reasons and simplicity of the model, it was assumed that TB treatment success did not differ by HIV status.
- TB treatment completion
   - Not affected by HIV status
   - A study in South Africa showed similar loss of people in both HIV unifected and HIV infected groups (Naidoo et al., 2017).

3.4 EQUATIONS

Below shows the equations used in the risk dimension: HIV in South Africa. On treatment equations are not included, due to there being no differences from core in section 1.4. Differences from core are noted using orange.

3.4.1 EXPRESSIONS

lambda_all = pT * cscal* pEC * k * (hiv0str - h) * ((t*(Ds_s_N + Ds_s_P) + (Dc_s_N +Dc_s_P) + (relfit *(t*(Ds_r_N + Ds_r_P) + (Dc_r_N +Dc_r_P)))/N)

lambda_s = pT * cscal* pEC * k * (hiv0str - h) * ((t*(Ds_s_N + Ds_s_P) + (Dc_s_N +Dc_s_P)/N)

lambda_r = pT * cscal * pEC * k * (hiv0str - h) * (relfit * (t*(Ds_r_N + Ds_r_P) + (Dc_r_N +Dc_r_P))/N)

treat_init = relinit * tinit * eta* dx_age * (hiv0str + dx_hiv * dx_hivstr)

3.4.2 HIV DIMENSION EQUATIONS

dHIV0/dt = unnoT * HIV0

dHIVnt/dt = + artnoT * HIVart - muHnt * HIVnt - age_noTart * noTart * HIVnt

dHIVart/dt = + age_noTart * noTart * HIVnt - artnoT * HIVart - muHnt * 0.1 * HIVart

3.4.3 NEVER TREATED

dS_N/dt = -(treat_init + lamda_all)*S_N

dC_N/dt = infclr*(I_s_N + I_r_N) - (treat_init + lambda_all)*C_N

dR_N/dt = nonrec*(1/prog_age)/(hiv0str + prog_hiv*prog_hivstr)*(Dn_s_N + Dn_r_N) - (treat_init + lambda_all*p)*R_N

dI_s_N/dt = lambda_s*(S_N + C_N + p*R_N) - prog_age*(hiv0str + prog_hiv*prog_hivstr)*(infnon + infsub)*I_s_N - (treat_init + lambda_r+infclr)*I_s_N

dI_r_N/dt = lambda_r*(S_N + C_N + p*R_N + I_s_N) - prog_age*(hiv0str + prog_hiv*prog_hivstr)*(infnon + infsub)*I_r_N – (treat_init+infclr)*I_r_N

dDn_s_N/dt = infnon*prog_age*(hiv0str + prog_hiv*prog_hivstr)*I_s_N + subnon*(1/prog_age)/(hiv0str + prog_hiv*prog_hivstr)*Ds_s_N - nonrec*(1/prog_age)/(hiv0str + prog_hiv*prog_hivstr)*Dn_s_N - nonsub*prog_age*(hiv0str + prog_hiv*prog_hivstr)*Dn_s_N - muDn*mort_age*Dn_s_N - treat_init*Dn_s_N

dDn_r_N/dt = infnon*prog_age*(hiv0str + prog_hiv*prog_hivstr)*I_r_N + subnon*(1/prog_age)/(hiv0str + prog_hiv*prog_hivstr)*Ds_r_N - nonrec*(1/prog_age)/(hiv0str + prog_hiv*prog_hivstr)*Dn_r_N - nonsub*prog_age*(hiv0str + prog_hiv*prog_hivstr)*Dn_r_N - muDn*mort_age*Dn_r_N - treat_init*Dn_r_N

dDs_s_N/dt = infsub*prog_age*(hiv0str + prog_hiv*prog_hivstr)*I_s_N + nonsub*prog_age*(hiv0str + prog_hiv*prog_hivstr)*Dn_s_N + clinsub*(1/prog_age)/(hiv0str + prog_hiv*prog_hivstr)*Dc_s_N - subnon*(1/prog_age)/(hiv0str + prog_hiv*prog_hivstr)*Ds_s_N - subclin*prog_age*(hiv0str + prog_hiv*prog_hivstr)*Ds_s_N - treat_init*Ds_s_N

dDs_r_N/dt = infsub*prog_age*(hiv0str + prog_hiv*prog_hivstr)*I_r_N + nonsub*prog_age*(hiv0str + prog_hiv*prog_hivstr)*Dn_r_N + clinsub*(1/prog_age)/(hiv0str + prog_hiv*prog_hivstr)*Dc_r_N - subnon*(1/prog_age)/(hiv0str + prog_hiv*prog_hivstr)*Ds_r_N - subclin*prog_age*(hiv0str + prog_hiv*prog_hivstr)*Ds_r_N - treat_init*Ds_r_N

dDc_s_N/dt = subclin*prog_age*(hiv0str + prog_hiv*prog_hivstr)*Ds_s_N - clinsub*(1/prog_age)/(hiv0str + prog_hiv*prog_hivstr)*Dc_s_N - muDc*(hiv0str + mort_hiv*mort_hivstr)*Dc_s_N - treat_init*Dc_s_N

dDc_r_N/dt = subclin*prog_age*(hiv0str + prog_hiv*prog_hivstr)*Ds_r_N - clinsub*(1/prog_age)/(hiv0str + prog_hiv*prog_hivstr)*Dc_r_N - muDc*(hiv0str + mort_hiv*mort_hivstr)*Dc_r_N - treat_init*Dc_r_N

3.4.4 PREVIOUSLY TREATED

dS_P/dt = comp_st*ST_S + comp_rt*RT_S - (treat_init + lambda_all)*S_P

dC_P/dt = infclr*(I_s_P + I_r_P) + comp_st*(ST_C + succ_stI*(ST_I_s + succ_strs*ST_I_r)) + comp_rt*(RT_C + succ_rtI*(RT_I_s + RT_I_r)) - (treat_init + lambda_all)*C_P

dR_P/dt = nonrec*(1/prog_age)/(hiv0str + prog_hiv*prog_hivstr)*(Dn_s_P + Dn_r_P) + comp_rt* + comp_st*(ST_R+succ_stDn*(ST_Dn_s + succ_strs*ST_Dn_r)) + comp_rt*(RT_R+succ_rtDn*(RT_Dn_s +RT_Dn_r)) - (treat_init + lambda_all*p)*R_P

dI_s_P/dt = (1-succ_stI)*(comp_st*ST_I_s + comp_rt*RT_I_s) +lambda_s*(S_P +C_P + p*R_P + g*Tc_P) - infclr*I_s_P - infnon*prog_age*(hiv0str + prog_hiv*prog_hivstr)*I_s_P - infsub*prog_age*(hiv0str + prog_hiv*prog_hivstr)*I_s_P – (treat_init + lambda_r)*I_s_P

dI_r_P/dt = comp_st*(1-succ_stI*succ_strs)*ST_I_r + comp_rt*(1-succ_rtI)*RT_I_r + lambda_r*(S_P + C_P + p*R_P + I_s_P + r*Tc_P) - (infclr + prog_age*(hiv0str + prog_hiv*prog_hivstr)*(infnon + infsub)*I_r_P + treat_init)*I_r_P

dDn_s_P/dt = infnon*prog_age*(hiv0str + prog_hiv*prog_hivstr)*I_s_P + subnon*(1/prog_age)/(hiv0str + prog_hiv*prog_hivstr)*Ds_s_P + comp_st*(((1-mort_stDn)*mort_age*(1-succ_stDn))+((1-mort_age)*(1-((1+succ_stDn)/2))))*ST_Dn_s + comp_rt*((1-mort_rtDn)*mort_age*(1-succ_rtDn))+((1-mort_age)*(1-((1+succ_rtDn)/2)))*RT_Dn_s + relnon_s*(hiv0str + prog_hiv*prog_hivstr)*Tr_s_P - nonrec*(1/prog_age)/(hiv0str + prog_hiv*prog_hivstr)*Dn_s_P - nonsub*prog_age*(hiv0str + prog_hiv*prog_hivstr)*Dn_s_P - muDn*mort_age*(hiv0str + mort_hiv*mort_hivstr)*Dn_s_P - treat_init*Dn_s_P

dDn_r_P/dt = infnon*prog_age*(hiv0str + prog_hiv*prog_hivstr)*I_r_P + subnon*(1/prog_age)/(hiv0str + prog_hiv*prog_hivstr)*Ds_r_P + comp_st*(((1-mort_stDn)*mort_age*((1-succ_stDn)*succ_strs))+((1-mort_age)*(1-(((1+succ_stDn)/2)*succ_strs)))*ST_Dn_r + comp_rt*(((1-mort_rtDn)*mort_age*(1-succ_rtDn))+((1-mort_age)*(1-((1+succ_rtDn)/2)))*RT_Dn_r + relnon_r*(hiv0str + prog_hiv*prog_hivstr)*Tr_r_P - nonrec*(1/prog_age)/(hiv0str + prog_hiv*prog_hivstr)*Dn_r_P - nonsub*prog_age*(hiv0str + prog_hiv*prog_hivstr)*Dn_r_P - muDn*mort_age*(hiv0str+ mort_hiv*mort_hivstr)*Dn_r_P - (treat_init)*Dn_r_P

dDs_s_P/dt = infsub*prog_age*(hiv0str + prog_hiv*prog_hivstr)*I_s_P + nonsub*prog_age*(hiv0str + prog_hiv*prog_hivstr)*Dn_s_P + clinsub*(1/prog_age)/(hiv0str + prog_hiv*prog_hivstr)*Dc_s_P + comp_st*(1-succ_stDs)*ST_Ds_s + comp_rt*(1-succ_rtDs)*RT_Ds_s + relsub_s*(hiv0str + prog_hiv*prog_hivstr)*Tr_s_P - subnon*(1/prog_age)/(hiv0str + prog_hiv*prog_hivstr)*Ds_s_P - subclin*prog_age*(hiv0str + prog_hiv*prog_hivstr)*Ds_s_P - treat_init*Ds_s_P

dDs_r_P/dt = infsub*prog_age*(hiv0str + prog_hiv*prog_hivstr)*I_r_P + nonsub*prog_age*(hiv0str + prog_hiv*prog_hivstr)*Dn_r_P + clinsub*(1/prog_age)/(hiv0str + prog_hiv*prog_hivstr)*Dc_r_P + comp_st*(1-succ_stDs*succ_strs)*ST_Ds_r + comp_rt*(1-succ_rtDs)*RT_Ds_r + relsub_r*(hiv0str + prog_hiv*prog_hivstr)*Tr_r_P + comp_st*(1-succ_stDs*succ_strs)*ST_Ds_r +

comp_rt*(1-succ_rtDs)*RT_Ds_r - subnon*(1/prog_age)/(hiv0str + prog_hiv*prog_hivstr)*Ds_r_P - subclin*prog_age*(hiv0str + prog_hiv*prog_hivstr)*Ds_r_P - treat_init*Ds_r_P

dDc_s_P/dt = subclin*prog_age*(hiv0str + prog_hiv*prog_hivstr)*Ds_s_P + comp_st*(1-(mort_stDc*(hiv0str+mortOT_hiv*mortOT_hivstr)))*(1-succ_stDc)*ST_Dc_s + comp_rt*(1-(mort_rtDc*(hiv0str+mortOT_hiv*mortOT_hivstr)))*(1-succ_rtDc)*RT_Dc_s - clinsub*(1/prog_age)/(hiv0str + prog_hiv*prog_hivstr)*Dc_s_P - muDc*(hiv0str + mort_hiv*mort_hivstr)*Dc_s_P - treat_init*Dc_s_P

dDc_r_P/dt = subclin*prog_age*(hiv0str + prog_hiv*prog_hivstr)*Ds_r_P + comp_st*(1-(mort_stDc*(hiv0str+mortOT_hiv*mortOT_hivstr)))*(1-succ_stDc*succ_strs)*ST_Dc_r + comp_rt*(1-(mort_rtDc*(hiv0str+mortOT_hiv*mortOT_hivstr)))*(1-succ_rtDc)*RT_Dc_r - clinsub*(1/prog_age)/(hiv0str + prog_hiv*prog_hivstr)*Dc_r_P - muDc*(hiv0str + mort_hiv*mort_hivstr)*Dc_r_P - treat_init*Dc_r_P

dTr_s_P/dt = comp_st*succ_stDs*ST_Ds_s + comp_rt*succ_rtDs*RT_Ds_s + comp_st*succ_stDc*ST_Dc_s + comp_rt*succ_rtDc*RT_Dc_s - trtot*Tr_s_P - relnon_s*(hiv0str + prog_hiv*prog_hivstr)*Tr_s_P - relsub_s*(hiv0str + prog_hiv*prog_hivstr)*Tr_s_P

dTr_r_P/dt = comp_st*succ_stDs*succ_strs*ST_Ds_r + comp_rt*succ_rtDs*RT_Ds_r + comp_st*succ_stDc*succ_strs*ST_Dc_r + comp_rt*succ_rtDc*RT_Dc_r - trtot*Tr_r_P - relnon_r*(hiv0str + prog_hiv*prog_hivstr)*Tr_r_P - relsub_r*(hiv0str + prog_hiv*prog_hivstr)*Tr_r_P

dTc_P/dt = comp_st*ST_Tc + comp_rt*RT_Tc + trtot*(Tr_s_P +Tr_r_P) - (treat_init + lambda_all*r)*Tc_P

3.5 PARAMETERS

The parameter table details the HIV dimension parameters, which include HIV and TB specific interaction parameters; categorised by prior/value, age and time dependency. References are given where applicable.

Table S8. HIV dimension parameters

Parameter	Description (units)	Prior/Value	Age-dependent?	Time-dependent?	Reference
HIV parameters					

	Incidence rate of HIV (per person year)	Trend data	Yes	Yes	(Stover & Glaubius, 2024)
unnoT[1]	Multiplier of the incidence rate of HIV	0 - 2.5	No	No	Fitted in calibration
noTart	Rate of treatment initiation (per year)	0.1 - 3	Yes	Yes	
noTart2010[2]	Multiplier in 2010 of noTart	0.1 - 3	No	No	
age_noTart	Relative risk of treatment initiation by age	0.75	0-14	No	(Sumner et al., 2024)
		1	15+	No	
artnoT	Rate of treatment discontinuation (per year)	0.092	0-14	No	(Mberi et al., 2015; Van Cutsem et al., 2011)
		0.074	+15	No	(Chandiwana et al., 2018; Fenner et al., 2010; Sengayi et al., 2013)
muHnt	Death rate of people with HIV not on treatment (per year)	0 - 0.304	No	No	(Stover et al., 2019)
-	Deaths rate ratio HIV+ on ART vs Not on treatment (per year)	0.1	No	No	(Badri et al., 2004)
TB-HIV interaction parameters					
prog_hiv[3]	Rate ratio of TB progression	2.48 - 8	No	No	(Corbett et al., 2003; Sonnenberg et al., 2005)
1/prog_hiv[3]	Natural recovery	Inverse of TB progression	No	No	Assumption
mort_hiv	TB mortality in Dc	2.7 - 27.4	No	No	(Mukadi et al., 2001)
mortOT_hiv	Mortality on TB treatment	1.15 - 1.56	No	No	(World Health Organization, 2023)
dx_hiv[4]	TB treatment initiation	2 - 4.6	No	No	(Claassens et al., 2016)
h	TB infectiousness	0.86	No	No	(Behr et al., 1999; Dye et al., 1999; Khan et al., 2017)
HIV stage parameters					
prog_hivstr	For TB transmission	*HIV0* = 0	No	No	(Lawn et al., 2006)
		HIVnt = 1			
		HIVart = 0.09-0.38			
mort_hivstr	For TB mortality	*HIV0* = 0	No	No	(Yang et al., 2023)
		HIVnt = 1			

		HIVart = 0.19-0.82			
mortOT_hivstr	For mortality on TB treatment	*HIV0* = 0	No	No	Assumption
		HIVnt = 1			
		HIVart = 1			
dx_hivstr	For TB treatment	*HIV0* = 0	No	No	Assumption
		HIVnt = 1			
		HIVart = 1			

[1] HIV transmission is not modelled directly. Instead, a file containing the incidence rate of HIV (per person-year) across ages and time is introduced. This file is obtained from the model Spectrum. The purpose of unnoT is to multiply the rates in that file to adjust the HIV prevalence in the model to that observed in UNAIDS (Joint United Nations Programme on HIV/AIDS, 2021, 2023).
[2] Treatment initiation (*noTart*) varies by age and time. In order to model the changes in time of ART introduction, we added a multiplier called *noTartmul*. This multiplier has specified 3 timepoints: 2003, 2010 and 2020. For 2003 and 2020 the values are fixed. While in 2010, the value is fitted during calibration.
[3] Both TB progression and natural recovery use the same parameter name because prog_hiv is sampled and its inverse is used in the interaction with the respective TB parameter.
[4] The lower bound of dx_hiv is assumed to be lower than in the paper because the study only included places in South Africa with >400 notifications/100,000. Therefore, the range could be lower across the country since areas with less TB may be less focused on TB diagnosis in people living with HIV.

4 RISK DIMENSION: NUTRITION IN INDIA

We modelled an additional key risk factor for TB in our three model countries. For India, this was undernutrition.

4.1 OVERVIEW

- An increased risk of TB disease, unsuccessful TB treatment, and TB mortality due to undernutrition—based on previous modelling exercises (McQuaid et al., 2025)—was captured by dividing the risk dimension in TBMod into four Body Mass Index (BMI) states:
    - Overweight or obese (>25.0 kg/m^2)
    - Normal BMI (18.5-24.9 kg/m^2)
    - Mild undernutrition (17.0-18.4 kg/m^2)
    - Moderate or severe undernutrition(<17 kg/m^2)
- BMI distribution in the population from 1974 to 2016 was defined based on data from the Global Health Observatory and the India Demographic and Health Survey datasets (International Institute for Population Sciences, 2022; World Health Organization, 2025). Flows between BMI strata were not dynamically modelled in the framework.

4.2 STRUCTURE

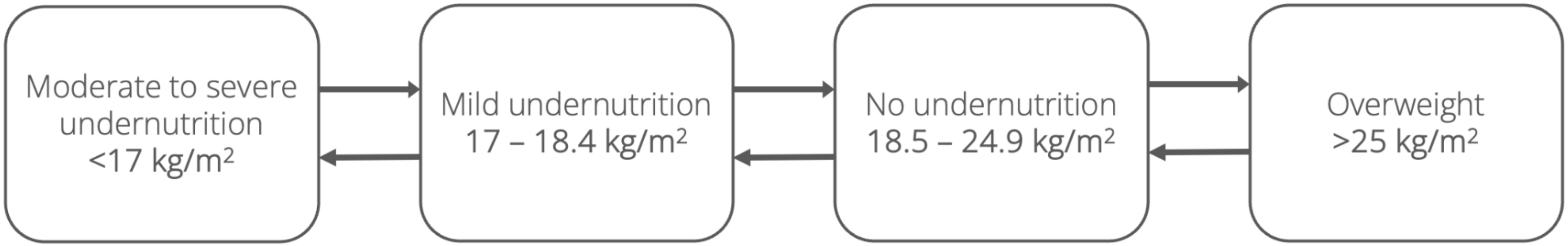

Figure S13. Nutrition dimension structure

4.3 INTERACTION BETWEEN NUTRITION AND TB DIMENSION

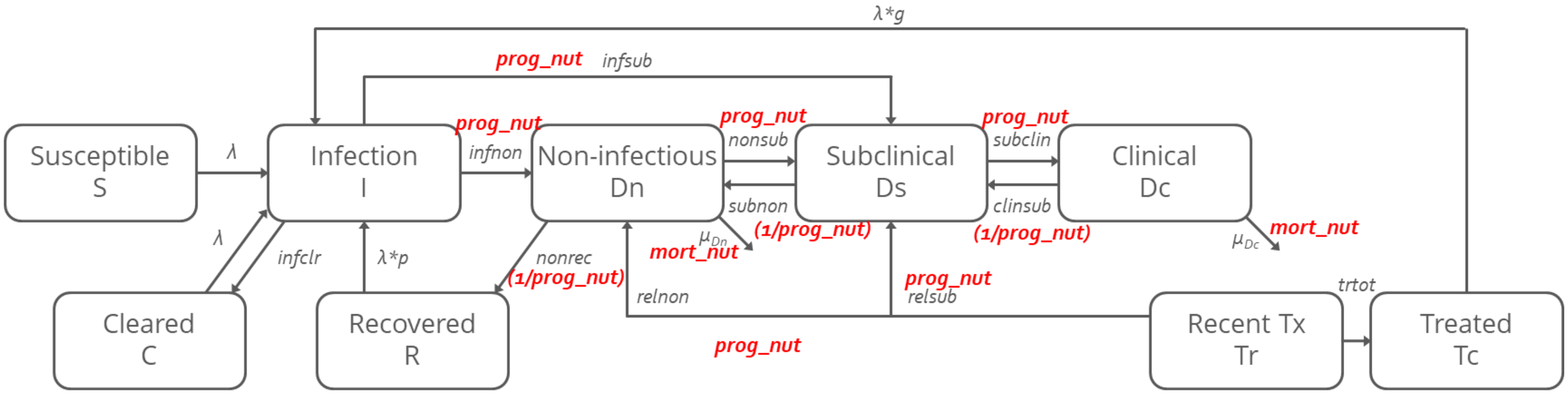

Drug-susceptible treatment initiation = treatment initiation rate * (1 – access to dst * probability of a positive dst)
Drug-resistant treatment initiation = treatment initiation rate * access to dst * probability of a positive dst
Successful treatment = completion rate * treatment success***tx_nut**
Unsuccessful treatment = completion rate * (1 – treatment success***tx_nut** – mortality on treatment ***mort_nut**)
Mortality on treatment = completion rate * mortality on treatment***mort_nut**

Figure S14. Nutrition adjustments to transitions in the TB dimension. TB disease state and parameter naming follow conventions from research over the past decade; results are reported using current WHO terminology.

- We assumed risk of progression to TB disease (*prog_nut*), treatment outcomes (*unsucc_nut*/*tx_nut*), and TB mortality (*mort_nut*) varied by BMI status. Adjustments are made by multiplying a given progression parameter that determines flow (e.g. *nonsub*) with the relative risks (*prog_nut*, relevant for nonsub, *tx_nut* or *mort_nut*, as applicable) experienced by people in particular BMI strata.

4.4 EQUATIONS

Below shows the equations used in the risk dimension: Undernutrition in India. On treatment equations are not included, due to there being no differences from core in section 1.4. Differences from core are noted using orange.

4.4.1 EXPRESSIONS

lambda_all = cscal * pT * k * C • (t*(Ds_s_N + Ds_s_P) + (Dc_s_N +Dc_s_P) + (relfit *(t*(Ds_s_N + Ds_s_P) + (Dc_s_N +Dc_s_P))))

lambda_s = cscal * pT * k * C • (t*(Ds_s_N + Ds_s_P) + (Dc_s_N +Dc_s_P))

lambda_r = cscal * pT * k * C • (relfit * (t*(Ds_s_N + Ds_s_P) + (Dc_s_N +Dc_s_P)))

treat_init = relinit * tinit * eta * dx_age

4.4.2 NUTRITION DIMENSION EQUATIONS

dover/dt = NA

dnormal/dt = NA

dmild/dt = NA

dmoderate/dt = NA

4.4.3 NEVER TREATED

dS_N/dt = - (treat_init + lambda_all) * S_N

dC_N/dt = infclr * (I_s_N + I_r_N) - (treat_init + lambda_all) * C_N

dR_N/dt = nonrec *(1/prog_age) * (1/prog_nut)* (Dn_s_N + Dn_r_N) – (treat_init + lambda_all * p) * R_N

dI_s_N/dt = lambda_s * (S_N + C_N + p*R_N) – (treat_init + infclr + lambda_r + (infnon + infsub * prog_nut) * prog_age) * I_s_N

dI_r_N/dt = lambda_r * (S_N + C_N + p*R_N + I_s_N) – (treat_init + infclr + prog_age * (infnon+ infsub * prog_nut) * I_r_N

dDn_s_N/dt = infnon * prog_age * I_s_N + subnon * (1/prog_age) * (1/prog_nut) * Ds_s_N – (treat_init +nonrec * (1/prog_age) * (1/prog_nut) + nonsub * prog_age * prog_nut + muDn * mort_age * mort_nut) * Dn_s_N

dDn_r_N/dt = infnon*prog_age*I_r_N + subnon*(1/prog_age)* (1/prog_nut)*Ds_r_N – (treat_init + nonrec*(1/prog_age)* (1/prog_nut) + nonsub*prog_age* prog_nut + muDn * mort_age * mort_nut) * Dn_r_N

dDs_s_N/dt = prog_age* prog_nut*(infsub*I_s_N + nonsub *Dn_s_N – subclin * Ds_s_N) + (1/prog_age)* [(1/prog_nut)*(clinsub*Dc_s_N – subnon) - treat_init] * Ds_s_N

dDs_r_N/dt = prog_age* prog_nut*(infsub* I_r_N + nonsub* Dn_r_N – subclin * Ds_r_N)+ (1/prog_age)* [(1/prog_nut)*(clinsub*Dc_r_N – subnon) – treat_init]*Ds_r_N)

dDc_s_N/dt = subclin*prog_age* prog_nut*Ds_s_N – (treat_init + (1/prog_nut)*clinsub*(1/prog_age) + mort_nut*muDc) * Dc_s_N

dDc_r_N/dt = subclin*prog_age* prog_nut*Ds_r_N – (treat_init + (1/prog_nut)*clinsub*(1/prog_age) + mort_nut*muDc)*Dc_r_N

4.4.4 PREVIOUSLY TREATED

dS_P/dt = comp_st * ST_S + comp_rt * RT_S – (treat_init + lambda_all) * S_P

dC_P/dt = infclr*(I_s_P + I_r_P) + comp_st*(ST_C + succ_stI* (ST_I_s + succ_strs*ST_I_r)) + comp_rt*(RT_C + succ_rtI*(RT_I_s + RT_I_r)) – (treat_init + lambda_all) * C_P

dR_P/dt = nonrec*(1/prog_age)* (1/prog_nut)*(Dn_s_P + Dn_r_P) + comp_st*(ST_R + succ_stDn*(ST_Dn_s + succ_strs*ST_Dn_r)) + comp_rt*(RT_R + succ_rtDn*(RT_Dn_s + RT_Dn_r)) – (treat_init + lambda_all*p) * R_P

dI_s_P/dt = comp_st*(1-succ_stI*(1/unsucc_nut))*ST_I_s + comp_rt*(1-succ_rtI*(1/unsucc_nut))*RT_I_s + lambda_s*(S_P + C_P + p*R_P + g*Tc_P) – (infclr + treat_init + lambda_r + prog_age* (infnon + infsub* prog_nut)*I_s_P

dI_r_P/dt = comp_st*(1-succ_stI*succ_strs*(1/unsucc_nut))*ST_I_r + comp_rt*(1-succ_rtI*(1/unsucc_nut))*RT_I_r + lambda_r*(S_P + C_P + p*R_P + I_s_P + g*Tc_P) – (treat_init + infclr + prog_age*(infnon + infsub* prog_nut))*I_r_P

dDn_s_P/dt = prog_nut*infnon*prog_age*I_s_P + (1/prog_nut)*subnon*(1/prog_age)*Ds_s_P + comp_st*(1-mort_nut*mort_stDn-succ_stDn*(1/unsucc_nut))*ST_Dn_s + comp_rt*(1-mort_nut*mort_rtDn-succ_rtDn*(1/unsucc_nut))*RT_Dn_s + prog_nut*relnon_s*Tr_s_P - (nonrec*(1/prog_age)* (1/prog_nut) + nonsub*prog_age* prog_nut + muDn*mort_age* mort_nut + treat_init)*Dn_s_P

dDn_r_P/dt = prog_nut *infnon*prog_age*I_r_P + (1/prog_nut)*subnon*(1/prog_age)*Ds_r_P + comp_st*(1-mort_nut *mort_stDn-succ_stDn*succ_strs*(1/unsucc_nut))*ST_Dn_r + comp_rt*(1-mort_nut *mort_rtDn-succ_rtDn(1/unsucc_nut))*RT_Dn_r + prog_nut *relnon_r*Tr_r_P - (nonrec*(1/prog_age)* (1/prog_nut) + nonsub*prog_age* prog_nut + muDn*mort_age* mort_nut + treat_init)*Dn_r_P

dDs_s_P/dt = prog_nut *prog_age*(infsub *I_s_P + nonsub*Dn_s_P) + (1/prog_nut)*clinsub*(1/prog_age)*Dc_s_P + *comp_st*(1-succ_stDs*(1/unsucc_nut))*ST_Ds_s + comp_rt*(1-succ_rtDs*(1/unsucc_nut))*RT_Ds_s + prog_nut *relsub_s*Tr_s_P - ((1/prog_nut)*subnon*(1/prog_age) + prog_nut *subclin*prog_age + treat_init)*Ds_s_P

dDs_r_P/dt = prog_nut *prog_age (*infsub *I_r_P + nonsub*Dn_r_P) + (1/prog_nut)*clinsub*(1/prog_age)*Dc_r_P + comp_st*(1-succ_stDs*succ_strs*(1/unsucc_nut))*ST_Ds_r + comp_rt*(1-succ_rtDs*(1/unsucc_nut))*RT_Ds_r + prog_nut *relsub_r*Tr_r_P - ((1/prog_nut)*subnon*(1/prog_age) - prog_nut *subclin*prog_age+ treat_init) *Ds_r_P

dDc_s_P/dt = prog_nut *subclin*prog_age*Ds_s_P + comp_st*((1-(mort_stDc*mort_nut))*(1-(succ_stDc*(1/unsucc_nut)))*ST_Dc_s + comp_rt*((1-(mort_rtDc*mort_nut))*(1-(succ_rtDc*(1/unsucc_nut)))*RT_Dc_s - ((1/prog_nut)*clinsub*(1/prog_age) + mort_nut *muDc + treat_init)*Dc_s_P

dDc_r_P/dt = prog_nut *subclin*prog_age*Ds_r_P + comp_st*((1-(mort_stDc*mort_nut))*(1-(succ_stDc*(1/unsucc_nut)*succ_strs)))*ST_Dc_r + comp_rt*((1-(mort_rtDc*mort_nut))*(1-(succ_rtDc*(1/unsucc_nut))))*RT_Dc_r - ((1/prog_nut)*clinsub*(1/prog_age) + mort_nut *muDc + treat_init)*Dc_r_P

dTr_s_P/dt = comp_st*succ_stDs*(1/unsucc_nut)*ST_Ds_s + comp_rt*succ_rtDs*(1/unsucc_nut)*RT_Ds_s + comp_st*succ_stDc*(1/unsucc_nut)*ST_Dc_s + comp_rt*succ_rtDc*(1/unsucc_nut)*RT_Dc_s – (trtot + prog_nut *relnon_s + prog_nut *relsub_s)*Tr_s_P

dTr_r_P/dt = comp_st*succ_stDs*succ_strs*(1/unsucc_nut)*ST_Ds_r + comp_rt*succ_rtDs*(1/unsucc_nut)*RT_Ds_r + comp_st*succ_stDc*succ_strs*(1/unsucc_nut)*ST_Dc_r + comp_rt*succ_rtDc*(1/unsucc_nut)*RT_Dc_r – (trtot + prog_nut *relnon_r + prog_nut *relsub_r)*Tr_r_P

dTc_P/dt = comp_st*ST_Tc + comp_rt*RT_Tc + trtot*Tr_s_P + trtot*Tr_r_P – (treat_init + lambda_all*g)*Tc_P

4.5 PARAMETERS

The relationship between BMI and TB incidence were replicated from the methods used by Clark et al., with minor revisions where necessary (Clark et al., 2025). The age-standardised proportion of the adult population >19 years within each strata was taken from the Global Health Observatory for the years 1975–2016 (World Health Organization, 2025). This consisted of the proportion of the population overweight (BMI ≥ 25.0 kg/m2) or underweight (BMI < 18.5 kg/m2), from which the proportion of normal weight was calculated. The proportion underweight was separated into moderate to severe underweigth and mild underweigth based on trends in the ratio of each in the India Demographic and Health Surveys from 2005–2006, 2015–2016 and 2019–2021, 2020–2022 weighted by the male:female ratio from UN population estimates and using the survey package in R. To estimate the proportion of children and adolescents in each BMI strata, we again used the Global Health Observatory (World Health Organization, 2025). We separated children aged 0–4 years into BMI strata based on standard deviations (SD) for weight-for-height of severe wasting (weight-for-height < -3SD), wasting (-3SD ≤ weight-for-height < - 2SD), normal (-2SD ≤ weight-for-height < +2SD) and overweight (weight-for-height ≥ +2SD), aligning with our four BMI strata. We note that estimates were only available for normal, wasting and severe wasting for the years 1999, 2006, 2014, 2015 and 2017, while for overweight these were available for 1994–2019.

We re-estimated the relationship between BMI and TB incidence from Lönnroth et al. using updated data from Cegielski et al. and assuming a uniform distribution for BMI within each BMI strata (Cegielski et al., 2012; Lönnroth et al., 2010). We used this to fit a log-linear model with a

dependent variable of TB incidence, an independent variable of BMI, and a categorical variable for the studies reported in Lönnroth et al., controlling for study-specific effects due to variation in study design and populations studied (Lönnroth et al., 2010).

Using the log-linear model, the BMI distributions from above, and estimated population-level TB incidence in 2020 (while 2005–2006 did not include primary sampling units in IPUMS and 1998–1999 did not include data for men), we estimated the expected TB incidence for each BMI strata weighted by the female:male ratio. We used this to calculate the risk ratio for TB in each strata compared to the strata with normal BMI, assuming that differences in risk of incident TB were a direct result of differences in rate of progression or reversion to TB disease. We estimated the mean and uncertainty intervals for this from 10,000 runs of the process.

Treatment outcome estimates were calculated using data collected from adults with microbiologically confirmed TB in five sites of the Regional Prospective Observational Research on Tuberculosis (RePORT) India consortium from 2015–2019.(Sinha et al., 2023) The primary exposure was BMI at treatment initiation and the outcome was a composite of death, treatment failure, and relapse/recurrence. We conducted multivariable analysis using Poisson regression, in which we used person-time as an offset to calculate adjusted incidence rate ratios (aIRR). We calculated person-time from the time of treatment initiation to the occurrence of the first mutually exclusive outcome of interest (failure/recurrence/death), loss to follow-up, or until right-censoring at 24 months of follow-up. In multivariable analysis, we included age, sex, and cough duration in the multivariable a priori and included other potential confounders based on prior literature that were significant at the $p<0.2$ level. The original publication calculated risk for different BMI categories than the ones required for this model. We recalculated the aIRR for the following BMI categories: moderately to severely thin (BMI < 17.0 kg/m^2), mildly thin (17.0 kg/m^2 ≤ BMI < 18.5 kg/m^2), normal (18.5 kg/m^2 ≤ BMI <25.0kg/m^2) and overweight to obese (BMI ≥ 25.0 kg/m^2). We assumed that the risk ratios for disease progression, reversion, and treatment outcomes were different by BMI strata, and were applied identically to adults, adolescents, and children.

The parameter table details the nutrition dimension parameters, which include nutrition and TB specific interaction parameters; categorised by prior/value, age and time dependency. References are given where applicable.

Table S9. Nutrition dimension parameters

Parameter	Description	Prior/Value	Age-dependent?	Time-dependent?	Reference
Nutrition parameters					
Rates between<br>● Moderate-mild<br>● Over-normal<br>● Normal-over	Rates of flow between BMI states per year (proportion of population moving from one state to the other)	Input from data	Yes	Yes	(International Institute for Population Sciences, 2022; World Health Organization, 2025)

Parameter	Description	Prior/Value	Age-dependent?	Time-dependent?	Reference
● Normal-mild<br>● Mild-normal<br>● Mild-moderate					
prog_nut	Relative risk of TB progression	Moderate: 1.25- 1.84<br>Mild: 1.12-1.47<br>Normal: 1<br>Overweight: 0.23-0.66	No	No	(International Institute for Population Sciences, 2022; McQuaid et al., 2025)
reg_nut	Relative risk of natural recovery	1/prog_nut	No	No	Assumption
mort_nut	Relative risk of TB mortality	Moderate: 1.84–6.48<br>Mild: 1<br>Normal: 1<br>Overweight:1	No	No	(McQuaid et al., 2025; Sinha et al., 2024)
unsucc_nut	Relative risk of treatment non-completion /unsuccessful treatment	Moderate: 1.09–2.31<br>Mild: 1<br>Normal: 1<br>Overweight: 1	No	No	
tx_nut	Relative risk of treatment success and/or completion	1/unsucc_nut	No	No	Assumption

5 RISK DIMENSION: INCARCERATION IN BRAZIL

5.1 OVERVIEW

There are three population risk strata that we are modelling TB transmission in: general population, currently incarcerated, and previously incarcerated. The model allows for movement and mixing between specific groups (described below).

- Prison: A correctional facility where individuals who are serving sentences, or awaiting trial are held.
- General population / community: Individuals who are not currently incarcerated, nor have been incarcerated within the last 7 years.
- Incarcerated persons: Individuals who are currently in prison. Incarcerated persons will have entered prison from the general population or from the previously incarcerated population.
- Previously incarcerated persons: Individuals who have been released from prison within the past 7 years. Previously incarcerated individuals will rejoin the general population after 7 years.
- Individuals can be incarcerated and released from prison at any stage of the TB pathway.
- To capture the higher risk of TB in prisons, currently incarcerated individuals will have an increased progression through disease states and a higher contact rate than the general population. The increased risk will be distributed equally between progression and contact rates to combat the uncertainty regarding the exact source of the elevated risk. Previously incarcerated individuals will have equal progression to the currently incarcerated individuals to account for their increased progression compared to the general population.

5.2 STRUCTURE

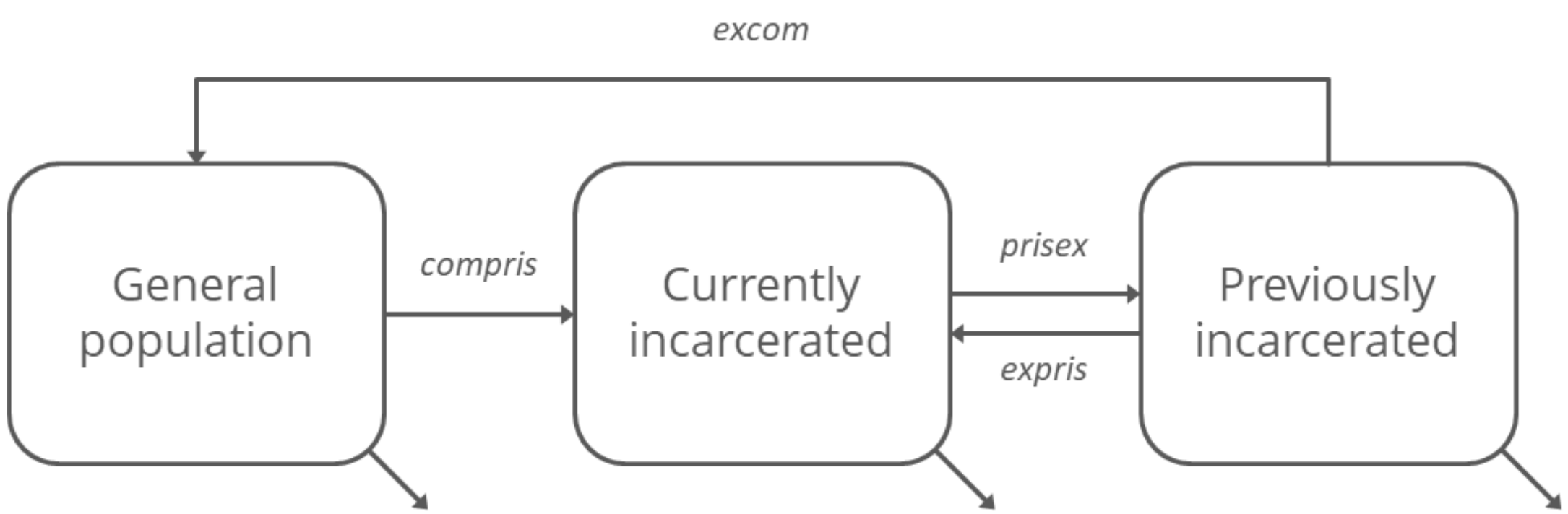

Figure S15. Prison dimension structure

5.3 INTERACTION OF PRISONS AND TB DIMENSION

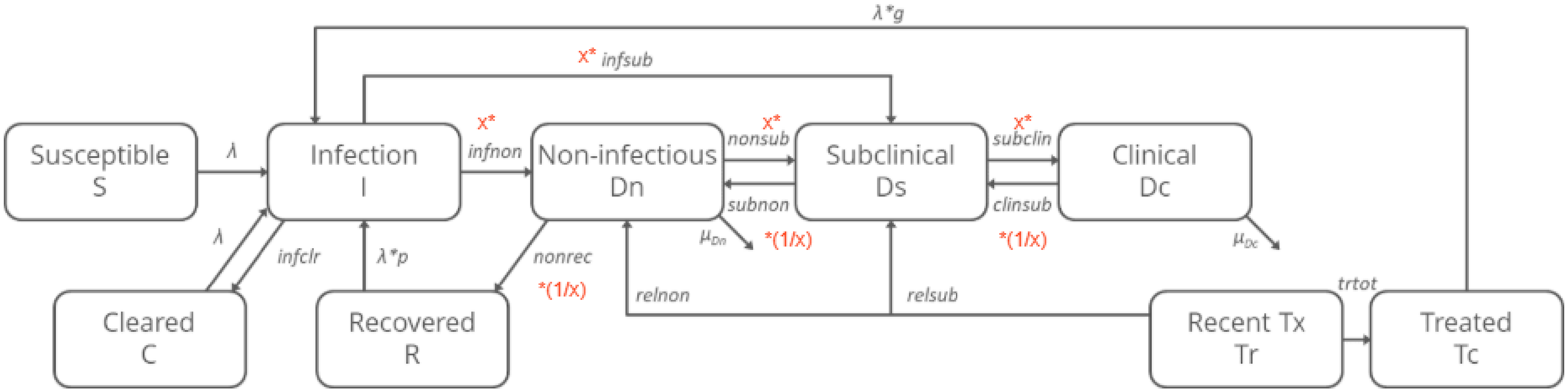

Figure 15. Prison adjustments to transitions in the TB dimension. The diagram shows an overview of which TB parameters are affected by incarceration. There are two types of parameters: those that increase the TB parameter and those that decrease the value of the TB parameter .Detailed treatment pathways are shown in Figure 4 and Figure 5.
X = prog_not_inprison+inprison*prog_inprison*prog_contRate_pri

5.4 EQUATIONS

Below shows the equations used in the risk dimension: Incarceration in Brazil. On treatment equations are not included, due to there being no differences from core in section 1.4. Differences from core are noted using yellow.

5.4.1 EXPRESSIONS

lambda_all = cscal * $C_{commexpris}$ * pT * mix_pri_cx * k * ((t*(Ds_s_N + Ds_s_P) + (Dc_s_N +Dc_s_P) + (relfit *(t*(Ds_r_N + Ds_r_P) + (Dc_r_N +Dc_r_P)))) + contactRate_pri_p * C_{prison} * pT * mix_pri_p * k * ((t*(Ds_s_N + Ds_s_P) + (Dc_s_N +Dc_s_P) + (relfit *(t*(Ds_r_N + Ds_r_P) + (Dc_r_N +Dc_r_P)))/N)

lambda_s_communityexpris = cscal * $C_{commexpris}$ * pT * mix_pri_cx*k * ((t*(Ds_s_N + Ds_s_P) + (Dc_s_N +Dc_s_P)/N)

lambda_r_communityexpris = cscal * $C_{commexpris}$ * pT * mix_pri_cx* k * (relfit * (t*(Ds_r_N + Ds_r_P) + (Dc_r_N +Dc_r_P))/N)

lambda_s_prison = contactRate_pri_p * C_{prison} * pT * mix_pri_cx*k * ((t*(Ds_s_N + Ds_s_P) + (Dc_s_N +Dc_s_P)/N)

lambda_r_prison = contactRate_pri_p * C_{prison} * pT * mix_pri_cx* k * (relfit * (t*(Ds_r_N + Ds_r_P) + (Dc_r_N +Dc_r_P))/N)

treat_init = relinit*tinit * eta* dx_age

5.4.2 PRISON DIMENSION EQUATIONS

dcommunity/dt = excom * exprsn - 1 * f * community

dexprsn/dt = exmortmult * 0.01696 * exprsn + 1 * prison - (excom + expris) * exprsn

dprison/dt = + expris*exprsn + 1*f*community - 1*prison

5.4.3 NEVER TREATED

dS_N/dt = - (treat_init + lamda_all)*S_N

dC_N/dt = infclr*(I_s_N + I_r_N) - (treat_init + lambda_all)*C_N

dR_N/dt = (nonrec*(1/prog_age)*(1/(prog_not_inprison+inprison*prog_inprison*prog_contRate_pri))** (Dn_s_N + Dn_r_N) - (treat_init + lambda_all*p)*R_N

dI_s_N/dt = lambda_s*(S_N + C_N + p*R_N) - infclr*I_s_N -
infnon*prog_age*(prog_not_inprison+inprison*prog_inprison*prog_contRate_pri)*I_s_N -
infsub*prog_age*(prog_not_inprison+inprison*prog_inprison*prog_contRate_pri)*I_s_N - (treat_init + lambda_r) *I_s_N

dI_r_N/dt = lambda_r*(S_N + C_N + p*R_N + I_s_N) - infclr*I_r_N -
infnon*prog_age*(prog_not_inprison+inprison*prog_inprison*prog_contRate_pri)*I_r_N -
infsub*prog_age*(prog_not_inprison+inprison*prog_inprison*prog_contRate_pri)*I_r_N - treat_init *I_r_N

dDn_s_N/dt = infnon*prog_age*(prog_not_inprison+inprison*prog_inprison*prog_contRate_pri)*I_s_N +
subnon*(1/prog_age)*(1/(prog_not_inprison+inprison*prog_inprison*prog_contRate_pri))**Ds_s_N - nonrec*(1/prog_age)
*(1/(prog_not_inprison+inprison*prog_inprison*prog_contRate_pri))**Dn_s_N -
nonsub*prog_age*(prog_not_inprison+inprison*prog_inprison*prog_contRate_pri)*Dn_s_N - muDn*mort_age*Dn_s_N -
treat_init*Dn_s_N

dDn_r_N/dt = infnon*prog_age*(prog_not_inprison+inprison*prog_inprison*prog_contRate_pri)*I_r_N +
subnon*(1/prog_age)*(1/(prog_not_inprison+inprison*prog_inprison*prog_contRate_pri))**Ds_r_N - nonrec*(1/prog_age)
*(1/(prog_not_inprison+inprison*prog_inprison*prog_contRate_pri))** Dn_r_N -
nonsub*prog_age*(prog_not_inprison+inprison*prog_inprison*prog_contRate_pri)*Dn_r_N - muDn*mort_age*Dn_r_N - treat_init
*Dn_r_N

dDs_s_N/dt = infsub*prog_age*(prog_not_inprison+inprison*prog_inprison*prog_contRate_pri)*I_s_N +
nonsub*prog_age*(prog_not_inprison+inprison*prog_inprison*prog_contRate_pri)*Dn_s_N +
clinsub*(1/prog_age)*(1/(prog_not_inprison+inprison*prog_inprison*prog_contRate_pri))**Dc_s_N -
subnon*(1/prog_age)*(1/(prog_not_inprison+inprison*prog_inprison*prog_contRate_pri))**Ds_s_N -
subclin*prog_age*(prog_not_inprison+inprison*prog_inprison*prog_contRate_pri)*Ds_s_N - treat_init *Ds_s_N

dDs_r_N/dt = infsub*prog_age*(prog_not_inprison+inprison*prog_inprison*prog_contRate_pri)*I_r_N +
nonsub*prog_age*(prog_not_inprison+inprison*prog_inprison*prog_contRate_pri)*Dn_r_N +
clinsub*(1/prog_age)*(1/(prog_not_inprison+inprison*prog_inprison*prog_contRate_pri))**Dc_r_N -
subnon*(1/prog_age)*(1/(prog_not_inprison+inprison*prog_inprison*prog_contRate_pri))**Ds_r_N -
subclin*prog_age*(prog_not_inprison+inprison*prog_inprison*prog_contRate_pri)*Ds_r_N - treat_init *Ds_r_N

dDc_s_N/dt = subclin*prog_age*(prog_not_inprison+inprison*prog_inprison*prog_contRate_pri)*Ds_s_N - clinsub*(1/prog_age)*(1/(prog_not_inprison+inprison*prog_inprison*prog_contRate_pri))**Dc_s_N - muDc*Dc_s_N - treat_init *Dc_s_N

dDc_r_N/dt = subclin*prog_age*(prog_not_inprison+inprison*prog_inprison*prog_contRate_pri)*Ds_r_N - clinsub*(1/prog_age)*(1/(prog_not_inprison+inprison*prog_inprison*prog_contRate_pri))**Dc_r_N - muDc*Dc_r_N - treat_init *Dc_r_N

5.4.4 PREVIOUSLY TREATED

dS_P/dt = comp_st*ST_S + comp_rt*RT_S - (treat_init + lambda_all)*S_P

dC_P/dt = infclr*(I_s_P + I_r_P) + comp_st*(ST_C + succ_stI*(ST_I_s + succ_strs*ST_I_r)) + comp_rt*(RT_C + succ_rtI*(RT_I_s + RT_I_r)) - (treat_init + lambda_all)*C_P

dR_P/dt = nonrec*(1/prog_age)*(1/(prog_not_inprison+inprison*prog_inprison*prog_contRate_pri))** (Dn_s_P + Dn_r_P) + comp_st*(ST_R + succ_stDn*(ST_Dn_s + succ_strs*ST_Dn_r)) + comp_rt*(RT_R + succ_rtDn*(RT_Dn_s + RT_Dn_r)) - (treat_init + lambda_all*p)*R_P

dI_s_P/dt = comp_st*(1-succ_stI)*ST_I_s + comp_rt*(1-succ_rtI)*RT_I_s + lambda_s*(S_P + C_P + p*R_P + r*Tc_P) - (infclr+ infnon*prog_age*(prog_not_inprison+inprison*prog_inprison*prog_contRate_pri))*I_s_P - infsub*prog_age*(prog_not_inprison+inprison*prog_inprison*prog_contRate_pri)*I_s_P - (treat_init +lambda_r)*I_s_P

dI_r_P/dt = comp_st*(1-succ_stI*succ_strs)*ST_I_r + comp_rt*(1-succ_rtI)*RT_I_r + lambda_r*(S_P + C_P + p*R_P + I_s_P + r*Tc_P) - infnon*prog_age*(prog_not_inprison+inprison*prog_inprison*prog_contRate_pri)*I_r_P - infsub*prog_age*(prog_not_inprison+inprison*prog_inprison*prog_contRate_pri)*I_r_P - treat_init *I_r_P

dDn_s_P/dt = infnon*prog_age*(prog_not_inprison+inprison*prog_inprison*prog_contRate_pri)*I_s_P + subnon*(1/prog_age)*(1/(prog_not_inprison+inprison*prog_inprison*prog_contRate_pri))**Ds_s_P + comp_st*(((1-mort_stDn)*mort_age*(1-succ_stDn))+((1-mort_age)*(1-((1+succ_stDn)/2))))*ST_Dn_s + comp_rt*((1-mort_rtDn)*mort_age*(1-succ_rtDn))+((1-mort_age)*(1-((1+succ_rtDn)/2)))*RT_Dn_s + relnon_s*Tr_s_P - nonrec*(1/prog_age)*

(1/(prog_not_inprison+inprison*prog_inprison*prog_contRate_pri))**Dn_s_P - nonsub*prog_age*(prog_not_inprison+inprison*prog_inprison*prog_contRate_pri)*Dn_s_P - muDn*mort_age*Dn_s_P- treat_init*Dn_s_P

dDn_r_P/dt = prog_age*(prog_not_inprison+inprison*prog_inprison*prog_contRate_pri)*(infnon*I_r_P - nonsub*Dn_r_P) + subnon*(1/prog_age)*(1/(prog_not_inprison+inprison*prog_inprison*prog_contRate_pri))**Ds_r_P + comp_st*(((1-mort_stDn)*mort_age*((1-succ_stDn)*succ_strs))+((1-mort_age)*(1-(((1+succ_stDn)/2)*succ_strs)))*ST_Dn_r + comp_rt*(((1-mort_rtDn)*mort_age*(1-succ_rtDn))+((1-mort_age)*(1-((1+succ_rtDn)/2)))*RT_Dn_r + relnon_r*Tr_r_P - nonrec*(1/prog_age)*(1/(prog_not_inprison+inprison*prog_inprison*prog_contRate_pri))**Dn_r_P - muDn*mort_age*Dn_r_P - treat_init *Dn_r_P

dDs_s_P/dt = prog_age*(prog_not_inprison+inprison*prog_inprison*prog_contRate_pri)*(infsub*I_s_P + nonsub*Dn_s_P - subclin*Ds_s_P) + (1/prog_age)*(1/(prog_not_inprison+inprison*prog_inprison*prog_contRate_pri))** (clinsub*Dc_s_P - subnon*Ds_s_P) + comp_st*(1-mort_stDc)*(1-succ_stDc)*ST_Ds_s + comp_rt*(1-succ_rtDs)*RT_Ds_s + relsub_s*Tr_s_P -treat_init*Ds_s_P

dDs_r_P/dt = prog_age*(prog_not_inprison+inprison*prog_inprison*prog_contRate_pri)* (infsub*I_r_P + nonsub*Dn_r_P - subclin*Ds_r_P) + (1/prog_age)*(1/(prog_not_inprison+inprison*prog_inprison*prog_contRate_pri))** (clinsub*Dc_r_P - subnon*Ds_r_P) + comp_st*(1-succ_stDs*succ_strs)*ST_Ds_r + comp_rt*(1-succ_rtDs)*RT_Ds_r + relsub_r* - treat_init *Ds_r_P

dDc_s_P/dt = subclin*prog_age*(prog_not_inprison+inprison*prog_inprison*prog_contRate_pri)*Ds_s_P + comp_st*(1-mort_stDc)*(1-succ_stDc)*ST_Dc_s + comp_rt*(1-mort_rtDc)*(1-succ_rtDc)*RT_Dc_s - clinsub*(1/prog_age)*(1/(prog_not_inprison+inprison*prog_inprison*prog_contRate_pri))**Dc_s_P - (muDc + treat_init)*Dc_s_P

dDc_r_P/dt = subclin*prog_age*(prog_not_inprison+inprison*prog_inprison*prog_contRate_pri)*Ds_r_P + comp_st*(1-mort_stDc)*(1-succ_stDc*succ_strs)*ST_Dc_r + comp_rt*((1-mort_rtDc)*(1-succ_rtDc)*RT_Dc_r - clinsub*(1/prog_age)*(1/(prog_not_inprison+inprison*prog_inprison*prog_contRate_pri))**Dc_r_P - muDc*Dc_r_P - treat_init *Dc_r_P

dTr_s_P/dt = comp_st*(succ_stDs*ST_Ds_s + succ_stDc*ST_Dc_s) + comp_rt*(succ_rtDs*RT_Ds_s + succ_rtDc*RT_Dc_s) - (trtot + relnon_s + relsub_s)*Tr_s_P

dTr_r_P/dt = comp_st*succ_strs*(succ_stDs*ST_Ds_r + succ_stDc*ST_Dc_r) + comp_rt*(succ_rtDs*RT_Ds_r + succ_rtDc*RT_Dc_r) - (trtot + relnon_r + relsub_r)*Tr_r_P

dTc_P/dt = comp_st*ST_Tc + comp_rt*RT_Tc + trtot*(Tr_s_P + Tr_r_P) - (treat_init + lambda_all*r)*Tc_P

5.5 PARAMETERS

The parameter table details the incarceration dimension parameters, which include general incarceration and TB specific interaction parameters; categorised by prior/value, age and time dependency. References are given where applicable.

Table S10. Incarceration dimension parameters

Parameter	Description (units)	Prior/Value	Age-dependent?	Time-dependent?	Reference
**Incarceration parameters**					
compris	Admission rate to prison from the general population (per year)	0	Yes: 0-14	No	(Liu et al., 2024)
		0.00018 - 0.00054	Yes: 15+	Yes (from 1990)	
		0.00122 - 0.00294		Yes (from 2020)	
		0.00084 - 0.00261		Yes (from 2024)	
expris	Admission to prison from previously incarcerated (per year)	0.126 - 0.325	No	No	(Liu et al., 2024)
prisex	Release from prison (per year)	0.479 - 1.201	No	Yes (from 1990)	(Liu et al., 2024)
		0.452 - 1.064		Yes (from 2015)	
		0.476 - 1.106		Yes (from 2024)	
excom	Ex-prison to the community (per year)	0.143	No	No	(Liu et al., 2024)
exmortmult	Multiplier on background mortality to allow previously incarcerated to have a higher mortality rate	0 - 0.3974	No	No	(Liu et al., 2024)
**TB-incarceration interaction parameters**					
prog_not_inprison	TB progression multiplier. This works in conjunction with prog_inprison to ensure TB progression in the community is 1*progression	*Community = 1*<br>*Prison = 0*<br>*Exprsn = 0*	No	No	Assumption
prog_inprison	TB progression multiplier, applied to all populations. This is then multiplied by the inprison parameter to ensure this	*1 - 2.5*	No	No	Assumption

	additional progression is applied only to previously incarcerated and incarcerated individuals				
1/prog_inprison	Natural recovery multiplier applied to all populations. This is then multiplied by the inprison parameter to ensure this reduced regression is applied only to previously incarcerated and incarcerated individuals	*Inverse of TB progression*	No	No	Assumption
inprison	Multiplier applied to TB progression to ensure increased progression and regression only affects the previously incarcerated and incarcerated populations	*Community = 0*<br>*Prison = 1*<br>*Exprsn = 1*	No	No	Assumption
prog_contRate	Multiplier applied to progression and contact rate in prisons equally. This is to ensure progression and contact rate in prisons change by the same proportion to spread the risk equally.	*1 - 2.5*	No	No	Assumption
inf_r	Contact rate for prisons	Prison = ((-155*(prog_inprison^2))+(583*prog_inprison)-410)*prog_contRate_pri<br>Community = 1<br>Exprsn = 1	No	No	Assumption

- Both TB progression and natural recovery use the same parameter name because *prog_not_inprison* and *prog_inprison* are sampled and its inverse is used in the interaction with the respective TB parameter.
- Prison dimension specific parameters include admission and release from prison. Individuals can move into the prison strata from the general population rate *compris*. Individuals in the previously incarcerated stratus can either be reincarcerated or eventually move back to the general population strata at rates and *expris* and *excom* respectively
- For the prison dimension, the background mortality parameter (*mu*) will be varied to capture the different mortality risk across the three populations: increased mortality in the previously incarcerated population compared to the community and currently incarcerated populations.
- TB mortality is constant across all population strata.
- The probability of transmission pT is the same in and out of prisons. Increased TB incidence in prison is accounted for by the increased mixing through the contact rate *contrate_p* which varies during calibration, as well as a multiplier (*prog_inprison*) and (*prog_not_inprison*) which has been added to the TB progression cascade to indicate increased TB progression in prisoners and ex prisoners. Similarly, to indicate slower TB clearance/recovery (*reg_inprison = 1/prog_inprison*) and (*reg_not_inprison = 1/prog_not_inprison*). A detailed description of mixing in and out of prison is provided below.

Mixing

In the Brazil country model, there are three population strata: community, currently incarcerated individuals, and individuals previously incarcerated within the last seven years. The community comprises individuals not currently incarcerated, nor incarcerated within the last seven years. Community and previously incarcerated individuals are able to mix with each other and this mixing is age dependent, while incarcerated individuals can only mix with each other and this mixing is homogeneous.

The force of infection between community and previously incarcerated individuals is expressed by the following:

(Eq1) $$\lambda = cscal * pT * \boldsymbol{C} \bullet \boldsymbol{inf}$$

with ***C* • *inf*** the matrix product of the contact matrix ***C*** [with elements $c_{r,i,j}$ for risk group r and age groups *i* and *j*] and a vector ***inf*** [with elements $inf_{r,i} = I_{r,i}/N_r$, with I infectious individuals (Ds + Dc) of risk group *r* and age group *i* and N_r the total number in risk group *r*]. Elements of both ***C*** and ***inf*** are ordered by risk group first and age group second (i.e. first 16 age groups for community and the 16 age groups for formerly incarcerated).

$$\begin{pmatrix} c_{comm,1,1} & \cdots & c_{comm,1,16} \\ \vdots & \ddots & \vdots \\ c_{comm,16,1} & \cdots & c_{comm,16,16} \\ c_{expris,1,1} & \cdots & c_{expris,1,16} \\ \vdots & \ddots & \vdots \\ c_{expris,16,1} & \cdots & c_{expris,16,16} \end{pmatrix} \begin{pmatrix} I_{comm,1}/N_{comm,1} \\ \vdots \\ I_{comm,16}/N_{comm,16} \\ I_{expris,1}/N_{expris,1} \\ \vdots \\ I_{expris,16}/N_{expris,16} \end{pmatrix} = \begin{pmatrix} c_{comm,1,1}I_{comm,1}/N_{comm,1} + \dots + c_{comm,1,16}I_{comm,16}/N_{comm,16} \\ \vdots \\ c_{comm,16,1}I_{comm,1}/N_{comm,1} + \dots + c_{comm,16,16}I_{comm,16}/N_{comm,16} \\ c_{expris,1,1}I_{expris,1}/N_{expris,1} + \dots + c_{expris,1,16}I_{expris,16}/N_{expris,16} \\ \vdots \\ c_{expris,16,1}I_{expris,1}/N_{expris,1} + \dots + c_{expris,16,16}I_{expris,16}/N_{expris,16} \end{pmatrix}$$

cscal is a contact scaling factor and will vary during calibration, while *pT* is the probability of transmission per respiratory contact between an infectious and healthy individual. *pT* has a fixed value of 0.0013 (0.008-0.0018) by the average number of social contacts per year (see note 1 in section 1.3).

The incarcerated population is modelled with homogenous mixing to reflect how interactions are unlikely age dependent, due to shared facilities, thereby assuming every inmate has an equal chance of interacting with another inmate. The incarcerated population can only mix with themselves. Contact with staff and the wider community has not been modelled for the incarcerated population because most interactions occur between incarcerated individuals, therefore external interactions would provide limited additional accuracy. The force of infection in prisons is estimated by the following:

(Eq2) $$\lambda_p = contrate_p * pT * I_p/N_p$$

whereby $contrate_p$ is the contact rate in prisons, pT the probability of infection per contact, I_p the infected population within prisons and N_p the overall population in prisons. This reflects homogenous mixing.

6 CALIBRATION

Calibration was performed using approximate Bayesian computation (ABC) Markov chain Monte Carlo (MCMC), seeded with manual calibrations, for each country. MCMC chains were run to at least 25,000 parameter sets and then thinned to select 500 parameter sets with posterior estimates consistent with all calibration targets.

6.1 CALIBRATION TARGETS

Table S11. Calibration targets for each country

Target	Description (units)	Year	Pop	Age	Country			Comments	Reference
					South Africa	India	Brazil		
**TB targets**									
TB incidence	Estimated incidence (all forms) per 100 000 population [subclin*Ds / population] (with adjustment for double counting)	2000	All	All	703 (387-1117)	264 (90-527)	n/a	Symptomatic incidence is currently closest to WHO estimates. Targets were adjusted to not include extrapulmonary TB.<br><br>Can apply a standard reduction to account for repeat episodes within the same individual/infection if needed.	(World Health Organization, 2023) [e_inc_100k]
			HIV+	All	529 (271-870)	n/a	n/a		(World Health Organization, 2023) [e_inc_tbhiv_100k]
		2007	Prison	All	n/a	n/a	1493 (1069-2118)		(Liu et al., 2024)
		2010	All	All	n/a	n/a	40(34-46)		(World Health Organization, 2023) [e_inc_100k]
		2019	Prison	All	n/a	n/a	1963 (1437-2677)		(Liu et al., 2024)
		2022	All	All	432 (280-614)	163 (139-189)	44 (37-51)		(World Health Organization, 2023) [e_inc_100k]
				0-14	146 (81-205)	74 (62-88)	10.5 (8.6-11.9)		(United Nations, n.d.; World Health Organization, 2023)

									[estimates disaggregated by age [best] / UN population (0-14)]
				15+	551 (316-783)	194 (161-228)	53 (45-61)		(United Nations, n.d.; World Health Organization, 2023) [estimates disaggregated by age [best] / UN population (15-99)]
			HIV+	All	235 (153-334)	n/a	n/a		(World Health Organization, 2023) [e_inc_tbhiv_100k]
TB mortality	Estimated mortality of TB cases (all forms) per 100 000 population [(muDn*Dn + muDc*Dc) / population]	2000	All	All	317 (199-461)	n/a	n/a		(World Health Organization, 2023) [e_mort_100k]
			HIV+	All	258 (144-405)	n/a	n/a		(World Health Organization, 2023) [e_mort_tbhiv_100k]
		2010	All	All	n/a	n/a	4.2 (3.7-4.7)		(World Health Organization, 2023) [e_mort_100k]
		2015	All	All	n/a	32 (25-40)	n/a		(World Health Organization, 2023) [e_mort_100k]
		2019	All	All	n/a	25 (19 -31)	n/a		(World Health Organization, 2023) [e_mort_100k]
		2022	All	All	90 (51-142)	n/a	6.2 (5.3-7.1)		(World Health Organization, 2023) [e_mort_100k]
			HIV+	All	52 (17-107)	n/a	n/a		(World Health Organization, 2023) [e_mort_tbhiv_100k]
TB notifications	Case notification rate, which is the total of new and relapse cases and	2000	All	All	321 (257-385)	177 (106.2-247)	n/a	Note values for access and diag differ across TB states.	(World Health Organization, 2023) [c_newinc_100k]

	cases with unknown previous TB treatment history per 100 000 population [tinit*relinit*(S+C+R+T+I+ Dn+Ds+Dc) / population]	2007	Prison	All	n/a	n/a	804 (643-965)	Notification rates for IND are adjusted by a reporting factor accounting for the mix in IND's public private TB diagnosis (Clark, Weerasuriya, et al., 2023)	(Liu et al., 2024)
		2010	All	All	n/a	n/a	39 (31.2-46.8)		(World Health Organization, 2023) [c_newinc_100k]
		2019	Prison	All	n/a	n/a	1303 (1042-1564)		(Liu et al., 2024)
		2022	All	All	344 (275-413)	182(145-218)	42 (33.6-50.4)		(World Health Organization, 2023) [c_newinc_100k]
			All	0-14	97 (77-116)	42(34-50)	6.3 (5-7.6)		(World Health Organization, 2023) [newrel_m014 + newrel_f014] / UN Population (0-14)
			All	15+	464 (372-558)	232 (186-239)	50.4 (40.3-60.5)		(World Health Organization, 2023) [newrel_m15plus + newrel_f15plus] / UN Population (15-99)
			HIV+	All	171 (137-206)	n/a	n/a		(World Health Organization, 2023) [newrel_hivpos] / UN Population (0-99)
TB prevalence	Prevalence of bacteriologically confirmed pulmonary TB per 100 000 population aged 15 years or over [((Ds+Dc) / N]	2019	All	All	680 (535-821)	n/a	n/a	Survey cases on treatment: 12.0% in India, 4.3% in South Africa. Targets were adjusted to not include them. India's targets were also adjusted to not include extrapulmonary TB. No data for Brazil	(World Health Organization, 2023) National survey reports
		2015	All	All	n/a	227 (152-381)	n/a		
		2021	All	All	n/a	225 (157-292)	n/a		
	Proportion of infectious TB prevalence that is asymptomatic	2019	All	15+	57.7 (51.4-64.0)	n/a	n/a		
		2021	All	15+	n/a	50.4 (36.1-79.7)	n/a		

	[Ds / (Ds+Dc * 100)]								
RR-TB	Estimated percentage of TB notifications with rifampicin resistant TB	2022	All	All	3.9 (3.12-4.68)	4 (3.2 - 4.8)	2.7 (0.7-7.7)	Confidence intervals were calculated as +/- 20% of value for ZAF and IND. Ranges for BRA were much larger so lower and upper bound estimates for new and previously treated were weighted by the proportion of cases that were new and previously treated.	(World Health Organization, 2023)
**HIV targets**									
HIV prevalence	%	2000	All	0-14	0.9 (0.5-1.2)	n/a	n/a		(Joint United Nations Programme on HIV/AIDS, 2021)
			All	15+	10.1 (6.1-13.8)	n/a	n/a		
		2022	All	0-14	1.4 (0.8-3.2)	n/a	n/a		(Joint United Nations Programme on HIV/AIDS, 2023)
			All	15+	16.1 (11.3-20.4)	n/a	n/a		
Percentage of PLHIV who are on ART	%	2010	All	0-14	24 (14-34)	n/a	n/a		(Joint United Nations Programme on HIV/AIDS, 2021)
			All	15+	23 (15-31)	n/a	n/a		
		2022	All	0-14	54 (34-100)				(Joint United Nations Programme on HIV/AIDS, 2023)
			All	15+	75 (53-98)	n/a	n/a		
HIV mortality rate	per 100,000	2000	All	All	289 (162-445)	n/a	n/a		(Joint United Nations Programme on HIV/AIDS, 2021)

		2022	All	All	72 (50-144)	n/a	n/a		(Joint United Nations Programme on HIV/AIDS, 2023)
**Nutrition targets**									
PAF	Prevalence of undernutrition and relative risk of TB due to undernutrition	2022	All	All	n/a	45.2 (17.0-71.0)	n/a		(Bhargava et al., 2022)
**Incarceration targets**									
Prison entry rate	Prison entry rates per 100k among population aged 15+ (years)	2016	All	15+	n/a	n/a	338 (210-468)		(Liu et al., 2024)
		2019	All	15+	n/a	n/a	442 (275-612)		
		2022	All	15+	n/a	n/a	347 (216-480)		
Prison prevalence	Incarceration prevalence per 100k among population aged 15+ (years)	2000	All	15+	n/a	n/a	189 (151-227)		(Liu et al., 2024)
		2019	All	15+	n/a	n/a	452 (362-542)		
		2022	All	15+	n/a	n/a	488 (390-586)		

7 REFERENCES

Andrews, J. R., Noubary, F., Walensky, R. P., Cerda, R., Losina, E., & Horsburgh, C. R. (2012). Risk of progression to active tuberculosis following reinfection with Mycobacterium tuberculosis. *Clinical Infectious Diseases: An Official Publication of the Infectious Diseases Society of America*, *54*(6), 784–791. https://doi.org/10.1093/cid/cir951

Badri, M., Bekker, L.-G., Orrell, C., Pitt, J., Cilliers, F., & Wood, R. (2004). Initiating highly active antiretroviral therapy in sub-Saharan Africa: an assessment of the revised World Health Organization scaling-up guidelines. *AIDS* , *18*(8), 1159–1168. https://doi.org/10.1097/00002030-200405210-00009

Behr, M. A., Warren, S. A., Salamon, H., Hopewell, P. C., Ponce de Leon, A., Daley, C. L., & Small, P. M. (1999). Transmission of Mycobacterium tuberculosis from patients smear-negative for acid-fast bacilli. *The Lancet*, *353*(9151), 444–449. https://doi.org/10.1016/s0140-6736(98)03406-0

Bhargava, A., Bhargava, M., Beneditti, A., & Kurpad, A. (2022). Attributable is preventable: Corrected and revised estimates of population attributable fraction of TB related to undernutrition in 30 high TB burden countries. *Journal of Clinical Tuberculosis and Other Mycobacterial Diseases*, *27*, 100309. https://doi.org/10.1016/j.jctube.2022.100309

Cauthen, G. M., Pio, A., & ten Dam, H. G. (2002). Annual risk of tuberculous infection. *Bulletin of the World Health Organization*, *80*(6). https://www.ncbi.nlm.nih.gov/pubmed/12132011

Cegielski, J. P., Arab, L., & Cornoni-Huntley, J. (2012). Nutritional risk factors for tuberculosis among adults in the United States, 1971-1992. *American Journal of Epidemiology*, *176*(5), 409–422. https://doi.org/10.1093/aje/kws007

Chandiwana, N., Sawry, S., Chersich, M., Kachingwe, E., Makhathini, B., & Fairlie, L. (2018). High loss to follow-up of children on antiretroviral

treatment in a primary care HIV clinic in Johannesburg, South Africa. *Medicine*, *97*(29), e10901. https://doi.org/10.1097/MD.0000000000010901

Claassens, M. M., van Schalkwyk, C., Dunbar, R., Ayles, H., & Beyers, N. (2016). Patient diagnostic rate as indicator of tuberculosis case detection, South Africa. *Emerging Infectious Diseases*, *22*(3), 535–537. https://doi.org/10.3201/eid2203.151618

Clark, R. A., Bakker, R., Alexander, P., Henry, R., White, R. G., Sinha, P., Houben, R., & McQuaid, C. F. (2025). Modelling the effect of nutritional shocks on tuberculosis in India. *In Preparation*.

Clark, R. A., Mukandavire, C., Portnoy, A., Weerasuriya, C. K., Deol, A., Scarponi, D., Iskauskas, A., Bakker, R., Quaife, M., Malhotra, S., Gebreselassie, N., Zignol, M., Hutubessy, R. C. W., Giersing, B., Jit, M., Harris, R. C., Menzies, N. A., & White, R. G. (2023). The impact of alternative delivery strategies for novel tuberculosis vaccines in low-income and middle-income countries: a modelling study. *The Lancet. Global Health*, *11*(4), e546–e555. https://doi.org/10.1016/S2214-109X(23)00045-1

Clark, R. A., Weerasuriya, C. K., Portnoy, A., Mukandavire, C., Quaife, M., Bakker, R., Scarponi, D., Harris, R. C., Rade, K., Mattoo, S. K., Tumu, D., Menzies, N. A., & White, R. G. (2023). New tuberculosis vaccines in India: modelling the potential health and economic impacts of adolescent/adult vaccination with M72/AS01E and BCG-revaccination. *BMC Medicine*, *21*(1), 288. https://doi.org/10.1186/s12916-023-02992-7

Corbett, E. L., Watt, C. J., Walker, N., Maher, D., Williams, B. G., Raviglione, M. C., & Dye, C. (2003). The growing burden of tuberculosis: global trends and interactions with the HIV epidemic. *Archives of Internal Medicine*, *163*(9), 1009–1021. https://doi.org/10.1001/archinte.163.9.1009

Coussens, A. K., Zaidi, S. M. A., Allwood, B. W., Dewan, P. K., Gray, G., Kohli, M., Kredo, T., Marais, B. J., Marks, G. B., Martinez, L.,

Ruhwald, M., Scriba, T. J., Seddon, J. A., Tisile, P., Warner, D. F., Wilkinson, R. J., Esmail, H., Houben, R. M. G. J., & International Consensus for Early TB (ICE-TB) group. (2024). Classification of early tuberculosis states to guide research for improved care and prevention: an international Delphi consensus exercise. *The Lancet. Respiratory Medicine*. https://doi.org/10.1016/S2213-2600(24)00028-6

Crampin, A. C., Glynn, J. R., Traore, H., Yates, M. D., Mwaungulu, L., Mwenebabu, M., Chaguluka, S. D., Floyd, S., Drobniewski, F., & Fine, P. E. M. (2006). Tuberculosis transmission attributable to close contacts and HIV status, Malawi. *Emerging Infectious Diseases*, *12*(5), 729–735. https://doi.org/10.3201/eid1205.050789

Crampin, A. C., Mwaungulu, J. N., Mwaungulu, F. D., Mwafulirwa, D. T., Munthali, K., Floyd, S., Fine, P. E., & Glynn, J. R. (2010). Recurrent TB: relapse or reinfection? The effect of HIV in a general population cohort in Malawi. *AIDS (London, England)*, *24*(3), 417–426. https://doi.org/10.1097/QAD.0b013e32832f51cf

Dye, C., Scheele, S., Dolin, P., Pathania, V., & Raviglione, M. C. (1999). Consensus statement. Global burden of tuberculosis: estimated incidence, prevalence, and mortality by country. WHO Global Surveillance and Monitoring Project. *JAMA: The Journal of the American Medical Association*, *282*(7), 677–686. https://doi.org/10.1001/jama.282.7.677

Emery, J. C., Dodd, P. J., Banu, S., Frascella, B., Garden, F. L., Horton, K. C., Hossain, S., Law, I., van Leth, F., Marks, G. B., Nguyen, H. B., Nguyen, H. V., Onozaki, I., Quelapio, M. I. D., Richards, A. S., Shaikh, N., Tiemersma, E. W., White, R. G., Zaman, K., … Houben, R. M. G. J. (2023). Estimating the contribution of subclinical tuberculosis disease to transmission: An individual patient data analysis from prevalence surveys. *eLife*, *12*. https://doi.org/10.7554/eLife.82469

Espinal, M. A., Kim, S. J., Suarez, P. G., Kam, K. M., Khomenko, A. G., Migliori, G. B., Baéz, J., Kochi, A., Dye, C., & Raviglione, M. C. (2000). Standard short-course chemotherapy for drug-resistant tuberculosis: treatment outcomes in 6 countries. *JAMA: The Journal of the*

American Medical Association, *283*(19), 2537–2545. https://doi.org/10.1001/jama.283.19.2537

Falzon, D., Miller, C., Law, I., Floyd, K., Arinaminpathy, N., Zignol, M., & Kasaeva, T. (2024). Managing tuberculosis before the onset of symptoms. *The Lancet. Respiratory Medicine*, *0*(0). https://doi.org/10.1016/s2213-2600(24)00372-2

Fenner, L., Brinkhof, M. W. G., Keiser, O., Weigel, R., Cornell, M., Moultrie, H., Prozesky, H., Technau, K., Eley, B., Vaz, P., Pascoe, M., Giddy, J., Van Cutsem, G., Wood, R., Egger, M., Davies, M.-A., & International epidemiologic Databases to Evaluate AIDS in Southern Africa. (2010). Early mortality and loss to follow-up in HIV-infected children starting antiretroviral therapy in Southern Africa. *Journal of Acquired Immune Deficiency Syndromes*, *54*(5), 524–532. https://doi.org/10.1097/QAI.0b013e3181e0c4cf

Glynn, J. R., Murray, J., Bester, A., Nelson, G., Shearer, S., & Sonnenberg, P. (2010). High rates of recurrence in HIV-infected and HIV-uninfected patients with tuberculosis. *The Journal of Infectious Diseases*, *201*(5), 704–711. https://doi.org/10.1086/650529

Horton, K. C., Richards, A. S., Emery, J. C., Esmail, H., & Houben, R. M. G. J. (2023). Reevaluating progression and pathways following *Mycobacterium tuberculosis* infection within the spectrum of tuberculosis. *Proceedings of the National Academy of Sciences of the United States of America*, *120*(47), e2221186120. https://doi.org/10.1073/pnas.2221186120

Houben, R. M. G. J., & Dodd, P. J. (2016). The Global Burden of Latent Tuberculosis Infection: A Re-estimation Using Mathematical Modelling. *PLoS Medicine*, *13*(10), e1002152. https://doi.org/10.1371/journal.pmed.1002152

International Institute for Population Sciences. (2022, March). *India: Standard DHS, 2019-21*. The DHS Program. https://dhsprogram.com/methodology/survey/survey-display-541.cfm

Joint United Nations Programme on HIV/AIDS. (2021). *UNAIDS Databook 2021*. Geneva, Switzerland: UNAIDS. https://www.unaids.org/sites/default/files/media_asset/JC3032_AIDS_Data_book_2021_En.pdf

Joint United Nations Programme on HIV/AIDS. (2023). *UNAIDS Databook 2023*. Geneva, Switzerland: UNAIDS. https://www.unaids.org/sites/default/files/media_asset/data-book-2023_en.pdf

Kendall, E. A., Azman, A. S., Cobelens, F. G., & Dowdy, D. W. (2017). MDR-TB treatment as prevention: The projected population-level impact of expanded treatment for multidrug-resistant tuberculosis. *PloS One*, *12*(3), e0172748. https://doi.org/10.1371/journal.pone.0172748

Khan, P. Y., Crampin, A. C., Mzembe, T., Koole, O., Fielding, K. L., Kranzer, K., & Glynn, J. R. (2017). Does antiretroviral treatment increase the infectiousness of smear-positive pulmonary tuberculosis? *The International Journal of Tuberculosis and Lung Disease: The Official Journal of the International Union against Tuberculosis and Lung Disease*, *21*(11), 1147–1154. https://doi.org/10.5588/ijtld.17.0162

Lawn, S. D., Bekker, L.-G., Middelkoop, K., Myer, L., & Wood, R. (2006). Impact of HIV infection on the epidemiology of tuberculosis in a peri-urban community in South Africa: the need for age-specific interventions. *Clinical Infectious Diseases: An Official Publication of the Infectious Diseases Society of America*, *42*(7), 1040–1047. https://doi.org/10.1086/501018

Liu, Y. E., Mabene, Y., Camelo, S., Rueda, Z. V., Pelissari, D. M., Dockhorn Costa Johansen, F., Huaman, M. A., Avalos-Cruz, T., Alarcón, V. A., Ladutke, L. M., Bergman, M., Cohen, T., Goldhaber-Fiebert, J. D., Croda, J., & Andrews, J. R. (2024). Mass incarceration as a driver of the tuberculosis epidemic in Latin America and projected effects of policy alternatives: a mathematical modelling study. *The Lancet. Public Health*, *0*(0). https://doi.org/10.1016/s2468-2667(24)00192-0

Lönnroth, K., Williams, B. G., Cegielski, P., & Dye, C. (2010). A consistent log-linear relationship between tuberculosis incidence and body mass index. *International Journal of Epidemiology*, *39*(1), 149–155. https://doi.org/10.1093/ije/dyp308

Marx, F. M., Dunbar, R., Enarson, D. A., Williams, B. G., Warren, R. M., van der Spuy, G. D., van Helden, P. D., & Beyers, N. (2014). The temporal dynamics of relapse and reinfection tuberculosis after successful treatment: a retrospective cohort study. *Clinical Infectious*

Diseases: An Official Publication of the Infectious Diseases Society of America, *58*(12), 1676–1683. https://doi.org/10.1093/cid/ciu186

Mberi, M. N., Kuonza, L. R., Dube, N. M., Nattey, C., Manda, S., & Summers, R. (2015). Determinants of loss to follow-up in patients on antiretroviral treatment, South Africa, 2004-2012: a cohort study. *BMC Health Services Research*, *15*, 259. https://doi.org/10.1186/s12913-015-0912-2

McQuaid, C. F., Clark, R. A., White, R. G., Bakker, R., Alexander, P., Henry, R., Velayutham, B., Muniyandi, M., Sinha, P., Bhargava, M., Bhargava, A., & Houben, R. M. G. J. (2025). Estimating the epidemiological and economic impact of providing nutritional care for tuberculosis-affected households across India: a modelling study. *The Lancet. Global Health*, *13*(3), e488–e496. https://doi.org/10.1016/S2214-109X(24)00505-9

Mukadi, Y. D., Maher, D., & Harries, A. (2001). Tuberculosis case fatality rates in high HIV prevalence populations in sub-Saharan Africa. *AIDS*, *15*(2), 143–152. https://doi.org/10.1097/00002030-200101260-00002

Naidoo, P., Theron, G., Rangaka, M. X., Chihota, V. N., Vaughan, L., Brey, Z. O., & Pillay, Y. (2017). The south African tuberculosis care cascade: Estimated losses and methodological challenges. *The Journal of Infectious Diseases*, *216*(suppl_7), S702–S713. https://doi.org/10.1093/infdis/jix335

Nyasulu, P. S., Ngasama, E., Tamuzi, J. L., Sigwadhi, L. N., Ozougwu, L. U., Nhandara, R. B. C., Ayele, B. T., Umanah, T., & Ncayiyana, J. (2022). Effect of HIV status and antiretroviral treatment on treatment outcomes of tuberculosis patients in a rural primary healthcare clinic in South Africa. *PloS One*, *17*(10), e0274549. https://doi.org/10.1371/journal.pone.0274549

Owiti, P., Zachariah, R., Bissell, K., Kumar, A. M. V., Diero, L., Carter, E. J., & Gardner, A. (2015). Integrating tuberculosis and HIV services in rural Kenya: uptake and outcomes. *Public Health Action*, *5*(1), 36–44. https://doi.org/10.5588/pha.14.0092

Portnoy, A., Arcand, J.-L., Clark, R. A., Weerasuriya, C. K., Mukandavire, C., Bakker, R., Patouillard, E., Gebreselassie, N., Zignol, M., Jit, M., White, R. G., & Menzies, N. A. (2023). The potential impact of novel tuberculosis vaccine introduction on economic growth in low- and middle-income countries: A modeling study. *PLoS Medicine*, *20*(7), e1004252. https://doi.org/10.1371/journal.pmed.1004252

Portnoy, A., Clark, R. A., Quaife, M., Weerasuriya, C. K., Mukandavire, C., Bakker, R., Deol, A. K., Malhotra, S., Gebreselassie, N., Zignol, M., Sim, S. Y., Hutubessy, R. C. W., Baena, I. G., Nishikiori, N., Jit, M., White, R. G., & Menzies, N. A. (2023). The cost and cost-effectiveness of novel tuberculosis vaccines in low- and middle-income countries: A modeling study. *PLoS Medicine*, *20*(1), e1004155. https://doi.org/10.1371/journal.pmed.1004155

Portnoy, A., Clark, R. A., Weerasuriya, C. K., Mukandavire, C., Quaife, M., Bakker, R., Garcia Baena, I., Gebreselassie, N., Zignol, M., Jit, M., White, R. G., & Menzies, N. A. (2023). The potential impact of novel tuberculosis vaccines on health equity and financial protection in low-income and middle-income countries. *BMJ Global Health*, *8*(7). https://doi.org/10.1136/bmjgh-2023-012466

Prem, K., van Zandvoort, K., Klepac, P., Eggo, R. M., Davies, N. G., Centre for the Mathematical Modelling of Infectious Diseases COVID-19 Working Group, Cook, A. R., & Jit, M. (2021). Projecting contact matrices in 177 geographical regions: An update and comparison with empirical data for the COVID-19 era. *PLoS Computational Biology*, *17*(7), e1009098. https://doi.org/10.1371/journal.pcbi.1009098

Ragonnet, R., Flegg, J. A., Brilleman, S. L., Tiemersma, E. W., Melsew, Y. A., McBryde, E. S., & Trauer, J. M. (2021). Revisiting the natural history of pulmonary tuberculosis: A Bayesian estimation of natural recovery and mortality rates. *Clinical Infectious Diseases: An Official Publication of the Infectious Diseases Society of America*, *73*(1), e88–e96. https://doi.org/10.1093/cid/ciaa602

Richards, A. S., Sossen, B., Emery, J. C., Horton, K. C., Heinsohn, T., Frascella, B., Balzarini, F., Oradini-Alacreu, A., Häcker, B., Odone, A., McCreesh, N., Grant, A. D., Kranzer, K., Cobelens, F., Esmail, H., & Houben, R. M. G. J. (2023). Quantifying progression and regression

across the spectrum of pulmonary tuberculosis: a data synthesis study. *The Lancet. Global Health*, *11*(5), e684–e692. https://doi.org/10.1016/S2214-109X(23)00082-7

Schwalb, A., Emery, J. C., Dale, K. D., Horton, K. C., Ugarte-Gil, C. A., & Houben, R. M. G. J. (2023). Impact of Reversion of Mycobacterium tuberculosis Immunoreactivity Tests on the Estimated Annual Risk of Infection. *American Journal of Epidemiology*. https://doi.org/10.1093/aje/kwad028

Sengayi, M., Dwane, N., Marinda, E., Sipambo, N., Fairlie, L., & Moultrie, H. (2013). Predictors of loss to follow-up among children in the first and second years of antiretroviral treatment in Johannesburg, South Africa. *Global Health Action*, *6*, 19248. https://doi.org/10.3402/gha.v6i0.19248

Sinha, P., Ezhumalai, K., Du, X., Ponnuraja, C., Dauphinais, M. R., Gupte, N., Sarkar, S., Gupta, A., Gaikwad, S., Thangakunam, B., Paradkar, M., Christopher, D. J., Mave, V., Viswanathan, V., Ellner, J. J., Kornfeld, H., Horsburgh, C. R., Jr, Padmapriyadarsini, C., & Gupte, A. (2024). Undernourished household contacts are at increased risk of tuberculosis (TB) disease, but not TB infection-a multicenter prospective cohort analysis. *Clinical Infectious Diseases: An Official Publication of the Infectious Diseases Society of America*, *79*(1), 233–236. https://doi.org/10.1093/cid/ciae149

Sinha, P., Ponnuraja, C., Gupte, N., Prakash Babu, S., Cox, S. R., Sarkar, S., Mave, V., Paradkar, M., Cintron, C., Govindarajan, S., Kinikar, A., Priya, N., Gaikwad, S., Thangakunam, B., Devarajan, A., Dhanasekaran, M., Tornheim, J. A., Gupta, A., Salgame, P., … Hochberg, N. S. (2023). Impact of undernutrition on tuberculosis treatment outcomes in India: A multicenter, prospective, cohort analysis. *Clinical Infectious Diseases: An Official Publication of the Infectious Diseases Society of America*, *76*(8), 1483–1491. https://doi.org/10.1093/cid/ciac915

Sonnenberg, P., Glynn, J. R., Fielding, K., Murray, J., Godfrey-Faussett, P., & Shearer, S. (2005). How soon after infection with HIV does the risk of tuberculosis start to increase? A retrospective cohort study in South African gold miners. *The Journal of Infectious Diseases*, *191*(2), 150–158. https://doi.org/10.1086/426827

South Africa Department of Health. (2014). *National Tuberculosis Management Guidelines 2014*. Department of Health, Republic of South Africa. https://knowledgehub.health.gov.za/system/files/elibdownloads/2023-04/National%252520TB%252520management%252520guidelines%2525202014.pdf

Stover, J., & Glaubius, R. (2024). Methods and Assumptions for Estimating Key HIV Indicators in the UNAIDS Annual Estimates Process. *Journal of Acquired Immune Deficiency Syndromes*, *95*(1S), e5–e12. https://doi.org/10.1097/QAI.0000000000003316

Stover, J., Glaubius, R., Mofenson, L., Dugdale, C. M., Davies, M.-A., Patten, G., & Yiannoutsos, C. (2019). Updates to the Spectrum/AIM model for estimating key HIV indicators at national and subnational levels. *AIDS* , *33 Suppl 3*(Suppl 3), S227–S234. https://doi.org/10.1097/QAD.0000000000002357

Sumner, T., Clark, R. A., Mukandavire, C., Portnoy, A., Weerasuriya, C. K., Bakker, R., Scarponi, D., Hatherill, M., Menzies, N. A., & White, R. G. (2024). Modelling the health and economic impacts ofM72/AS01E vaccination and BCG-revaccination: Estimates for South Africa. *Vaccine*, *42*(6), 1311–1318. https://doi.org/10.1016/j.vaccine.2024.01.072

United Nations. (n.d.). *World Population Prospects - Population Division*. World Population Prospects 2022. Retrieved June 2023, from https://population.un.org/wpp/

Van Cutsem, G., Ford, N., Hildebrand, K., Goemaere, E., Mathee, S., Abrahams, M., Coetzee, D., & Boulle, A. (2011). Correcting for mortality among patients lost to follow up on antiretroviral therapy in South Africa: a cohort analysis. *PloS One*, *6*(2), e14684.

https://doi.org/10.1371/journal.pone.0014684

Verver, S., Warren, R. M., Beyers, N., Richardson, M., van der Spuy, G. D., Borgdorff, M. W., Enarson, D. A., Behr, M. A., & van Helden, P. D. (2005). Rate of reinfection tuberculosis after successful treatment is higher than rate of new tuberculosis. *American Journal of Respiratory and Critical Care Medicine*, *171*(12), 1430–1435. https://doi.org/10.1164/rccm.200409-1200OC

Winter, J. R., Smith, C. J., Davidson, J. A., Lalor, M. K., Delpech, V., Abubakar, I., & Stagg, H. R. (2020). The impact of HIV infection on tuberculosis transmission in a country with low tuberculosis incidence: a national retrospective study using molecular epidemiology. *BMC Medicine*, *18*(1), 385. https://doi.org/10.1186/s12916-020-01849-7

World Health Organization. (2022a). *WHO Consolidated Guidelines on Tuberculosis. Module 4: Treatment - Drug-resistant tuberculosis treatment*. Geneva, Switzerland: WHO. https://iris.who.int/bitstream/handle/10665/365308/9789240063129-eng.pdf?sequence=1

World Health Organization. (2022b). *WHO Consolidated Guidelines on Tuberculosis. Module 4: Treatment - Drug-susceptible tuberculosis treatment*. Geneva, Switzerland: WHO.

World Health Organization. (2023). *Global Tuberculosis Report 2023*. Geneva, Switzerland: WHO. https://iris.who.int/bitstream/handle/10665/373828/9789240083851-eng.pdf?sequence=1

World Health Organization. (2025). *Global Health Observatory*. WHO. https://www.who.int/data/gho

Yang, N., He, J., Li, J., Zhong, Y., Song, Y., & Chen, C. (2023). Predictors of death among TB/HIV co-infected patients on tuberculosis treatment in Sichuan, China: A retrospective cohort study. *Medicine*, *102*(5), e32811. https://doi.org/10.1097/MD.0000000000032811

Zifodya, J. S., Kreniske, J. S., Schiller, I., Kohli, M., Dendukuri, N., Schumacher, S. G., Ochodo, E. A., Haraka, F., Zwerling, A. A., Pai, M., Steingart, K. R., & Horne, D. J. (2021). Xpert Ultra versus Xpert MTB/RIF for pulmonary tuberculosis and rifampicin resistance in adults

with presumptive pulmonary tuberculosis. *Cochrane Database of Systematic Reviews* , *2*, CD009593.

https://doi.org/10.1002/14651858.CD009593.pub5

S2 INTERVENTIONS

This document describes the TB interventions modelled, first describing the principles and then each intervention in turn, followed by the scale-up scenarios.

1. PRINCIPLES

When deciding what to model, we are looking for the following characteristics for each intervention category:

- *One intervention per category:* model a single variation of the same intervention (e.g., one algorithm for community screening)
    - Rationale: With three countries, multiple interventions with multiple outcomes, the number of results will be quite large already. Also, the aim of this exercise is to compare across intervention categories, not within.
- *Similar level of novelty:* for each intervention category that is at the forefront of current development, but ideally has an empirical estimate of potential epidemiological impact.
    - Rationale: We are looking to have a fair comparison across the interventions. If one intervention is more aspirational rather than supported by empirical evidence of epidemiological impact, this introduces an imbalance.
- *Same interventions in each country: the* format of the intervention and empirical impact are applied equally across countries.
    - Rationale: the reason for differences in outcomes (epi, CEA, budget) is driven by differences in the epidemiological baseline situation between Brazil, India and South Africa. An additional reason for between-country differences is baseline levels of overlapping interventions, such as treatment for DS-TB and DR-TB. This will be explored as well.
- *Same roll-out and scale up:* for all interventions, we will start scale-up in 2025, and reach 80% coverage of the target population by 2030 (i.e., a 5-year scale-up period).
    - Rationale: Alignment in roll-out and scale-up means that comparisons across the outcomes are more likely to be comparable.

2. INTERVENTIONS

We considered the following list of intervention categories, across four domains:

1. **Prevention**: TB vaccination, TB Preventative Treatment
2. **Screening and diagnosis**: Community screening, enhanced diagnostic in clinics, drug susceptibility testing for all
3. **Treatment**: Shorter DS-TB regimen, shorter DR-TB regimen
4. **Structural drivers**: Mass screening in prisons in Brazil, nutritional support for HHC of individuals starting TB treatment in India.

Note: We will also define a ‘business-as-usual’ (BAU) scenario for each country to provide a baseline to compare against.

2.1 BUSINESS-AS-USUAL

The following tables present the BAU scenario for each country. These scenarios share some assumptions to enable clearer comparisons between BAU and intervention scenarios, and facilitate costing:

- All TB treatment (DS- and DR-) is at home with only monthly visits to the clinic: no inpatient stays, no DOTS.
- Adverse events caused by TB treatment are not taken into account (for costs or utility).
- BCG vaccination occurs at current rates and remains unchanged in all scenarios, and there are no additional vaccinations against TB other than in the Vaccination intervention.
- It is assumed that there is 0% TPT for child contacts at present, only TPT for people living with HIV in South Africa (in reality, there is also some TPT for child contacts of DS-TB).
- It is assumed that there is 0% nutrition support provided for people with TB and low BMI or their families.
- It is assumed that there is 0% routine community screening.
- Costs do not vary over time.
- Comorbidities (other than HIV in South Africa and malnutrition in India) are not considered in the model.
- Wider or longer-term health service expenditure for the subjects is not considered.

2.1.1 BUSINESS-AS-USUAL: BRAZIL

The following table describes the intervention-specific changes and costs for BAU in Brazil.

**Aspect**	**Details**
**Intervention summary**	
Intervention number, abbreviation, name	1a. BAU (BRA) - Business-as-usual in Brazil

<table>
<tr><td>Summary description</td><td colspan="4">Current diagnosis (clinical examination, some CXR and Xpert) and standard TB treatments in Brazil</td></tr>
<tr><td>Population affected</td><td colspan="4">All (no changes for any group)</td></tr>
<tr><td colspan="5">Changes to the model</td></tr>
<tr><td>What is the effectiveness of the intervention?</td><td colspan="4">N/A - No intervention</td></tr>
<tr><td>Which compartments are affected?</td><td colspan="4">N/A - No intervention</td></tr>
<tr><td>Which transitions are affected?</td><td colspan="4">N/A - No intervention</td></tr>
<tr><td>Which parameters are changed? From what value to what value?</td><td colspan="4">N/A - No intervention</td></tr>
<tr><td>Any further details of changes</td><td colspan="4">N/A - No intervention</td></tr>
<tr><td colspan="5">Costs</td></tr>
<tr><td rowspan="3">What is being costed, and where are the costs applied?</td><td>Activity</td><td>Resource use</td><td>Population</td><td>Model output</td></tr>
<tr><td>Standard TB assessment</td><td>1x appointment at a clinic
35% of patients x CXR
50% of patients x GeneXpert Ultra</td><td>Those entering the diagnostic pathway</td><td>n_assess_y[n]</td></tr>
<tr><td>Standard DS-TB treatment</td><td>7x monthly appointments at a clinic
6 months of H+R+Z+E</td><td>Those entering treatment for DS-TB</td><td>n_treat_DS_y[n]</td></tr>
</table>

	Standard DR-TB treatment	**19x** monthly appointments at a clinic<br>**18 months** of R+Lfx+Z+E	Those entering treatment for DR-TB	n_treat_DR_y[n]
Do costs vary by age?	No			
**Implementation / Scale-up**				
Duration of scale-up	N/A - No intervention			
What happens after implementation?	N/A - No intervention			
How is this implemented in the model?	N/A - No intervention			
How is this implemented in the costings?	N/A - No intervention			
**Assumptions**				
Any other assumptions to note?	It is assumed that baseline diagnosis and treatment in prison are the same as in the community<br>The proportion of patients who received a CXR and GeneXpert were estimated using the Notifiable Diseases Information System (SINAN), a health information system that consolidates data and monitors TB care and prevention across Brazil (Governo Brasileiro, n.d.).<br>- CXR: The precise number of patients receiving CXR is not known. The proportion of all TB patients that were clinically confirmed (2022) was 31% (28,326/91,808). 35% was chosen as a slightly higher value to reflect the fact that some other individuals will have had inconclusive CXR<br>- Xpert: % of patients that received Xpert (year 2022) over all cases: 50% (46,252/91,808)			

2.1.2 BUSINESS-AS-USUAL: INDIA

The following table describes the intervention-specific changes and costs for BAU in India.

**Aspect**	**Details**
**Intervention summary**	
Intervention number, abbreviation, name	1b. BAU (IND) - Business-as-usual in India
Summary description	Current diagnosis (clinical examination, some CXR and Xpert) and standard TB treatments in India
Population affected	ALL (no changes for any group)
**Changes to the model**	
What is the effectiveness of the intervention?	N/A - No intervention
Which compartments are affected?	N/A - No intervention
Which transitions are affected?	N/A - No intervention
Which parameters are changed? From what value to what value?	N/A - No intervention
Any further details of changes	N/A - No intervention
**Costs**	

What is being costed, and where are the costs applied?	**Activity**	**Resource use**	**Population**	**Model output**
	Standard TB assessment	**1x** appointment at a clinic<br>**10%** of patients x CXR<br>**21%** of patients x GeneXpert Ultra	Those entering the diagnostic pathway	n_assess_y[n]
	Standard DS-TB treatment	**7x** monthly appointments at a clinic<br>**6 months** of H+R+Z+E	Those entering treatment for DS-TB	n_treat_DS_y[n]
	Standard DR-TB treatment	**10x** monthly appointments at a clinic<br>**9 months** of Lfx+Cfz+Z+E+Hh+Eto+Bdq	Those entering treatment for DR-TB	n_treat_DR_y[n]
Do costs vary by age?	No			
**Implementation / Scale-up**				
Duration of scale-up	N/A - No intervention			
What happens after implementation?	N/A - No intervention			
How is this implemented in the model?	N/A - No intervention			
How is this implemented in the costings?	N/A - No intervention			
**Assumptions**				
Any other assumptions to note?	The proportion of individuals receiving CXR was considered as the lower quartile of CXR tests used			

	among the number of individuals diagnosed in 2022 (Central Tuberculosis Division, 2023)<br>The proportion of individuals receiving Xpert was considered as the % of NAATs among those tested in an examination for presumptive TB in 2023 (Central Tuberculosis Division, 2024).

2.1.3 BUSINESS-AS-USUAL: SOUTH AFRICA

The following table describes the intervention-specific changes and costs for BAU in South Africa.

Aspect	Details
**Intervention summary**	
Intervention number, abbreviation, name	1c. BAU (ZAF) - Business-as-usual in South Africa
Summary description	Current diagnosis (clinical examination, some CXR and Xpert) and standard TB treatments in South Africa
Population affected	All (no changes for any group)
**Changes to the model**	
What is the effectiveness of the intervention?	N/A - No intervention
Which compartments are affected?	N/A - No intervention
Which transitions are affected?	N/A - No intervention
Which parameters are	N/A - No intervention

changed? From what value to what value?				
Any further details of changes	N/A - No intervention			
**Costs**				
What is being costed, and where are the costs applied?	**Activity**	**Resource use**	**Population**	**Model output**
	Standard TB assessment	**1x** appointment at a clinic<br>**3.12%** of patients x CXR<br>**61%** of patients x GeneXpert Ultra	Those entering the diagnostic pathway	n_assess_y[n]
	Standard DS-TB treatment	**7x** monthly appointments at a clinic<br>**6 months** of H+R+Z+E	Those entering treatment for DS-TB	n_treat_DS_y[n]
	Standard DR-TB treatment	**10x** monthly appointments at a clinic<br>**9 months** of Lfx+Lzd+Cfz+Z+E+Hh+Bdq	Those entering treatment for DR-TB	n_treat_DR_y[n]
Do costs vary by age?	No			
**Implementation / Scale-up**				
Duration of scale-up	N/A - No intervention			
What happens after implementation?	N/A - No intervention			
How is this implemented in the model?	N/A - No intervention			

How is this implemented in the costings?	N/A - No intervention
**Assumptions**	
Any other assumptions to note?	It is assumed that people living with HIV receive TPT for DS-TB in all scenarios (and so this is not modelled). TPT for DR-TB in PLHIV is not investigated in this model.<br>Proportion of individuals receiving Xpert was calculated from WHO data (World Health Organization, 2024a), which suggested that 61% of the diagnoses were bacteriologically confirmed. Data from South Africa suggested that more than 90% of the tests conducted in people suspected of TB were Xpert (National Department of Health, Republic of South Africa, 2023). Therefore, it was assumed that everyone with a bacteriological diagnosis in South Africa would have received an Xpert test. Proportion of individuals receiving a CXR was calculated as the number of CXRs performed divided by the number of Xpert tests performed (National Department of Health, Republic of South Africa, 2023)

2.2 VACCINATION

The following table describes the specific changes and costs for TB vaccination across all three settings.

Aspect	Details
**Intervention summary**	
Intervention number, abbreviation, name	2. VAX - TB vaccination
Summary description	Annually vaccinate current unvaccinated 15-19-year-olds with 2 doses of a new M72-like TB vaccine. Catch-up campaign for unvaccinated 20+ year-olds over five years, repeated after 10 years
Population affected	All 15+ year-olds
**Changes to the model**	
What is the effectiveness of the intervention?	50% reduction of progression to infectious disease (Tait et al., 2019)
Which compartments are affected?	Uninfected (S, C), infected (I_s_N, I_r_N, I_s_P, I_r_P), non-infectious TB (Dn_s_N, Dn_r_N, Dn_s_P, Dn_r_P), and recently treated (Tr_s_P, Tr_r_P) compartments
Which transitions are affected?	Progression to infectious TB
Which parameters are changed? From what value to what value?	*infsub, nonsub* and *relsub* were reduced by 50% in individuals who received vaccination
Any further details of changes	Loss of vaccine efficacy was assumed to was assumed to wane exponentially with an average duration of protection of 10 years. Individuals who were vaccinated and progressed to infectious TB anyway were assumed to immediately lose that protection from vaccines (rate = 10).

**Costs**				
What is being costed, and where are the costs applied?	**Activity**	**Resource use**	**Population**	**Model output**
	Standard TB assessment	As BAU (varies by country)	Those entering the diagnostic pathway	n_assess_y[n]
	Standard DS-TB treatment	As BAU (6 months of H+R+Z+E)	Those entering treatment for DS-TB	n_treat_DS_y[n]
	Standard DR-TB treatment	As BAU (varies by country)	Those entering treatment for DR-TB	n_treat_DR_y[n]
	Routine vaccination	Annually:<br>**2x** vaccine dose (including 5% wastage)<br>**2x** vaccination (including supply costs)	15-19-year-olds	n_vaccinated_y[n] (also includes below)
	Catch-up vaccination	At scale-up and repeated after 10 years:<br>**2x** vaccine dose (including 5% wastage)<br>**2x** vaccination (including supply costs)	20+ year-olds	n_vaccinated_y[n] (also includes above)
Do costs vary by age?	No			
**Implementation / Scale-up**				
Duration of scale-up	5 years			
What happens after implementation?	2030 - 2050: 80% of 15-19-year-olds vaccinated annually.<br>2039: one-year (re)vaccination campaign for all 15+ year olds.			

How is this implemented in the model?	Over each of the first 5 years, 16% of those of ages eligible for either initial or catch-up vaccination (15+ years) receive vaccination - and so by the end of 2029 the equivalent of 80% of 15+ year olds have been vaccinated (noting that this will not be exactly 80% as the number of people 15+ will change with demographics as some will die or turn 15 during this period). This means that approximately the same number of vaccinations will be delivered in each of the first 5 years, not a gradual increase as in other interventions.<br>From 2030 - 2050, 80% of unvaccinated 15-19-year-olds are vaccinated each year.<br>In 2039, 80% of all 20+ year-olds are vaccinated
How is this implemented in the costings?	The model output n_vaccinated_y[n] counts the exact number vaccinated each year, regardless of age and incorporates scale-up, so the cost of vaccination is just multiplied by the number vaccinated.
**Assumptions**	
Any other assumptions to note?	Costs of introducing the vaccination programme in the first year ($2.40 per person, estimated by GAVI) are currently excluded from the analysis, to keep consistent with other interventions for which we do not have introduction costs.

2.3 TB PREVENTIVE TREATMENT

The following table describes the specific changes and costs for TB preventive treatment across all three settings.

Aspect	Details
**Intervention summary**	
Intervention number, abbreviation, name	3. TPT - TB preventive treatment
Summary description	Screen household members of individuals with asymptomatic or symptomatic TB diagnosed with TB (considered as bacteriologically diagnosed), and treat if diagnosed with TB. Give TPT to all children (<15 years old) without TB disease in households with an adult with TB (DS-TPT: 3HP, if index patient has DS-TB; DR-TPT: Lfx, if index patient has DR-TB).
Population affected	People newly diagnosed with and starting treatment for TB (both DS-TB and DR-TB). All household contacts of newly diagnosed asymptomatic or symptomatic TB on TB treatment. Household members of those newly diagnosed who are a) children <15 years old without TB disease, and b) children and adults with infectious TB.
**Changes to the model**	
What is the effectiveness of the intervention?	Reduction in progression among recently exposed individuals (DS-TPT: 64%; DR-TPT: 62%) (World Health Organization, 2024b)
Which compartments are affected?	Eligible for TPT: Children HHCs in susceptible (*S*), cleared (*C*), infection (*I*) and treated more than 1 year ago (*Tc*) compartments. Eligible for TB treatment: Adult or children HHCs with bacteriologically confirmed TB (*Ds* and *Dc*).
Which transitions are affected?	TB progression and the rate at which the household contacts start treatment are affected
Which parameters are changed? From what value to what value?	For TPT in children with no TB: TPT reduces the chances of progressing to TB by multiplying 0.36 in DS-TB TPT and 0.4 in DR-TB TPT to the following progression parameters: *infnon*, *infsub*, *nonsub*, and

	subclin. Children receiving TPT can not receive TB treatment.<br><br>For TB treatment in adults and children with infectious TB: We assumed a proportion of 3.4% with prevalent TB (Fox et al., 2013), which bypasses the usual treatment cascade and starts treatment through the following equation:<br>screening_coverage * HH_size_children/adults * proportion_prevalent_TB<br>N.B. The HHC starts DS-TB or DR-TB treatment based on their contact's treatment regimen.			
Any further details of changes	We used the following equations to represent the movement from no intervention to TPT:<br><br>- For *I*:<br>screening_coverage * HH_size_children * (1 - proportion_prevalent_TB) * proportion_completion_TPT * proportion_IGRApositive<br><br>- For *S*, *C*, *R* and *Tc*:<br>screening_coverage * HH_size_children * (1 - proportion_prevalent_TB) * proportion_completion_TPT * (1 - proportion_IGRApositive)<br><br>N.B. The *proportion_IGRApositive* was 35% (Martinez et al., 2020).			
**Costs**				
What is being costed, and where are the costs applied?	**Activity**	**Resource use**	**Population**	**Model output**
	Standard TB assessment	As BAU (varies by country)	Those entering the diagnostic pathway	n_assess_y[n]
	Standard DS-TB treatment	As BAU (6 months of H+R+Z+E)	Those entering treatment for DS-TB	n_treat_DS_y[n]
	Standard DR-TB treatment	As BAU (varies by country)	Those entering treatment for DR-TB	n_treat_DR_y[n]

	Household contact assessment	**1x** appointment at a clinic<br>**1x** GeneXpert MTB/RIF Ultra	All household contacts of people newly diagnosed with DS-TB or DR-TB	n_HHC_DS_y[n] + n_HHC_DR_y[n]
	TPT for DS-TB	**4x** monthly appointments at a clinic<br>**3 months** DS-TPT drugs (3HP)	Household contacts receiving TPT for DS-TB	n_HHC_DSTPT_y[n]
	TPT for DR-TB	**7x** monthly appointments at a clinic<br>**6 months** DR-TPT drugs (Lfx)	Household contacts receiving TPT for DR-TB	n_HHC_DRTPT_y[n]
Do costs vary by age?	No. TPT costs are based on a 25kg child (only children receive TPT).			
**Implementation / Scale-up**				
Duration of scale-up	5 years			
What happens after implementation?	Continues at 80% for the remaining 20 years			
How is this implemented in the model?	Contacts being assessed for TB and contacts receiving TPT: Straight line continuous increase from 0% at the start of 2025 to 80% at the end of 2029, then maintained at 80% (noting caveats under 'Population affected' above)			
How is this implemented in the costings?	Cost of assessment applied to 16% of total eligible populations (n_HHC_DS/DR_y) at the end of Y1, 32% at the end of Y2, 48% at the end of Y3, 64% at the end of Y4, and 80% in Y5 and in all remaining years. n_HHC_DS/DRTPT_y outputs incorporate scale up (i.e. these outputs report the exact number of people receiving TPT each year), so the cost of TPT is just directly applied per person without further adjustment.			

Assumptions	
Any other assumptions to note?	Adverse events caused by taking TPT are not included in the results, either in terms of loss of utility/increased disability or in terms of the cost of additional medical care.

2.4 NUTRITION

The following table describes the specific changes and costs for nutrition in India.

Aspect	Details
**Intervention summary**	
Intervention number, abbreviation, name	4. NTN - Nutrition
Summary description	Provide nutritional support for 6 months to all those diagnosed with TB and their household members.
Population affected	All individuals starting TB treatment and their households.
**Changes to the model**	
What is the effectiveness of the intervention?	For individuals with TB: 33% reduced risk of on-treatment mortality (McQuaid et al., 2025).<br>For household contacts: 39% reduced progression to disease (Bhargava et al., 2023)
Which compartments are affected?	For the reduced risk of on-treatment mortality: DS and DR treatment for symptomatic TB (*Dc*).<br>For the reduced progression to disease: Infected (*I*) compartments.
Which transitions are affected?	Reduced risk of treatment mortality: on-treatment mortality parameters (*imort_stDc* and *imort_rtDc*)<br>Reduced progression to disease: progression from infected (*I*) to asymptomatic TB (*Ds*) (*nonsub*)
Which parameters are changed? From what value to what value?	Intervention-specific parameter T_mort = 0.67 represents the reduced risk of on-treatment mortality<br>The reduced risk among household contacts is implemented using the following intervention-specific parameters (McQuaid et al., 2025):<br>- Number of HHC = 3.4 (household size in IND - index case)<br>- Proportion of HHC who would develop TB = 3419/100,000<br>- Relative risk of progression = 0.39

Any further details of changes	The reduction in on-treatment mortality is implemented as a weighted average of the BAU and intervention risks (weighted by the time-varying coverage of the intervention)<br><br>The reduction in progression is implemented using the “trigger” functionality. This uses the number of individuals starting treatment to reduce the progression rate for a proportion of the population (based on the parameters described above).			
**Costs**				
What is being costed, and where are the costs applied?	**Activity**	**Resource use**	**Population**	**Model output**
	Standard TB assessment	As BAU (India):<br>**1x** appointment at a clinic<br>**10%** of patients x CXR<br>**21%** of patients x Xpert Ultra	Those entering the diagnostic pathway	n_assess_y[n]
	Standard DS-TB treatment	As BAU (India): **7x** monthly appointments at a clinic<br>**6 months** of H+R+Z+E	Those entering treatment for DS-TB	n_treat_DS_y[n]
	Standard DR-TB treatment	As BAU (India): **10x** monthly appointments at a clinic<br>**9 months** of Lfx+Cfz+Z+E+Hh+Eto+Bdq	Those entering treatment for DR-TB	n_treat_DR_y[n]
	Nutritional supplementation for TB patients	**6x** monthly food baskets, including delivery<br>**0x** additional appointments	People diagnosed with TB receiving nutritional support	n_nutrition_index_y[n]
	Nutritional supplementation for household contacts	**6x** monthly food baskets, including delivery [N.B. different from baskets for TB patients]<br>**0x** appointments	Household contacts of people diagnosed with TB receiving nutritional support	n_nutrition_HHC_y[n]

Do costs vary by age?	No
**Implementation / Scale-up**	
Duration of scale-up	5 years
What happens after implementation?	Continues at 80% for the remaining years
How is this implemented in the model?	Straight line continuous increase from 0% at the start of 2025 to 80% at the end of 2029, then maintained at 80% (for both people with TB and their household contacts)
How is this implemented in the costings?	Model outputs (n_nutrition_index_y and n_nutrition_HHC_y) count the actual number of people receiving the intervention in the model and so incorporate the scale-up, so the unit costs are applied to the number of people in each of the two categories.
**Assumptions**	
Any other assumptions to note?	Cost of delivery includes transportation, and also salaries for community health workers who check that the nutrition has reached the intended recipients.

2.5 COMMUNITY-WIDE SCREENING

The following table describes the specific changes and costs for community-wide screening across all three settings.

Aspect	Details
**Intervention summary**	
Intervention number, abbreviation, name	5. SCR - Community screening
Summary description	Annually for 5 years, screen the adult population (≥15 years old) for TB using mobile CXR for all and a confirmatory Xpert MTB/RIF Ultra for those with abnormal findings on the CXR, then start TB treatment on all those diagnosed.
Population affected	All, including incarcerated individuals in Brazil.
**Changes to the model**	
What is the effectiveness of the intervention?	It is expected to mirror the impact observed in the ACT3 trial (Marks et al., 2019)
Which compartments are affected?	Adults in all states except those on treatment.
Which transitions are affected?	The diagnostic pathway was added as a separate screening process from the usual one.
Which parameters are changed? From what value to what value?	Access to community screening was defined as *iaccess*, and scaled up to 80% of the adult population being screened.<br><br>Test positivity (*itestpos*) was modified to represent CXR screening with confirmatory Xpert Ultra (Schwalb et al., 2025)**:**

<table>
<tr><td></td><td colspan="4">- S, C, and I = 0.0005 (CXR = 0.085, Xpert Ultra = 0.006)
- R and Tc = 0.0201 (CXR = 0.503, Xpert Ultra = 0.040)
- Dn = 0.0298 (CXR = 0.677, Xpert Ultra = 0.044)
- Ds = 0.5247 (CXR = 0.677, Xpert Ultra = 0.775)
- Dc = 0.7725 (CXR = 0.850, Xpert Ultra = 0.909)

N.B.: This test positivity is assumed to be the same between PLHIV and HIV negative individuals.
- Xpert sensitivity and specificity showed confidence intervals that overlapped (Zifodya et al., 2021)
- CXR does not seem to differ by HIV status (Fehr et al., 2021)

Given that Xpert can also be used to test for drug-susceptibility and everybody that tests positive for CXR is tested with Xpert, the probability of receiving a DST (idst_n and idst_p) was set as 1.

Mortality on treatment (imort_st and imort_rt) was null to represent the improvement in outcome due to early diagnosis, slightly increasing as a result of the success and failure.

We assumed that individuals would be more likely to accept and receive treatment (iaccept) after being diagnosed with TB with both CXR and Xpert, thus the value was set to 1.</td></tr>
<tr><td>Any further details of changes</td><td colspan="4">None</td></tr>
<tr><td colspan="5">Costs</td></tr>
<tr><td rowspan="3">What is being costed, and where are the costs applied?</td><td>Activity</td><td>Resource use</td><td>Population</td><td>Model output</td></tr>
<tr><td>Standard TB assessment</td><td>As BAU (varies by country)</td><td>Those entering the diagnostic pathway</td><td>n_assess_y[n]</td></tr>
<tr><td>Standard DS-TB</td><td>As BAU (6 months of</td><td>Those entering</td><td>n_treat_DS_y[n]</td></tr>
</table>

	treatment	H+R+Z+E)	treatment for DS-TB	
	Standard DR-TB treatment	As BAU (varies by country)	Those entering treatment for DR-TB	n_treat_DR_y[n]
	Community screening - CXR	**1x** CXR in a mobile clinic	People receiving screening	n_screen_y[n]
	Community screening - Xpert	**1x** GeneXpert MTB/RIF Ultra	Those with abnormal findings on CXR	n_screen_CXRpositive_y[n]
Do costs vary by age?	No			
**Implementation / Scale-up**				
Duration of scale-up	5 years			
What happens after implementation?	Remains at 80% for 5 years, then 0% for the remaining 15 years			
How is this implemented in the model?	Straight line continuous increase from 0% at the start of 2025 to 80% at the end of 2029, then maintained at 80% until the end of 2034			
How is this implemented in the costings?	Model output (n_Test_count_y) counts the exact number of people screened each year in the model, and n_screen_CXRpositive_y counts the exact number of people getting follow-up Xpert, so unit costs are just applied directly to these counts.			
**Assumptions**				
Any other assumptions to note?	The cost analysis for this intervention is being run twice for two separate scenarios:<br>‘high-cost community screening’: based on costs consistent with data from ZAF			

	‘low-cost community screening: based on costs consistent with data from BRA/IND

2.6 IMPROVED DIAGNOSIS

The following table describes the specific changes and costs for improved diagnosis across all three settings.

**Aspect**	**Details**
**Intervention summary**	
Intervention number, abbreviation, name	6. DGN - Improved diagnosis
Summary description	Everyone assessed for TB will receive GeneXpert MTB/RIF Ultra instead of the previous standard care
Population affected	People entering the diagnostic pathway
**Changes to the model**	
What is the effectiveness of the intervention?	Increased sensitivity for bacteriologically confirmed TB, increased specificity for non-disease states, and increased DST coverage (Zifodya et al., 2021)
Which compartments are affected?	All individuals in states not on treatment
Which transitions are affected?	Diagnostic pathway in the clinic (in line with the usual care pathway)
Which parameters are changed? From what value to what value?	Test positivity (*testpos*) is changed to reflect 80% receiving Xpert Ultra and 20% utilising the previous diagnostic standard practice. New *testpos* values varied by TB state:<br>- *S*, *C,* and *I* = 0.0140<br>- *R* = 0.047<br>- *Tc* = 0.1345<br>- *Dn* = 0.08<br>- *Ds* = 0.525

	- *Dc* = 0.8189<br><br>Previous values of *testpos* varied by country and TB state.<br>- *S*, *C* and *I* (BRA // IND // ZAF) = 0.0208 // 0.0294 // 0.0251<br>- *R* (BRA // IND // ZAF) = 0.0635 // 0.0821 // 0.067<br>- *Tc* (BRA // IND // ZAF) = 0.1983 // 0.2778 // 0.2366<br>- *Dn* (BRA // IND // ZAF) = 0.0909 // 0.1307 // 0.0882<br>- *Ds* (BRA // IND // ZAF) = 0.4420 // 0.3549 // 0.4481<br>- *Dc* (BRA // IND // ZAF) = 0.7901 // 7599 // 0.7922<br><br>The probability of getting a DST (*dst_n* and *dst_p*) changed to 0.8, as the proportion receiving Xpert Ultra also increased. The previous value of *dst_n* and *dst_p* varied by country and treatment history:<br>- *dst_n* (BRA // IND // ZAF) = 0.318 // 0.06 // 0.12<br>- *dst_p* (BRA // IND // ZAF) = 0.341 // 0.26 // 0.28			
Any further details of changes	Test positivity values for *Dn* were changed to 0.08 for all countries to ensure prevalence did not increase in ZAF due to their large proportion of individuals in *Dn* (the previously calculated value was 0.053).			
**Costs**				
What is being costed, and where are the costs applied?	**Activity**	**Resource use**	**Population**	**Model output**
	Improved TB assessment	**1x** appointment at a clinic<br>**1x** GeneXpert MTB/RIF Ultra<br>**0x** CXR	Those entering the diagnostic pathway	n_assess_y[n]<br>[subject to scale-up]
	Standard TB assessment	As BAU (see tables 2.1.1-2.1.3):<br>**1x** appointment at a clinic<br>**%** CXR - varies by country<br>**%** Xpert Ultra - varies by country	Those entering the diagnostic pathway	n_assess_y[n]<br>[subject to scale-down]

	Standard DS-TB treatment	As BAU (6 months of H+R+Z+E)	Those entering treatment for DS-TB	n_treat_DS_y[n]
	Standard DR-TB treatment	As BAU (varies by country)	Those entering treatment for DR-TB	n_treat_DR_y[n]
Do costs vary by age?	No			
**Implementation / Scale-up**				
Duration of scale-up	5 years			
What happens after implementation?	Remains at 80% for the remaining 20 years			
How is this implemented in the model?	Straight line continuous increase from 0% at the start of 2025 to 80% at the end of 2029, then maintained at 80% for the remaining 20 years. Conversely, those not receiving the improved intervention continue to receive the current standard assessment (100% at start, declining to 20% by 5 years)			
How is this implemented in the costings?	Cost of improved assessment applied to 16% of total eligible population at the end of Y1, 32% at the end of Y2, 48% at the end of Y3, 64% at the end of Y4, and 80% at the end of Y5 onwards, with the cost of the current standard assessment applied to the rest of the eligible population in each year.			
**Assumptions**				
Any other assumptions to note?	None.			

2.7 DRUG SUSCEPTIBILITY TESTING

The following table describes the specific changes and costs for drug susceptibility testing across all three settings.

**Aspect**	**Details**
**Intervention summary**	
Intervention number, abbreviation, name	7. DST - Drug susceptibility testing
Summary description	Everyone diagnosed with TB will be given a GeneXpert MTB/RIF Ultra test to test for rifampicin-resistance, except those who have already had an Xpert test through BAU diagnosis
Population affected	Those newly diagnosed with TB
**Changes to the model**	
What is the effectiveness of the intervention?	Increased initiation of drug-resistant treatment (Zifodya et al., 2021)
Which compartments are affected?	All individuals in states not on treatment
Which transitions are affected?	Usual diagnostic pathway
Which parameters are changed? From what value to what value?	The probability of getting a DST (*dst_n* and *dst_p*) was increased to 0.8 from 2025 (with a five-year scale-up) to 2030. The previous values of *dst_n* and *dst_p* varied by country and treatment history:<br>- *dst_n* (BRA // IND // ZAF) = 0.318 // 0.06 // 0.12<br>- *dst_p* (BRA // IND // ZAF) = 0.341 // 0.26 // 0.28

Any further details of changes	Mortality on DR-TB treatment decreased over time (2025 - 2030) to ensure that a higher number of people accessing DR treatment does not lead to an increased number of deaths. The new values varied by country and year:<br>- BRA: *mort_rt* (2026, 2027, 2028, 2029, 2030) = 0.192, 0.186, 0.181, 0.178, 0.175<br>- IND: *mort_rt* (2026, 2027, 2028, 2029, 2030) = 0.230, 0.200, 0.185, 0.170, 0.160<br>- ZAF: *mort_rt* (2026, 2027, 2028, 2029, 2030) = 0.253, 0.234, 0.220, 0.210, 0.200			
**Costs**				
What is being costed, and where are the costs applied?	**Activity**	**Resource use**	**Population**	**Model output**
	Standard TB assessment	As BAU:<br>**1x** appointment at a clinic<br>**%** CXR - varies by country<br>**%** Xpert Ultra -varies by country	Those entering the diagnostic pathway	n_assess_y[n]
	Drug susceptibility testing	**1x** Xpert MTB/RIF Ultra	Those diagnosed with TB and not already tested using Xpert	n_diag_noXpert_y[n] [subject to scale-up]
	Standard DS-TB treatment	As BAU (6 months of H+R+Z+E)	Those entering treatment for DS-TB	n_treat_DS_y[n]
	Standard DR-TB treatment	As BAU (varies by country)	Those entering treatment for DR-TB	n_treat_DR_y[n]
Do costs vary by age?	No			
**Implementation / Scale-up**				

Duration of scale-up	5 years
What happens after implementation?	Remains at 80% for the remaining 20 years
How is this implemented in the model?	Straight line continuous increase from 0% at the start of 2025 to 80% at the end of 2029, then maintained at 80% for the remaining 20 years. The number diagnosed with TB and assumed not to have already been tested with Xpert is calculated outside the model.
How is this implemented in the costings?	Cost of additional DST applied to 16% of those diagnosed with TB and estimated not to have had an Xpert during BAU diagnostic process at the end of Y1, 32% at the end of Y2, 48% at the end of Y3, 64% at the end of Y4, and 80% at the end of Y5 onwards.
**Assumptions**	
Any other assumptions to note?	Note that *n_diag_noXpert_y* is estimated through a simplistic approach, using the proportion of patients evaluated with GeneXpert under the standard TB assessment (BRA = 50%, IND = 21%, ZAF = 61%).

2.8 SCREENING IN PRISONS

The following table describes the specific changes and costs for screening in prisons in Brazil.

Aspect	Details
**Intervention summary**	
Intervention number, abbreviation, name	8. PRI - Screening in prisons
Summary description	Annually screen all adults in prison for TB using GeneXpert MTB/RIF Ultra, then start TB treatment for all those diagnosed.
Population affected	Incarcerated individuals in Brazil.
**Changes to the model**	
What is the effectiveness of the intervention?	Early diagnosis of bacteriologically confirmed TB, increased DST coverage (Pivetta de Araujo et al., 2024).
Which compartments are affected?	Adults in all states, except those on treatment, on the prison risk dimension.
Which transitions are affected?	The diagnostic pathway, which was added as a separate screening process from the usual diagnostic pathway
Which parameters are changed? From what value to what value?	Access to screening was defined as *iaccess*, and scaled up to 80% of the prison population.<br><br>Test positivity (*itestpos*) values were modified to show the probability of testing positive with GeneXpert Ultra. These values varied by TB state:<br>- *S*, *C,* and *I* = 0.006 (Kendall et al., 2021)<br>- *Dn* = 0.044 (Zifodya et al., 2021)

	- *R and Tc* = 0.040 (World Health Organization, 2023)<br>- *Ds* = 0.775 (Zifodya et al., 2021)<br>- *Dc* = 0.909 (Zifodya et al., 2021)<br><br>Given that Xpert can also be used to test for drug-susceptibility, the probability of receiving a DST (*idst_n* and *idst_p*) was set as 1.<br><br>Mortality on treatment (*imort_st* and *imort_rt*) was null to represent the improvement in outcome due to early diagnosis, slightly increasing as a result of the success and failure.			
Any further details of changes	None			
**Costs**				
What is being costed, and where are the costs applied?	**Activity**	**Resource use**	**Population**	**Model output**
	Standard TB assessment	As BAU (Brazil):<br>**1x** appointment at a clinic<br>**35%** of patients x CXR<br>**50%** of patients x GeneXpert Ultra	Those entering the diagnostic pathway	n_assess_y[n]
	Standard DS-TB treatment	As BAU (Brazil):<br>**7x** monthly appointments at a clinic<br>**6 months** of H+R+Z+E	Those entering treatment for DS-TB	n_treat_DS_y[n]
	Standard DR-TB treatment	As BAU (Brazil):<br>**19x** monthly appointments at a clinic<br>**18 months** of R+Lfx+Z+E	Those entering treatment for DR-TB	n_treat_DR_y[n]
	Prison screening	**1x** appointment in prison<br>**1x** GeneXpert MTB/RIF Ultra	People screened in prison	n_screen_prison_y[n]

Do costs vary by age?	No. This intervention only applies to adults
**Implementation / Scale-up**	
Duration of scale-up	5 years
What happens after implementation?	Remains at 80% for the remaining 20 years
How is this implemented in the model?	Straight line continuous increase from 0% at the start of 2025 to 80% at the end of 2029, and then maintained for 20 years.
How is this implemented in the costings?	The model output n_screen_prison_y[n] counts the exact number of people screened in prison each year, and incorporates scale-up, so the cost of screening is just multiplied by the number screened.
**Assumptions**	
Any other assumptions to note?	Note that ‘n_prison_y’ counts the number of people in prison at any one point - i.e. this intervention is equivalent to having a screening campaign on a particular day once a year and screening (80% of) all the people in prison on that day. It does not take into account the constant entry and exit of individuals from prison throughout the year, and so it is not the case that (80% of) all people who go into prison will be screened, as some who are in prison for less than a year may miss screening.

2.9 SHORTER DS-TB TREATMENT

The following table describes the specific changes and costs for shorter DS-TB treatment across all three settings.

Aspect	Details
**Intervention summary**	
Intervention number, abbreviation, name	9. SDS - Shorter DS-TB treatment
Summary description	The current standard DS-TB treatment (6 months of H+R+Z+E) replaced with 4 months of H+P+Z+M.
Population affected	All individuals receiving treatment for DS-TB.
**Changes to the model**	
What is the effectiveness of the intervention?	We assumed equal effectiveness of the current DS-TB treatment, given observed non-inferiority in a treatment-shortening trial (Dorman et al., 2021)
Which compartments are affected?	None
Which transitions are affected?	None
Which parameters are changed? From what value to what value?	None
Any further details of changes	None

**Costs**				
What is being costed, and where are the costs applied?	**Activity**	**Resource use**	**Population**	**Model output**
	Standard TB assessment	As BAU (varies by country)	Those entering the diagnostic pathway	n_assess_y[n]
	Short DS-TB treatment	**5x** monthly appointments at a clinic<br>**4 months** of H+P+Z+M	Those entering treatment for DS-TB	n_treat_DS_y[n]<br>[subject to scale-up]
	Standard DS-TB treatment	As BAU (same in all countries):<br>**7x** monthly appointments at a clinic<br>**6 months** of H+R+Z+E	Those entering treatment for DS-TB	n_treat_DS_y[n]<br>[subject to scale-down]
	Standard DR-TB treatment	As BAU (varies by country)	Those entering treatment for DR-TB	n_treat_DR_y[n]
Do costs vary by age?	No			
**Implementation / Scale-up**				
Duration of scale-up	5 years			
What happens after implementation?	Remains at 80% for the remaining 20 years			
How is this implemented in the model?	Not directly applied in the model as treatment effectiveness is assumed to be the same for both options, and treatment time is not varied for the purpose of the model to avoid paradoxical increase in reinfection, so no distinction within the model between those treated with standard treatment or short treatment.			

How is this implemented in the costings?	Cost of short DS-TB treatment applied to 16% of those treated for DS-TB at the end of Y1, 32% at the end of Y2, 48% at the end of Y3, 64% at the end of Y4, and 80% at the end of Y5 onwards, with the cost of current DS-TB treatment applied to the rest of the eligible population in each year
**Assumptions**	
Any other assumptions to note?	None.

2.10 SHORTER DR-TB TREATMENT

The following table describes the specific changes and costs for shorter DR-TB treatment across all three settings.

Aspect	Details
**Intervention summary**	
Intervention number, abbreviation, name	10. SDR - Shorter DR-TB treatment
Summary description	The current standard DR-TB treatment (9 months of Lfx+Lzd+Cfz+Z+E+Hh+Bdq in ZAF; 9 months of Lfx+Cfz+Z+E+Hh+Eto+Bdq in IND; 18 months of R+Lfx+Z+E in BRA) replaced with 6 months of BPaLM in all countries.
Population affected	All individuals receiving treatment for DR-TB.
**Changes to the model**	
What is the effectiveness of the intervention?	DR-TB treatment success of 89% (Nyang'wa et al., 2022)
Which compartments are affected?	Individuals starting DR-TB treatment
Which transitions are affected?	DR-TB treatment outcomes
Which parameters are changed? From what value to what value?	Reducing the duration of DR treatment: Increase the value of DR-TB treatment completion rate to reflect the shorter duration of the regimen from 9-18 months to 6 months.<br><br>Increase success in DR-TB (*isucc_rt*) treatment to 0.89 when previous success ranged between 0.52 and 0.55. The difference in increase is assumed to come half from the proportion of mortality on treatment and half from the proportion with unsuccessful treatment. Therefore, mortality on DR-TB (*mort_rt*) treatment also changes. To calculate new *mort_rt* values, we used the following equations:

	- Difference between new and BAU success in DR-TB treatment: new_succ_rt - BAU_succ_rt = difference_succ_rt<br><br>- Calculate half of the difference: difference_succ_rt/2 = half_difference<br><br>- Half of the difference comes from mortality, so the mortality decrease are calculated: BAU_mort_rt - half_difference = new_mort_rt<br><br>The change in *mort_rt* differs by country:<br>- BRA new *mort_rt* = 0.03<br>- BAU_succ_rt = 0.55<br>- BAU_mort_rt = 0.17<br>- IND new *mort_rt* = 0.075<br>- BAU_succ_rt = 0.52<br>- BAU_mort_rt = 0.26<br>- ZAF new *mort_rt* = 0.095<br>- BAU_succ_rt = 0.52<br>- BAU_mort_rt = 0.28			
Any further details of changes	None			
**Costs**				
What is being costed, and where are the costs applied?	**Activity**	**Resource use**	**Population**	**Model output**
	Standard TB assessment	As BAU (varies by country)	Those entering the diagnostic pathway	n_assess_y[n]
	Standard DS-TB	As BAU (6 months of H+R+Z+E)	Those entering treatment for DS-	n_treat_DS_y[n]

	treatment		TB	
	Short DR-TB treatment	**7x** monthly appointments at a clinic<br>**6 months** of B+Pa+L+M	Those entering treatment for DR-TB receiving short regimen	n_treat_DR_short_y[n]
	Standard DR-TB treatment	As BAU (varies by country)	Those entering treatment for DR-TB receiving BAU regimen	n_treat_DR_y[n] - n_treat_DR_short_y[n]
Do costs vary by age?	No			
**Implementation / Scale-up**				
Duration of scale-up from 0% to 80%	5 years			
What happens after implementation?	Remains at 80% for the remaining 20 years			
How is this implemented in the model?	Straight line continuous increase from 0% at the start of 2025 to 80% at the end of 2029, and then maintained for 20 years. Conversely, those not receiving the shorter treatment continue to receive the current DR-TB treatment (100% at start, declining to 20% by 5 years).			
How is this implemented in the costings?	Model outputs (number receiving any DR-TB treatment and number receiving shorter DR-TB treatment) count the exact number treated in the model each year, so unit costs are just applied directly per person to these outputs.			
**Assumptions**				

Any other assumptions to note?	Adverse events caused by standard or shorter DR-TB treatment are not included in the results, either in terms of loss of utility/increased disability or in terms of the cost of additional medical care.

3. SCALE-UP

The figure shows the shape of coverage for each intervention. Notably, for TB vaccines, we implemented the consensus schedule (World Health Organization, 2022), with 2 mass-campaigns for all adults and continued vaccination of 15-19 year olds. Mass community screening would not be maintained for 25 years, as historical and contemporary data suggest the impact will reduce over time (Esmail et al., 2025; Marks et al., 2019). As a mid-point, we assumed a year year scale up to 80%, followed by five annual campaigns, after which screening would stop.

Figure S1. Coverage scale-up for interventions.

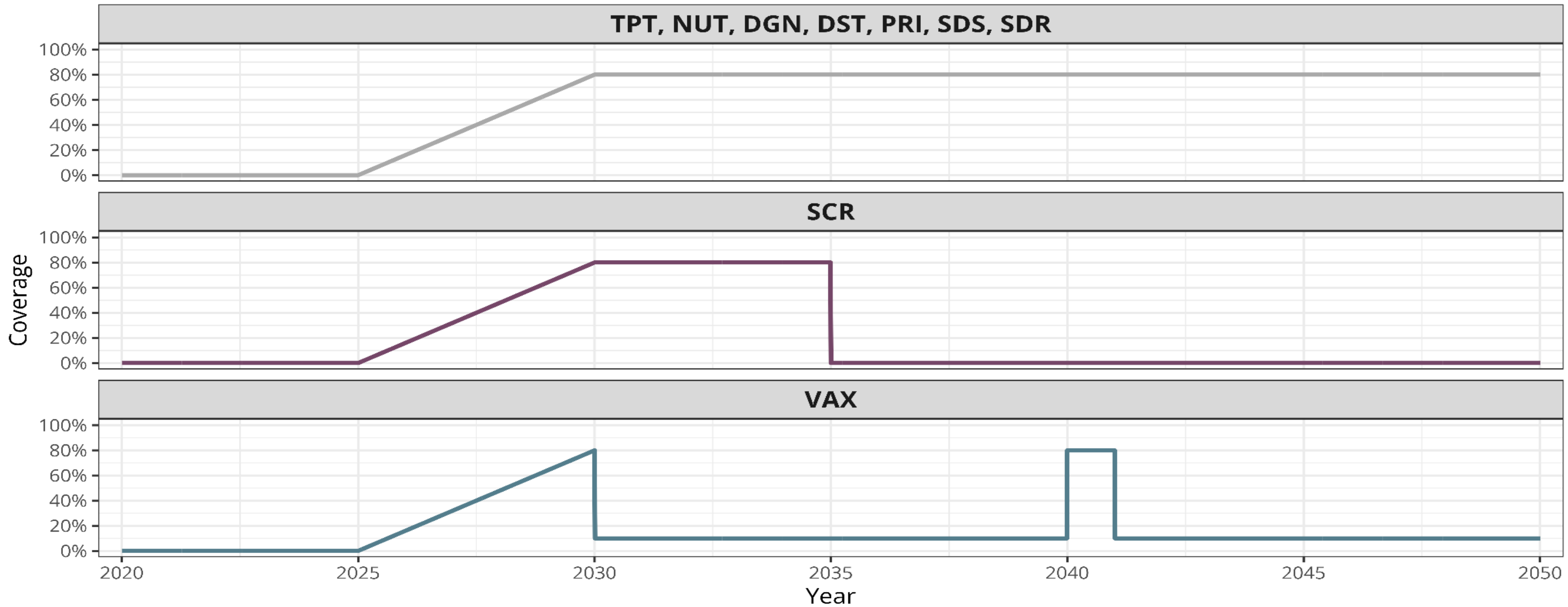

4 REFERENCES

Bhargava, A., Bhargava, M., Meher, A., Benedetti, A., Velayutham, B., Sai Teja, G., Watson, B., Barik, G., Pathak, R. R., Prasad, R., Dayal, R., Madhukeshwar, A. K., Chadha, V., Pai, M., Joshi, R., Menzies, D., & Swaminathan, S. (2023). Nutritional supplementation to prevent tuberculosis incidence in household contacts of patients with pulmonary tuberculosis in India (RATIONS): a field-based, open-label, cluster-randomised, controlled trial. *Lancet*, *402*(10402), 627–640. https://doi.org/10.1016/S0140-6736(23)01231-X

Central Tuberculosis Division. (2023). *India TB Report 2023*. National Tuberculosis Elimination Programme. https://tbcindia.mohfw.gov.in/wp-content/uploads/2023/06/5646719104TB_AR_2023_04-04-2023_LRP_final.pdf

Central Tuberculosis Division. (2024). *India TB Report 2024*. National Tuberculosis Elimination Programme. https://tbcindia.mohfw.gov.in/wp-content/uploads/2024/10/TB-Report_for-Web_08_10-2024-1.pdf

Dorman, S. E., Nahid, P., Kurbatova, E. V., Phillips, P. P. J., Bryant, K., Dooley, K. E., Engle, M., Goldberg, S. V., Phan, H. T. T., Hakim, J., Johnson, J. L., Lourens, M., Martinson, N. A., Muzanyi, G., Narunsky, K., Nerette, S., Nguyen, N. V., Pham, T. H., Pierre, S., … Tuberculosis Trials Consortium. (2021). Four-month rifapentine regimens with or without moxifloxacin for tuberculosis. *The New England Journal of Medicine*, *384*(18), 1705–1718. https://doi.org/10.1056/NEJMoa2033400

Esmail, H., Miller, C., Falzon, D., de Vries, G., Chijioke-Akaniro, O., Horton, K. C., Kohli, M., Dharmapuri Vachaspathi, T., Vo, L. N. Q., Zaidi, S. M. A., Squire, S. B., Coussens, A. K., & Houben, R. M. G. J. (2025). Scaling-up symptom-agnostic, community-wide screening toward global tuberculosis elimination: opportunities, challenges, and lessons from history. *International Journal of Infectious Diseases: IJID: Official Publication of the International Society for Infectious Diseases*, *155*(107875), 107875. https://doi.org/10.1016/j.ijid.2025.107875

Fehr, J., Konigorski, S., Olivier, S., Gunda, R., Surujdeen, A., Gareta, D., Smit, T., Baisley, K., Moodley, S., Moosa, Y., Hanekom, W., Koole,

O., Ndung'u, T., Pillay, D., Grant, A. D., Siedner, M. J., Lippert, C., & Wong, E. B. (2021). Computer-aided interpretation of chest radiography reveals the spectrum of tuberculosis in rural South Africa. *Npj Digital Medicine*, *4*(1), 1–10. https://doi.org/10.1038/s41746-021-00471-y

Fox, G. J., Barry, S. E., Britton, W. J., & Marks, G. B. (2013). Contact investigation for tuberculosis: a systematic review and meta-analysis. *The European Respiratory Journal: Official Journal of the European Society for Clinical Respiratory Physiology*, *41*(1), 140–156. https://doi.org/10.1183/09031936.00070812

Governo Brasileiro. (n.d.). *Sistema de Informação de Agravos de Notificação*. SINAN. Retrieved June 5, 2025, from https://portalsinan.saude.gov.br/

Kendall, E. A., Kitonsa, P. J., Nalutaaya, A., Erisa, K. C., Mukiibi, J., Nakasolya, O., Isooba, D., Baik, Y., Robsky, K. O., Kato-Maeda, M., Cattamanchi, A., Katamba, A., & Dowdy, D. W. (2021). The Spectrum of Tuberculosis Disease in an Urban Ugandan Community and Its Health Facilities. *Clinical Infectious Diseases: An Official Publication of the Infectious Diseases Society of America*, *72*(12), e1035–e1043. https://doi.org/10.1093/cid/ciaa1824

Marks, G. B., Nguyen, N. V., Nguyen, P. T. B., Nguyen, T.-A., Nguyen, H. B., Tran, K. H., Nguyen, S. V., Luu, K. B., Tran, D. T. T., Vo, Q. T. N., Le, O. T. T., Nguyen, Y. H., Do, V. Q., Mason, P. H., Nguyen, V.-A. T., Ho, J., Sintchenko, V., Nguyen, L. N., Britton, W. J., & Fox, G. J. (2019). Community-wide Screening for Tuberculosis in a High-Prevalence Setting. *The New England Journal of Medicine*, *381*(14), 1347–1357. https://doi.org/10.1056/NEJMoa1902129

Martinez, L., Cords, O., Horsburgh, C. R., Andrews, J. R., & Pediatric TB Contact Studies Consortium. (2020). The risk of tuberculosis in children after close exposure: a systematic review and individual-participant meta-analysis. *The Lancet*, *395*(10228), 973–984.

https://doi.org/10.1016/S0140-6736(20)30166-5

McQuaid, C. F., Clark, R. A., White, R. G., Bakker, R., Alexander, P., Henry, R., Velayutham, B., Muniyandi, M., Sinha, P., Bhargava, M., Bhargava, A., & Houben, R. M. G. J. (2025). Estimating the epidemiological and economic impact of providing nutritional care for tuberculosis-affected households across India: a modelling study. *The Lancet. Global Health*, *13*(3), e488–e496. https://doi.org/10.1016/S2214-109X(24)00505-9

National Department of Health, Republic of South Africa. (2023). *National TB Recovery Plan 2.0*. South Africa: MoH. https://tbthinktank.org/wp-content/uploads/2023/09/TB-Recovery-Plan-2.0-April-2023-March-2024.pdf

Nyang'wa, B.-T., Berry, C., Kazounis, E., Motta, I., Parpieva, N., Tigay, Z., Solodovnikova, V., Liverko, I., Moodliar, R., Dodd, M., Ngubane, N., Rassool, M., McHugh, T. D., Spigelman, M., Moore, D. A. J., Ritmeijer, K., du Cros, P., & Fielding, K. (2022). A 24-Week, All-Oral Regimen for Rifampin-Resistant Tuberculosis. *The New England Journal of Medicine*, *387*(25), 2331–2343. https://doi.org/10.1056/NEJMoa2117166

Pivetta de Araujo, R. C., Martinez, L., da Silva Santos, A., Ferreira Lemos, E., Dias de Oliveira, R., Croda, M., Batestin Silva, D. P., Lemes, I. B. G., Cunha, E. A. T., Gonçalves, T. O., Dos Santos, P. C. P., da Silva, B. O., Gonçalves, C. C., Andrews, J. R., & Croda, J. (2024). Serial Mass Screening for Tuberculosis among Incarcerated Persons in Brazil. *Clinical Infectious Diseases: An Official Publication of the Infectious Diseases Society of America*. https://doi.org/10.1093/cid/ciae055

Schwalb, A., Horton, K. C., Emery, J. C., Harker, M. J., Goscé, L., Veeken, L. D., Garden, F. L., Nguyen, H. V., Nguyen, T.-A., Boi, K. L., Cobelens, F., Fox, G. J., Dinh, V. L., Nguyen, H. B., Marks, G. B., & Houben, R. M. G. J. (2025). Potential impact, costs, and benefits of population-wide screening interventions for tuberculosis in Viet Nam: A mathematical modelling study. *PLOS Global Public Health*, *5*(9), e0005050. https://doi.org/10.1371/journal.pgph.0005050

Tait, D. R., Hatherill, M., Van Der Meeren, O., Ginsberg, A. M., Van Brakel, E., Salaun, B., Scriba, T. J., Akite, E. J., Ayles, H. M., Bollaerts, A., Demoitié, M.-A., Diacon, A., Evans, T. G., Gillard, P., Hellström, E., Innes, J. C., Lempicki, M., Malahleha, M., Martinson, N., … Roman, F. (2019). Final analysis of a trial of M72/AS01E vaccine to prevent tuberculosis. *The New England Journal of Medicine*, *381*(25), 2429–2439. https://doi.org/10.1056/NEJMoa1909953

World Health Organization. (2022). *An investment case for new tuberculosis vaccines*. World Health Organization. https://iris.who.int/bitstream/handle/10665/365230/9789240064690-eng.pdf

World Health Organization. (2023). *National tuberculosis prevalence surveys: what diagnostic algorithms should be used in future?* Geneva, Switzerland: WHO. https://iris.who.int/bitstream/handle/10665/367909/9789240073913-eng.pdf?sequence=1

World Health Organization. (2024a). *Global Tuberculosis Report 2024*. Geneva, Switzerland: WHO. https://iris.who.int/bitstream/handle/10665/379339/9789240101531-eng.pdf

World Health Organization. (2024b). *WHO Consolidated Guidelines on Tuberculosis. Module 1: Prevention - Tuberculosis preventive treatment*. Geneva, Switzerland: WHO. https://iris.who.int/bitstream/handle/10665/378536/9789240096196-eng.pdf?sequence=1

Zifodya, J. S., Kreniske, J. S., Schiller, I., Kohli, M., Dendukuri, N., Schumacher, S. G., Ochodo, E. A., Haraka, F., Zwerling, A. A., Pai, M., Steingart, K. R., & Horne, D. J. (2021). Xpert Ultra versus Xpert MTB/RIF for pulmonary tuberculosis and rifampicin resistance in adults with presumptive pulmonary tuberculosis. *Cochrane Database of Systematic Reviews* , *2*, CD009593. https://doi.org/10.1002/14651858.CD009593.pub5

S3 HEALTH ECONOMIC MODEL

This section describes the health economic model used, first describing the analyses and key principles, resource use and cost (including data sources, followed by the calculation of key health effects.

1 ECONOMIC ANALYSES

A cost–utility analysis was conducted for each of the three countries to compare nationwide implementation of each of the modelled interventions with business as usual (BAU) in that country. The epidemiological outputs generated (listed in detail in *Supplementary Material S2*) were combined with the costs associated with each person assessed for TB, treated for TB or subject to the intervention to generate a total cost of implementing each intervention. Disability-adjusted life years (DALYs) were calculated for each intervention based on the number of years of life lost (YLL) and the number of years lived with disability (YLD) due to TB under each intervention. These were combined to calculate the incremental cost-effectiveness ratio (ICER) for each intervention compared to BAU and to display these results on a cost-effectiveness plane, compared with a proposed cost-effectiveness threshold range for each country.

Each intervention was compared to BAU, rather than an incremental analysis comparing each intervention in turn to the next most effective intervention, because these interventions are not exclusive alternatives, and we are not interested only in the single intervention which would be cost-effective compared to all other interventions. National policy-makers may consider implementing more than one of the interventions concurrently, and will be taking into account other considerations such as total budget impact and local feasibility and acceptability in addition to the cost–utility analysis results. Therefore it would not be helpful in this context to exclude an intervention solely due to being dominated by another intervention (that is, being more costly and less effective), but instead we have supplied full costs and DALY results for all interventions, which can be used to calculate incremental analysis comparing any set of interventions chosen as most relevant in a particular context. Full results can be found in Section 3 of *Supplementary Material S4*.

2 ECONOMIC PRINCIPLES

2.1 PERSPECTIVE

The cost–utility analyses were conducted from the perspective of the health system in each country. This enabled ICERs to be calculated with reference to a cost-effectiveness threshold range in each country, thus allowing the cost-effectiveness of each of the TB control measures investigated in this study to be judged in comparison with other areas of healthcare in each country. This is therefore the most useful perspective for health decision-makers and health funders in each country.

2.2 TIME HORIZON

The analyses consider the effects of the TB interventions being implemented for 25 years from the start of 2025 to the end of 2049 in line with the time period being modelled. We assume a five-year implementation period (2025 - 2029) during which the interventions are steadily rolled out across each country, scaling up to reach an implementation rate of 80% by 2030, which is then maintained for the remaining 20 years. Some interventions, such as community-wide screening and vaccination, have different intervention trajectories post-scale-up.

The expected future health effects of TB episodes and deaths due to TB which occur during the study period are included in the analyses, even if part of these effects would fall after 2049 – for example, if an individual dies from TB in 2045, then the years of life lost included in the calculations of DALYs are based on their age at death and their standard life expectancy had they not had TB, and are not capped at 5 years. The total budget costs of each intervention include all costs of the intervention, as well as TB diagnosis and TB treatment required over the whole 25-year period. During the implementation phase, these costs reflect the gradual scale-up of the interventions. We note that some interventions may be more challenging than others to roll out nationally to 80% coverage within a 5-year timeframe, and that some interventions (notably vaccination) are not currently ready for large scale implementation, however the aim of this analysis is to compare the potential future effects of all of the interventions studied on an equal basis.

2.3 DISCOUNTING

Costs and health benefits in future years are discounted at 5% annually for each country. In South Africa, a 5% discount rate is specified in the Essential Drugs Programme reference case (National Department of Health, 2023). India and Brazil have no officially recommended discount rates (Sharma et al., 2021), and expert opinion varies on the optimal approach (Severens & Milne, 2004). We note the guidance of Haacker et al. that discount rates for lower-middle-income countries should be at least 5% (Haacker et al., 2020), and have chosen to use the same rate in

each of the countries to aid in the comparability of the results. Discounting is calculated using the following formula, where *r* is the discount rate and *n* is the number of years into the future:

$$Present\ value = \frac{1}{(1+r)^n}$$

2.4 CURRENCIES AND COST YEAR

Costs and economic results in the paper are reported in US dollars (US$) for the convenience of comparison between countries and for the international audience. These results are repeated below in both US$ and local currencies for each country. Costs are reported for 2023, as that was the latest year for which cost data were available during the study.

2.5 COMPOSITE MEASURES OF TOTAL HEALTH

To assess the overall impact of health interventions on the total health of a population, measures of health which combine both morbidity and mortality into a single valuation are preferred. The most well-known and widely accepted of these are the disability-adjusted life year (DALY) and the quality-adjusted life year (QALY). In this study, we report outcomes in DALYs, to be consistent with the majority of existing tuberculosis studies in the target countries, to aid in comparison.

2.6 COST-EFFECTIVENESS THRESHOLDS

We have chosen to use cost-effectiveness threshold estimates (US$ per DALYs averted) calculated by Ochalek et al. for the International Decision Support Initiative for all of the countries (Ochalek et al., 2018). We have selected the highest and lowest of the four estimates Ochalek et al. calculated for each country to produce a range of potential values for the threshold, to illustrate the uncertainty in these values, which are not officially endorsed as policy thresholds in these countries. We inflated these from 2018 to 2023 values by applying the percentage of per capita GDP estimated by Ochalek et al. in 2015 to the 2023 GDP of each country (Ochalek et al., 2018; World Bank, 2025a).

Table E1. Cost-effectiveness threshold ranges for each country

Country	High or low threshold	Value (2015 US$)*	% 2015 GDP	Value (2023 US$)
Brazil	Bottom of threshold range	6048.00	68%	6967.55
Brazil	Top of threshold range	9318.00	104%	10734.72

India	Bottom of threshold range	264.00	17%	413.47
India	Top of threshold range	363.00	23%	568.52
South Africa	Bottom of threshold range	2480.00	41%	2443.59
South Africa	Top of threshold range	3334.00	55%	3285.06

*Source: (Ochalek et al., 2018)

3 RESOURCE USE AND COSTS

We calculated the cost of implementing each intervention across the whole population by assessing the additional resources required for the intervention, as well as the effect of the intervention on the quantity of TB diagnoses and TB treatment provided. We multiply each resource used by the unit cost of that resource in each country and the number of people affected per year to calculate the total cost of each intervention over 25 years.

3.1 RESOURCE USE

The definition of each intervention was described in detail in the Intervention Supplementary Material, and this was translated into a list of resources required per person receiving the intervention. These were principally informed by existing published research studies most closely resembling the interventions being modelled, with details checked by experts and agreed by the study team. We identified 17 separate activities which are required to deliver the 10 interventions, shown in Table E2 below. These activities were further divided into 17 resource use components (see Table E3 below). These components were chosen based on the availability of existing cost data. Each component represents a collection of resources required as part of a TB intervention. For example, ‘DS-TB standard treatment drugs’ includes the cost of each of the four drugs included in a standard six-month treatment regimen for DS-TB. ‘CXR at mobile van’ includes all the resources required to run a mobile screening van, including the vehicle, CXR machine and staffing, divided proportionately among the expected number of users. Resource use includes equipment (such as CXR and GeneXpert machines), drugs, consumable supplies, and staffing. Unrelated health costs, that is, any impacts on future demand for healthcare unrelated to TB, are not included in this analysis, nor are potential impacts on other areas of public spending, such as education or welfare.

Table E2. Activities making up each intervention.

	Intervention \ Activity	Vaccination	HHCM assessment	TPT for DS-TB	TPT for DR-TB	Nutrition for TB patients	Nutrition for contacts	Screening CXR	Screening Xpert	Clinic diagnosis - standard	Clinic diagnosis - improved	Drug sensitivity testing	DS-TB treatment - standard	DS-TB treatment - shortened	DR-TB treatment - standard	DR-TB treatment - shortened	Prison screening
BAU	Business as usual									■			■		■		
VAX	Vaccination	■								■			■		■		
TPT	TB preventive treatment		■	■	■					■			■		■		
NTN	Nutrition					■	■			■			■		■		
SCR	Community screening							■	■	■			■		■		
DGN	Improved diagnosis									■	■		■		■		
DST	Drug susceptibility testin									■		■	■		■		
PRI	Screening in prisons									■			■		■		■
SDS	Shorter DS-TB treatmen									■			■	■	■		
SDR	Shorter DR-TB treatmen									■			■		■	■	

Table E3. Resource use components required, per person, for each activity.

Component	Vaccination	HHCM assessment	TPT for DS-TB	TPT for DR-TB	Nutrition for TB patients	Nutrition for household contacts	Screening CXR (high estimate)	Screening CXR (low estimate)	Screening Xpert	Clinic diagnosis – standard [BRA]	Clinic diagnosis – standard [IND]	Clinic diagnosis – standard [ZAF]	Clinic diagnosis - improved	DST	DS-TB treatment - standard	DS-TB treatment - shortened	DR-TB treatment – standard [BRA]	DR-TB treatment – standard [IND]	DR-TB treatment – standard [ZAF]	DR-TB treatment - shortened	Prison screening
Vaccine	2	0	0	0	0	0	0	0	0	0	0	0	0	0	0	0	0	0	0	0	0
Carry out vaccination	2	0	0	0	0	0	0	0	0	0	0	0	0	0	0	0	0	0	0	0	0
Appointment at clinic	0	1	4	7	0	0	0	0	0	1	1	1	1	0	7	5	19	10	10	7	0
Appointment in prison	0	0	0	0	0	0	0	0	0	0	0	0	0	0	0	0	0	0	0	0	1
CXR at clinic/hospital	0	0	0	0	0	0	0	0	0	0.35	0.10	0.03	0	0	0	0	0	0	0	0	0
CXR at mobile van - high estimate	0	0	0	0	0	0	1	0	0	0	0	0	0	0	0	0	0	0	0	0	0
CXR at mobile van - low estimate	0	0	0	0	0	0	0	1	0	0	0	0	0	0	0	0	0	0	0	0	0
Xpert Ultra sample collection/processing in clinic	0	1	0	0	0	0	0	0	1	0.50	0.21	0.61	1	1	0	0	0	0	0	0	0
Xpert Ultra sample collection/processing in prison	0	0	0	0	0	0	0	0	0	0	0	0	0	0	0	0	0	0	0	0	1
DS-TB standard treatment drugs	0	0	0	0	0	0	0	0	0	0	0	0	0	0	1	0	0	0	0	0	0
DS-TB shortened treatment drugs	0	0	0	0	0	0	0	0	0	0	0	0	0	0	0	1	0	0	0	0	0
DR-TB standard treatment drugs	0	0	0	0	0	0	0	0	0	0	0	0	0	0	0	0	1	1	1	0	0
DR-TB shortened treatment drugs	0	0	0	0	0	0	0	0	0	0	0	0	0	0	0	0	0	0	0	1	0
TPT for DS-TB drugs	0	0	1	0	0	0	0	0	0	0	0	0	0	0	0	0	0	0	0	0	0
TPT for DR-TB drugs	0	0	0	1	0	0	0	0	0	0	0	0	0	0	0	0	0	0	0	0	0
Nutritional support for TB patients	0	0	0	0	1	0	0	0	0	0	0	0	0	0	0	0	0	0	0	0	0
Nutritional support for household contacts	0	0	0	0	0	1	0	0	0	0	0	0	0	0	0	0	0	0	0	0	0

3.2 COSTS

3.2.1 COSTS PER COMPONENT

Costs were obtained for each of the resource use components for each country. As for resource use, where possible, costs were taken from the existing published research studies on which each of the interventions was based, where these were applicable to one or more of the target countries. Additional data were taken from other publicly available sources, preferring official national sources where available. Drug prices were taken from the Global Drug Facility international TB drugs price list (Global Drug Facility, 2025). World Health Organisation country-specific costs for inpatient stays and outpatient consultations were used. Costs quoted in previous years or in different currencies were inflated to the common cost year and converted to US dollars using World Bank exchange rates (World Bank, 2025b). Vaccination costs were sourced from costs for the roll-out of the human papillomavirus vaccine among adolescents and expert opinion (Clark 2023).

For the cost of community screening, using chest X-ray in a mobile clinic, the costs obtained varied notably between countries, being considerably higher in South Africa than in Brazil or India. As these interventions are provided to very large populations this introduces large uncertainty into the true potential cost of implementing this intervention at a national scale. We therefore decided to model two alternative scenarios as separate interventions: 'high-cost community screening' using the cost of screening from South Africa in all countries; and 'low-cost community screening' using the Brazilian cost of screening in Brazil and South Africa, and the Indian cost in India. Results for both these alternatives are presented alongside results for BAU and the eight other interventions.

Table E4. Costs by resource use component in each country (2023 US$)

Component	Brazil		India		South Africa	
	Cost	Source	Cost	Source	Cost	Source
Vaccine	2.625	(Clark et al., 2023)	2.625	(Clark et al., 2023)	2.625	(Clark et al., 2023)
Carry out vaccination	2.610	(Clark et al., 2023)	2.610	(Clark et al., 2023)	2.610	(Clark et al., 2023)
Appointment at clinic	4.498	(WHO: Economic Evaluation and Analysis, 2021)	7.795	(WHO: Economic Evaluation and Analysis, 2021)	19.949	(WHO: Economic Evaluation and Analysis, 2021)

Appointment in prison	2.257	(da Silva Santos et al., 2021)	N/A		N/A	
CXR at clinic or hospital	6.744	(da Silva Santos et al., 2021)	4.002	(Chatterjee et al., 2021)	18.292	(Kubjane et al., 2024)
CXR at mobile van - high estimate	41.396	Using South Africa cost	41.396	Using South Africa cost	41.396	(Kubjane et al., 2024)
CXR at mobile van - low estimate	5.360	(da Silva Santos et al., 2021)	2.425	(Datta et al., 2019)	5.360	Using Brazil cost
GeneXpert Ultra sample collection & processing in clinic	19.360	(da Silva Santos et al., 2021; Global Drug Facility, 2025)	24.365	(Chatterjee et al., 2021; Global Drug Facility, 2025)	16.932	(Kubjane et al., 2024)
GeneXpert Ultra sample collection & processing in prison	19.260	(da Silva Santos et al., 2021; Global Drug Facility, 2025)	n/a		n/a	
DS-TB standard treatment drugs	87.831	(Global Drug Facility, 2025)	87.831	(Global Drug Facility, 2025)	87.831	(Global Drug Facility, 2025)
DS-TB shorter treatment drugs	176.389	(Global Drug Facility, 2025)	176.389	(Global Drug Facility, 2025)	176.389	(Global Drug Facility, 2025)
DR-TB standard treatment drugs	3514.819	(Global Drug Facility, 2025)	372.588	(Global Drug Facility, 2025)	343.539	(Global Drug Facility, 2025)
DR-TB shorter treatment drugs	362.898	(Global Drug Facility, 2025)	362.898	(Global Drug Facility, 2025)	362.898	(Global Drug Facility, 2025)
TPT for DS-TB drugs	4.247	(Global Drug Facility, 2025)	4.247	(Global Drug Facility, 2025)	4.247	(Global Drug Facility, 2025)

TPT for DR-TB drugs	9.648	(Global Drug Facility, 2025)	9.648	(Global Drug Facility, 2025)	9.648	(Global Drug Facility, 2025)
Nutritional support for TB patients	n/a		16.527	(Bhargava et al., 2023)	n/a	
Nutritional support for household contact	n/a		5.968	(Bhargava et al., 2023)	n/a	

3.2.2 COSTS PER ACTIVITY

Applying the component costs in Table E4 to the resource use in Table E3 gives the following costs for each activity:

Table E5. Costs by activity in each country (2023 US$)

Activity	Parameter name	Activity cost		
		Brazil	India	South Africa
Vaccination	c_a_vax	10.470	10.470	10.470
HHCM assessment	c_a_HHCM	23.858	32.161	36.882
TPT for DS-TB	c_a_TPT_DS	22.241	35.428	84.044
TPT for DR-TB	c_a_TPT_DR	41.136	64.215	149.292
Nutrition for TB patients	c_a_nutrition_index	n/a	16.527	n/a
Nutrition for household contacts	c_a_nutrition_contact	n/a	5.968	n/a
Community screening CXR (high-cost estimate)	c_a_screen_CXR_hi	41.396	41.396	41.396

Community screening CXR (low-cost estimate)	c_a_screen_CXR_lo	5.360	2.425	5.360
Community screening Xpert Ultra	c_a_screen_Xpert	19.360	24.365	16.932
Clinic diagnosis - standard	c_a_diag_standard	16.539	13.312	30.849
Clinic diagnosis - improved	c_a_diag_improved	23.858	32.161	36.882
Drug sensitivity testing	c_a_DST	19.360	24.365	16.932
DS-TB treatment - standard	c_a_treat_DS_standard	119.320	142.398	227.476
DS-TB treatment - shorter	c_a_treat_DS_improved	198.881	215.366	276.135
DR-TB treatment - standard	c_a_treat_DR_standard	3600.287	450.541	543.030
DR-TB treatment - shorter	c_a_treat_DR_improved	394.386	417.465	502.542
Prison screening	c_a_prison	21.517	n/a	n/a

To capture uncertainty in costs we generated a gamma distribution around each point estimate in Table E5 using a broad range of +/-50%, which was chosen to be conservative. Using these distributions we randomly sampled 500 sets of costs for each country, without correlating costs within each set. Each cost set was randomly matched to one of the epidemiological model parameter sets to provide cost multipliers for each activity, generating 500 unique runs for each intervention.

3.2.3 COSTS PER INTERVENTION

The cost of each intervention was calculated annually, based on relevant model outputs for each activity (for example, the number of people receiving the intervention, or the number of people receiving treatment for DS-TB), multiplied by the activity costs above, then summed across the 25-year span of the intervention. Calculations were based on the following equations for each intervention, where y[n] represents the year in the model (from y1 to y25). For the improved diagnosis and shorter DS-TB treatment interventions, annual adjustments were made to calibrate the scale-up of the intervention from 0% to 80% of those eligible over the first 5 years, thereafter remaining at 80%. For the other interventions, scale-up of the interventions was embedded in the model outputs, included in these calculations (see *Supplementary Material S2*

Interventions for more information on the model outputs and the methodology used to scale up each of the interventions). Future costs were discounted as explained in section 2.3 above.

Table E6. Cost equations by intervention

Code	Intervention	Cost equation
BAU	Business as usual	n_assess_y[n] * c_a_diag_standard + n_treat_DS_y[n] * c_a_treat_DS_standard + n_treat_DR_y[n] * c_a_treat_DR_standard
VAX	Vaccination	n_assess_y[n] * c_a_diag_standard + n_treat_DS_y[n] * c_a_treat_DS_standard + n_treat_DR_y[n] * c_a_treat_DR_standard + n_vaccinated_y[n] * c_a_vax
TPT	TB preventive treatment	n_assess_y[n] * c_a_diag_standard + n_treat_DS_y[n] * c_a_treat_DS_standard + n_treat_DR_y[n] * c_a_treat_DR_standard + (n_HHC_DS_y[n] + n_HHC_DR_y[n]) * c_a_HHCM + n_HHC_DSTPT_y[n] * c_a_TPT_DS + n_HHC_DRTPT_y[n] * c_a_TPT_DR
NTN	Nutrition	n_assess_y[n] * c_a_diag_standard + n_treat_DS_y[n] * c_a_treat_DS_standard + n_treat_DR_y[n] * c_a_treat_DR_standard + n_nutrition_index_y[n] * c_a_nutrition_index + n_nutrition_HHC_y[n] * c_a_nutrition_contact
SCR	Community screening	n_assess_y[n] * c_a_diag_standard + n_treat_DS_y[n] * c_a_treat_DS_standard + n_treat_DR_y[n] * c_a_treat_DR_standard + n_screen_y[n] * c_a_screen_CXR + n_screen_CXRpositive_y[n] * c_a_screen_Xpert
DGN	Improved diagnosis	n_assess_y[n] * ([scale-down %] * c_a_diag_standard + [scale-up %] * c_a_diag_improved) + n_treat_DS_y[n] * c_a_treat_DS_standard + n_treat_DR_y[n] * c_a_treat_DR_standard
DST	Drug susceptibility testing	n_assess_y[n] * c_a_diag_standard + n_treat_DS_y[n] * c_a_treat_DS_standard + n_treat_DR_y[n] * c_a_treat_DR_standard + n_diag_noXpert_y[n] * c_a_DST
PRI	Screening in prisons	n_assess_y[n] * c_a_diag_standard + n_treat_DS_y[n] * c_a_treat_DS_standard + n_treat_DR_y[n] * c_a_treat_DR_standard + n_prison_y[n] * c_a_prison
SDS	Shorter DS-TB treatment	n_assess_y[n] * c_a_diag_standard + n_treat_DS_y[n] * ([scale-down %] * c_a_treat_DS_standard + [scale-up %] * c_a_treat_DS_improved) + n_treat_DR_y[n] * c_a_treat_DR_standard

SDR	Shorter DR-TB treatment	n_assess_y[n] * c_a_diag_standard + n_treat_DS_y[n] * c_a_treat_DS_standard + (n_treat_DR_y[n] - n_treat_DR_short_y[n]) * c_a_treat_DR_standard + n_treat_DR_short_y[n] * c_a_treat_DR_improved

The total costs of each intervention include both the cost of implementing the particular intervention and the costs of TB diagnosis in clinics and TB treatment. The amount of TB diagnosis or treatment required may be decreased by the intervention (for example, for preventive interventions), or increased (for example, for diagnostic interventions), whether immediately or in subsequent years, and so these changes will also be captured in the total costs.

For community screening, the cost of the screening (c_a_screen_CXR) was varied between c_a_screen_CXR_hi and c_a_screen_CXR_lo in the high-cost or low-cost scenarios

4 HEALTH EFFECTS

4.1 DALY CALCULATIONS

The time horizon was set at 2050. DALYs were calculated for each intervention in each country, according to the WHO and Global Burden of Disease definition as the sum of years of life lost (YLL) due to premature mortality in the population and years lived with disability (YLD) for people living with the health condition or its consequences (Institute for Health Metrics and Evaluation, 2024; World Health Organization, n.d.). YLL were further divided into years lost from TB mortality (deaths of people with symptomatic clinical TB disease) and post-TB mortality (the increased death rate expected in people who have previously had TB). YLD were further divided into disability for people with symptomatic TB and disability for people with post-TB conditions. DALYs were calculated by combining epidemiological outputs from the modelling in TBMod (such as the number of TB episodes and the number of deaths from TB), life table data, and disability weights, according to the equations below:

YLL = TB death DALYs + Post-TB death DALYs

YLD = TB disability DALYs + Post-TB disability DALYs

TB death DALYs = number of lives lost from TB × expected life years lost per death (discounted)

DALY_mort_TB_y[n]_ag[n] = n_TBdeath_y[n]_ag[n] * LY_healthy_y[n]_ag[n]

Post-TB death DALYs = number of people with post-TB × expected life years lost per person from premature deaths (discounted)

DALY_mort_postTB_y[n]_ag[n] = n_postTB_y[n]_ag[n] * LY_lost_postTB_y[n]_ag[n]

TB disability DALYs = number of people with clinical TB × duration of clinical TB × disability weight for TB × discount factor

DALY_dis_TB_y[n]_ag[n] = n_Dc_y[n]_ag[n] * duration_Dc_ag[n] * dw_TB * (1+disc)^-(n-1)

Post-TB disability DALYs = number of people with post-TB × duration of post-TB (discounted) × disability weight for post-TB

DALY_dis_postTB_y[n]_ag[n] = n_postTB_y[n]_ag[n] * LY_postTB_y[n]_ag[n] * dw_postTB

Where y[n] represents the year in the model (from y1 to y25), and ag[n] represents the age groups in the model (from ag1 [0-4 years] to ag16 [75-99 years]). These were calculated annually over each of the 25 years and for all age groups and were summed to give the total DALYs for each intervention. DALYs averted were calculated by subtracting the total DALYs for a particular intervention from the total DALYs for BAU.

4.2 POST-TB SEQUELAE

A history of previous TB impacts on the future expected health of an individual due to a wide range of 'post-TB' sequelae, contributing a substantial proportion of the DALYs lost due to TB (Menzies et al., 2021; Quaife et al., 2020). This includes both a decrease in the quality of life of the individual (that is, a disability weight should be applied to years of life following TB disease) and an increase in the expected mortality rate above the standard age-based national mortality rate. We adopt the reductions in future morbidity and mortality reported in previous work (Menzies et al., 2021; Quaife et al., 2020).

The effects of post-TB are only applied to people who have experienced symptomatic TB in the model. This is because research on the impacts of post-TB sequelae is based on individuals who have been diagnosed and treated with TB, who will normally have had symptomatic TB. It is possible that there may also be long-term effects of asymptomatic or non-infectious TB, in which case the health burden of post-TB sequelae calculated here may conservatively underestimate the true total burden. The additional mortality impact was not modelled within TBMod, but has been calculated based on an increase in the age-based mortality rate for those individuals who have previously experienced TB.

4.3 DISABILITY WEIGHTS

A disability weight for TB disease (dw_TB) of 0.333 for all countries was sourced from the Global Burden of Disease project (Institute for Health Metrics and Evaluation, 2024). This was applied to each individual in the 'symptomatic TB' state and in the 'TB treatment' states, for as long as they remained in those states. No disability weight was applied to people with asymptomatic TB or non-infectious TB. Total years of life with disability due to TB are therefore calculated from the mean duration of an episode of symptomatic TB in the model (duration_Dc_ag[n]) multiplied by the number of people experiencing symptomatic TB each year (n_Dc_y[n]_ag[n]) multiplied by the disability weight (dw_TB), discounted with respect to the relevant year (equation for 'TB disability DALYs' above). For people who have previously had TB, a disability weight of 0.036 (dw_postTB) was applied for the rest of their lifetimes. This is the median weight found by Menzies et al. in their study of the impact of post-TB sequelae (Menzies et al., 2021).

4.4 LIFE YEARS

Life tables from the UN World Population Prospects were used to calculate for each country the standard (healthy) discounted life expectancy of an individual in each of the 16 age groups used in the modelling in each year from 2025 to 2049 (LY_healthy_y[n]_ag[n]) (United Nations, n.d.). This equates to the years of life lost when an individual in a given age group dies in a given year due to TB (equation 'TB death DALYs' above). A mortality rate modifier of 1.14x was adopted from Menzies et al. (Menzies et al., 2021) to recalculate discounted life expectancy for those who have previously experienced TB (LY_postTB_y[n]ag[n]). This equates to the years lived with disability after an individual in a given age group has symptomatic TB in a given year. The number of people in this group (n_postTB_y[n]_ag[n]) is calculated by subtracting the number of TB deaths from the number of people who experience symptomatic TB for the first time each year (equation 'Post-TB disability DALYs' above). By subtracting post-TB life expectancy from health life expectancy, we calculate the expected years of life lost due to post-TB sequelae (LY_lost_postTB_y[n]_ag[n]). This equates to the years of life lost when an individual in a given age group dies in a given year due to post-TB sequelae (equation 'Post-TB death DALYs' above).

Table E7. Discounted healthy and post-TB life expectancy by year and age group for each country

Year	Age group	Healthy life expectancy			Post-TB life expectancy			Life years lost from post-TB		
		BRA	IND	ZAF	BRA	IND	ZAF	BRA	IND	ZAF

2025 (year 1)	0 - 4 years	19.657	19.451	19.061	19.585	19.363	18.936	0.072	0.087	0.125
	5 - 9 years	19.464	19.288	18.811	19.378	19.194	18.671	0.086	0.094	0.140
	10 - 14 years	19.202	19.013	18.418	19.095	18.901	18.249	0.107	0.112	0.169
	15 - 19 years	18.900	18.666	17.924	18.771	18.532	17.718	0.129	0.135	0.205
	20 - 24 years	18.578	18.257	17.349	18.430	18.098	17.104	0.149	0.159	0.245
	25 - 29 years	18.199	17.764	16.707	18.029	17.577	16.422	0.170	0.187	0.285
	30 - 34 years	17.717	17.155	16.008	17.520	16.935	15.687	0.197	0.220	0.322
	35 - 39 years	17.128	16.421	15.265	16.899	16.164	14.912	0.229	0.257	0.353
	40 - 44 years	16.417	15.546	14.471	16.152	15.248	14.093	0.265	0.298	0.378
	45 - 49 years	15.575	14.511	13.566	15.271	14.170	13.167	0.304	0.341	0.399
	50 - 54 years	14.602	13.332	12.530	14.259	12.949	12.113	0.343	0.383	0.417
	55 - 59 years	13.486	12.033	11.335	13.105	11.615	10.904	0.381	0.418	0.430
	60 - 64 years	12.231	10.615	9.970	11.817	10.171	9.531	0.414	0.444	0.439
	65 - 69 years	10.871	9.110	8.480	10.433	8.653	8.044	0.438	0.457	0.436
	70 - 74 years	9.417	7.606	6.925	8.969	7.153	6.507	0.448	0.454	0.417
	75+ years	7.284	5.573	4.708	6.846	5.153	4.343	0.438	0.420	0.365
2026 (year 2)	0 - 4 years	18.730	18.535	18.170	18.662	18.453	18.052	0.068	0.082	0.117
	5 - 9 years	18.548	18.380	17.934	18.466	18.291	17.802	0.081	0.089	0.132
	10 - 14 years	18.300	18.119	17.563	18.200	18.013	17.404	0.101	0.106	0.159
	15 - 19 years	18.014	17.790	17.096	17.893	17.663	16.903	0.121	0.127	0.193
	20 - 24 years	17.710	17.402	16.553	17.569	17.252	16.322	0.140	0.150	0.231
	25 - 29 years	17.350	16.934	15.945	17.189	16.757	15.677	0.160	0.177	0.268
	30 - 34 years	16.893	16.356	15.281	16.707	16.148	14.977	0.186	0.208	0.303
	35 - 39 years	16.335	15.659	14.571	16.118	15.415	14.238	0.216	0.243	0.334
	40 - 44 years	15.661	14.828	13.811	15.411	14.545	13.454	0.250	0.282	0.357
	45 - 49 years	14.862	13.844	12.946	14.575	13.520	12.568	0.287	0.324	0.378
	50 - 54 years	13.938	12.722	11.955	13.613	12.358	11.560	0.325	0.364	0.395
	55 - 59 years	12.877	11.485	10.814	12.517	11.088	10.404	0.361	0.397	0.409
	60 - 64 years	11.684	10.135	9.511	11.291	9.713	9.094	0.393	0.422	0.418
	65 - 69 years	10.387	8.700	8.090	9.972	8.265	7.675	0.415	0.435	0.415
	70 - 74 years	9.000	7.265	6.607	8.574	6.833	6.209	0.426	0.432	0.398
	75+ years	6.960	5.323	4.493	6.544	4.923	4.145	0.416	0.400	0.348

Healthy and post-TB life expectancy in years 1 and 2 per age group for each country. Values for the complete time horizon (up to 2050) are available in the GitHub repository: https://github.com/lshtm-tbmg/PACE-TB/.

5 REFERENCES

Bhargava, A., Bhargava, M., Meher, A., Benedetti, A., Velayutham, B., Sai Teja, G., Watson, B., Barik, G., Pathak, R. R., Prasad, R., Dayal, R., Madhukeshwar, A. K., Chadha, V., Pai, M., Joshi, R., Menzies, D., & Swaminathan, S. (2023). Nutritional supplementation to prevent tuberculosis incidence in household contacts of patients with pulmonary tuberculosis in India (RATIONS): a field-based, open-label, cluster-randomised, controlled trial. *Lancet*, *402*(10402), 627–640. https://doi.org/10.1016/S0140-6736(23)01231-X

Chatterjee, S., Toshniwal, M. N., Bhide, P., Sachdeva, K. S., Rao, R., Laurence, Y. V., Kitson, N., Cunnama, L., Vassall, A., Sweeney, S., & Baena, I. G. (2021). Costs of TB services in India (no 1). *The International Journal of Tuberculosis and Lung Disease: The Official Journal of the International Union against Tuberculosis and Lung Disease*, *25*(12), 1013–1018. https://doi.org/10.5588/ijtld.21.0105

Clark, R. A., Weerasuriya, C. K., Portnoy, A., Mukandavire, C., Quaife, M., Bakker, R., Scarponi, D., Harris, R. C., Rade, K., Mattoo, S. K., Tumu, D., Menzies, N. A., & White, R. G. (2023). New tuberculosis vaccines in India: modelling the potential health and economic impacts of adolescent/adult vaccination with M72/AS01E and BCG-revaccination. *BMC Medicine*, *21*(1), 288. https://doi.org/10.1186/s12916-023-02992-7

da Silva Santos, A., de Oliveira, R. D., Lemos, E. F., Lima, F., Cohen, T., Cords, O., Martinez, L., Gonçalves, C., Ko, A., Andrews, J. R., & Croda, J. (2021). Yield, Efficiency, and Costs of Mass Screening Algorithms for Tuberculosis in Brazilian Prisons. *Clinical Infectious Diseases: An Official Publication of the Infectious Diseases Society of America*, *72*(5), 771–777. https://doi.org/10.1093/cid/ciaa135

Datta, B., Prakash, A. K., Ford, D., Tanwar, P. K., Goyal, P., Chatterjee, P., Vipin, S., Jaiswal, A., Trehan, N., & Ayyagiri, K. (2019). Comparison of clinical and cost-effectiveness of two strategies using mobile digital x-ray to detect pulmonary tuberculosis in rural India. *BMC Public Health*, *19*(1), 99. https://doi.org/10.1186/s12889-019-6421-1

Global Drug Facility. (2025). *GDF Budgeting Prices for TB Medicines*. Geneva, Switzerland: Stop TB Partnership. https://www.stoptb.org/sites/default/files/documents/2024.12.16_tb_medicines_budgeting_prices_jan_2025_KL.pdf

Haacker, M., Hallett, T. B., & Atun, R. (2020). On discount rates for economic evaluations in global health. *Health Policy and Planning*, *35*(1), 107–114. https://doi.org/10.1093/heapol/czz127

Institute for Health Metrics and Evaluation. (2024, May 16). *Global Burden of Disease Study 2021 (GBD 2021) Disability Weights*. Global Health Data Exchange. https://ghdx.healthdata.org/record/ihme-data/gbd-2021-disability-weights

Kubjane, M., Coetzee, L., Hirasen, K., Jamieson, L., Evans, D., & Meyer-Rath, G. (2024). *National TB Cost Model, South Africa*. Health Economics and Epidemiology Research Office, University of the Witwatersrand, Johannesburg.

Menzies, N. A., Quaife, M., Allwood, B. W., Byrne, A. L., Coussens, A. K., Harries, A. D., Marx, F. M., Meghji, J., Pedrazzoli, D., Salomon, J. A., Sweeney, S., van Kampen, S. C., Wallis, R. S., Houben, R. M. G. J., & Cohen, T. (2021). Lifetime burden of disease due to incident tuberculosis: a global reappraisal including post-tuberculosis sequelae. *The Lancet. Global Health*, *9*(12), e1679–e1687. https://doi.org/10.1016/S2214-109X(21)00367-3

National Department of Health. (2023). *Health Technology Assessment Methods Guide*. Pretoria, South Africa: NDH. https://www.health.gov.za/wp-content/uploads/2024/04/HTA-Methods-Guide_FINAL_Sep-2023.pdf

Ochalek, J., Lomas, J., & Claxton, K. (2018). Estimating health opportunity costs in low-income and middle-income countries: a novel approach and evidence from cross-country data. *BMJ Global Health*, *3*(6), e000964. https://doi.org/10.1136/bmjgh-2018-000964

Quaife, M., Houben, R. M. G. J., Allwood, B., Cohen, T., Coussens, A. K., Harries, A. D., van Kampen, S., Marx, F. M., Sweeney, S., Wallis, R. S., & Menzies, N. A. (2020). Post-tuberculosis mortality and morbidity: valuing the hidden epidemic. *The Lancet. Respiratory Medicine*,

8(4), 332–333. https://doi.org/10.1016/S2213-2600(20)30039-4

Severens, J. L., & Milne, R. J. (2004). Discounting health outcomes in economic evaluation: the ongoing debate. *Value in Health: The Journal of the International Society for Pharmacoeconomics and Outcomes Research*, *7*(4), 397–401. https://doi.org/10.1111/j.1524-4733.2004.74002.x

Sharma, D., Aggarwal, A. K., Downey, L. E., & Prinja, S. (2021). National healthcare economic evaluation guidelines: A cross-country comparison. *PharmacoEconomics Open*, *5*(3), 349–364. https://doi.org/10.1007/s41669-020-00250-7

United Nations. (n.d.). *World Population Prospects - Population Division*. World Population Prospects 2022. Retrieved June 2023, from https://population.un.org/wpp/

WHO: Economic Evaluation and Analysis. (2021). *WHO-CHOICE estimates of cost for inpatient and outpatient health service delivery*. Geneva, Switzerland: WHO. https://www.who.int/publications/m/item/who-choice-estimates-of-cost-for-inpatient-and-outpatient-health-service-delivery

World Bank. (2025a). *GDP per capita (current US$)*. World Bank Open Data. https://data.worldbank.org/indicator/NY.GDP.PCAP.CD

World Bank. (2025b). *World Development Indicators: Exchange rates and prices*. World Bank. https://wdi.worldbank.org/table/4.16#

World Health Organization. (n.d.). *Disability-adjusted life years (DALYs)*. Global Health Observatory WHO. Retrieved September 9, 2024, from https://www.who.int/data/gho/indicator-metadata-registry/imr-details/158

S4 RESULTS

This section provides additional results to those reported in the main paper. Throughout, we report median and 95% quantiles for the uncertainty intervals, except for the ICERS, where we use mean differences (section 3.3-3.4).

1 CALIBRATION PLOTS AND POSTERIORS

See supplementary materials S1, section 7, for calibration targets, data sources and methods.

1.1 CALIBRATION PLOTS

1.1.1 Brazil calibration results

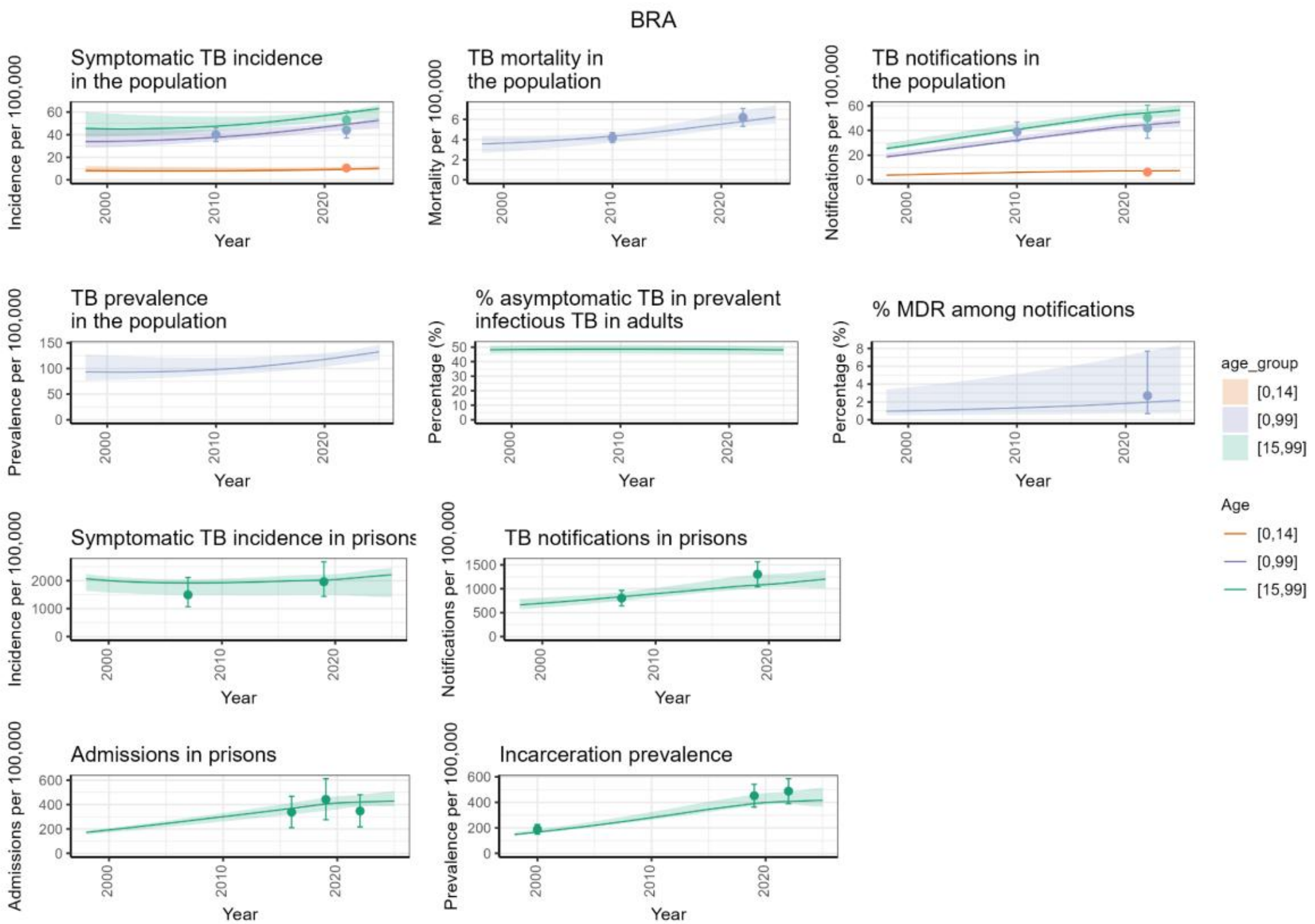

Lines show median model estimates; shaded areas show the range of model estimates across 500 calibrated parameter sets. Points show calibration target estimates; error bars indicate uncertainty in calibration targets.

1.1.2 India calibration results

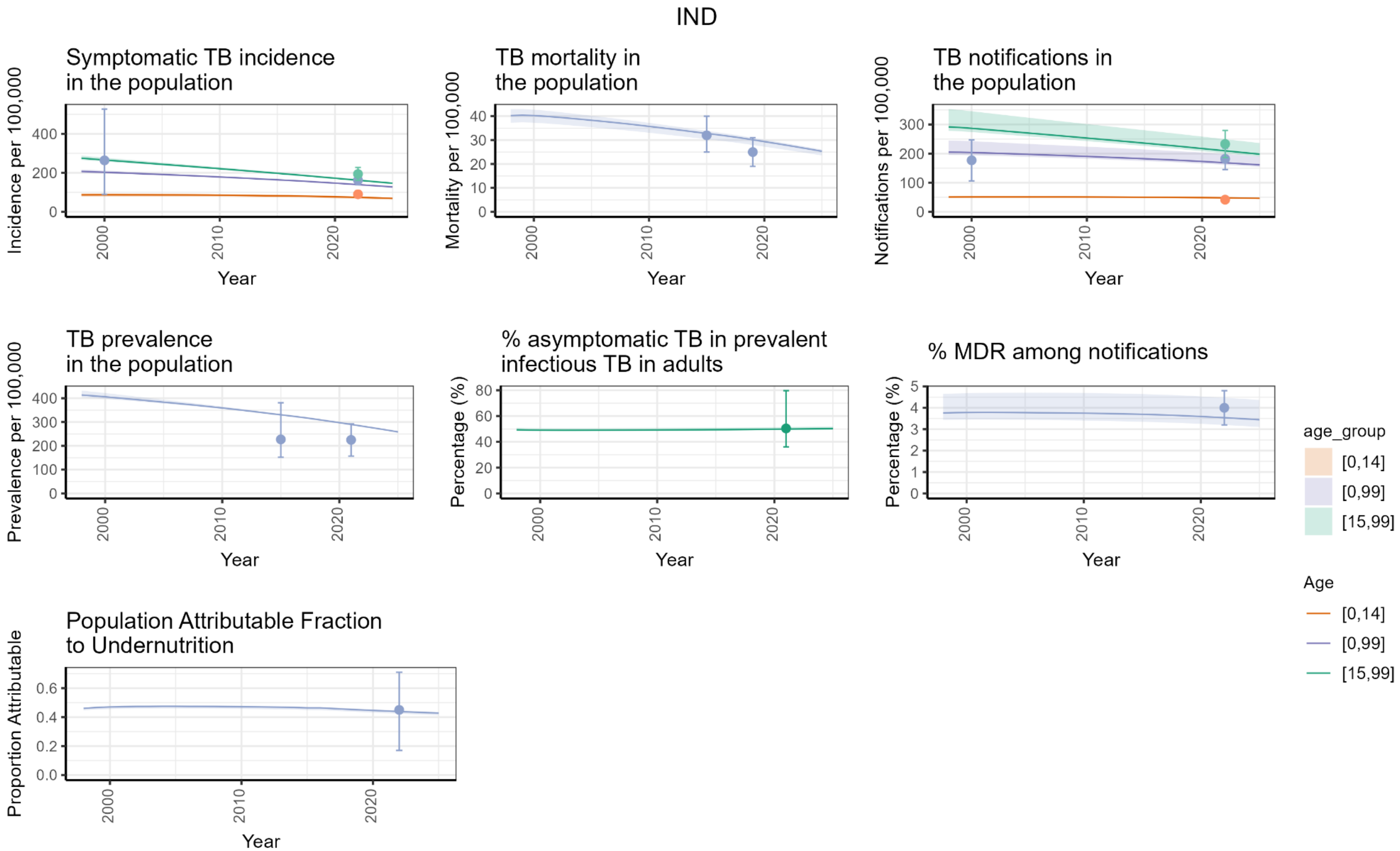

Lines show median model estimates; shaded areas show the range of model estimates across 500 calibrated parameter sets. Points show calibration target estimates; error bars indicate uncertainty in calibration targets.

1.1.3 South Africa calibration results

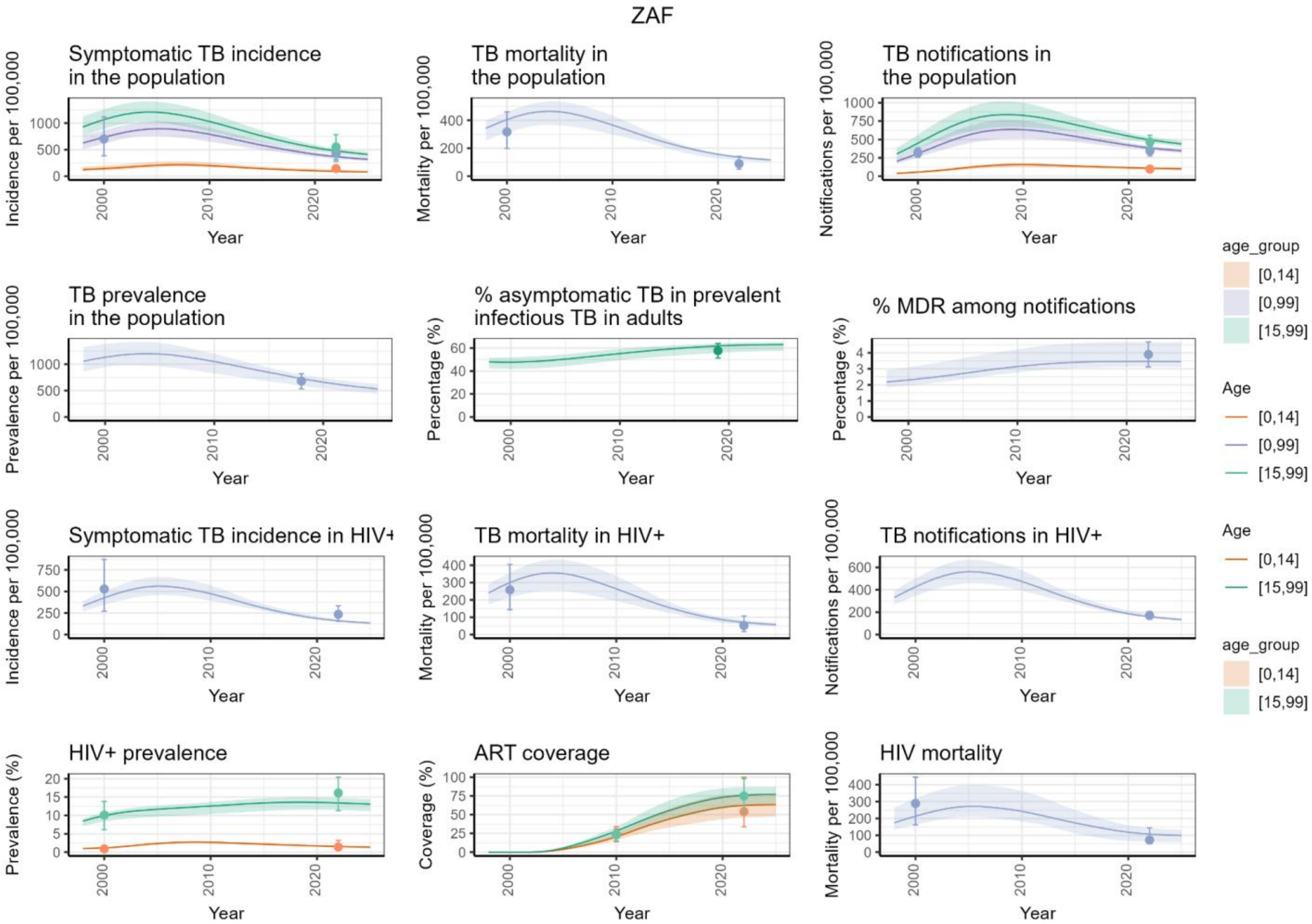

Lines show median model estimates; shaded areas show the range of model estimates across 500 calibrated parameter sets. Points show calibration target estimates; error bars indicate uncertainty in calibration targets.

1.2 CALIBRATION POSTERIORS

Figures posterior distributions of parameters for each country. See supplementary materials S1 for parameters and prior ranges.

1.2.1 Brazil calibration posteriors

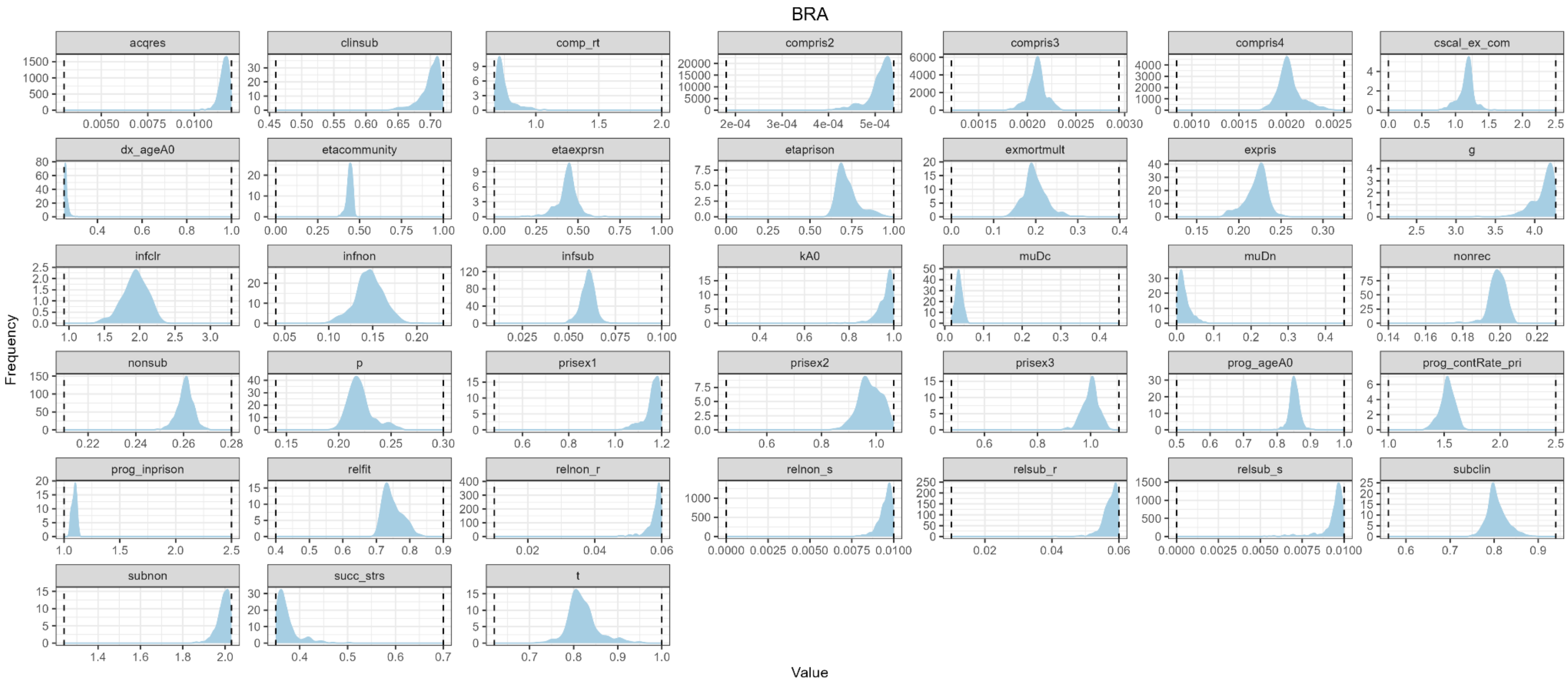

1.2.2 India calibration posteriors

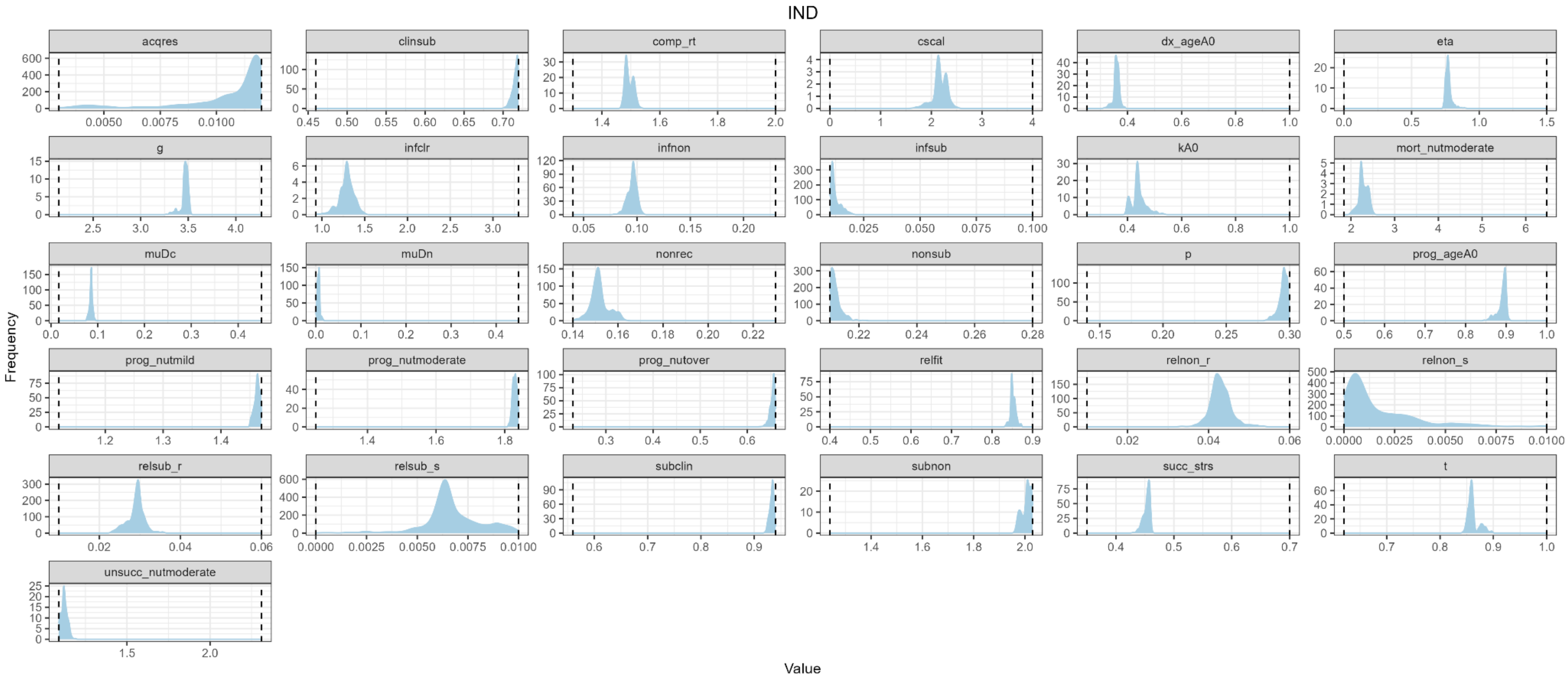

1.2.3 South Africa calibration posteriors

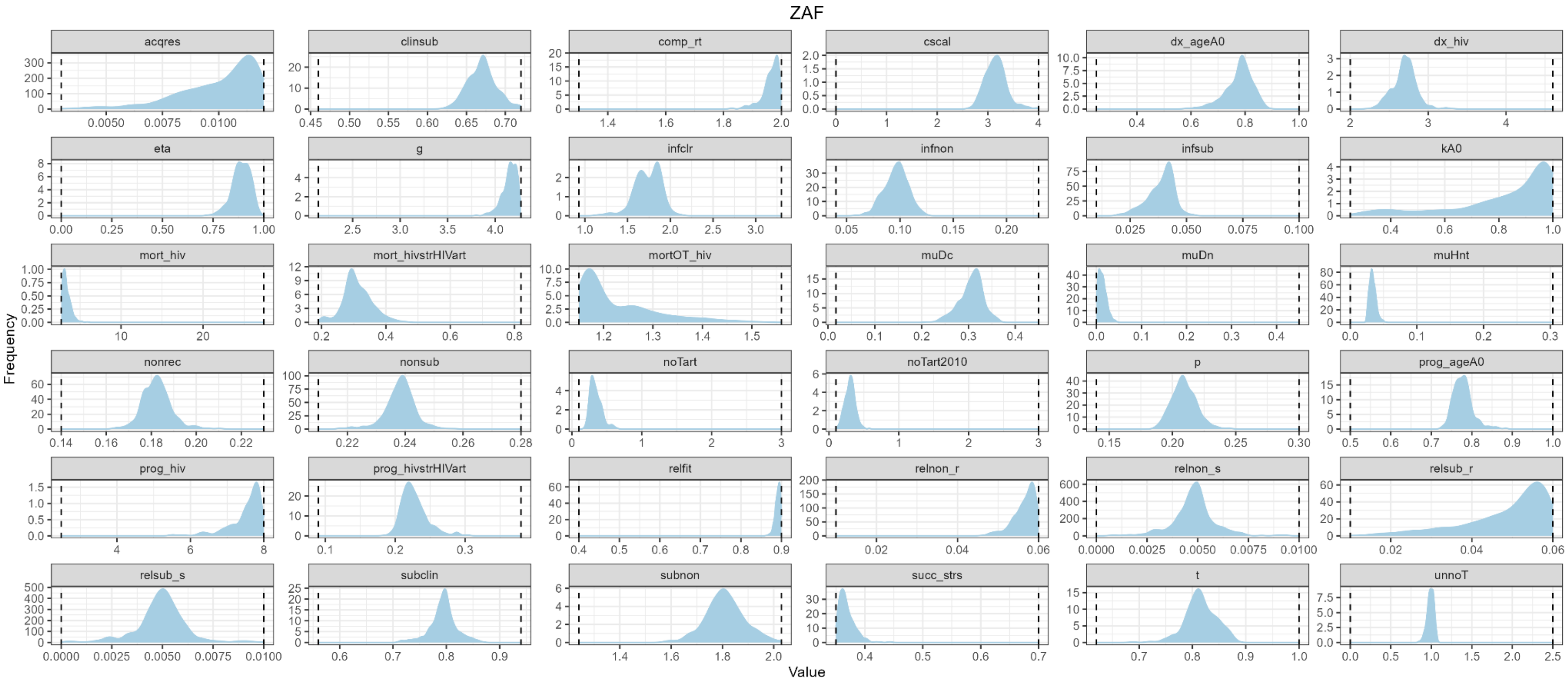

2 EPIDEMIOLOGICAL IMPACT

2.1 TB prevalence trends

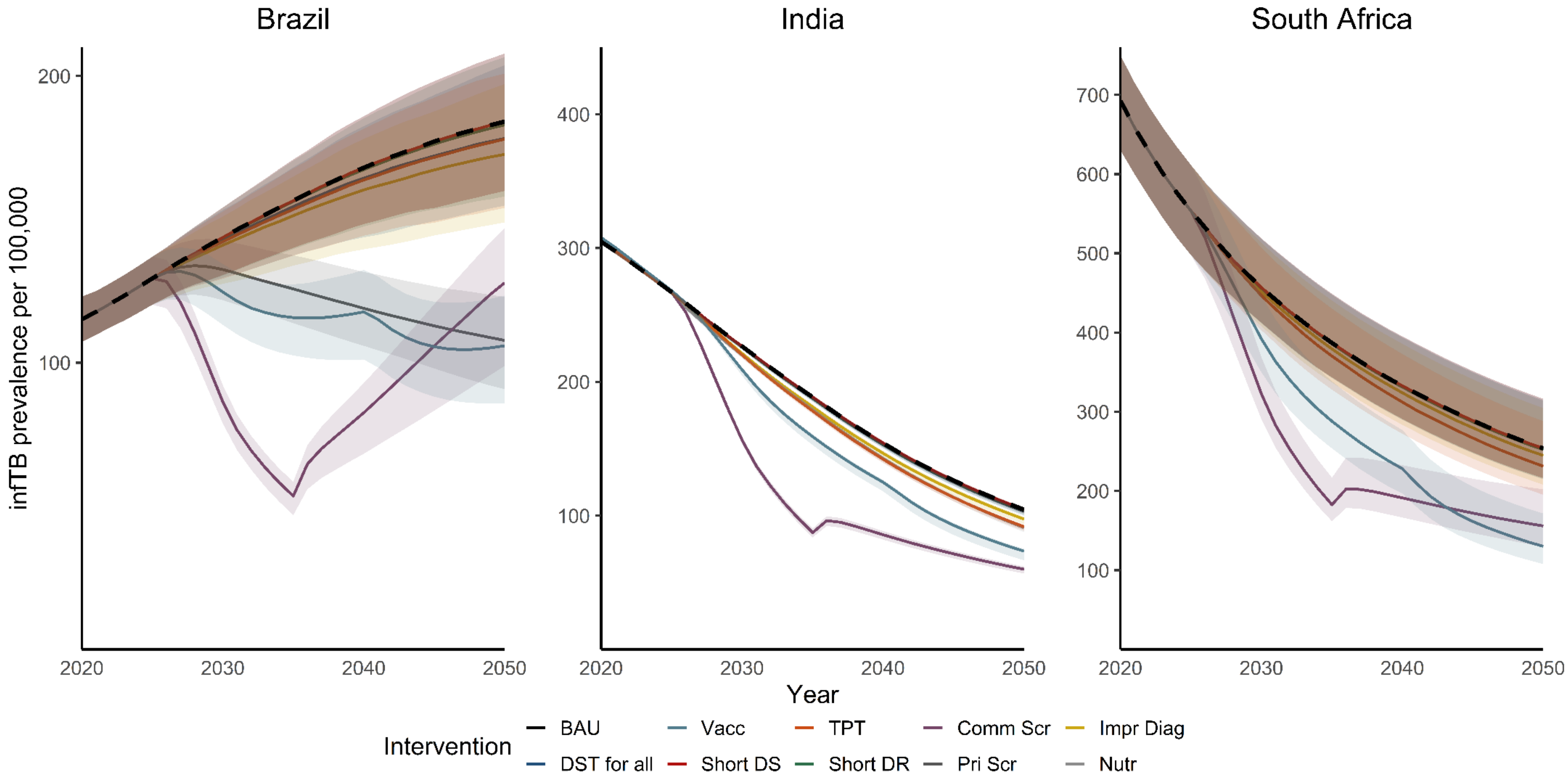

Lines show median model estimates; shaded areas show 95% uncertainty intervals across 500 calibrated parameter sets.

2.2 TB mortality trends

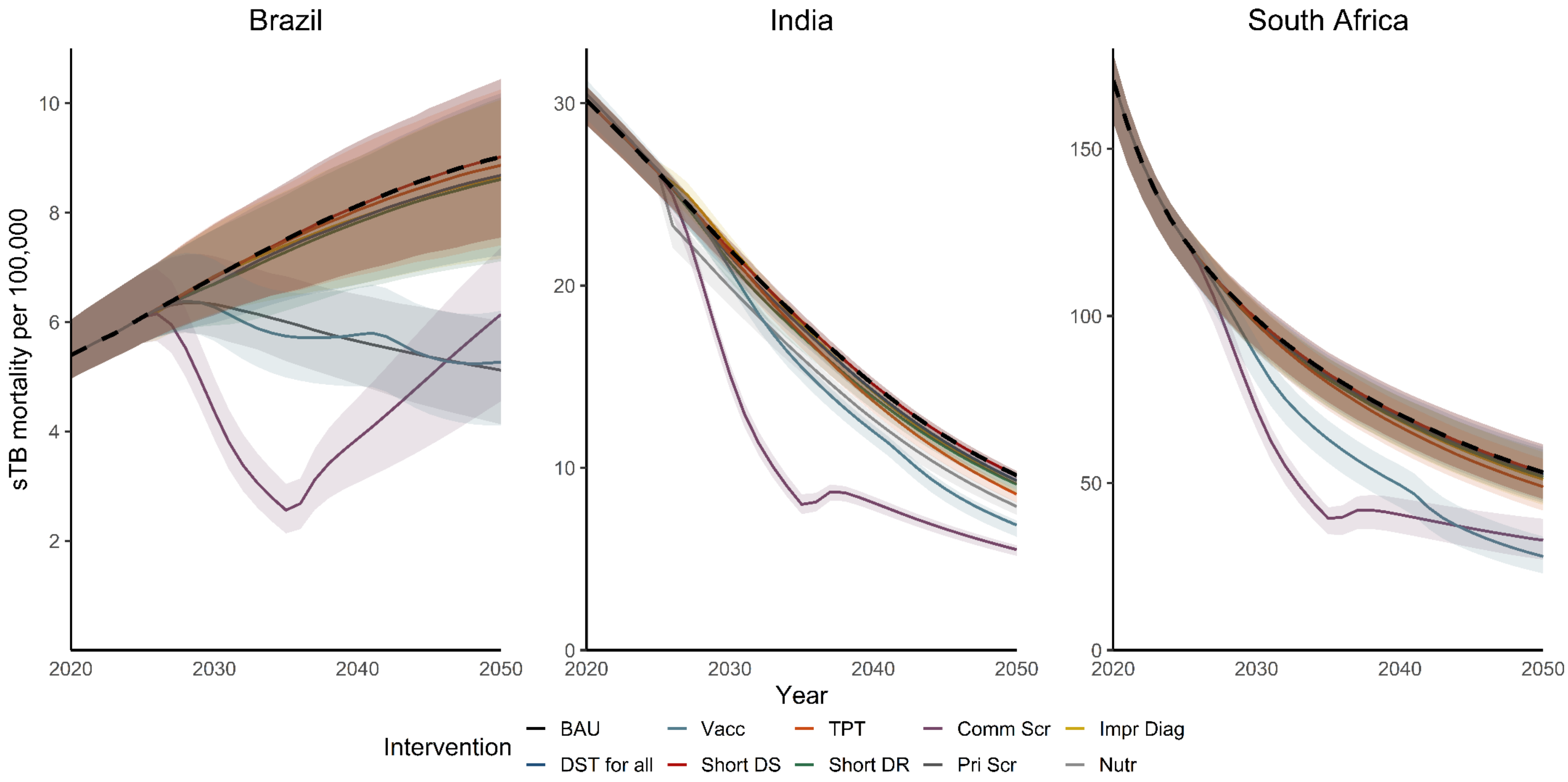

Lines show median model estimates; shaded areas show 95% uncertainty intervals across 500 calibrated parameter sets.

2.3 Incident sTB episodes averted - proportion of total

Incident symptomatic TB episodes averted by 2050 (percent relative to BAU)			
Intervention	Brazil	India	South Africa
Vacc	27.8% (26.5-30.1%)	14.9% (14.3-17.2%)	24.6% (22.2-27.6%)
TPT	1.6% (1.4-1.8%)	4.3% (4.3-4.7%)	3.5% (3-3.9%)
Comm Scr	38% (35.8-42%)	31.7% (30.5-32.8%)	31% (29.8-32.4%)
Impr Diag	2.4% (1.8-6%)	2.2% (2-2.6%)	1.3% (1.2-1.5%)
DST for all	0.8% (0.2-4%)	0.4% (0.3-0.5%)	0.1% (0.1-0.1%)
Short DS	0% (0-0%)	0% (0-0%)	0% (0-0%)
Short DR	0.1% (0-0.7%)	0.3% (0.2-0.3%)	0.2% (0.1-0.2%)
Pri Scr	23.3% (22-25.2%)	-	-
Nutr	-	5.1% (4.9-5.5%)	-

2.4 Incident sTB episodes averted - absolute numbers (1000s)

Incident symptomatic TB episodes averted by 2050 (number relative to BAU, in thousands)			
Intervention	Brazil	India	South Africa
Vacc	1,219K (1,080-1,325K)	6,065K (5,757-6,919K)	1,095K (948-1,308K)
TPT	71K (60-81K)	1,759K (1,726-1,904K)	159K (133-179K)
Comm Scr	1,662K (1,536-1,821K)	12,860K (12,419-13,182K)	1,382K (1,250-1,556K)
Impr Diag	103K (74-262K)	880K (816-1,052K)	60K (51-71K)
DST for all	34K (7-175K)	146K (126-205K)	4K (3-6K)
Short DS	0K (0-0K)	0K (0-0K)	0K (0-0K)
Short DR	6K (2-29K)	105K (92-138K)	7K (5-11K)
Pri Scr	1,022K (870-1,151K)	-	-
Nutr	-	2,050K (2,006-2,255K)	-

2.5 Reduction in TB prevalence in 2050 - proportional (graph)

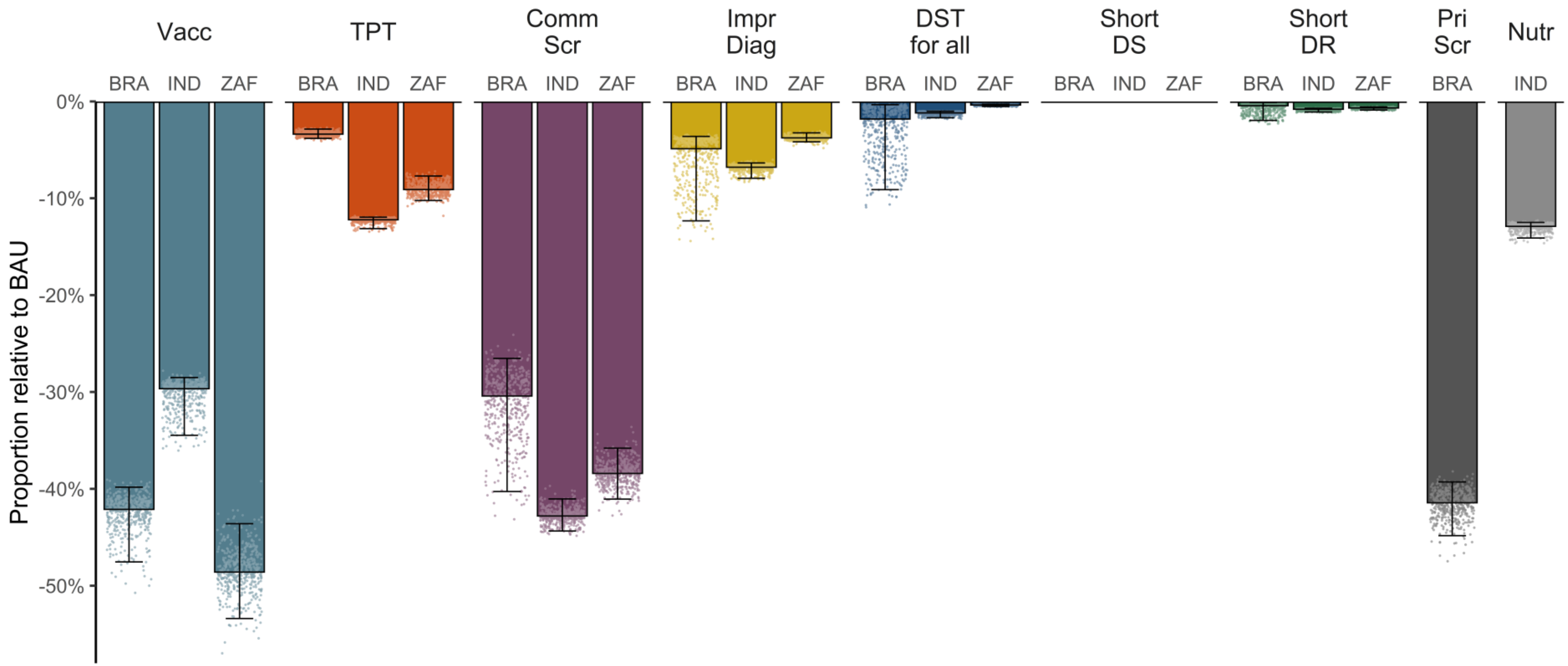

Bars show median model estimates; error bars show 95% uncertainty intervals across 500 calibrated parameter sets; points show model estimates for each calibrated parameter set.

2.6 Reduction in TB prevalence in 2050 - proportional (table)

Decline in infectious TB prevalence in 2050
(percent relative to BAU)

Intervention	Brazil	India	South Africa
Vacc	42.1% (39.8-47.5%)	29.7% (28.5-34.5%)	48.6% (43.6-53.4%)
TPT	3.4% (2.9-3.8%)	12.2% (12-13.1%)	9.1% (7.7-10.2%)
Comm Scr	30.4% (26.5-40.3%)	42.8% (41-44.4%)	38.4% (35.8-41.1%)
Impr Diag	4.9% (3.6-12.3%)	6.8% (6.4-7.9%)	3.8% (3.2-4.2%)
DST for all	1.8% (0.3-9.1%)	1.2% (1-1.7%)	0.4% (0.3-0.5%)
Short DS	0% (0-0%)	0% (0-0%)	0% (0-0%)
Short DR	0.4% (0.1-2%)	0.8% (0.7-1.1%)	0.7% (0.6-0.9%)
Pri Scr	41.4% (39.3-44.8%)	-	-
Nutr	-	12.9% (12.5-14.1%)	-

2.7 TB deaths averted - proportion of total (graph)

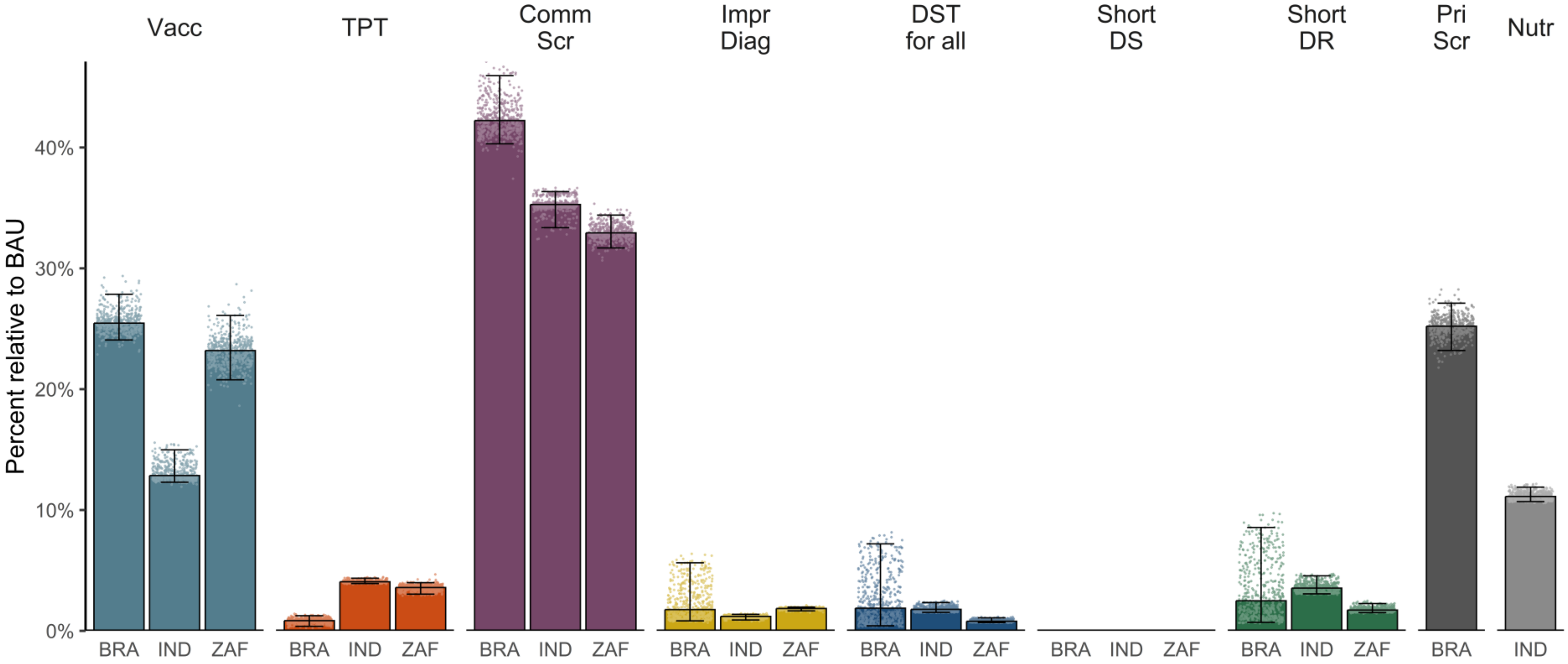

Bars show median model estimates; error bars show 95% uncertainty intervals across 500 calibrated parameter sets; points show model estimates for each calibrated parameter set.

2.8 TB deaths averted - proportion of total

TB deaths averted by 2050 (percent relative to BAU)			
Intervention	Brazil	India	South Africa
Vacc	25.5% (24.1-27.8%)	12.8% (12.3-15%)	23.2% (20.8-26.1%)
TPT	0.8% (0.4-1.3%)	4.1% (3.9-4.4%)	3.6% (3-4%)
Comm Scr	42.2% (40.3-45.9%)	35.3% (33.4-36.3%)	32.9% (31.7-34.4%)
Impr Diag	1.8% (0.8-5.6%)	1.2% (0.9-1.4%)	1.8% (1.6-2%)
DST for all	1.9% (0.4-7.2%)	1.8% (1.5-2.3%)	0.8% (0.7-1.1%)
Short DS	0% (0-0%)	0% (0-0%)	0% (0-0%)
Short DR	2.5% (0.7-8.6%)	3.5% (3.1-4.6%)	1.7% (1.5-2.3%)
Pri Scr	25.2% (23.2-27.1%)	-	-
Nutr	-	11.1% (10.7-11.9%)	-

TB deaths averted by 2050
(number relative to BAU, in thousands)

Intervention	Brazil	India	South Africa
Vacc	117K (101-135K)	874K (804-1,007K)	319K (275-375K)
TPT	4K (2-6K)	274K (261-294K)	50K (40-55K)
Comm Scr	194K (174-217K)	2,392K (2,238-2,458K)	456K (410-498K)
Impr Diag	8K (3-26K)	80K (61-93K)	26K (22-28K)
DST for all	9K (2-33K)	120K (104-159K)	11K (9-15K)
Short DS	0K (0-0K)	0K (0-0K)	0K (0-0K)
Short DR	11K (3-39K)	239K (209-304K)	24K (20-32K)
Pri Scr	115K (97-135K)	-	-
Nutr	-	749K (727-798K)	-

2.10 Disability-adjusted life years - total (millions)

Disability-adjusted life years (number)			
Intervention	Brazil	India	South Africa
BAU	6.5M (5.7-7.2M)	91.5M (88.2-93.4M)	14.8M (13.4-16M)
Vacc	5M (4.4-5.6M)	81M (77.5-83M)	11.8M (10.6-12.8M)
TPT	6.4M (5.7-7.1M)	88.3M (85.1-90.1M)	14.3M (13-15.5M)
Comm Scr	3.9M (3.3-4.4M)	62.3M (59.6-64.8M)	10.3M (9.2-11.1M)
Impr Diag	6.4M (5.6-7.1M)	90.6M (87.4-92.5M)	14.6M (13.2-15.7M)
DST for all	6.4M (5.6-7.1M)	90.3M (87.1-92.3M)	14.7M (13.3-15.9M)
Short DS	6.5M (5.7-7.2M)	91.5M (88.2-93.4M)	14.8M (13.4-16M)
Short DR	6.4M (5.6-7.1M)	89.3M (86-91.4M)	14.6M (13.2-15.7M)
Pri Scr	5.2M (4.6-5.8M)	-	-
Nutr	-	83.4M (80.2-85.3M)	-

2.11 Disability-adjusted life years averted compared to BAU (millions)

Disability-adjusted life years averted (number relative to BAU)			
Intervention	Brazil	India	South Africa
Vacc	1.5M (1.3-1.6M)	10.3M (9.6-11.9M)	3M (2.5-3.4M)
TPT	0.1M (0-0.1M)	3.1M (3-3.4M)	0.4M (0.4-0.5M)
Comm Scr	2.6M (2.4-2.8M)	29.2M (27.7-30M)	4.5M (4.1-4.9M)
DST for all	0.1M (0-0.3M)	1.1M (0.9-1.4M)	0.1M (0.1-0.1M)
Short DS	0M (0-0M)	0M (0-0M)	0M (0-0M)
Short DR	0.1M (0-0.3M)	2.1M (1.8-2.7M)	0.2M (0.2-0.3M)
Pri Scr	1.3M (1.1-1.5M)	-	-
Nutr	-	8M (7.8-8.5M)	-

3 ECONOMIC IMPACT

3.1 Total cost of each intervention

	Budget impact (USD)		
Intervention	Brazil	India	South Africa
BAU	1.6B (1.0-2.4B)	17.5B (12.3-25.6B)	2.5B (1.7-3.6B)
Vacc	3.9B (2.6-5.5B)	33.1B (23.5-44.0B)	3.1B (2.2-4.2B)
TPT	1.6B (1.0-2.4B)	18.7B (13.5-27.0B)	2.7B (1.8-3.7B)
Comm Scr (high)	37.6B (22.6-60.4B)	259.5B (165.7-370.2B)	12.0B (8.4-17.5B)
Comm Scr (low)	8.0B (5.7-10.8B)	49.1B (36.6-63.7B)	4.5B (3.4-5.8B)
Impr Diag	2.1B (1.4-3.4B)	29.0B (20.4-43.2B)	2.7B (1.9-3.7B)
DST for all	1.8B (1.1-2.9B)	18.3B (13.0-26.5B)	2.5B (1.7-3.6B)
Short DS	1.7B (1.1-2.5B)	19.1B (13.3-27.4B)	2.6B (1.7-3.7B)
Short DR	1.3B (0.8-1.9B)	17.2B (12.0-25.2B)	2.5B (1.6-3.5B)
Pri Scr	1.8B (1.2-2.6B)	-	-
Nutr	-	19.7B (14.5-28.0B)	-

3.2 Incremental cost of each intervention compared to BAU

	Incremental cost (USD relative to BAU)		
Intervention	Brazil	India	South Africa
Vacc	2.3B (1.3-3.8B)	15.4B (8.6-24.9B)	0.6B (0.3-1.0B)
TPT	0.0B (0.0-0.1B)	1.2B (0.8-1.8B)	0.2B (0.1-0.2B)
Comm Scr (high)	35.8B (20.8-58.8B)	241.5B (146.9-353.6B)	9.4B (5.9-14.9B)
Comm Scr (low)	6.4B (4.4-9.0B)	30.9B (21.4-42.8B)	1.9B (1.4-2.7B)
Impr Diag	0.5B (-0.2-1.4B)	11.2B (0.8-25.2B)	0.2B (-0.8-1.2B)
DST for all	0.2B (0.1-0.7B)	0.7B (0.5-1.2B)	0.0B (0.0-0.0B)
Short DS	0.1B (0.0-0.2B)	1.5B (-1.1-4.2B)	0.1B (-0.2-0.4B)
Short DR	-0.2B (-1.0--0.1B)	-0.3B (-0.6-0.0B)	0.0B (-0.1-0.0B)
Pri Scr	0.2B (0.1-0.3B)	-	-
Nutr	-	2.2B (1.5-3.1B)	-

3.3 Cost–utility analysis by country

ICERs are calculated based on the mean costs and mean DALYs across the 500 model runs, hence these figures differ slightly from the median values shown in tables 2.9, 2.10, 3.1 and 3.2 above. The probability of being cost-effective at the lower or upper end of the estimated cost-effectiveness threshold range is based on the proportion of the 500 model runs that produced results giving an ICER below the relevant threshold value. For more on how the threshold ranges were selected see *Supplementary Material S3*, section 2.6; these ranges are not official policy thresholds in the respective countries.

3.3.1 Brazil

**Intervention**	**Mean total cost** (billion R$)	**Mean total cost** (billion US$)	**Incremental cost compared to BAU** (million US$)	**Mean total DALYs** (million)	**DALYs averted compared to BAU** (million)	**ICER compared to BAU** (US$ per DALY averted)	**Probability cost-effective at lower end of threshold range** (US$6,968 per DALY)	**Probability cost-effective at upper end of threshold range** (US$10,735 per DALY)
Business as usual	8.04	1.61	-	6.49	-	-	-	-
Vaccination	19.78	3.96	2,353	5.00	1.49	1,582	100%	100%
TB preventive treatment	8.29	1.66	50	6.43	0.06	799	100%	100%
Community screening (high-cost)	192.29	38.54	36,924	3.90	2.59	14,275	1%	18%
Community screening (low-cost)	40.42	8.10	6,490	3.90	2.59	2,509	100%	100%

Improved diagnosis	10.89	2.18	572	6.35	0.14	4,091	81%	92%
Drug susceptibility testing for all	9.27	1.86	247	6.38	0.11	2,190	100%	100%
Shorter DS-TB treatment	8.53	1.71	98	6.49	0	Dominated by BAU	0%	0%
Shorter DR-TB treatment	6.36	1.27	−336	6.38	0.11	Dominates BAU	100%	100%
Screening in prisons	8.99	1.80	191	5.19	1.30	146	100%	100%

3.3.2 India

Intervention	**Mean total cost** (trillion INR)	**Mean total cost** (billion US$)	**Incremental cost compared to BAU** (billion US$)	**Mean total DALYs** (million)	**DALYs averted compared to BAU** (million)	**ICER compared to BAU** (US$ per DALY averted)	**Probability cost-effective at lower end of threshold range** (US$413 per DALY)	**Probability cost-effective at upper end of threshold range** (US$569 per DALY)
Business as usual	1.48	17.92	-	91.2	-	-	-	-
Vaccination	2.77	33.48	15.56	80.7	10.5	1,484	0%	0%
TB preventive treatment	1.58	19.14	1.23	88.1	3.1	394	63%	98%
Community screening (high-cost)	21.69	261.99	244.08	62.1	29.1	8,393	0%	0%
Community screening (low-cost)	4.06	49.09	31.18	62.1	29.1	1,072	0%	0%
Improved diagnosis	2.44	29.43	11.52	90.3	0.9	13,215	2%	2%
Drug susceptibility testing for all	1.55	18.68	0.76	90.1	1.1	691	2%	24%

Shorter DS-TB treatment	1.60	19.36	1.45	91.2	0	Dominated by BAU	0%	0%
Shorter DR-TB treatment	1.46	17.61	−0.31	89.1	2.1	Dominates BAU	100%	100%
Nutrition	1.67	20.17	2.26	83.2	8.0	281	99%	100%

3.3.3 South Africa

**Intervention**	**Mean total cost** (billion R)	**Mean total cost** (billion US$)	**Incremental cost compared to BAU** (million US$)	**Mean total DALYs** (million)	**DALYs averted compared to BAU** (million)	**ICER compared to BAU** (US$ per DALY averted)	**Probability cost-effective at lower end of threshold range** (US$2,444 per DALY)	**Probability cost-effective at upper end of threshold range** (US$3,285 per DALY)
Business as usual	47.22	2.56	-	14.76	-		-	-
Vaccination	57.90	3.14	579	11.80	2.96	196	100%	100%
TB preventive treatment	50.18	2.72	160	14.32	0.44	362	100%	100%
Community screening (high-cost)	227.12	12.31	9,751	10.23	4.53	2,153	72%	97%
Community screening (low-cost)	82.80	4.49	1,929	10.23	4.53	426	100%	100%
Improved diagnosis	50.46	2.74	176	14.55	0.21	824	75%	83%
Drug susceptibility testing for all	47.62	2.58	22	14.67	0.09	246	100%	100%

Shorter DS-TB treatment	48.81	2.65	87	14.76	0	Dominated by BAU	0%	0%
Shorter DR-TB treatment	46.53	2.52	−37	14.57	0.19	Dominates BAU	100%	100%

3.4 Cost-effectiveness planes for all interventions (including high-cost community-wide screening)

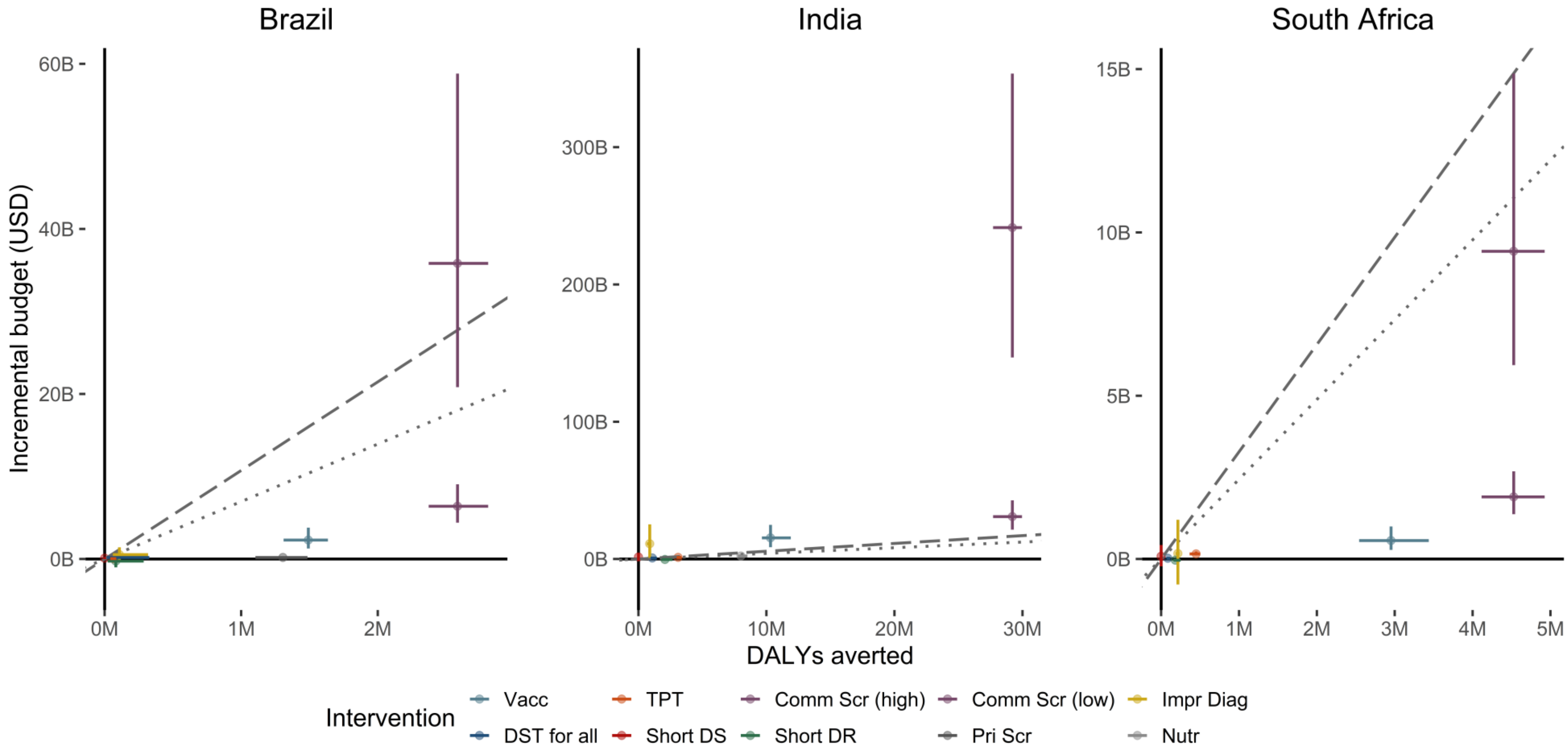

Points show median model estimates with extending lines indicating 95% uncertainty intervals across 500 calibrated parameter sets. The dashed line shows the upper bound and the dotted line the lower bound of the estimated cost-effectiveness threshold range for each country. For a version of these graphs excluding high-cost community screening and showing the remaining interventions in more detail, see Figure 4 in the main paper.

4 SENSITIVITY ANALYSIS: ALIGNED PARAMETERS

4.1 Aligned regression and progression parameters between India and South Africa

We transferred the posterior parameters for TB natural history variables (*infsub, nonsub, nonrec and infclr*) for the South Africa calibration to India. While the calibration targets were not met, the proportional epidemiological impact for vaccination was now similar for India and South Africa.

Reduction in incident symptomatic TB in 2050

	IND	ZAF
Vaccination	30.3% (29.1–35.0)	48.4% (43.3–53.2)
Sensitivity analysis	43.2% (NA–NA)	NA

5 SENSITIVITY ANALYSIS: 50% COVERAGE

Sensitivity analyses assuming 50% coverage (instead of 80% coverage as in the primary analysis) were run for all interventions in each country, except where baseline implementation already exceeds 50% coverage (i.e., “Impr Diag” in BRA and ZAF, and “DST for all” in IND and ZAF).

5.1 TB prevalence trends [50% coverage]

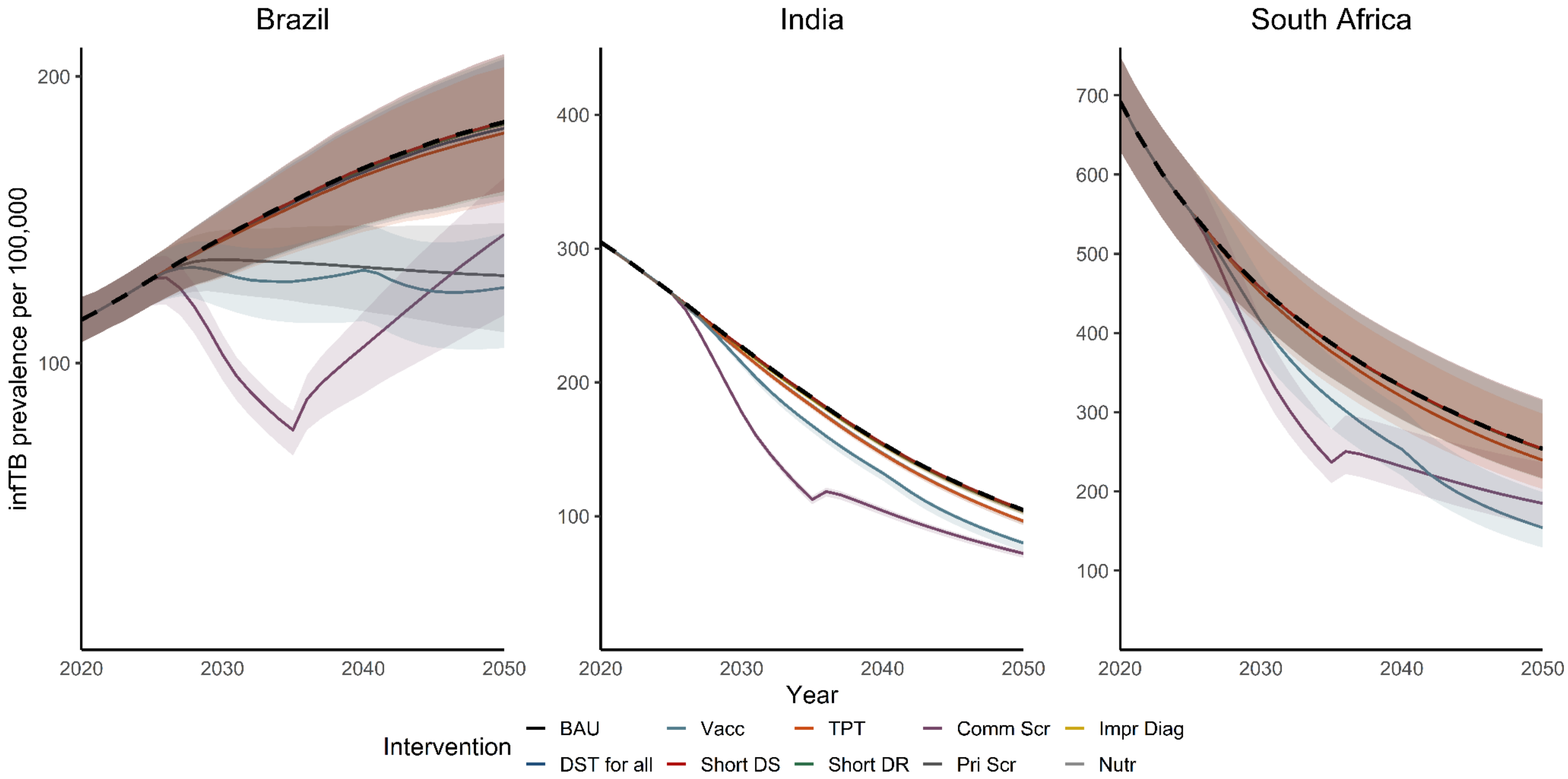

Lines show median model estimates; shaded areas show 95% uncertainty intervals across 500 calibrated parameter sets.

5.2 TB mortality trends [50% coverage]

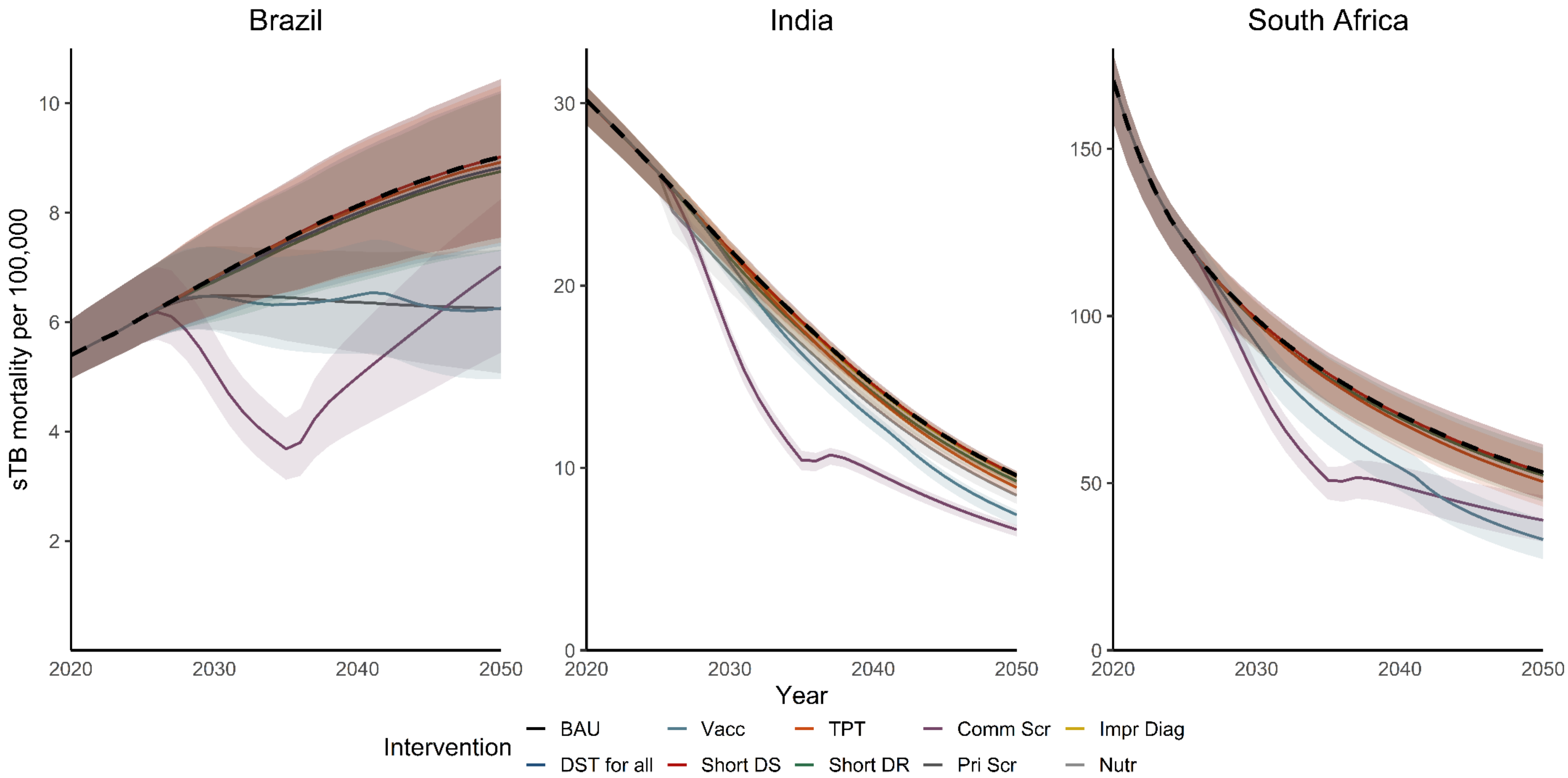

Lines show median model estimates; shaded areas show 95% uncertainty intervals across 500 calibrated parameter sets.

5.3 Incident sTB episodes averted - proportion of total (graph) [50% coverage]

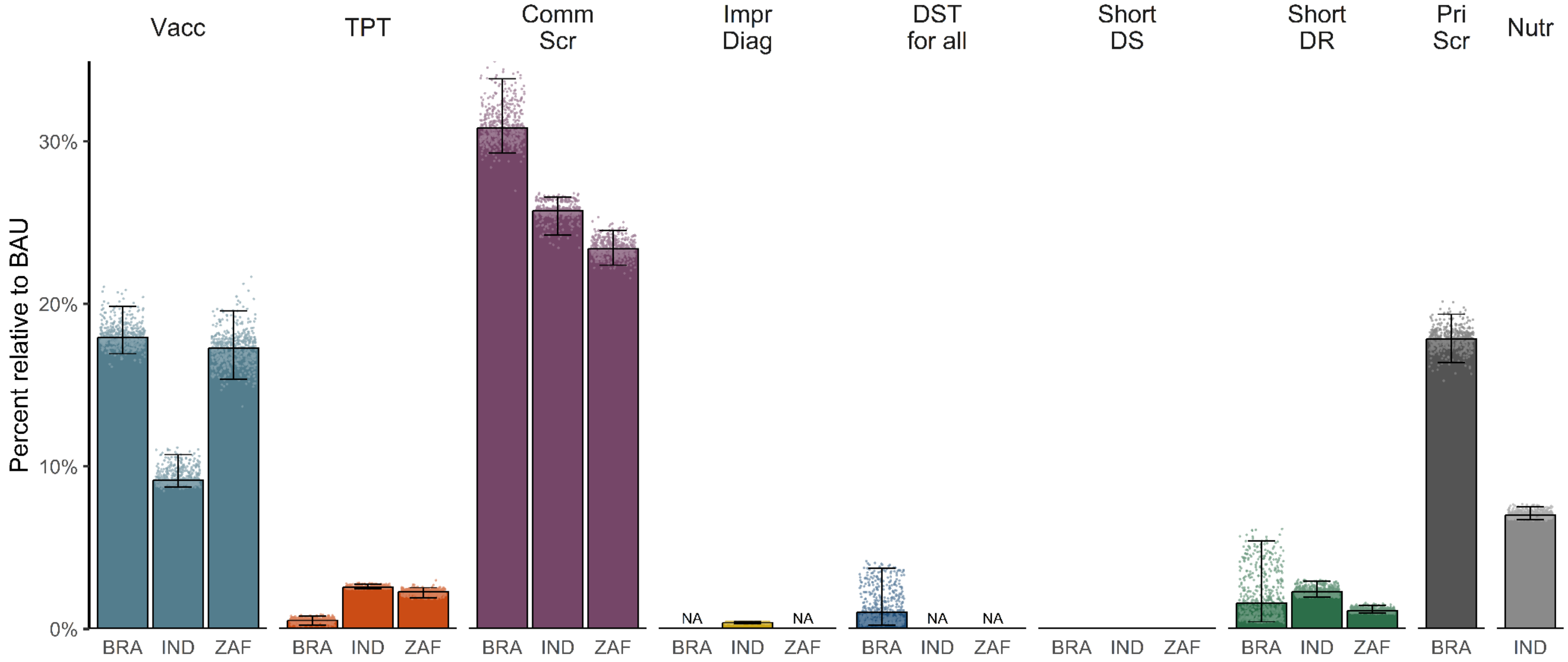

Bars show median model estimates; error bars show 95% uncertainty intervals across 500 calibrated parameter sets; points show model estimates for each calibrated parameter set.

5.4 Incident sTB episodes averted - proportion of total (table) [50% coverage]

Incident symptomatic TB episodes averted by 2050 (percent relative to BAU)			
Intervention	Brazil	India	South Africa
Vacc	19.7% (18.7-21.6%)	10.7% (10.3-12.4%)	18.4% (16.5-20.8%)
TPT	1% (0.9-1.2%)	2.8% (2.7-3%)	2.3% (1.9-2.5%)
Comm Scr	27.3% (25.6-30.6%)	22.9% (22-23.8%)	21.9% (20.9-23%)
Impr Diag	-	0.1% (0.1-0.2%)	-
DST for all	0.3% (0.1-1.6%)	-	-
Short DS	0% (0-0%)	0% (0-0%)	0% (0-0%)
Short DR	0.1% (0-0.4%)	0.2% (0.2-0.2%)	0.1% (0.1-0.1%)
Pri Scr	16.1% (15.3-17.6%)	-	-
Nutr	-	3.2% (3.1-3.5%)	-

5.5 Incident sTB episodes averted - absolute numbers (1000s) [50% coverage]

Incident symptomatic TB episodes averted by 2050 (number relative to BAU, in thousands)			
Intervention	Brazil	India	South Africa
Vacc	866K (769-945K)	4,344K (4,123-4,989K)	820K (705-979K)
TPT	45K (38-51K)	1,116K (1,095-1,213K)	101K (84-114K)
Comm Scr	1,200K (1,103-1,323K)	9,290K (8,943-9,556K)	976K (881-1,095K)
Impr Diag	-	59K (23-88K)	-
DST for all	14K (3-69K)	-	-
Short DS	0K (0-0K)	0K (0-0K)	0K (0-0K)
Short DR	4K (1-18K)	75K (65-98K)	5K (4-7K)
Pri Scr	707K (612-799K)	-	-
Nutr	-	1,287K (1,260-1,417K)	-

5.6 Reduction in TB prevalence in 2050 - proportional (graph) [50% coverage]

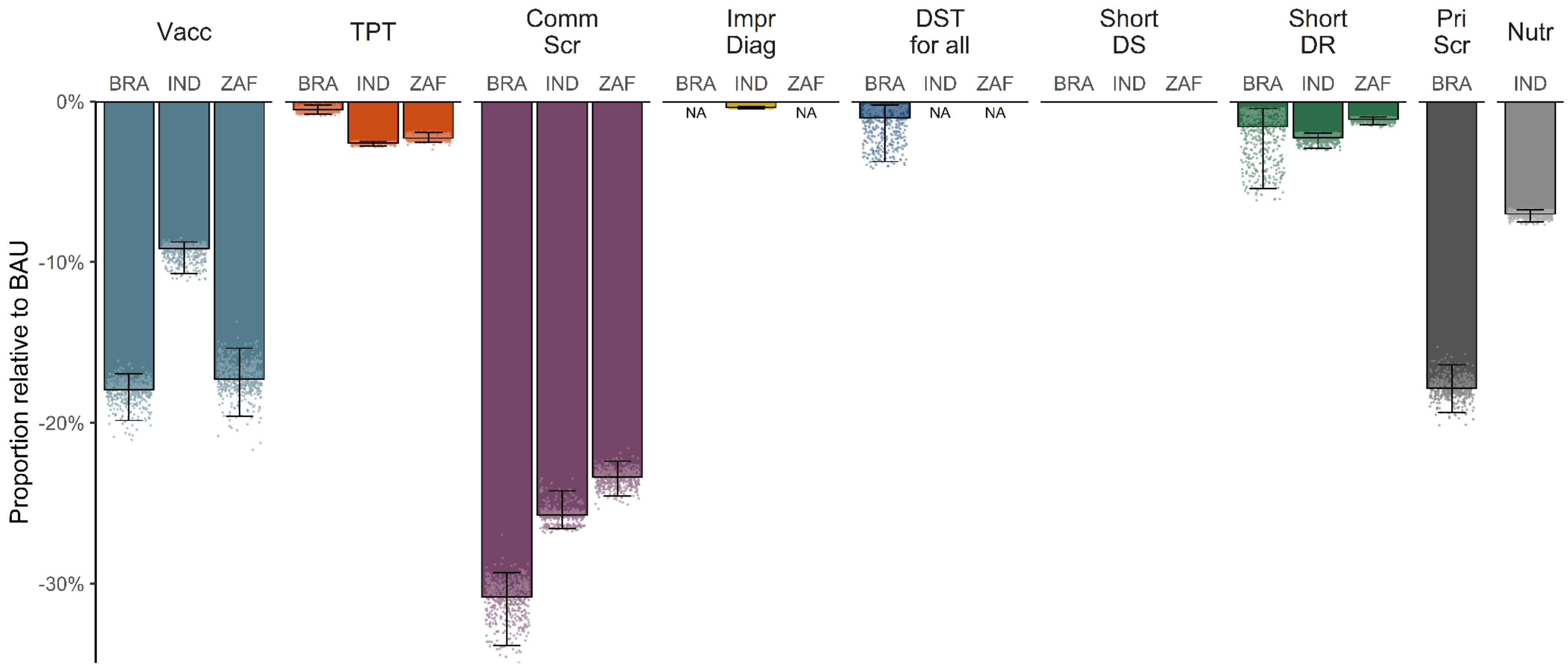

Bars show median model estimates; error bars show 95% uncertainty intervals across 500 calibrated parameter sets; points show model estimates for each calibrated parameter set.

5.7 Reduction in TB prevalence in 2050 - proportional (table) [50% coverage]

Decline in infectious TB prevalence in 2050 (percent relative to BAU)			
Intervention	Brazil	India	South Africa
Vacc	31.2% (29.4-35.7%)	23.5% (22.6-27.4%)	39.1% (34.6-43.4%)
TPT	2.2% (1.8-2.4%)	7.9% (7.7-8.5%)	5.8% (4.9-6.6%)
Comm Scr	21.2% (18.5-28.5%)	31.2% (29.8-32.5%)	27% (24.9-29.1%)
Impr Diag	-	0.8% (0.6-1%)	-
DST for all	0.8% (0.1-3.7%)	-	-
Short DS	0% (0-0%)	0% (0-0%)	0% (0-0%)
Short DR	0.3% (0.1-1.2%)	0.5% (0.5-0.7%)	0.4% (0.4-0.6%)
Pri Scr	29.1% (27.4-32.2%)	-	-
Nutr	-	8.1% (7.9-8.9%)	-

5.8 TB deaths averted - proportion of total (graph) [50% coverage]

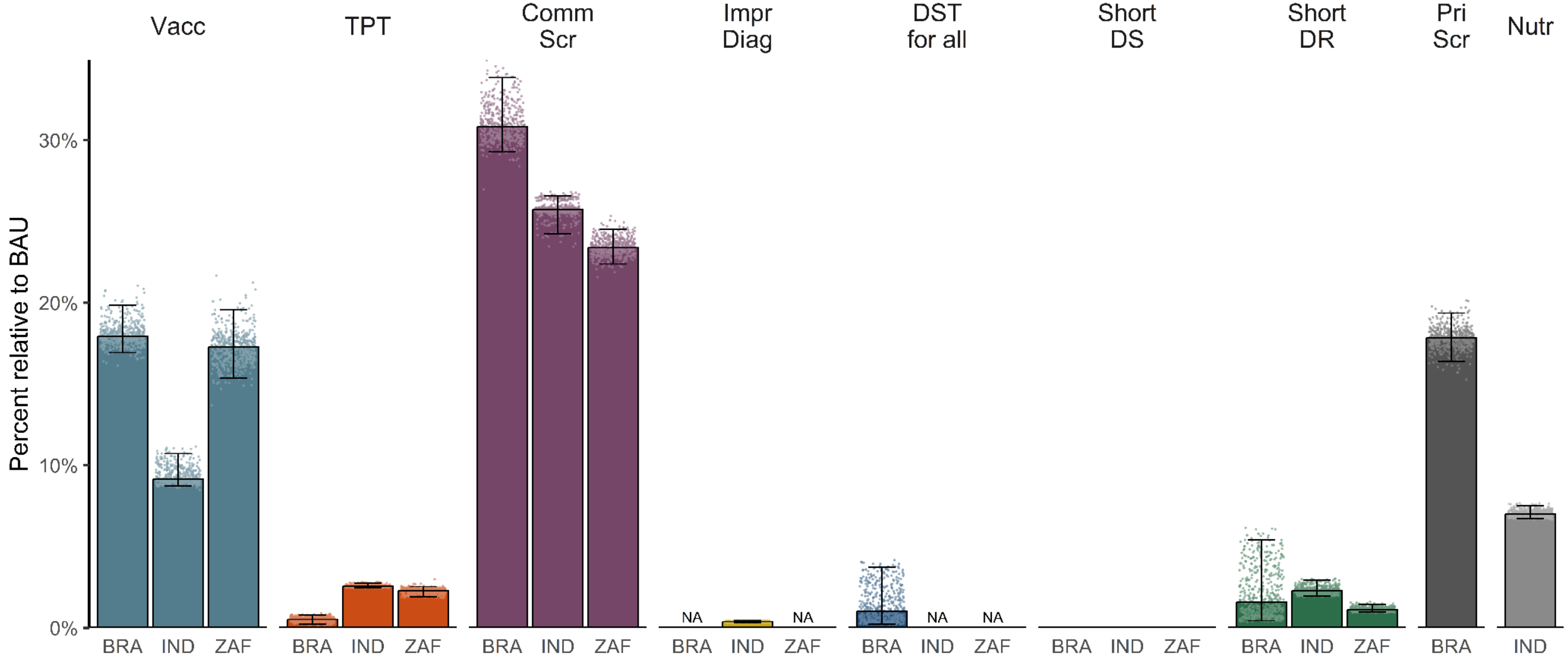

Bars show median model estimates; error bars show 95% uncertainty intervals across 500 calibrated parameter sets; points show model estimates for each calibrated parameter set.

5.9 TB deaths averted - proportion of total [50% coverage]

TB deaths averted by 2050 (percent relative to BAU)			
Intervention	Brazil	India	South Africa
Vacc	18% (16.9-19.9%)	9.2% (8.7-10.7%)	17.3% (15.4-19.6%)
TPT	0.5% (0.2-0.8%)	2.6% (2.5-2.8%)	2.3% (1.9-2.5%)
Comm Scr	30.8% (29.3-33.8%)	25.7% (24.2-26.6%)	23.4% (22.4-24.5%)
Impr Diag	-	0.4% (0.3-0.4%)	-
DST for all	1% (0.2-3.7%)	-	-
Short DS	0% (0-0%)	0% (0-0%)	0% (0-0%)
Short DR	1.6% (0.4-5.4%)	2.3% (2-2.9%)	1.1% (1-1.5%)
Pri Scr	17.9% (16.4-19.4%)	-	-
Nutr	-	7% (6.7-7.5%)	-

5.10 TB deaths - absolute numbers (1000s) [50% coverage]

TB deaths averted by 2050 (number relative to BAU, in thousands)			
Intervention	Brazil	India	South Africa
Vacc	82K (72-95K)	622K (573-722K)	237K (203-281K)
TPT	2K (1-4K)	174K (165-187K)	32K (26-35K)
Comm Scr	142K (127-158K)	1,746K (1,626-1,798K)	324K (292-353K)
Impr Diag	-	27K (22-30K)	-
DST for all	5K (1-17K)	-	-
Short DS	0K (0-0K)	0K (0-0K)	0K (0-0K)
Short DR	7K (2-25K)	153K (134-196K)	15K (13-21K)
Pri Scr	81K (69-95K)	-	-
Nutr	-	472K (458-503K)	-

5.11 Disability-adjusted life years - total (millions) [50% coverage]

Disability-adjusted life years (number)			
Intervention	Brazil	India	South Africa
BAU	6.5M (5.7-7.2M)	91.5M (88.2-93.4M)	14.8M (13.4-16M)
Vacc	5.4M (4.8-6M)	84M (80.8-86.1M)	12.6M (11.4-13.6M)
TPT	6.5M (5.7-7.1M)	89.5M (86.3-91.3M)	14.5M (13.1-15.7M)
Comm Scr	4.6M (4-5.1M)	70.3M (67.6-72.8M)	11.6M (10.5-12.5M)
Impr Diag	-	91.2M (87.9-93.1M)	-
DST for all	6.5M (5.7-7.1M)	-	-
Short DS	6.5M (5.7-7.2M)	91.5M (88.2-93.4M)	14.8M (13.4-16M)
Short DR	6.4M (5.7-7.1M)	90.1M (86.8-92.1M)	14.7M (13.2-15.8M)
Pri Scr	5.6M (4.9-6.2M)	-	-
Nutr	-	86.4M (83.2-88.2M)	-

5.12 Disability-adjusted life years averted compared to BAU (millions) [50% coverage]

Disability-adjusted life years averted (number relative to BAU)			
Intervention	Brazil	India	South Africa
Vacc	1M (0.9-1.1M)	7.3M (6.8-8.5M)	2.2M (1.9-2.5M)
TPT	0M (0-0.1M)	2M (1.9-2.1M)	0.3M (0.2-0.3M)
Comm Scr	1.9M (1.7-2.1M)	21.2M (20-21.7M)	3.2M (2.9-3.5M)
DST for all	0M (0-0.2M)	-	-
Short DS	0M (0-0M)	0M (0-0M)	0M (0-0M)
Short DR	0.1M (0-0.2M)	1.3M (1.2-1.7M)	0.1M (0.1-0.2M)
Pri Scr	0.9M (0.8-1M)	-	-
Nutr	-	5M (4.9-5.4M)	-

5.13 Total cost of each intervention [50% coverage]

	Budget impact (USD)		
Intervention	Brazil	India	South Africa
BAU	1.6B (1.0-2.4B)	17.5B (12.3-25.6B)	2.5B (1.7-3.6B)
Vacc	3.2B (2.2-4.3B)	28.3B (20.5-37.7B)	2.9B (2.0-4.0B)
TPT	1.6B (1.0-2.4B)	18.3B (13.1-26.5B)	2.6B (1.8-3.7B)
Comm Scr (high)	24.1B (14.7-38.3B)	168.9B (109.1-238.1B)	8.5B (6.1-11.9B)
Comm Scr (low)	5.6B (4.1-7.5B)	37.5B (28.4-48.8B)	3.7B (2.8-4.9B)
Impr Diag	-	25.2B (19.0-34.3B)	-
DST for all	1.7B (1.0-2.6B)	-	-
Short DS	1.6B (1.1-2.5B)	18.5B (13.0-26.7B)	2.6B (1.7-3.6B)
Short DR	1.4B (0.9-2.1B)	17.3B (12.1-25.4B)	2.5B (1.6-3.6B)
Pri Scr	1.7B (1.1-2.6B)	-	-
Nutr	-	18.9B (13.7-27.2B)	-

5.14 Incremental cost of each intervention compared to BAU [50% coverage]

	Incremental cost (USD relative to BAU)		
Intervention	Brazil	India	South Africa
Vacc	1.5B (0.8-2.5B)	10.6B (6.0-17.1B)	0.4B (0.2-0.7B)
TPT	0.0B (0.0-0.0B)	0.8B (0.5-1.1B)	0.1B (0.1-0.1B)
Comm Scr (high)	22.4B (13.0-36.8B)	151.0B (91.9-221.2B)	5.9B (3.7-9.3B)
Comm Scr (low)	4.0B (2.8-5.7B)	19.4B (13.4-26.8B)	1.2B (0.9-1.7B)
Impr Diag	-	7.4B (1.2-16.4B)	-
DST for all	0.1B (0.0-0.3B)	-	-
Short DS	0.1B (0.0-0.1B)	0.9B (-0.7-2.6B)	0.1B (-0.1-0.3B)
Short DR	-0.2B (-0.6-0.0B)	-0.2B (-0.4-0.0B)	0.0B (-0.1-0.0B)
Pri Scr	0.1B (0.1-0.2B)	-	-
Nutr	-	1.4B (0.9-2.0B)	-

5.15 Cost–utility analysis by country [50% coverage]

ICERs are calculated as described at 3.3 above.

5.15.1 Brazil [50% coverage]

**Intervention**	**Mean total cost** (billion R$)	**Mean total cost** (billion US$)	**Incremental cost compared to BAU** (million US$)	**Mean total DALYs** (million)	**DALYs averted compared to BAU** (million)	**ICER compared to BAU** (US$ per DALY averted)	**Probability cost-effective at lower end of threshold range** (US$6,968 per DALY)	**Probability cost-effective at upper end of threshold range** (US$10,735 per DALY)
Business as usual	8.04	1.61	-	6.49	-	-	-	-
Vaccination	15.84	3.17	1,564	5.44	1.05	1,496	100%	100%
TB preventive treatment	8.20	1.64	32	6.45	0.04	797	100%	100%
Community screening (high-cost)	123.42	24.73	23,122	4.61	1.88	12,274	3%	34%
Community screening (low-cost)	28.48	5.71	4,097	4.61	1.88	2,175	100%	100%
Improved diagnosis	NA	NA	NA	NA	NA	NA	NA	NA
Drug	8.57	1.72	106	6.43	0.06	1,888	100%	100%

susceptibility testing for all								
Shorter DS-TB treatment	8.35	1.67	61	6.49	0	Dominated by BAU	0%	0%
Shorter DR-TB treatment	6.98	1.40	−213	6.42	0.07	Dominates BAU	100%	100%
Screening in prisons	8.68	1.74	128	5.58	0.91	141	100%	100%

5.15.2 India [50% coverage]

**Intervention**	**Mean total cost** (trillion INR)	**Mean total cost** (billion US$)	**Incremental cost compared to BAU** (billion US$)	**Mean total DALYs** (million)	**DALYs averted compared to BAU** (million)	**ICER compared to BAU** (US$ per DALY averted)	**Probability cost-effective at lower end of threshold range** (US$413 per DALY)	**Probability cost-effective at upper end of threshold range** (US$569 per DALY)
Business as usual	1.48	17.92	-	91.2	-	-	-	-
Vaccination	2.37	28.63	10.71	83.8	7.4	1,440	0%	0%
TB preventive treatment	1.55	18.70	0.78	89.2	2.0	397	61%	97%
Community screening (high-cost)	14.12	170.57	152.66	70.1	21.1	7,249	0%	0%
Community screening (low-cost)	3.10	37.46	19.54	70.1	21.1	928	0%	1%
Improved diagnosis	2.13	25.68	7.76	91.0	0.3	30,974	1%	1%
Drug susceptibility testing for all	NA	NA	NA	NA	NA	NA	NA	NA

Shorter DS-TB treatment	1.56	18.82	0.90	91.2	0	Dominated by BAU	0%	0%
Shorter DR-TB treatment	1.47	17.72	−0.20	89.8	1.4	Dominates BAU	100%	100%
Nutrition	1.60	19.33	1.42	86.1	5.1	280	99%	100%

5.15.3 South Africa [50% coverage]

**Intervention**	**Mean total cost** (billion R)	**Mean total cost** (billion US$)	**Incremental cost compared to BAU** (million US$)	**Mean total DALYs** (million)	**DALYs averted compared to BAU** (million)	**ICER compared to BAU** (US$ per DALY averted)	**Probability cost-effective at lower end of threshold range** (US$2,444 per DALY)	**Probability cost-effective at upper end of threshold range** (US$3,285 per DALY)
Business as usual	47.22	2.56	-	14.76	-		-	-
Vaccination	54.42	2.95	391	12.59	2.17	180	100%	100%
TB preventive treatment	49.09	2.66	102	14.48	0.28	361	100%	100%
Community screening (high-cost)	159.74	8.66	6,099	11.56	3.21	1,903	88%	100%
Community screening (low-cost)	69.48	3.77	1,207	11.56	3.21	376	100%	100%
Improved diagnosis	NA	NA	NA	NA	NA	NA	NA	NA
Drug susceptibility testing for all	NA	NA	NA	NA	NA	NA	NA	NA

Shorter DS-TB treatment	48.22	2.61	54	14.76	0	Dominated by BAU	0%	0%
Shorter DR-TB treatment	46.78	2.54	−24	14.64	0.12	Dominates BAU	100%	100%

5.16 Cost-effectiveness planes for all interventions (including high-cost community-wide screening) [50% coverage]

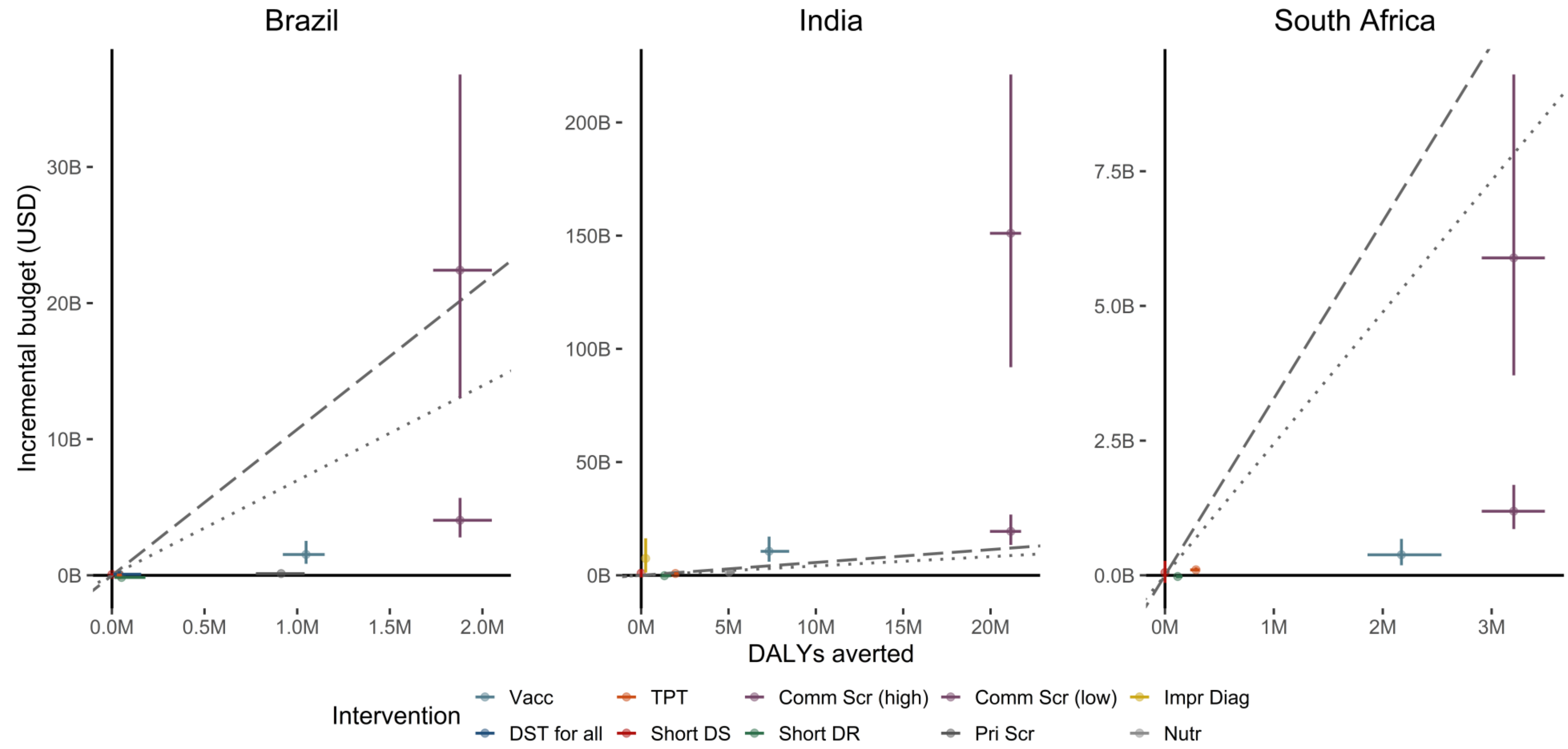

Points show median model estimates with extending lines indicating 95% uncertainty intervals across 500 calibrated parameter sets. The dashed line shows the upper bound and the dotted line the lower bound of the estimated cost-effectiveness threshold range for each country.

5.17 Cost-effectiveness planes for all interventions (excluding high-cost community-wide screening) [50% coverage]

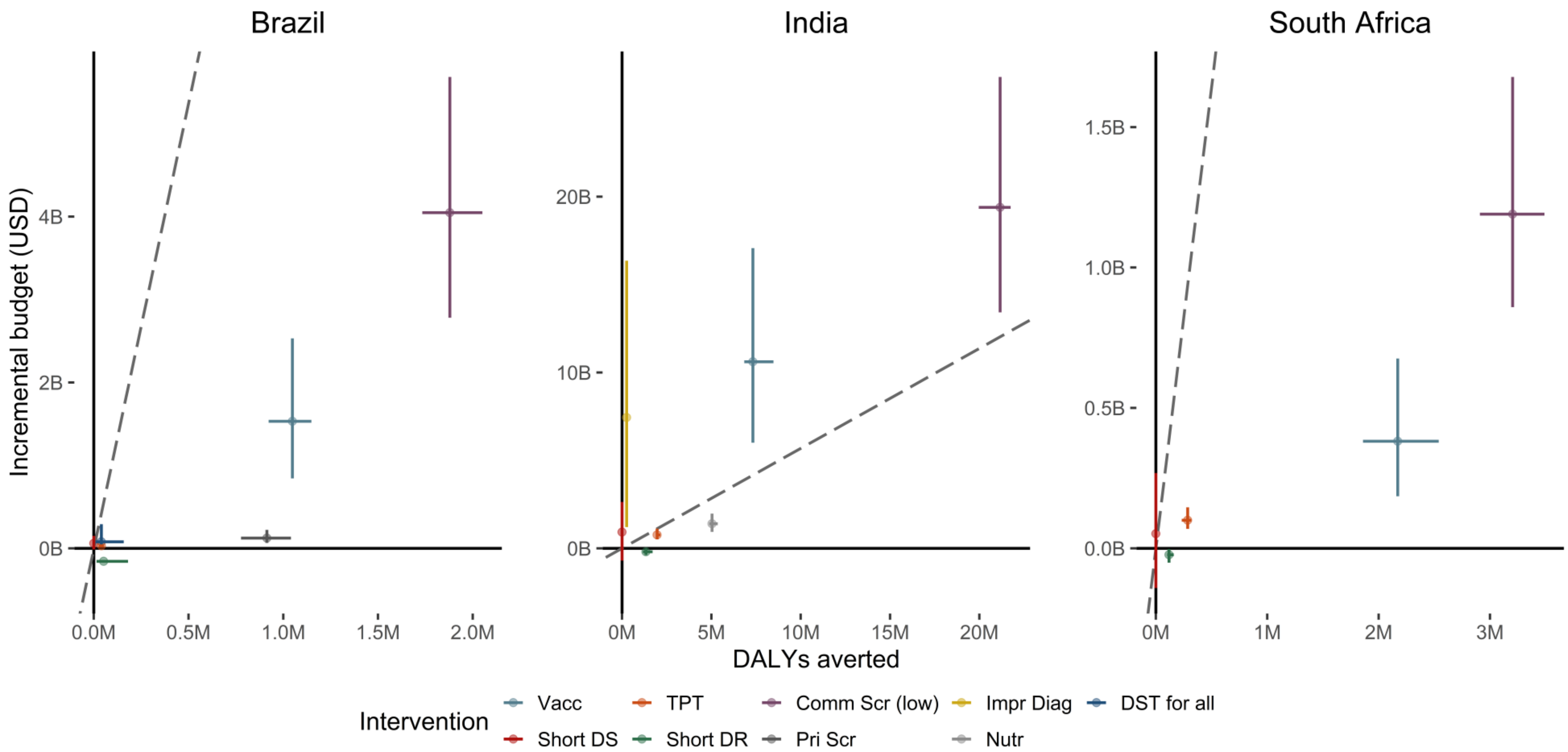

Points show median model estimates with extending lines indicating 95% uncertainty intervals across 500 calibrated parameter sets. The dashed line shows the upper bound of the estimated cost-effectiveness threshold range for each country.

6 SENSITIVITY ANALYSIS: 20% COVERAGE

Sensitivity analyses assuming 20% coverage (instead of 80% coverage as in the primary analysis) were run for all interventions in each country, except where baseline implementation already exceeds 20% coverage (i.e., “Impr Diag” and “DST for all” in all three countries).

6.1 TB prevalence trends [20% coverage]

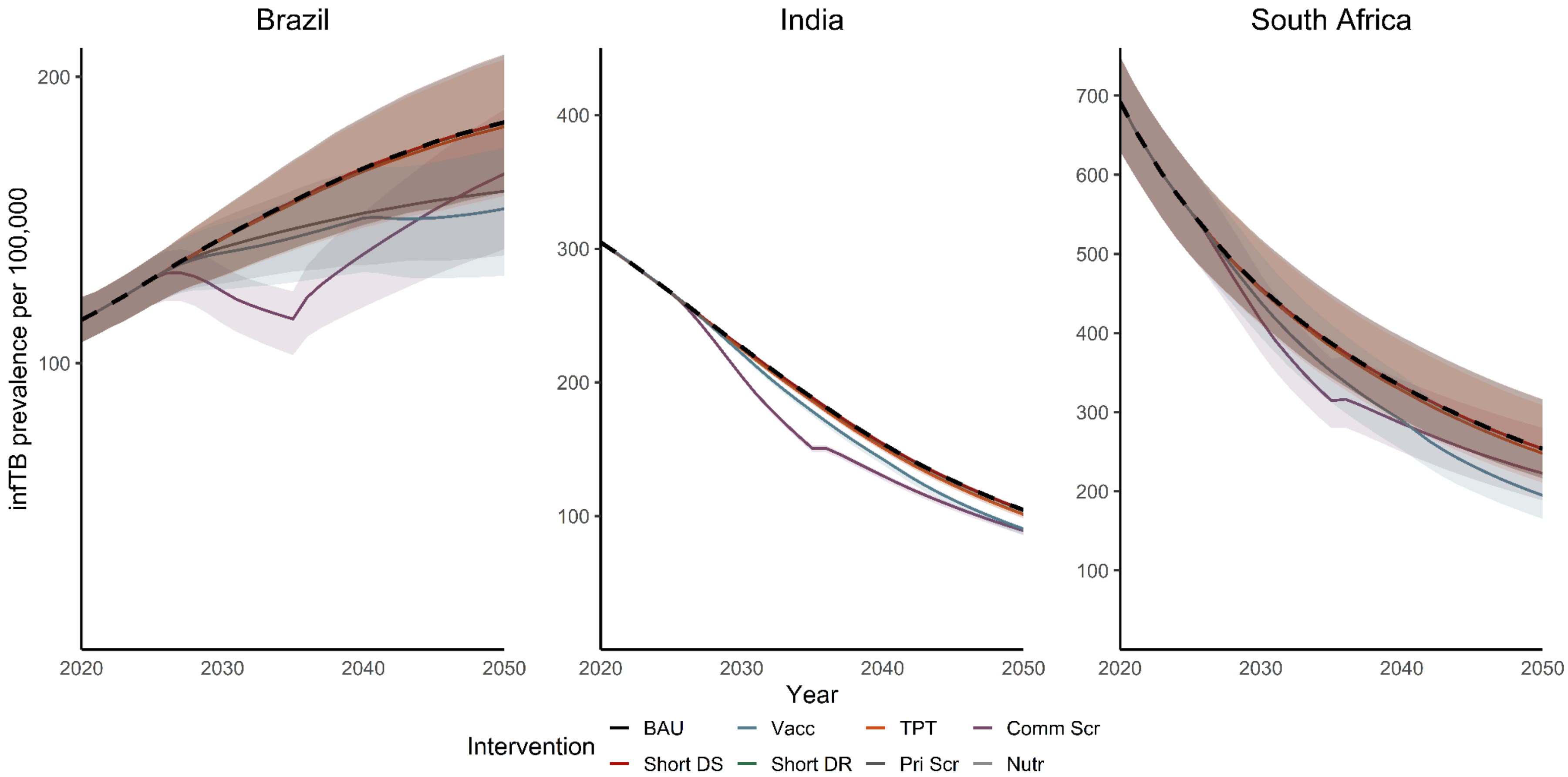

Lines show median model estimates; shaded areas show 95% uncertainty intervals across 500 calibrated parameter sets.

6.2 TB mortality trends [20% coverage]

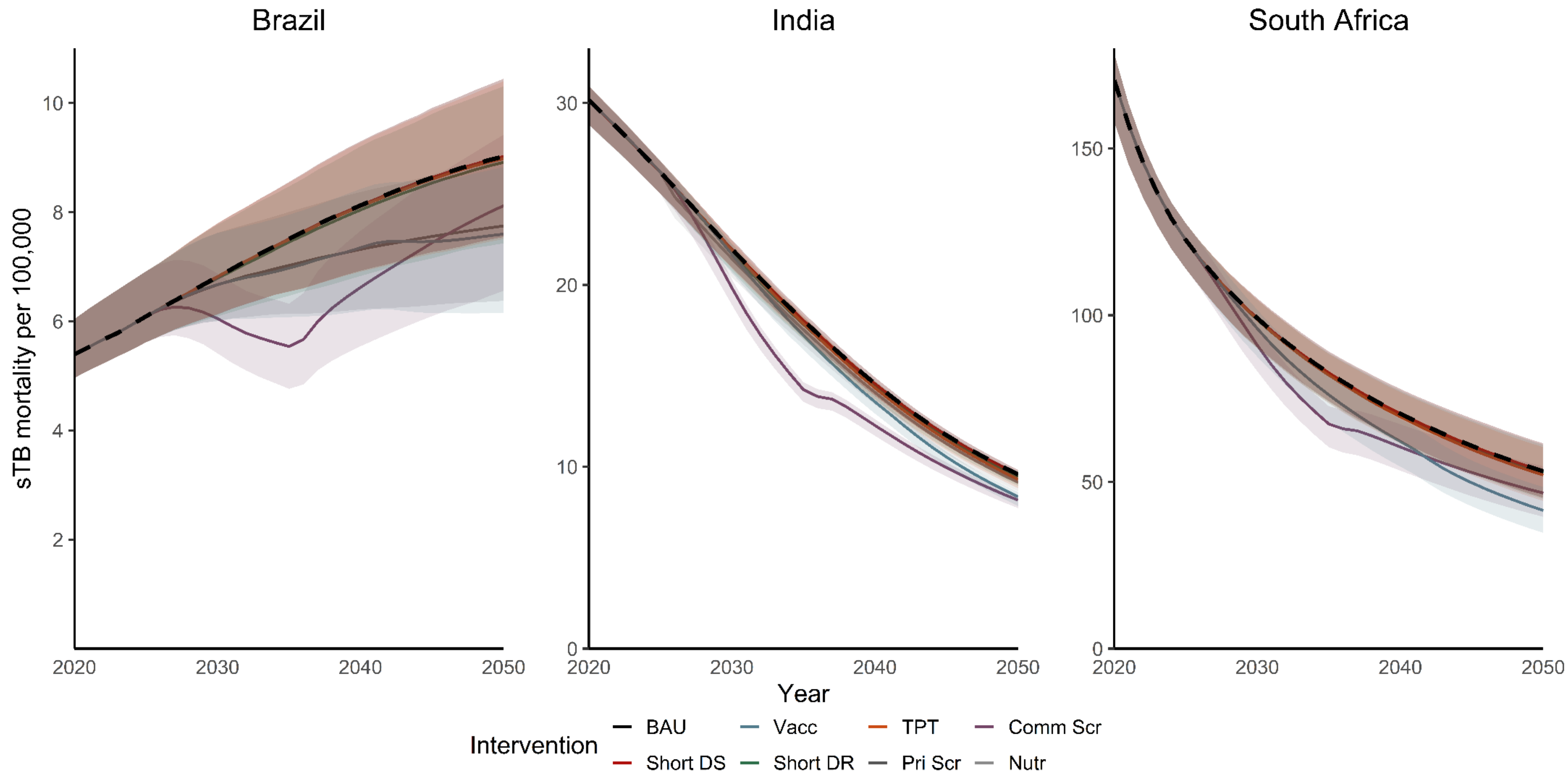

Lines show median model estimates; shaded areas show 95% uncertainty intervals across 500 calibrated parameter sets.

6.3 Incident sTB episodes averted - proportion of total (graph) [20% coverage]

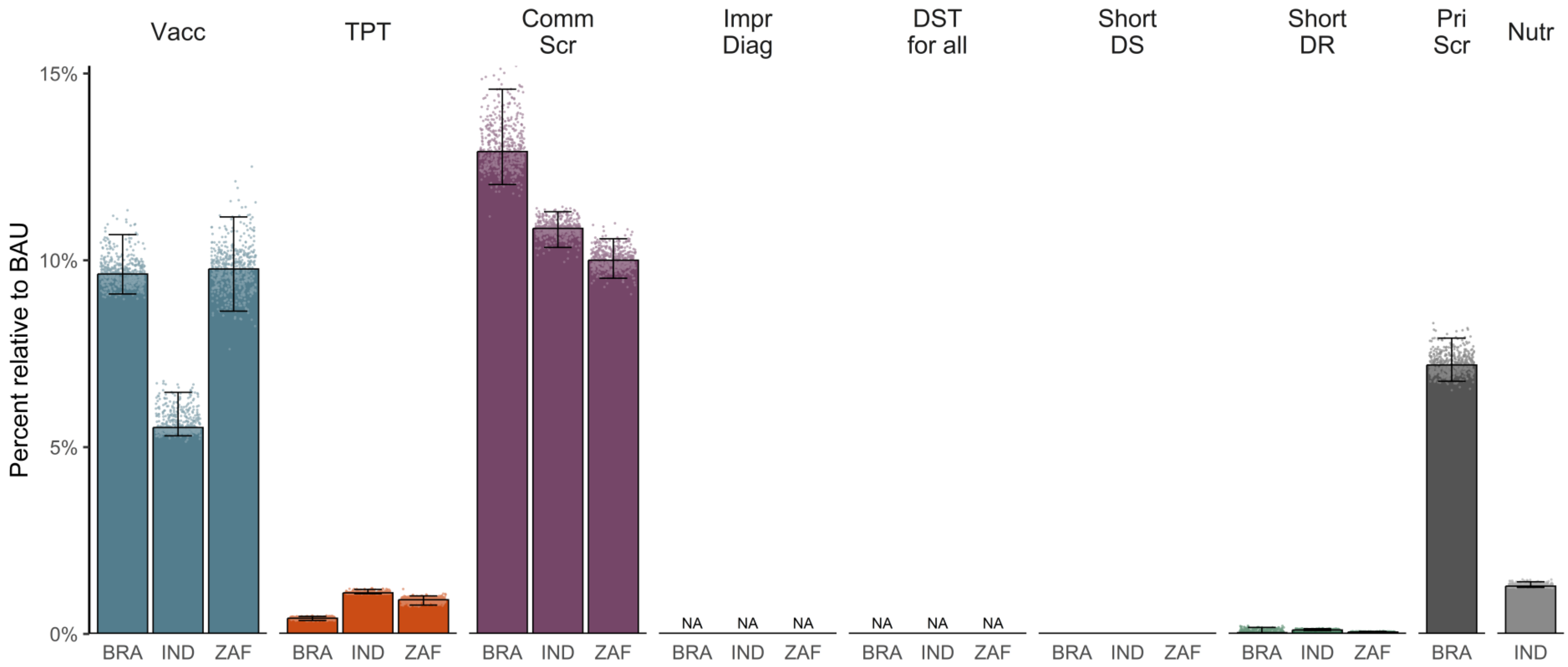

Bars show median model estimates; error bars show 95% uncertainty intervals across 500 calibrated parameter sets; points show model estimates for each calibrated parameter set.

6.4 Incident sTB episodes averted - proportion of total (table) [20% coverage]

Incident symptomatic TB episodes averted by 2050 (percent relative to BAU)			
Intervention	Brazil	India	South Africa
Vacc	9.6% (9.1-10.7%)	5.5% (5.3-6.5%)	9.8% (8.6-11.2%)
TPT	0.4% (0.4-0.5%)	1.1% (1.1-1.2%)	0.9% (0.8-1%)
Comm Scr	12.9% (12-14.6%)	10.9% (10.3-11.3%)	10% (9.5-10.6%)
Impr Diag	-	-	-
DST for all	-	-	-
Short DS	0% (0-0%)	0% (0-0%)	0% (0-0%)
Short DR	0% (0-0.2%)	0.1% (0.1-0.1%)	0.1% (0-0.1%)
Pri Scr	7.2% (6.8-7.9%)	-	-
Nutr	-	1.3% (1.2-1.4%)	-

6.5 Incident sTB episodes averted - absolute numbers (1000s) [20% coverage]

Incident symptomatic TB episodes averted by 2050 (number relative to BAU, in thousands)			
Intervention	Brazil	India	South Africa
Vacc	423K (376-463K)	2,247K (2,129-2,603K)	435K (371-523K)
TPT	18K (15-21K)	446K (437-486K)	41K (34-46K)
Comm Scr	567K (518-630K)	4,397K (4,213-4,542K)	447K (403-501K)
Impr Diag	-	-	-
DST for all	-	-	-
Short DS	0K (0-0K)	0K (0-0K)	0K (0-0K)
Short DR	1K (0-7K)	43K (36-56K)	2K (2-3K)
Pri Scr	315K (275-357K)	-	-
Nutr	-	517K (506-570K)	-

6.6 Reduction in TB prevalence in 2050 - proportional (graph) [20% coverage]

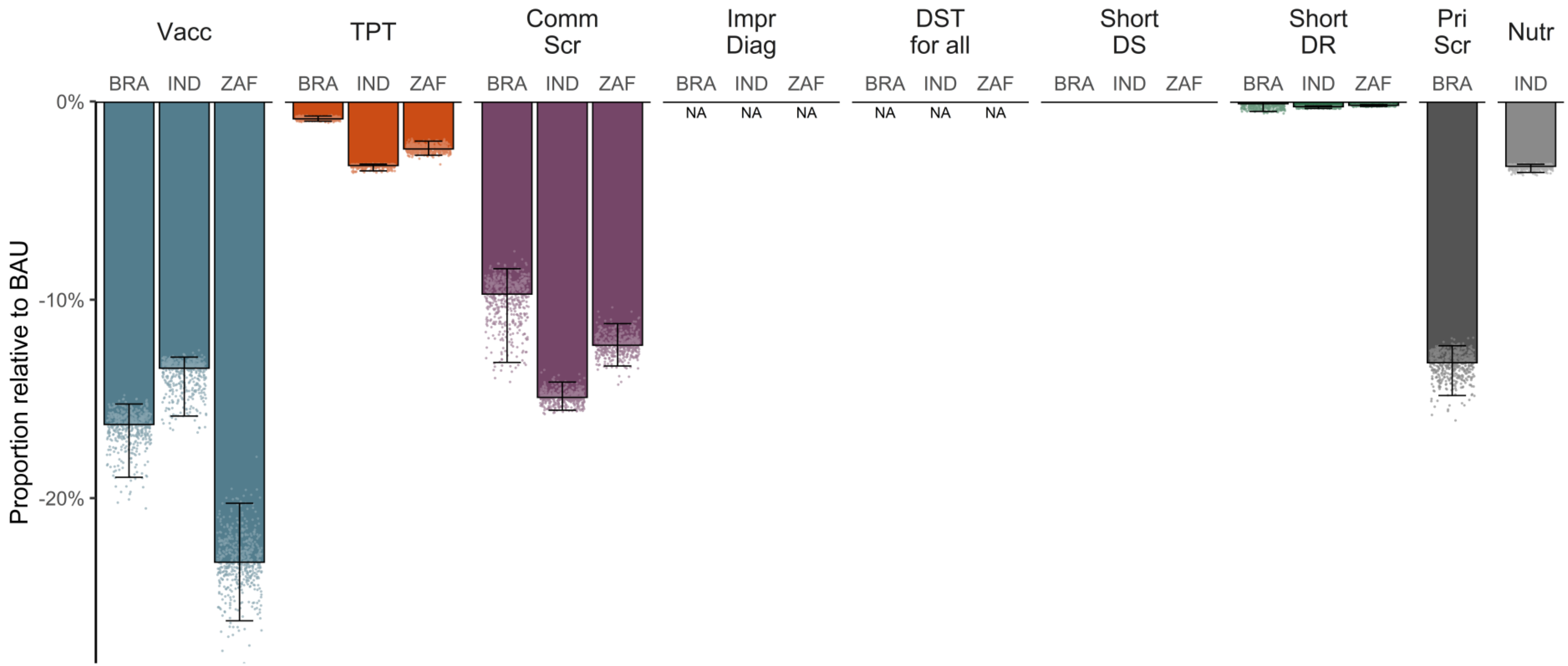

Bars show median model estimates; error bars show 95% uncertainty intervals across 500 calibrated parameter sets; points show model estimates for each calibrated parameter set.

6.7 Reduction in TB prevalence in 2050 - proportional (table) [20% coverage]

Decline in infectious TB prevalence in 2050 (percent relative to BAU)			
Intervention	Brazil	India	South Africa
Vacc	16.3% (15.3-19%)	13.4% (12.9-15.9%)	23.2% (20.3-26.2%)
TPT	0.9% (0.7-1%)	3.2% (3.2-3.5%)	2.4% (2-2.7%)
Comm Scr	9.7% (8.4-13.2%)	14.9% (14.2-15.6%)	12.3% (11.2-13.3%)
Impr Diag	-	-	-
DST for all	-	-	-
Short DS	0% (0-0%)	0% (0-0%)	0% (0-0%)
Short DR	0.1% (0-0.5%)	0.3% (0.2-0.4%)	0.2% (0.2-0.3%)
Pri Scr	13.2% (12.3-14.8%)	-	-
Nutr	-	3.3% (3.2-3.6%)	-

6.8 TB deaths averted - proportion of total (graph) [20% coverage]

Bars show median model estimates; error bars show 95% uncertainty intervals across 500 calibrated parameter sets; points show model estimates for each calibrated parameter set.

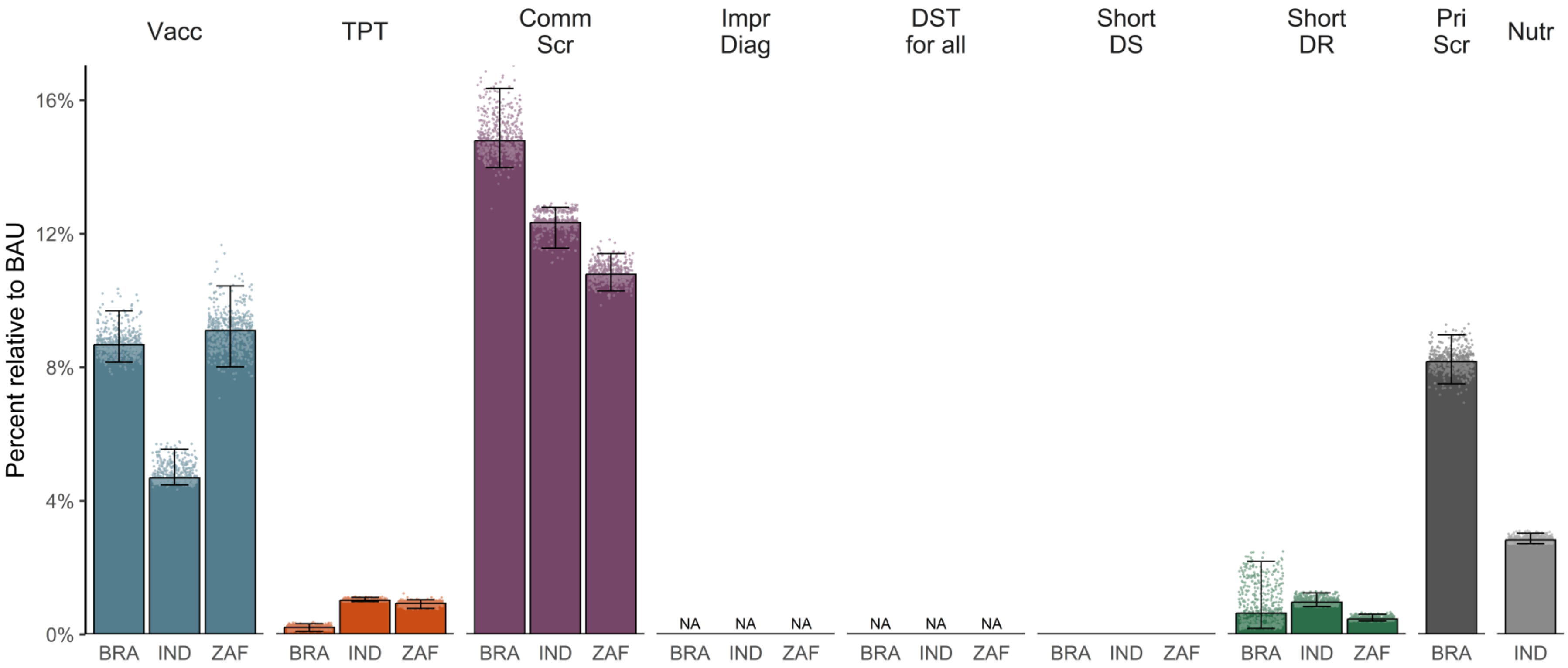

6.9 TB deaths averted - proportion of total [20% coverage]

TB deaths averted by 2050 (percent relative to BAU)			
Intervention	Brazil	India	South Africa
Vacc	8.7% (8.2-9.7%)	4.7% (4.5-5.5%)	9.1% (8-10.4%)
TPT	0.2% (0.1-0.3%)	1% (1-1.1%)	0.9% (0.8-1%)
Comm Scr	14.8% (14-16.4%)	12.3% (11.6-12.8%)	10.8% (10.3-11.4%)
Impr Diag	-	-	-
DST for all	-	-	-
Short DS	0% (0-0%)	0% (0-0%)	0% (0-0%)
Short DR	0.6% (0.2-2.2%)	1% (0.8-1.2%)	0.5% (0.4-0.6%)
Pri Scr	8.2% (7.5-9%)	-	-
Nutr	-	2.8% (2.7-3%)	-

6.10 TB deaths - absolute numbers (1000s) [20% coverage]

TB deaths averted by 2050 (number relative to BAU, in thousands)			
Intervention	Brazil	India	South Africa
Vacc	40K (35-46K)	319K (293-373K)	125K (106-149K)
TPT	1K (0-2K)	69K (66-75K)	13K (10-14K)
Comm Scr	68K (61-76K)	837K (775-865K)	149K (135-163K)
Impr Diag	-	-	-
DST for all	-	-	-
Short DS	0K (0-0K)	0K (0-0K)	0K (0-0K)
Short DR	3K (1-10K)	65K (57-83K)	6K (5-9K)
Pri Scr	37K (32-44K)	-	-
Nutr	-	191K (185-203K)	-

6.11 Disability-adjusted life years - total (millions) [20% coverage]

Disability-adjusted life years (number)			
Intervention	Brazil	India	South Africa
BAU	6.5M (5.7-7.2M)	91.5M (88.2-93.4M)	14.8M (13.4-16M)
Vacc	6M (5.3-6.6M)	87.7M (84.5-89.7M)	13.7M (12.4-14.7M)
TPT	6.5M (5.7-7.2M)	90.7M (87.4-92.5M)	14.7M (13.3-15.9M)
Comm Scr	5.6M (4.9-6.2M)	81.4M (78.4-83.5M)	13.3M (12-14.4M)
Impr Diag	-	-	-
DST for all	-	-	-
Short DS	6.5M (5.7-7.2M)	91.5M (88.2-93.4M)	14.8M (13.4-16M)
Short DR	6.5M (5.7-7.2M)	90.9M (87.6-92.8M)	14.7M (13.3-15.9M)
Pri Scr	6.1M (5.4-6.7M)	-	-
Nutr	-	89.4M (86.2-91.3M)	-

6.12 Disability-adjusted life years averted compared to BAU (millions) [20% coverage]

Disability-adjusted life years averted (number relative to BAU)			
Intervention	Brazil	India	South Africa
Vacc	0.5M (0.4-0.6M)	3.7M (3.4-4.3M)	1.1M (1-1.3M)
TPT	0M (0-0M)	0.8M (0.7-0.8M)	0.1M (0.1-0.1M)
Comm Scr	0.9M (0.8-1M)	10.1M (9.4-10.4M)	1.5M (1.3-1.6M)
DST for all	-	-	-
Short DS	0M (0-0M)	0M (0-0M)	0M (0-0M)
Short DR	0M (0-0.1M)	0.6M (0.5-0.7M)	0M (0-0.1M)
Pri Scr	0.4M (0.4-0.5M)	-	-
Nutr	-	2M (2-2.2M)	-

6.13 Total cost of each intervention [20% coverage]

	Budget impact (USD)		
Intervention	Brazil	India	South Africa
BAU	1.6B (1.0-2.4B)	17.5B (12.3-25.6B)	2.5B (1.7-3.6B)
Vacc	2.3B (1.6-3.2B)	22.6B (16.3-31.2B)	2.7B (1.8-3.7B)
TPT	1.6B (1.0-2.4B)	17.8B (12.6-26.0B)	2.5B (1.7-3.6B)
Comm Scr (high)	10.6B (6.8-16.3B)	78.6B (53.6-106.6B)	4.9B (3.7-6.5B)
Comm Scr (low)	3.2B (2.4-4.3B)	25.3B (19.3-34.2B)	3.0B (2.1-4.0B)
Impr Diag	-	-	-
DST for all	-	-	-
Short DS	1.6B (1.0-2.4B)	17.9B (12.9-26.0B)	2.5B (1.7-3.6B)
Short DR	1.5B (1.0-2.2B)	17.5B (12.2-25.5B)	2.5B (1.7-3.6B)
Pri Scr	1.6B (1.0-2.5B)	-	-
Nutr	-	18.1B (13.0-26.3B)	-

6.14 Incremental cost of each intervention compared to BAU [20% coverage]

	Incremental cost (USD relative to BAU)		
Intervention	Brazil	India	South Africa
Vacc	0.7B (0.4-1.1B)	4.9B (2.8-7.9B)	0.2B (0.1-0.3B)
TPT	0.0B (0.0-0.0B)	0.3B (0.2-0.5B)	0.0B (0.0-0.1B)
Comm Scr (high)	9.0B (5.2-14.7B)	60.5B (36.8-88.5B)	2.4B (1.5-3.7B)
Comm Scr (low)	1.6B (1.1-2.3B)	7.8B (5.4-10.8B)	0.5B (0.3-0.7B)
Impr Diag	-	-	-
DST for all	-	-	-
Short DS	0.0B (0.0-0.1B)	0.4B (-0.3-1.1B)	0.0B (-0.1-0.1B)
Short DR	-0.1B (-0.3-0.0B)	-0.1B (-0.2-0.0B)	0.0B (0.0-0.0B)
Pri Scr	0.1B (0.0-0.1B)	-	-
Nutr	-	0.6B (0.4-0.8B)	-

6.15 Cost–utility analysis by country [20% coverage]

ICERs are calculated as described at 3.3 above.

6.15.1 Brazil [20% coverage]

**Intervention**	**Mean total cost** (billion R$)	**Mean total cost** (billion US$)	**Incremental cost compared to BAU** (million US$)	**Mean total DALYs** (million)	**DALYs averted compared to BAU** (million)	**ICER compared to BAU** (US$ per DALY averted)	**Probability cost-effective at lower end of threshold range** (US$6,968 per DALY)	**Probability cost-effective at upper end of threshold range** (US$10,735 per DALY)
Business as usual	8.04	1.61	-	6.49	-	-	-	-
Vaccination	11.56	2.32	705	5.99	0.50	1,406	100%	100%
TB preventive treatment	8.10	1.62	13	6.47	0.02	797	100%	100%
Community screening (high-cost)	54.32	10.89	9,275	5.59	0.90	10,294	9%	61%
Community screening (low-cost)	16.34	3.27	1,663	5.59	0.90	1,846	100%	100%
Improved diagnosis	NA	NA	NA	NA	NA	NA	NA	NA
Drug	NA	NA	NA	NA	NA	NA	NA	NA

susceptibility testing for all								
Shorter DS-TB treatment	8.16	1.64	25	6.49	0	Dominated by BAU	0%	0%
Shorter DR-TB treatment	7.61	1.52	−86	6.46	0.03	Dominates BAU	100%	100%
Screening in prisons	8.33	1.67	58	6.08	0.41	142	100%	100%

6.15.2 India [20% coverage]

**Intervention**	**Mean total cost** (trillion INR)	**Mean total cost** (billion US$)	**Incremental cost compared to BAU** (billion US$)	**Mean total DALYs** (million)	**DALYs averted compared to BAU** (million)	**ICER compared to BAU** (US$ per DALY averted)	**Probability cost-effective at lower end of threshold range** (US$413 per DALY)	**Probability cost-effective at upper end of threshold range** (US$569 per DALY)
Business as usual	1.48	17.92	-	91.2	-	-	-	-
Vaccination	1.89	22.89	4.97	87.4	3.8	1,323	0%	1%
TB preventive treatment	1.51	18.24	0.32	90.4	0.8	411	55%	97%
Community screening (high-cost)	6.54	79.03	61.11	81.2	10.0	6,107	0%	0%
Community screening (low-cost)	2.13	25.76	7.85	81.2	10.0	784	0%	6%
Improved diagnosis	NA	NA	NA	NA	NA	NA	NA	NA
Drug susceptibility testing for all	NA	NA	NA	NA	NA	NA	NA	NA

Shorter DS-TB treatment	1.51	18.28	0.36	91.2	0	Dominated by BAU	0%	0%
Shorter DR-TB treatment	1.48	17.83	−0.08	90.6	0.6	Dominates BAU	100%	100%
Nutrition	1.53	18.48	0.57	89.2	2.0	279	99%	100%

6.15.3 South Africa [20% coverage]

**Intervention**	**Mean total cost** (billion R)	**Mean total cost** (billion US$)	**Incremental cost compared to BAU** (million US$)	**Mean total DALYs** (million)	**DALYs averted compared to BAU** (million)	**ICER compared to BAU** (US$ per DALY averted)	**Probability cost-effective at lower end of threshold range** (US$2,444 per DALY)	**Probability cost-effective at upper end of threshold range** (US$3,285 per DALY)
Business as usual	47.22	2.56	-	14.76	-		-	-
Vaccination	50.57	2.74	182	13.64	1.12	162	100%	100%
TB preventive treatment	47.98	2.60	41	14.65	0.11	360	100%	100%
Community screening (high-cost)	92.26	5.00	2,441	13.29	1.47	1,657	95%	100%
Community screening (low-cost)	56.14	3.04	483	13.29	1.47	328	100%	100%
Improved diagnosis	NA	NA	NA	NA	NA	NA	NA	NA
Drug susceptibility testing for all	NA	NA	NA	NA	NA	NA	NA	NA

Shorter DS-TB treatment	47.62	2.58	22	14.76	0	Dominated by BAU	0%	0%
Shorter DR-TB treatment	47.04	2.55	−10	14.71	0.05	Dominates BAU	100%	100%

6.16 Cost-effectiveness planes for all interventions (including high-cost community-wide screening) [20% coverage]

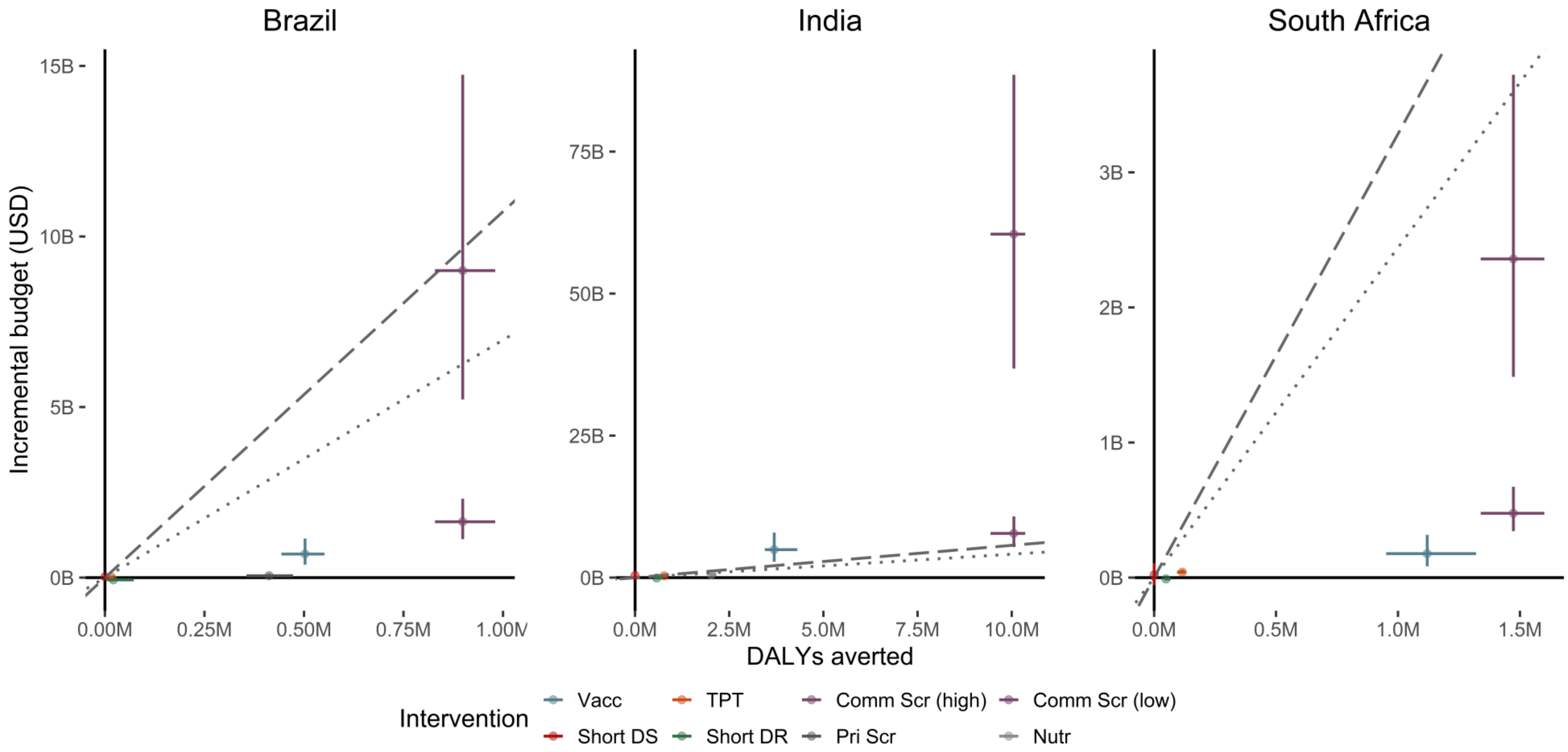

Points show median model estimates with extending lines indicating 95% uncertainty intervals across 500 calibrated parameter sets. The dashed line shows the upper bound and the dotted line the lower bound of the estimated cost-effectiveness threshold range for each country.

6.17 Cost-effectiveness planes for all interventions (excluding high-cost community-wide screening) [20% coverage]

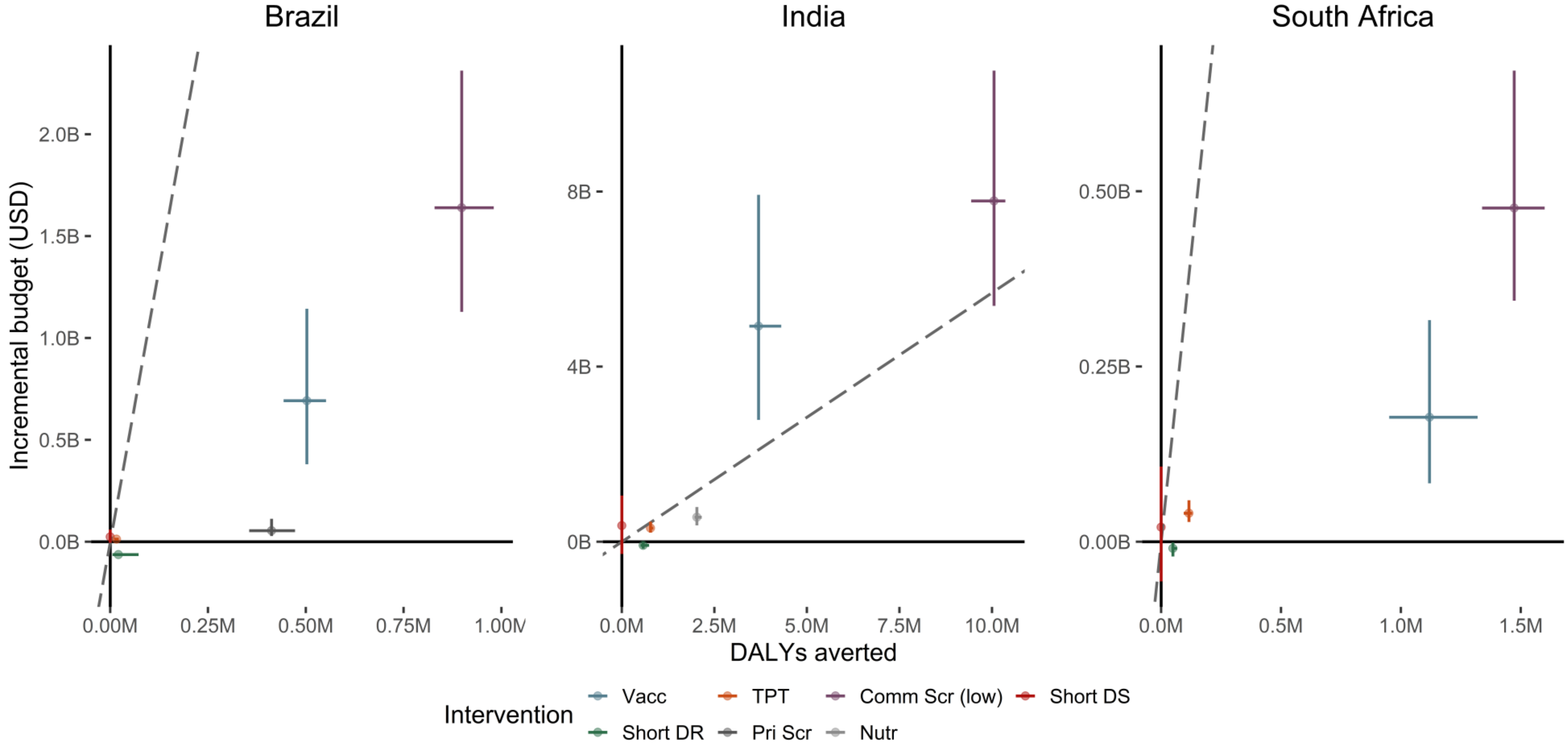

Points show median model estimates with extending lines indicating 95% uncertainty intervals across 500 calibrated parameter sets. The dashed line shows the upper bound of the estimated cost-effectiveness threshold range for each country.
