# Supplementary material for: Global impact and cost-effectiveness of tuberculosis interventions
Source: Nat Health. 2026 Jul 21;1(11):1320–30. doi: 10.1038/s44360-026-00171-5 (PMC13727693; doi:10.1038/s44360-026-00171-5)
Supplement: Supplementary file 3 [file 44360_2026_171_MOESM3_ESM.pdf]

nature portfolio

Peer Review File

Epidemiological impact and cost-effectiveness of tuberculosis interventions: a modelling study in Brazil, India, and South Africa

Corresponding Author: Professor Rein Houben

This manuscript has been previously reviewed at another journal. This document only contains information relating to versions considered at Nature Health.

This file contains all reviewer reports in order by version, followed by all author rebuttals in order by version.

Version 0:

Reviewer comments:

Reviewer #1

(Remarks to the Author)
Thank you for allowing me to re-review this paper after initial revision by the authors. The authors have clarified or addressed most of the concerns raised in the initial review.

However, I still find the author's use of 80% coverage--without performing any sensitivity analyses at all--to be problematic. I completely understand the authors' approach of trying to put "coverage" for all interventions on a level playing field, and to take an optimistic view of what is achievable in terms of coverage for the purposes of the modeling exercise. This allows comparison across interventions and also provides motivation to TB programs and governments to improve coverage and implementation of interventions that are deemed to have the greatest potential theoretical public health impact.

But even a single alternative analysis using a different coverage level for all interventions (say 60%) would be incredibly informative--in part, because it may be more feasible to achieve higher coverage for some interventions as compared to others, and this could be informative to TB programs that consume this article for decision making. Even if these findings are placed in the supplementary appendix and mentioned efficiently in the main manuscript (e.g., using a figure similar to the one for 80%) coverage, this might be a valuable contribution.

I should note that Reviewer #2 also raised this as one of their concerns / recommendations in the initial review, and I found this comment by Reviewer #2 to be helpful and highlight it for the authors:

"The robustness of this paper's findings and the translatability to policy decisions would be strengthened if alternative coverage targets could be considered. For example, if uptake is 50%, would similar patterns of relative effectiveness of interventions be found, or would the impact of
some interventions be reduced to a greater degree than others? Additionally, some interventions (e.g., shorter treatment regimens) might more readily achieve the target levels than others (e.g., vaccination or screening)."

As to whether this should be a mandatory additional analysis or not, I leave to the discretion of the journal editors.

(Remarks on code availability)

Reviewer #3

(Remarks to the Author)
Thank you for addressing the comments succinctly and sufficiently.

(Remarks on code availability)

Reviewer #4

(Remarks to the Author)
I appreciate the opportunity to review the authors' thoughtful responses to the comments. The authors have added clarity around many of the issues identified by the other reviewers and me. There are two issues that warrant further consideration for this manuscript to attain its maximum potential to inform policy and decision making. Each of these issues was identified by me and at least one other reviewer.

First, the author's assumption of 80% coverage across all interventions limits interpretability for decision makers, who need to determine how much effort and resources to commit to each intervention. As the authors note, multiple interventions could be adopted in parallel, but it is unlikely that each intervention would attain the identical 80% coverage (and then why not just assume 100% coverage across all interventions?). Analyzing different coverage levels of interventions (at least the particularly impactful ones like screening and vaccination) would enable decision makers to see the relative epidemiologic, clinical, and budget impact and cost-effectiveness of coverage combinations like 80% screening/40% vaccination versus 40% screening/80% vaccination versus 60% screening/60% vaccination. It is not obvious that changing the coverage level would be a simple linear scaling up/down in the model. Adding these comparisons would help inform decision makers how to allocate limited resources. The authors seem to have done most of the hard work in completing this methodologically robust and important analysis, and it would be a shame if different coverage levels were not added; these additions seem less complex than what they have already done.

Second, given the importance of TPT and of HIV in this analysis (especially in South Africa for HIV), the authors could provide an analysis of improved uptake and completion of TPT among people with HIV, or a justification within the manuscript for why such an analysis was not pursued given its prioritization by WHO. There are some data around TPT uptake among people with HIV:
- WHO Global Tuberculosis Report 2023: 49% of people with HIV in South Africa who started TPT completed TPT (see Section 3.3 and Figure 3.9, https://www.who.int/teams/global-programme-on-tuberculosis-and-lung-health/tb-reports/global-tuberculosis-report-2023/tb-prevention).
- WHO Global Tuberculosis Report 2025: TPT coverage among people with HIV globally was 58%. In Africa, among those newly initiated ART, TPT coverage was 63% (Section 3.3, https://www.who.int/teams/global-programme-on-tuberculosis-and-lung-health/tb-reports/global-tuberculosis-report-2025/tb-prevention-and-screening).

(Remarks on code availability)

Open Access This Peer Review File is licensed under a Creative Commons Attribution 4.0 International License, which permits use, sharing, adaptation, distribution and reproduction in any medium or format, as long as you give appropriate credit to the original author(s) and the source, provide a link to the Creative Commons license, and indicate if changes were made.
In cases where reviewers are anonymous, credit should be given to 'Anonymous Referee' and the source.
The images or other third party material in this Peer Review File are included in the article's Creative Commons license,

unless indicated otherwise in a credit line to the material. If material is not included in the article's Creative Commons license and your intended use is not permitted by statutory regulation or exceeds the permitted use, you will need to obtain permission directly from the copyright holder.
To view a copy of this license, visit https://creativecommons.org/licenses/by/4.0/

Reviewers' Comments:

Reviewer #1 (Remarks to the Author):

Thank you for allowing me to re-review this paper after initial revision by the authors. The authors have clarified or addressed most of the concerns raised in the initial review.

We appreciate the reviewer's time and consideration to re-review our manuscript and are pleased that we have addressed most of the reviewer's previous concerns.

However, I still find the author's use of 80% coverage--without performing any sensitivity analyses at all--to be problematic. I completely understand the authors' approach of trying to put "coverage" for all interventions on a level playing field, and to take an optimistic view of what is achievable in terms of coverage for the purposes of the modeling exercise. This allows comparison across interventions and also provides motivation to TB programs and governments to improve coverage and implementation of interventions that are deemed to have the greatest potential theoretical public health impact.

But even a single alternative analysis using a different coverage level for all interventions (say 60%) would be incredibly informative--in part, because it may be more feasible to achieve higher coverage for some interventions as compared to others, and this could be informative to TB programs that consume this article for decision making. Even if these findings are placed in the supplementary appendix and mentioned efficiently in the main manuscript (e.g., using a figure similar to the one for 80%) coverage, this might be a valuable contribution.

I should note that Reviewer #2 also raised this as one of their concerns / recommendations in the initial review, and I found this comment by Reviewer #2 to be helpful and highlight it for the authors:

"The robustness of this paper's findings and the translatability to policy decisions would be strengthened if alternative coverage targets could be considered. For example, if uptake is 50%, would similar patterns of relative effectiveness of interventions be found, or would the impact of some interventions be reduced to a greater degree than others? Additionally, some interventions (e.g., shorter treatment regimens) might more readily achieve the target levels than others (e.g., vaccination or screening)."

As to whether this should be a mandatory additional analysis or not, I leave to the discretion of the journal editors.

We understand the reviewer's interest in alternative coverage assumptions. We have expanded our analysis to include two additional coverages – 50% and 20% – in addition to the original 80% coverage. We present detailed epidemiological and cost effectiveness results for these scenarios in Supplementary Note 4 (Sections 5 and 6) and summarise our findings briefly in the main text, as follows (p. 7):

"Costs generally scaled with coverage, while epidemiological impacts scaled non-linearly (Fig. 5), so probabilities of an intervention being cost-effective were higher at lower coverages for some interventions (Supplementary Note 4 Sections 5 and 6). Notably, implementation of vaccination, community-wide screening, or screening in prisons at 20% coverage was projected to avert more incident symptomatic TB episodes than implementation of any other intervention at 80% coverage (Fig. 4).

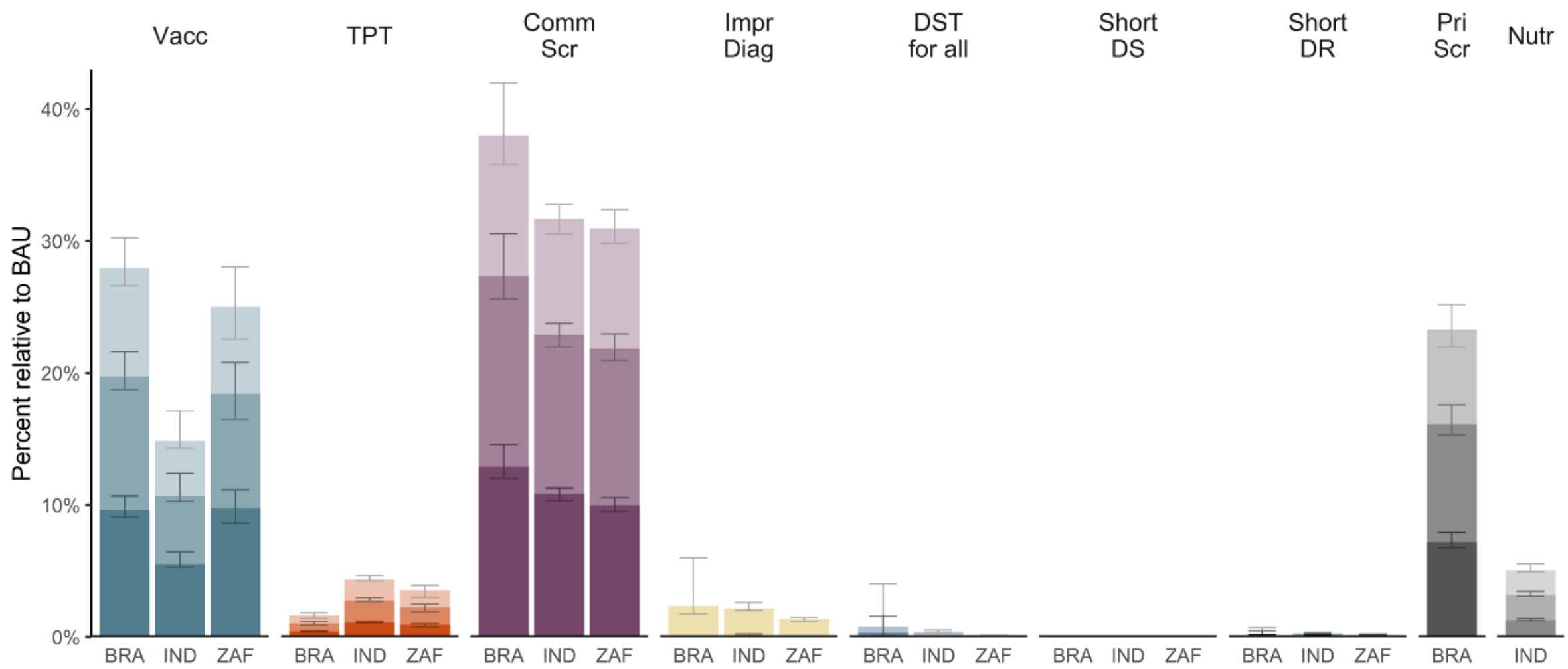

Fig. 4: Percentage of incident symptomatic TB episodes averted (2025-2050) for 80% (lightest shade), 50% (medium shade), and 20% (darkest shade) coverage. Vacc = Vaccination, TPT = Tuberculosis Preventive Treatment, Comm Scr = Community-wide screening, Impr Diag = Improved diagnosis in clinics, DST for all = Drug Susceptibility Testing for all clinic-diagnosed individuals, Short DS = Shortened treatment for Drug Susceptible TB, Short DR = Shortened treatment for Drug Resistant TB, Pri Scr = Mass screening in incarcerated individuals, Nutr = Nutritional support for households of individuals receiving TB treatment. BRA = Brazil, IND = India, ZAF = South Africa. Error bars reflect 95% Uncertainty Intervals."

Reviewer #3 (Remarks to the Author):

Thank you for addressing the comments succinctly and sufficiently.

We thank the reviewer for their time and consideration.

Reviewer #4 (Remarks to the Author):

I appreciate the opportunity to review the authors' thoughtful responses to the comments. The authors have added clarity around many of the issues identified by the other reviewers and me. There are two issues that warrant further consideration for this manuscript to attain its maximum potential to inform policy and decision making. Each of these issues was identified by me and at least one other reviewer.

We appreciate the reviewer's time and consideration to re-review our manuscript and are pleased that we have been able to add clarity.

First, the author's assumption of 80% coverage across all interventions limits interpretability for decision makers, who need to determine how much effort and resources to commit to each intervention. As the authors note, multiple interventions could be adopted in parallel, but it is unlikely that each intervention would attain the identical 80% coverage (and then why not just assume 100% coverage across all interventions?). Analyzing different coverage levels of interventions (at least the particularly impactful ones like screening and vaccination) would enable decision makers to see the relative epidemiologic, clinical, and budget impact and cost-effectiveness of coverage combinations like 80% screening/40% vaccination versus 40% screening/80% vaccination versus 60% screening/60% vaccination. It is not obvious that changing the coverage level would be a simple linear scaling up/down in the model. Adding these comparisons would help inform decision makers how to allocate limited resources. The authors seem to have done most of the hard work in completing this methodologically robust and important analysis, and it would be a shame if different coverage levels were not added; these additions seem less complex than what they have already done.

We understand the reviewer's interest in alternative coverage assumptions. We have expanded our analysis to include two additional coverages – 50% and 20% – in addition to the original 80% coverage. We present detailed epidemiological and cost effectiveness results for these scenarios in Supplementary Note 4 (Sections 5 and 6) and summarise our findings briefly in the main text, as follows (p. 7):

"Costs generally scaled with coverage, while epidemiological impacts scaled non-linearly (Fig. 5), so probabilities of an intervention being cost-effective were higher at lower coverages for some interventions (Supplementary Note 4 Sections 5 and 6). Notably, implementation of vaccination, community-wide screening, or screening in prisons at 20% coverage was projected to avert more incident symptomatic TB episodes than implementation of any other intervention at 80% coverage (Fig. 4).

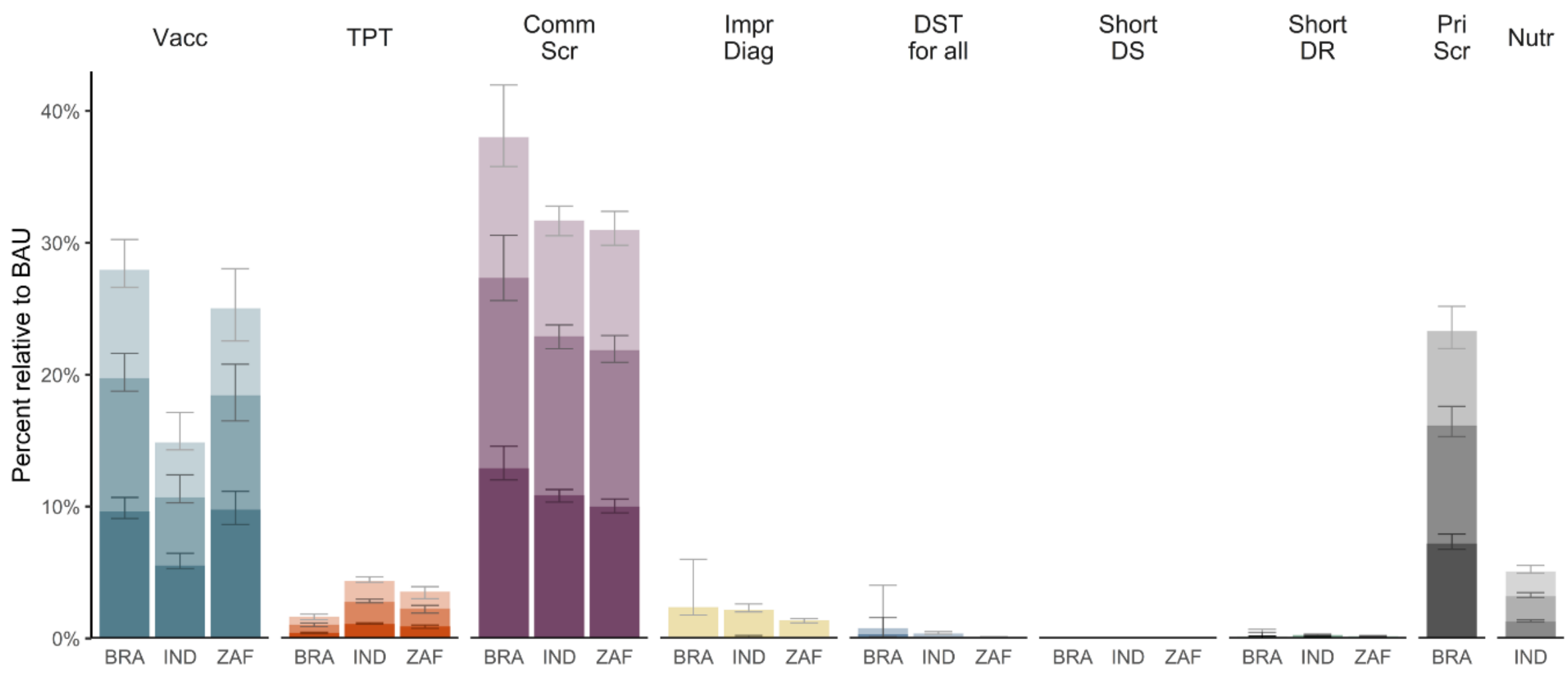

Fig. 4: Percentage of incident symptomatic TB episodes averted (2025-2050) for 80% (lightest shade), 50% (medium shade), and 20% (darkest shade) coverage. Vacc = Vaccination, TPT =

Tuberculosis Preventive Treatment, Comm Scr = Community-wide screening, Impr Diag = Improved diagnosis in clinics, DST for all = Drug Susceptibility Testing for all clinic-diagnosed individuals, Short DS = Shortened treatment for Drug Susceptible TB, Short DR = Shortened treatment for Drug Resistant TB, Pri Scr = Mass screening in incarcerated individuals, Nutr = Nutritional support for households of individuals receiving TB treatment. BRA = Brazil, IND = India, ZAF = South Africa. Error bars reflect 95% Uncertainty Intervals."

Second, given the importance of TPT and of HIV in this analysis (especially in South Africa for HIV), the authors could provide an analysis of improved uptake and completion of TPT among people with HIV, or a justification within the manuscript for why such an analysis was not pursued given its prioritization by WHO. There are some data around TPT uptake among people with HIV:

- WHO Global Tuberculosis Report 2023: 49% of people with HIV in South Africa who started TPT completed TPT (see Section 3.3 and Figure 3.9, https://www.who.int/teams/global-programme-on-tuberculosis-and-lung-health/tb-reports/global-tuberculosis-report-2023/tb-prevention).

- WHO Global Tuberculosis Report 2025: TPT coverage among people with HIV globally was 58%. In Africa, among those newly initiated ART, TPT coverage was 63% (Section 3.3, https://www.who.int/teams/global-programme-on-tuberculosis-and-lung-health/tb-reports/global-tuberculosis-report-2025/tb-prevention-and-screening).

We appreciate the reviewer highlighting the potential importance of TPT among individuals living with HIV in South Africa and understand that there would be interest in such analysis. However, we did not include that analysis here because of the high TPT initiation rates recently achieved among people living with HIV. We have added our reasoning for not including this analysis in the main text (p. 12), as follows:

> *"We did not model a separate intervention for South Africa as the key intervention addressing HIV (wide availability of ART) is already implemented, nor did we model TPT for people living with HIV given high initiation rates recently achieved in South Africa." [REF 1 in paper]*
